# Positive selection-driven fixation of a hominin-specific amino acid mutation related to dephosphorylation in *IRF9*

**DOI:** 10.1186/s12862-022-02088-5

**Published:** 2022-11-10

**Authors:** Jianhai Chen, Xuefei He, Ivan Jakovlić

**Affiliations:** 1 Institutes for Systems Genetics, Frontiers Science Center for Disease-Related Molecular Network, West China Hospital, Sichuan University, Chengdu, 610041 China; 2 State Key Laboratory of Grassland Agro-Ecosystems, and College of Ecology, Lanzhou University, Lanzhou, 730000 China; 3 Bio-Transduction Lab, Wuhan, China

**Keywords:** Protein phosphorylation, Positive selection, Purifying selection, Molecular adaptation, *IRF9*

## Abstract

The arms race between humans and pathogens drives the evolution of the human genome. It is thus expected that genes from the interferon-regulatory factors family (IRFs), a critical family for anti-viral immune response, should be undergoing episodes of positive selection. Herein, we tested this hypothesis and found multiple lines of evidence for positive selection on the amino acid site Val129 (NP_006075.3:p.Ser129Val) of human *IRF9*. Interestingly, the ancestral reconstruction and population distribution analyses revealed that the ancestral state (Ser129) is conserved among mammals, while the derived positively selected state (Val129) was fixed before the “out-of-Africa” event ~ 500,000 years ago. The motif analysis revealed that this young amino acid (Val129) may serve as a dephosphorylation site of *IRF9*. Structural parallelism between homologous genes further suggested the functional effects underlying the dephosphorylation that may affect the immune activity of *IRF9*. This study provides a model in which a strong positive Darwinian selection drives a recent fixation of a hominin-specific amino acid leading to molecular adaptation involving dephosphorylation in an immune-responsive gene.

**Supplementary Information:**

The online version contains supplementary material available at 10.1186/s12862-022-02088-5.

## Introduction

How positive Darwinian selection has shaped the evolution of immune-related genes has long been spotlighted. The never-ending arms race between pathogens and immune system-related genes is one of the most long-lasting forces to shape the infection susceptibility of humans [1, 2]. Indeed, a genomic scanning of primate species revealed that the most enriched pathways with positively selected genes in the human genome are related to the immune system [3], and it was estimated that over 30% of adaptive protein changes in the human genome were driven by virus-human interactions over millions of years [4]. This is not limited to humans; a study incorporating data from birds and mammals suggested a general pattern of positive selection on immune-related genes [5].

In mammals, the interferon regulatory factors (IRFs), which include nine family members (Table 1), are particularly essential due to the complex regulatory effects they exert on the immune cell differentiation, as well as many other aspects of innate and adaptive immune responses, especially those directed against viral infection [6]. For example, once viral RNA and DNA are detected, *IRF3*, *IRF5*, and *IRF7* are activated to coordinately generate type I interferons downstream of pathogen recognition receptors [7]. *IRF4*, *IRF8*, and *IRF5* can regulate myeloid cell activity to regulate inflammatory responses [8]. *IRF9* can cooperate with unphosphorylated STAT to regulate interferon-driven gene expression [9]. One of the common features of IRFs is the presence of serine residues which are phosphorylated to regulate the protein activity [10]. Previous studies have revealed positive selection signals in specific sites of several IRF genes (*IRF3*, *IRF5*, and *IRF9*) in multiple vertebrate species [11], but it remains unknown if and which IRF genes and sites are positively selected in humans. In addition, it also remains unknown whether there exists a relationship between the site-level positive selection and phosphorylation in human IRFs.

**Table 1:** Positive selection tests in nine IRFs genes and the Val129 site of human *IRF9* detected to be under positive selection by the Branch-site model in PAML

Gene	Gene Ensembl ID	*p*-value	*AAlen*	*GC(%)*
*IRF1*	ENSG00000125347	0.998	325	54.60%
*IRF2*	ENSG00000168310	0.995	349	50.67%
*IRF3*	ENSG00000126456	1.000	427	62.25%
*IRF4*	ENSG00000137265	0.448	451	57.74%
*IRF5*	ENSG00000128604	0.469	514	61.10%
*IRF6*	ENSG00000117595	0.996	467	53.92%
*IRF7*	ENSG00000185507	1.000	503	68.28%
*IRF8*	ENSG00000140968	1.000	426	58.24%
*IRF9*	ENSG00000213928	0.004	393	58.46%

PSS	Location in genome	Codon
V129	chr14:24,163,398–24,163,400	GTA

The *p*-values were estimated with the χ^2^ test. PSS indicates the positively selected site. AAlen and GC(%) indicate the longest length of isoforms and GC content of the human *IRF9*, respectively

In this study, we applied multiple tools to identify positive selection signals in all IRF genes in the human genome against the backdrop of a selected number of high-quality mammalian genomes. We found signals of positive selection on a specific site Val129 (NP_006075.3:p.Ser129Val) of the Interferon Regulatory Factor 9 (*IRF9*). Interestingly, Val129 is an evolutionarily young substitution of the ancestral amino acid serine (“S”), that took place in a common ancestor of humans, Neanderthals, and Denisovans some ~ 500,000 years ago. The motif modelling analysis revealed that this change may have resulted in the loss of a phosphorylation site. We propose that advantages underlying the molecular adaptation of this dephosphorylation may potentially correlate with the elevation and extension of immune activity of *IRF9*.

## Results

### The branch-site model and multiple tools revealed that a site (Val129) of *IRF9* is under a significant positive selection

Under the phylogenetic framework of orthologous genes across multiple mammalian species (Additional file 1), covering phylogenetic clades of Primates, Euarchontoglires, Boreoeutheria, Eutheria, and Theria, we tried to identify positively selected sites in IRF genes in the human lineage. We first set the human branch as the foreground (test branch) in the PAML’s branch-site model. Among the nine genes, we identified a significant signal in *IRF9* even after the rigorous Bonferroni correction for multiple tests (*p* = 0.004 < 0.05/9). The site Val129 in *IRF9* exhibited the highest probability of positive selection (NEB, probability = 0.992) (Table 1). This substitution is located in the fourth exon of *IRF9* in the chromosome 14 of the human genome (hg38). Herein, if not otherwise specified, we termed the substitution “Val129” for convenience. The distributions of amino acid lengths and GC contents indicated that *IRF9* is not an outlier among the IRF genes, suggesting that the significant signal of *IRF9* should not be due to its sequence features. In addition, no signal of recombination was detected in the human *IRF9* using SimPlot [12] (Additional file 2). This confirms that recombination was not mistakenly identified as the positive selection in human *IRF9*.

**Fig. 1:**
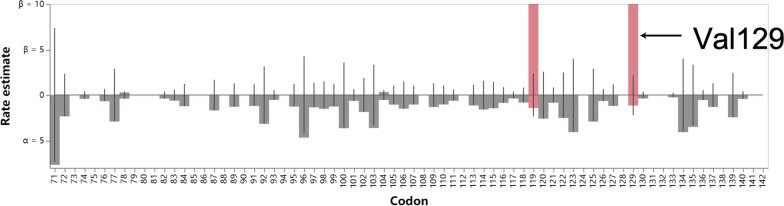
The FEL method identified positive selection on Val129 (shown with black arrow). The red bars indicate positively selected sites. Maximum likelihood estimates of synonymous (α) and non-synonymous rates (β) at each site are shown as bars. The base line shows the estimates under the null model (α = β)

Despite the significant signal based on the brach-site model, due to the relatively low accuracy of NEB method, we set out to further corroborate the identification of the site Val129 using multiple independent tools and methodologies [13]. These tests include several tools in the HyPhy package: aBSREL, MEME and FEL. The aBSREL method found evidence of episodic positive diversifying selection on the human branch (*p* = 0.0114), after correcting for multiple testing with the Holm-Bonferroni correction (Additional file 3). The MEME method confirmed that Val129 is under positive selection (LRT, *p* = 0.00291, Additional file 4). The FEL method further found statistically significant evidence of diversifying selection on three sites, one of which was Val129 (Fig. 1 and Table 2, LRT *p* = 0.000403, Additional file 5). Therefore, results are highly congruent among different algorithms, and all strongly suggest that *IRF9* site Val129 is under a significant positive selection in the human lineage.

**Table 2:** The sites of *IRF9* detected to be under significant selection by the FEL algorithm

Alignment site	α	β	α = β	LRT	p value	Class
129	1.148	383.28	2.201	16.854	0	Diversifying
340	0	116.021	0.649	10.083	0.0015	Diversifying
119	1.422	86.249	2.345	5.483	0.0192	Diversifying
377	10,000	0	53.61	4.645	0.0312	Purifying

α and β denote synonymous and non-synonymous rates, and LRT is the likelihood ratio test

### The secondary and tertiary structures of *IRF9* are conserved between the human and rat orthologues

**Fig. 2:**
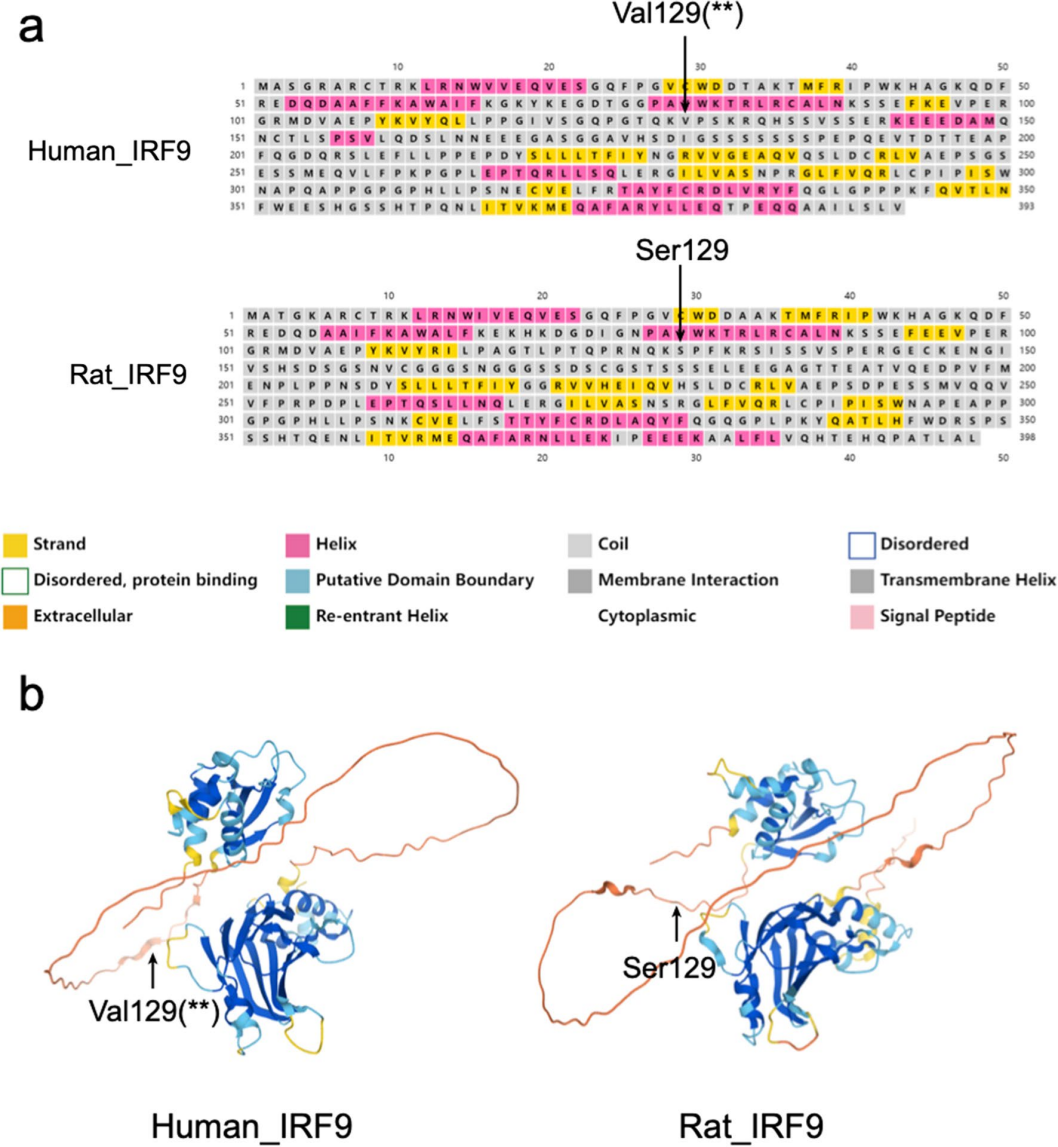
The secondary and tertiary structures of *IRF9* with a focus on Val219 detected by multiple tests as a positively selected site. **a** The secondary structure of *IRF9* and regions around the site Val219 based on the prediction of PSIPRED in human and rat orthologues. **b** The tertiary structure of *IRF9* in human and rat orthologues based on the inference by Alphafold2. The black arrows show the site inferred to be under positive selection. The two stars (**) show the probability of the prediction of PSIPRED (> 99%)

To examine whether the amino acid substitutions may have caused a change in the structure, we compared both secondary and tertiary structures between human and rat orthologues. Based on the predictions of PSIPRED and the machine learning software Alphafold2, we revealed that, despite the above-mentioned changes in the conserved sites, the secondary and tertiary structures did not change between the human and rat orthologues (Fig. 2). The site Val129 in humans and its orthologous site Ser129 in rats were both located in the coil region.

### Ancestral reconstruction revealed that the ancestral state of *IRF9* site 129 is “Ser (S)”

**Fig. 3:**
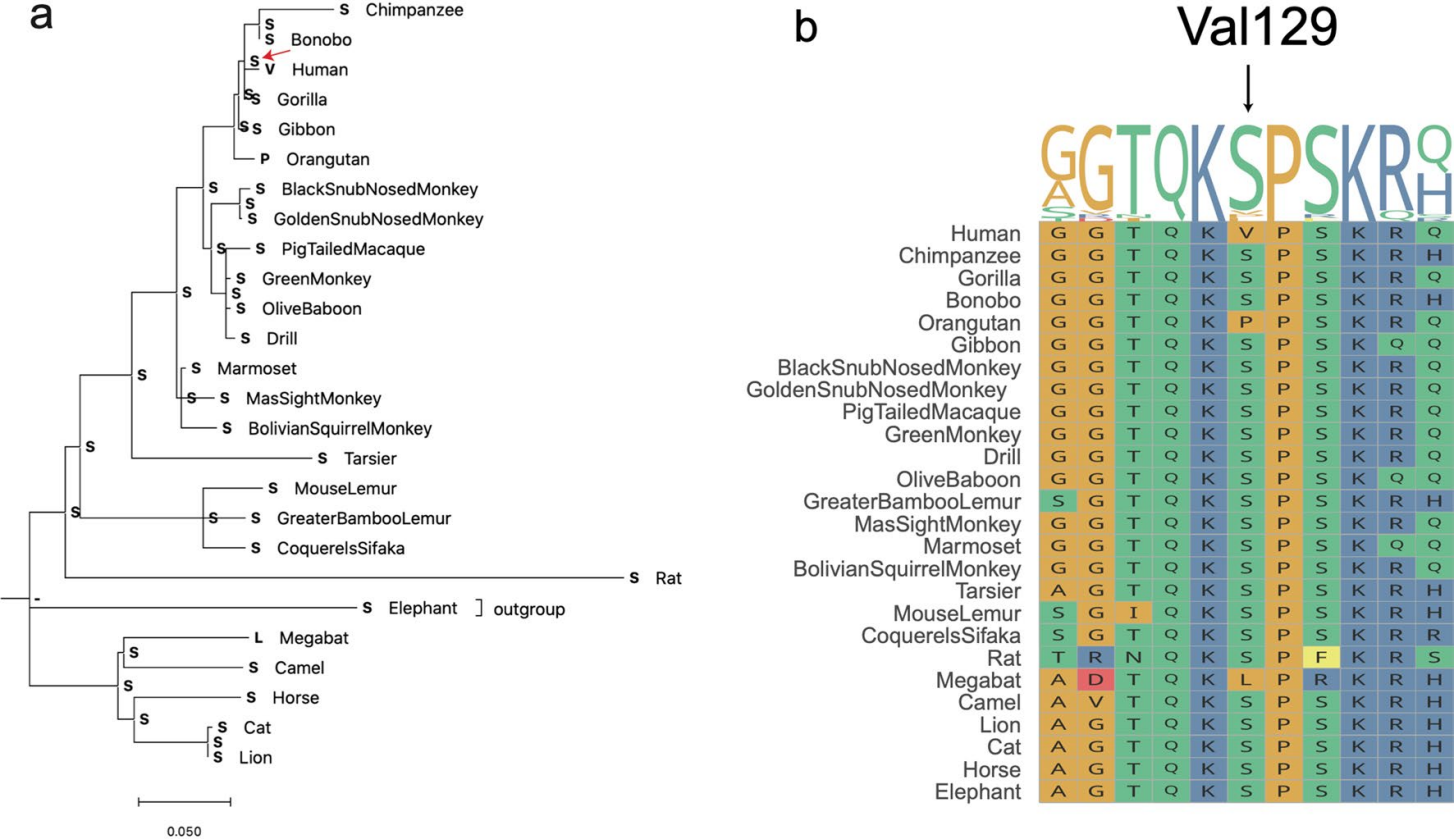
The ancestral state reconstruction and cross-species orthologous alignment. **a** Ancestral state reconstruction for human Val129 (red arrow) based on the Maximum Parsimony (MP) method in MEGA11. **b** Regional alignment comprising the human Val129 site (black arrow above the alignment)

We further recovered the ancestral state of human Val129 using the Maximum likelihood (ML) and Maximum parsimony (MP) ancestral sequence reconstruction methods implemented in PAML [13] and MEGA11 [14] respectively (Fig. 3a and Additional file 6). Both methods supported that the ancestral state of human Val129 should be Ser129. Interestingly, coding sequence (CDS) comparison indicates that the Val129 substitution involves changes of two consecutive nucleotides from “TC” to “GT”. Following the HGVS conventions (https://varnomen.hgvs.org/recommendations/DNA/variant/substitution/), the nomenclature Val129 should be assigned as NC_000014.9:g.24163398_24163399delinsGT, NM_006084.4:c.385_386delinsGT, NP_006075.3:p.Ser129Val.

We further assessed whether Val129 is conserved in non-human species (Fig. 3b). The alignment of orthologous genes also provided insights into the ancestral states of Val129. The alignment revealed that site 129 had a conserved “S” amino acid in most mammalian species included in the dataset (23/26 = 88.5%); the only outliers were extant humans (V), orangutans (P) and megabats (L). These results offer further evidence that the ancestral state of human *IRF9* Val129 should be “Ser129”.

### The ancestral Ser129 IRF9 is a phosphorylation site

To understand the potential functional effect of the evolutionary change from “S” to “V” in the alignment site of 129 in the human *IRF9* protein, we conducted a motif prediction analysis using SCANSITE 4.0. The ancestral state Ser129 was identified as a phosphorylation site (Table 3). However, the derived state Val129 (“PGTQK[V]PSKRQ”, where Val129 is emphasized using brackets) is not a phosphorylation site. This suggests that this mutation resulted in a (local) dephosphorylation of IRF9. The potential kinase for the ancestral Ser129 was identified as Cyclin-dependent kinase 1 (CDK1). The ancestral core sequence around the human site 129 (“PGTQK[S]PSKRQ”) conformed to the requirements of the CDK1 consensus phosphorylation site (Ser/Thr-Pro-X-Lys/Arg) [15]. Although the human V129 site was conserved across mammals, two outgroup species, megabat and orangutan, exhibited amino acid mutations on the orthologous site, P and L, respectively (Fig. 3b). Thus, we also included these species to predict potential kinases (Table 3). Interestingly, the orthologous site of human Val129 in rats, encoding the amino acid Ser, was predicted to be a phosphorylation site. In contrast, no phosphorylation signal was found in the orthologous site of human Val129 in megabat and orangutan. This further indirectly supported that the ancestral Ser129 may have a phosphorylation state.

**Table 3:** The SCANSITE 4.0 prediction of phosphorylation sites and potential kinases around the orthologous region of the ancestral Ser129 for two outgroup species that do not have the ancestral Ser129 (see Fig. 3)

Species	*IRF9* site	Sequence	Potential kinases
Human_V129 (reference)	V129	PGTQK[V]PSKRQ	None
	T348	PKFQVTLNFWE	NEK1/NEK3
Human_V129S (ancestral)	S129	PGTQK[S]PSKRQ	CDK1/CDK5
	T348	PKFQVTLNFWE	NEK1/NEK3
Rat	S129	PRNQK[S]PEKRS	CDK1/CDK5
	T215	YSLLLTFIYGG	NEK5
	T354	PSSSHTQENLI	PRKDC
Megabat	P307	ISWSAPQAPPG	ABL1
	T352	PKFQVTLNFWE	NEK1/NEK3
Orangutan	T347	PKFQVTLNFWE	NEK1/NEK3

The amino acids highlighted by brackets correspond to the ancestral Ser129 and human Val129, while underlined amino acids show other sites with phosphorylation signals. The human pseudo-mutant to the ancestral state is named “Human_V129S”, which is a reverse mutation from Val129 to Ser129 (hereafter referred to as V129S)

### Homologous alignment of the *IRFs* family revealed a correspondence between the ancestral Ser129 of *IRF9* and Ser123 of *IRF3*

**Fig. 4:**
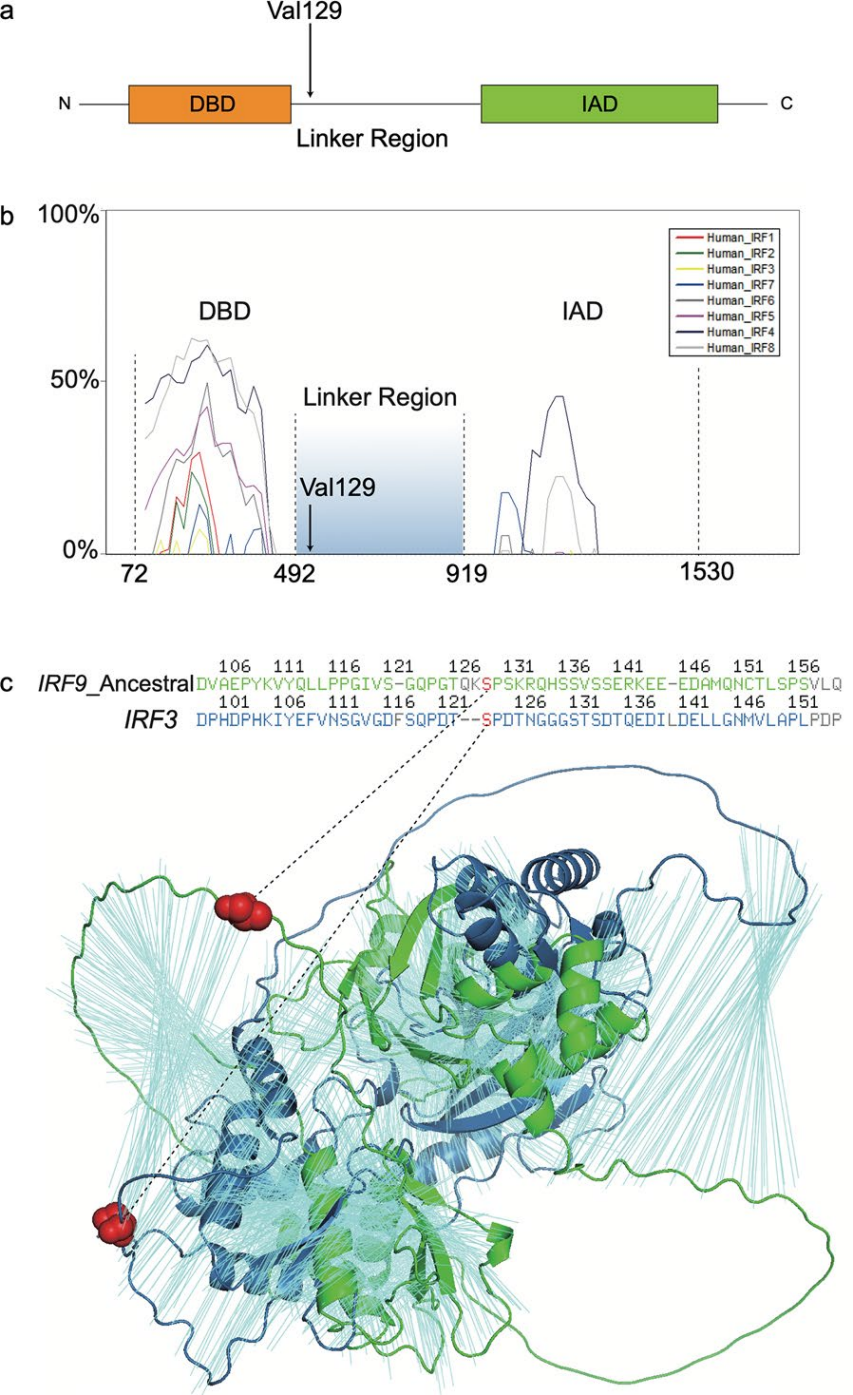
Structural similarities among homologous IRF proteins. **a** A simplified scheme of the *IRF9 domain*, showing DBD, a linker region, and IAD; **b** The nucleotide similarities between *IRF9* and other homologous genes. The positively selected site Val129 is shown with a black arrow. The horizontal axis in **b** indicates CDS coordinates. The vertical axis shows the similarity between *IRF9* and its homologs. **c** The enlarged regional structural alignment of IRF9 and IRF3 around the positively-selected site 129 of human IRF9. The 3D alignment between IRF9 and IRF3 was computed with PyMol v2.5. The 3D structures were based on Alphafold2 modeling

The IRF family has been investigated extensively. Structurally, the IAD and DBD domains were found to be conserved in all IRFs, except IRF1 and IRF2 [16] (Fig. 4a). Functionally, DBD can bind to its interferon-stimulated response element (ISRE), while IAD is responsible for binding with the signal transducer and activator of transcription 2 (STAT2) [17, 18]. In addition, *IRF3* is one of the most well-characterized transcription factors involving innate immune responses [19]. Here, we tried to understand the structural differences between the homologous proteins within the *IRF* family with a focus on the positively selected site (Val129) in *IRF9.*

The homologous alignment of nine human IRF proteins revealed that common structural features, the DBD (DNA Binding Region), IAD (IRF Associated Domain), and the linker region, were consistently aligned among these proteins (Fig. 4b). The similarities between these proteins were much higher in the DBD and IAD regions than in the linker region. The human Val129 lies within the linker region between the DBD and IAD (Fig. 4b). To recover the corresponding amino acid for the ancestral Ser129 of IRF9 in homologous genes, we explored three alignment methods: the Ensembl database alignment with CLUSTAL W [20], local alignment with MAFFT [21], and tertiary structural alignment (3D) with PyMol [22] (Fig. 4c). The CLUSTAL_W alignment revealed that, among all other IRFs, from IRF1 to IRF8, only IRF3 had an identical amino acid “S” corresponding to the ancestral state of site 129 of the human IRF9 (Additional file 7). In addition, both the secondary structure and tertiary alignment revealed that the ancestral state of site 129 of IRF9 (“S”) is a counterpart to the amino acid “S” of the IRF3 site 123 (Fig. 4c). These sequential and structural correspondences are consistent with the long-held evolutionary theory that gene duplication is a predominant force for the origination of homologous gene [23–25]. In addition, this homologous inference offers further indirect support to the hypothesis that the ancestral state of the site 129 in human IRF9 is Serine, as well as for the independent origin of the derived state Val129*.*

Interestingly, a previous study revealed that the Ser123 in IRF3 is related to the immune regulation of phosphorylation [26]. Indeed, there is abundant evidence for the role of serine in phosphorylation, as a regulatory mechanism in bacteria [27] and mammals [28, 29]. Thus, considering the two findings here, (1) the predicted phosphorylation function in the ancestral Ser129 of human IRF9 and (2) the structural correspondence between the Ser129 of IRF9 and Ser123 of IRF3, we propose that positive selection on the evolutionary change from Ser to Val in the human IRF9 site 129 might be related to the molecular adaptation involving the absence of phosphorylation.

### The evolutionary origin and population allele frequency of Val129

Based on the human population RNAseq data (GTEX), we found that the top two highest expression organs/tissues/cells of *IRF9* are the spleen and EBV-transformed lymphocytes (Additional file 8). Interestingly, based on the GTEx database, *IRF9* is expressed higher than other *IRF gene*s in the spleen. To further understand whether there are polymorphisms at the site of Val129 in human populations, we examined the gnomAD database (v3.1), which has curated allele frequency data for 76,156 individuals. We found that there is no “S” type in any human population. This result suggests that Val129 should have been fixed before the “out-of-Africa” event. To identify the evolutionary stage when the change from “S” to “V” occurred, we manually assessed the mapping results of archaic Denisovan and Neanderthal genomic reads publicly available in the UCSC browser against the human genome. We found that Val129 was shared among all three *Homo* species (Additional file 9). It is known that the three Homo species (*H. neanderthalensis* and Denisova people) diverged at ~ 0.5 Mya [30]. Thus, we propose that this mutation was most probably fixed at least half a million years ago.

## Discussion

IRF genes are critical for complex immune processes, including the anti-viral responses. Previous studies have revealed signals of positive selection on vertebrate IRFs (*IRF3*, *IRF5*, and *IRF7*) [11], but we still do not know whether there are positively selected sites in human IRF genes. In addition, we also do not know whether phosphorylation sites in IRFs [10] can be selected to influence the regulation of IRF proteins. Among the nine human IRF genes, we found that at least two sites in *IRF9* are evolving under a positive Darwinian selection. These results were supported by several different methods and models (branch-site model and HyPhy methods), supporting the reliability of our results.

We found a particularly interesting pattern for the site Val129. Evolutionary alignment across species and ancestral sequence reconstruction support “S” as the ancestral state of Val129, suggesting a selective sweep resulting in the replacement of “S” with “V”. As Neanderthals and Denisovans both possess the mutant allele, this indicates that fixation of “V” most probably took place in hominin lineages more than half a million years ago (an alternative but less likely hypothesis would be that it was independently fixed in two or three hominin lineages). Though evolutionarily conserved, the “S” site is located within the random coil region of IRF9 (supported by both secondary and tertiary structure predictions). A study on *Drosophila* has revealed that random coil structure is the preferred hotspot for positive selection [31]. Our analyses support their findings and indicate that mutations in the random coil region of IRF proteins may contribute to the adaptive evolution of human immune-responsive genes.

**Fig. 5:**
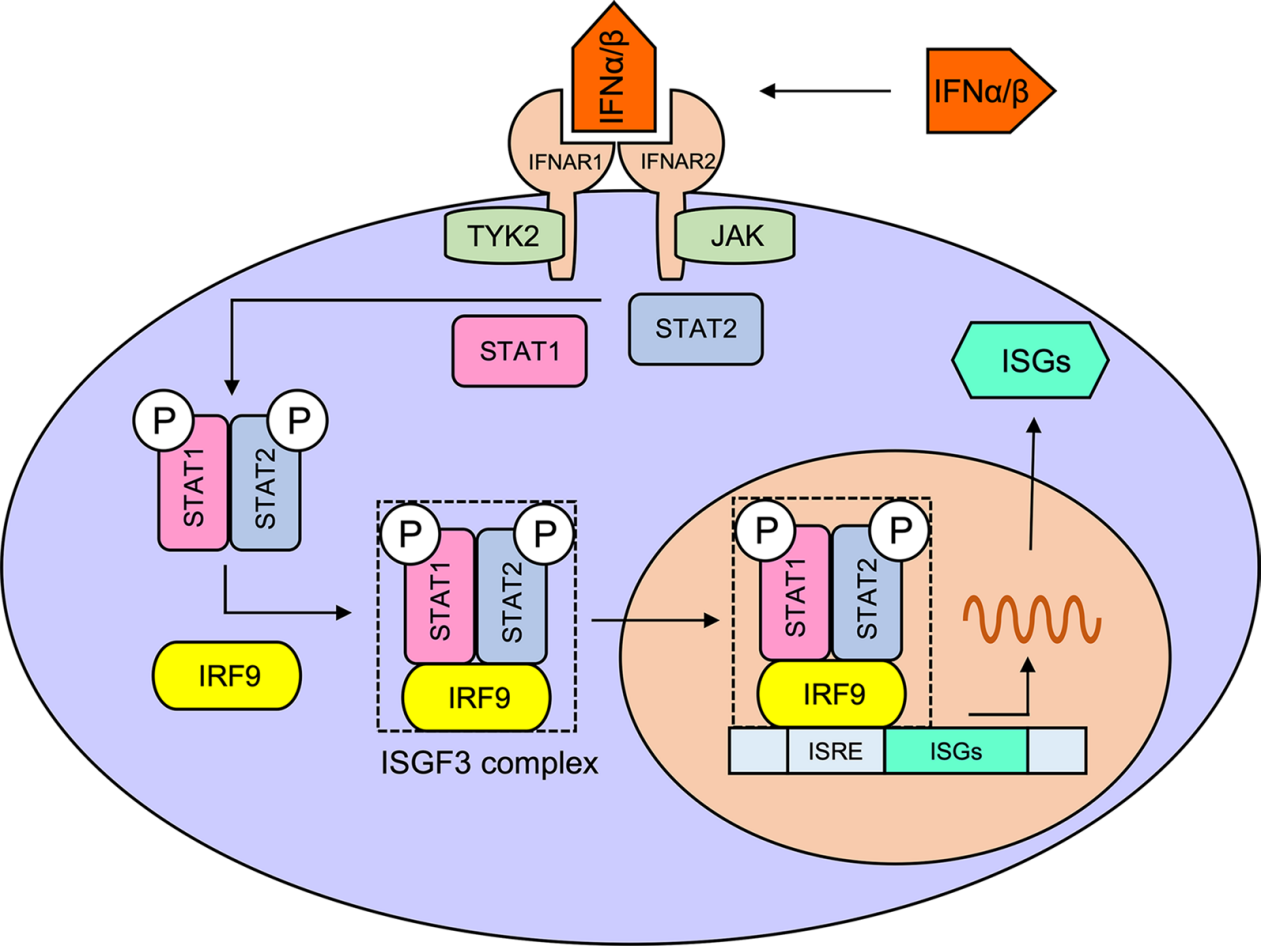
Schematic molecular processes of the type I IFN signalling based on a literature review. The abbreviations and full names are IFNAR1 (Interferon Alpha and Beta Receptor Subunit 1), IFNAR2 (Interferon Alpha and Beta Receptor Subunit 2), TYK2 (Tyrosine Kinase 2), JAK (Janus kinase), STAT1 (Signal Transducer and Activator Of Transcription 1), STAT2 (Signal Transducer And Activator Of Transcription 2), ISRE (IFN stimulated response elements), and ISGs (interferon-stimulated genes)

IRF9, the interferon regulatory factor 9, is a transcription factor critical for mediating the type I interferon antiviral immunity. It is associated with the human disease phenotype that was recognised as the Immunodeficiency 65 Viral Infections. Specifically, the inherited IRF9 deficiency is related to a life-threatening influenza pneumonitis in early infancy [32] and impaired control of multiple viral infections [33]. We summarized the *IRF9*-related processes inferred via a literature review in Fig. 5. Briefly, the canonical type I IFN signalling initiates from the binding of type I IFNs (IFN-alpha and IFN-beta) to two types of cell surface transmembrane receptors: IFNAR1 and IFNAR2. This binding results in the activation of two cytoplasmic Jak kinases, TYK2 and JAK1*,* to further phosphorylate two transcription factors, STAT1 and STAT2. The ISGF3, a transcriptional activator with a multi-subunit structure, is then formed via an interaction between the phosphorylated STAT1:STAT2 dimer and IRF9. IRF9 can facilitate the DNA binding activity of the ISGF3 complex to stabilize the complex with the aid of STAT1. The transcriptionally active ISGF3 then enters the nucleus and directly binds to the promoter regions of IFN-stimulated response elements (ISRE) [10]. This binding process can then activate the transcription of interferon-stimulated genes (ISGs) [34], thereby triggering the full-blown antiviral response in the cell [35, 36] (Fig. 5). The type I IFN signalling can effectively stimulate the transcription of IRF genes (*IRF1* to *IRF9*), thereby serving as a positive feedback-amplifier circuit [34].

Herein, based on the motif prediction, we found that the ancestral state “S” is located within a linker region and that it is a conserved putative phosphorylation site across multiple species. Interestingly, the alignment between IRF3 and IRF9 revealed that the IRF9 ancestral state Ser129 can be aligned to the IRF3 Ser123. In Eukaryotes, serine (S), threonine (T), and tyrosine (Y) residues are the most commonly used phosphorylation sites [26, 37, 38]. Through the linker region phosphorylation, the EBV-encoded kinase, BGLF4, can down-regulate the IRF3 transactivation [26]. In contrast, a phosphorylation-defective mutant, IRF3_S123A, showed higher activity [26]. In addition, Glycogen synthase kinase 3 (GSK3) inhibitor can enhance the activity of IRF3 [26]. Recent studies revealed that unphosphorylated STAT2 (U-STAT2), IRF9, and U-STAT1 can form unphosphorylated ISGF3, which can prolong and sustain resistance to virus infection and DNA damage [34]. Thus, we propose that the loss of the phosphorylation site that was a consequence of the evolutionary change from “S” to “V” may have resulted in the molecular adaptation of transactivation activity in *IRF9*.

Protein phosphorylation is an evolutionarily ancient process in all living organisms ranging from prokaryotes to eukaryotes [39]. Despite the deep conservation of phosphoproteins, phosphorylation sites can evolve rapidly to enable phenotypic plasticity and diversity [40]. This makes protein phosphorylation a rapid and versatile mechanism to drive signal tuning and protein regulation. The S to V amino acid mutation in IRF9 was fixed during the hominin evolution due to positive selection. The potential advantage of the novel form could be linked to the loss of phosphorylation, which might enhance and prolong the transactivation activity during the anti-viral immune response. Therefore, this study putatively provides an elegant case of positive Darwinian selection underlying the fixation of a young amino acid leading to molecular adaptation of dephosphorylation for immune response.

## Conclusions

IRFs are critical transcription factors for numerous immune activities, including anti-viral responses. However, it is still unknown if any and which genes and sites are positively selected in the human lineage. In this study, we identified an evolutionary young amino acid (Val129) in *IRF9* with a significant signal of positive selection (a change from S to V). Based on the homologous parallelism, the change may be associated with protein dephosphorylation. Thus, the adaptive change from Ser129 to Val129 in hominids was possibly driven by its different transactivation activity during the immune response. To our knowledge, this is the first report to link positive selection on a specific human gene site with potential phosphorylation regulation.

## Materials and methods

### Selective pressure analyses based on the branch-site model, MK test, and HyPhy package

The coding sequences for IRFs covering multiple mammalian species were retrieved from Ensembl (v105). Based on the Ensembl gene annotation, we used only the genes annotated as “one to one” interspecies orthologs with conserved gene synteny (two conserved neighboring genes). We also considered only proteins with identity values over 60% for further analyses. We aligned the coding region with TranslatorX with default parameters [41] (Additional file 1), and visualized the alignments with AliView v1.28 [42]. The phylogenetic tree was generated with FastTree v2.1 with default parameters [43]. The branch-site model in PAML was used initially to detect positive selection on both branches and sites [44]. The null hypothesis was set as “fix_omega = 1” and “omega = 1”. Statistical significance was computed using a chi-square distribution, with two times the difference in log-likelihood values and degree of freedom as the difference in the number of parameters for the two models. The identification of positive selection is often not consistent due to variances in time-frames, assumptions, methodologies, and gene conversion bias, among different methods [45, 46]. Thus, it is generally informative to seek consistent support from different algorithms. Here, for PAML branch-site model, we applied a Bonferroni correction (α = 0.05/9). We further applied a number of independent tools in the HyPhy package [47, 48] to confirm these results.

The following methods from the Hyphy packages were used: the MEME (mixed-effects model of evolution) method aims to detect individual sites under episodic positive selection or diversifying selection [49]; the FEL (fixed effects likelihood) method can be used to test which sites in a gene may be associated with adaptation to a different environment [50]; the aBSREL (adaptive branch-site random effects likelihood) method is an improved version of "branch-site" models, which models both site- and branch-heterogeneity, though it does not test for selection at specific sites [51].

### Protein structure and motifs prediction

Positively selected genes from the previous step were used to conduct further structural analyses. Differences between the secondary and 3-dimensional structures of human and rat proteins were predicted using PSIPRED 4.0 [52] and the AlphaFold2 protein structure database [53, 54], respectively. SCANSITE 4.0 was used to predict the specific sites of kinase phosphorylation and binding domains using 81 mammalian kinases/domains as the background database. The result was further filtered with stringency set at "high". The linker region and domains were then visualized manually.

### Population allele frequency, gene expression, and ancestral state reconstruction of positively selected sites

The population allele frequency can be used to understand whether a specific amino acid mutation has been fixed in human populations, which is important for evolutionary and medical studies [55]. We conducted this analysis using Genome Aggregation Database (gnomAD) 3.0 (https://gnomad.broadinstitute.org), which has curated allele frequencies for human variants [56]. We further checked whether the positively selected genes are expressed in the immune-related tissues or cells (such as the spleen, lymphocytes, and blood cells) using GTEx [57]. To trace the origin, or ancestral state, of positively selected sites, we first examined whether the sites are divergently fixed between humans and outgroups. If substitutions diverged between humans and outgroup species, we distinguished the ancestral and derived states based on phylogenetic distribution: the ancestral sites are expected to have a wider distribution across ancestral phylogenetic nodes. This intuitive method was further reexamined with the Maximum likelihood (ML) approaches in both PAML [13] and MEGA X [14]. Finally, we checked the origin branch of derived novel substitutions using the UCSC genome browser by focusing on the comparative mapped reads between the Denisovan, Neanderthal and human genomes.

## Supplementary Information

**Additional file 1. **The alignments of IRFs genes in “fasta” format. The sequences were mammalian “one to one” orthologous genes retrieved from Ensembl (v105).

**Additional file 2. **Recombination test by the SimPlot v3.5.1.

**Additional file 3. **The aBSREL (HyPhy) results after correcting for multiple testing with the Holm-Bonferroni correction.

**Additional file 4. **The MEME (HyPhy) results.

**Additional file 5.** The FEL (HyPhy) results.

**Additional file 6. ** The ML inference of ancestral sequence by the Maximum likelihood method in PAML software. The reconstructed ancestral states of mammalian species are highlighted with larger red letters.

**Additional file 7.** The CLUSTAL_W alignment of IRF proteins with a focus on the linker region around site 129 of IRF9.

**Additional file 8.** The population RNAseq expression quantification of *IRF9*. The boxplots show the expression levels within different tissues/organs/cells.

**Additional file 9. **The UCSC mapping of Denisovan and Neanderthal reads to the human genome. The red arrows show the DNA substitutions in Denisovan and Neanderthal reads. The amino acid Val129, identical among all three *Homo* species, is boxed.

## Data Availability

Finally, we checked the origin branch of derived novel substitutions using the UCSC genome browser by focusing on the comparative mapped reads between the Denisovan, Neanderthal and human genomes (https://genome.ucsc.edu/cgibin/hgTracks?db=hg19&lastVirtModeType=default&lastVirtModeExtraState=&virtModeType=default&virtMode=0&nonVirtPosition=&position=chr14%3A24632607%2D24632608&hgsid=1491727265_nE2AKk4tyRBW1y39J4aGfvCad2HM).
