# Supplementary material for: Positive selection-driven fixation of a hominin-specific amino acid mutation related to dephosphorylation in IRF9
Source: BMC Ecol Evol. 2022 Nov 10;22:132. doi: 10.1186/s12862-022-02088-5 (PMC9650800; doi:10.1186/s12862-022-02088-5)
Supplement: Supplementary file 1 — Additional file 1. The alignments of IRFs genes in “fasta” format. The sequences were mammalian “one to one” orthologous genes retrieved from Ensembl (v105). [file 12862_2022_2088_MOESM1_ESM.docx]

Additional file 1

Sequence alignments

[IRF1 1](#_Toc117418516)

[IRF2 3](#_Toc117418517)

[IRF3 5](#_Toc117418518)

[IRF4 8](#_Toc117418519)

[IRF5 11](#_Toc117418520)

[IRF6 15](#_Toc117418521)

[IRF7 18](#_Toc117418522)

[IRF8 20](#_Toc117418523)

[IRF9 23](#_Toc117418524)

# IRF1

>Rabbit_ENSOCUG00000004165

------------------------------------------------------------------------------------------------------------------------------------------------------------------------------------------------------------------------------------------------------------------------------------------------------------------------------------------------------ATGGGTCCACCCGAGCCAGATGTCAGGGGGAAAAGAGGAAGTGAGGATTTTCTCAGGCCTGGGAAGCTG---GGCAGACTCTGGCCCAAGCCCGTGGGCTCTGCGCTGACACAGCCTTGTTTGTGTGTTGCCCAGGAGGAGATGATCTTCCAGATCCCGTGGAAGCACGCCGCCAAGCACGGCTGGGACATCAACAAGGACGCCTGTCTGTTCCGGAGCTGGGCCATTCACACAGGCCGATACAAAGCTGGGGAAAAGGAGCCAGATCCCAAGACGTGGAAGGCCAACTTCCGCTGTGCCATGAACTCCCTGCCAGACATCGAGGAGGTGAAGGACCAGAGCAGGAACAAGGGCAGCTCGGCCGTGCGGGTGTACCGGATGCTCCCGCCCCTCACCAGGAACCAGAGGAAAGAGAGAAAGTCCAAGTCCACCCGAGATGCTAAGGGCAAGGCCAAGAGGAAGCAGTCCTGTGGGGATTCCAGCCCAGACACCTTCTCTGATGGACTCAGCAGCTCCACCCTGCCTGATGACCACAGTGGCTACACTGCCCAAGGCTACATTGGGCAGGACTCGGAAGTGGAACAGGCCCTCACTCCAGCACTGTCACCA---------CATAGCACTCTCCCCTCCTGG---------GGGGACATCGTGCCGGACAGCACCAGTGACCTGTACAGCTTCCAGGTGTCTCCCATGCCCTCCGCCTCTGAAGCTGCGACAGATGAGGATGAGGAAGGGAAATTACCGGAGGACATCATGAAGCTCTTGGAGCAGCCAGAGTGGCAGCCGACACATGTGGATGGGAAGGGGTACCTGCTCAATGAGCCCGGGGCCCAGGCCACTGCTGTCTACGGAGATTTCAGCTGCAAGGAGGAGCCAGAAGTTGACAGCCCTGGAGGGGACATTGGTCTGAGCCTACACCGCGTCTTCACAGATCTCAAGAACATGGACAGCAGC---TGGTTGGACAGCCTGCTG---CCCCAAGTCAGGCTG---CCTTCTATCCAGGCCATTCCTTGTGCACCGTAG

>Mouse_ENSMUSG00000018899

------------------------------------------------------------------------------------------------------------------------------------------------------------------------------------------------------------------------------------------------------------------------------------------------------------------------------------------------------------ATGCCAATCACTCGAATGCGGATGAGACCCTGGCTAGAGATGCAGATTAATTCCAACCAAATCCCAGGGCTGATCTGG------------------------------------------ATCAATAAAGAAGAGATGATCTTCCAGATTCCATGGAAGCACGCTGCTAAGCACGGCTGGGACATCAACAAGGATGCCTGTCTGTTCCGGAGCTGGGCCATTCACACAGGCCGATACAAAGCAGGAGAAAAAGAGCCAGATCCCAAGACATGGAAGGCAAACTTCCGTTGTGCCATGAACTCCCTGCCAGACATCGAGGAAGTGAAGGATCAGAGTAGGAACAAGGGCAGCTCTGCTGTGCGGGTGTACCGGATGCTGCCACCCCTCACCAGGAACCAGAGGAAAGAGAGAAAGTCCAAGTCCAGCCGAGACACTAAGAGCAAAACCAAGAGGAAG---CTGTGTGGAGATGTTAGCCCGGACACTTTCTCTGATGGACTCAGCAGCTCTACCCTACCTGATGACCACAGCAGTTACACCACTCAGGGCTACCTGGGTCAGGACTTGGATATGGAAAGGGACATAACTCCAGCACTGTCACCGTGTGTCGTCAGCAGCAGTCTCTCTGAGTGGCATATGCAGATGGACATTATACCAGATAGCACCACTGATCTGTATAACCTACAGGTGTCACCCATGCCTTCCACCTCCGAAGCCGCAACAGACGAGGATGAGGAAGGGAAGATAGCCGAAGACCTTATGAAGCTCTTTGAACAGTCTGAGTGGCAGCCGACACACATCGATGGCAAGGGATACTTGCTCAATGAGCCAGGGACCCAGCTCTCTTCTGTCTATGGAGACTTCAGCTGCAAAGAGGAACCAGAGATTGACAGCCCTCGAGGGGACATTGGGATAGGCATACAACATGTCTTCACGGAGATGAAGAATATGGACTCCATCATGTGGATGGACAGCCTGCTGGGCAACTCTGTGAGGCTGCCGCCCTCTATTCAGGCCATTCCTTGTGCACCATAG

>Rat_ENSRNOG00000008144

ATGATGAGGCGAAGTGGGCCAATGGGCGCGCGCGAGCGGCCCGGCGGGGGCGTGGCCGAGTTCGGGCCGGGGAATCCCGCTAAGTGTTTAGATTTCTTCGCGGCGCCGCCGACTCGCCAGTGCGCACTACTCCTTCGTCGAGGTAGGACGAGCTCTCACTGTCTGAGCCAAACCGAACCGGGCCGAGCTGAGCCGAGGTCAGCGGTGGCCAGAGGAACCCAGCATCTCGGGCATCATTCGCTC---------------------CGTGCACGCATCGTGTACCTACACCGCAACTCCGTGCCTCATTCCCGGGTACCCTCTGTGACTCGCTCCTGCAGCAAAGCCACCATGCCTATCACTCGGATGCGAATGAGACCCTGGCTAGAGATGCAGATTAATTCCAACCAAATTCCAGGGCTGAGCTGG------------------------------------------ATCAATAAAGAAGAGATGATCTTCCAGATCCCATGGAAGCATGCTGCCAAGCACGGTTGGGACATCAACAAGGATGCCTGTCTGTTCCGGAGCTGGGCCATTCACACAGGCCGATACAAAGCAGGGGAAAAAGAGCCAGATCCCAAGACTTGGAAGGCAAACTTCCGGTGTGCCATGAACTCCCTACCAGACATCGAGGAAGTGAAGGACCAGAGCAGGAACAAGGGCAGCTCTGCTGTACGCGTGTACCGGATGCTGCCACCCCTCACCAAGAACCAGAGGAAAGAGAGAAAGTCCAAGTCCAGCCGTGACACTAAGAGCAAAACCAAGAGGAAG---CTGTGCGGAGATTCTAGCCCTGACACCTTATCTGACGGACTGAGCAGCTCTACTCTGCCTGATGACCACAGCAGTTACACAGCTCAGGGATACCTGGGTCAGGACTTGGACATGGACAGGGACATTACCCCAGCTCTGTCACCGTGCGTCGTCAGCAGCAGTCTCTCTGAGTGGCATATGCAGATGGACATCATGCCAGACAGCACCACTGATCTGTACAACTTGCAGGTGTCGCCCATGCCCTCCACCTCTGAAGCTGCAACAGATGAGGATGAGGAAGGGAAGTTACCTGAGGACATCATGAAGCTCTTTGAACAGTCTGAGTGGCAGCCGACGCACGTGGATGGCAAGGGATACTTGCTCAATGAACCAGGAGCCCAACTCTCTACTGTCTATGGAGACTTCAGCTGCAAGGAGGAACCAGAGATCGACAGCCCTGGAGGGGACATCGAGATAGGCATACAGCGTGTCTTCACAGAGATGAAGAATATGGACCCCGTCATGTGGATGGACACCCTGCTGGGCAACTCTACCAGGCCG---CCCTCCATTCAGGCTATTCCTTGTGCACCATAA

>Cow_ENSBTAG00000031231

------------------------------------------------------------------------------------------------------------------------------------------------------------------------------------------------------------------------------------------------------------------------------------------------------------------------------------------------------------ATGCCCATCACTCGGATGCGCATGAGACCTTGGCTAGAGATGCAGATTAATTCCAACCAAATCCCAGGGCTGATCTGG------------------------------------------ATTAATAAAGAGGAGATGATCTTCCAGATCCCATGGAAGCACGCTGCGAAGCATGGCTGGGACATCAACAAGGATGCCTGTCTGTTTCGGAGCTGGGCCATTCACACAGGCCGATACAAAGCAGGGGAAAAGGAGCCGGATCCCAAGACCTGGAAGGCCAACTTTCGCTGCGCCATGAACTCGCTGCCAGATATTGAGGAGGTGAAGGACCAGAGCAGGAACAAGGGCAGTTCAGCTGTGCGGGTGTACCGGATGCTCCCACCCCTCACCAAGAGCCAGAGGAAAGAGAGAAAGTCCAAGTCCAGCCGAGACGCTAGGAGCAAGGCCAAGAAGAAG---CCATATGGGGAATACAGCCCCGATACCTTCTCTGATGGACTCAGTAGCTCCACCCTGCCTGATGACCACAGCAACTACACAGTTCGCAGCTACATGGGGCAGGACCTGGACATCGAACGGACTCTCACTCCAGCGCTGTCACCGTGTGGCGTCAGTAGCACTCTCCCTAACTGGTCCATTCCGGTGGAAATTGTGCCAGACAGCACCAGCGACCTGTACAACTTTCAGGTGTCGCCCATGCCCTCCACCTCTGAAGCTGCAACAGATGAGGACGAGGAAGGGAAATTAACTGAGGACATCATGAAGCTCTTGGAGCAAACAGGGTGGCAGCAGACAAGCGTGGATGGGAAGGGGTATCTGCTTAATGAACCTGGGGCCCAGCCCACCTCTGTCTATGGAGAGTTCAGTTGCAAGGAGGAGCCAGAAGTTGACAGCCCTGGGGGTTATATTGGCCTG------------ATATCTTCAGATATGAAGAACATGGATCCCAGC---TGGTTGGACAGCCTGCTC---ACCCCAGTCAGGCTG---CCCTCCATCCAGGCCATTCCTTGCGCACCATAG

>MouseLemur_ENSMICG00000008190

------------------------------------------------------------------------------------------------------------------------------------------------------------------------------------------------------------------------------------------------------------------------------------------------------------------------------------------------------------ATGCCCATCACTCGAATGCGCATGAGACCCTGGCTAGAGATGCAGATTAATTCCAACCAAATCCCAGGGTTGATCTGG------------------------------------------ATTAATAAAGAAGAGATGATCTTCCAGATCCCTTGGAAGCATGCTGCCAAGCATGGCTGGGACATCAACAAGGATGCCTGTCTGTTCCGGAGTTGGGCCATTCACACAGGCCGATACAAAGCAGGGGAAAAGGAGCCAGATCCCAAGACGTGGAAGGCCAACTTTCGCTGTGCCATGAACTCCCTGCCAGATATTGAGGAGGTGAAGGACCAGAGCAGGAACAAGGGCAGCTCTGCAGTGCGGGTGTACCGGATGCTCCCGCCTCTCACCAAGAACCAGAGGAAAGAGAGAAAGTCCAAGTCTAGCCGAGATGCTAAGAGCAAGGCCAAGAGGAAG---TCATATGGGGATTCCAGCCCTGATACCTTCTCTGACGGACTCAGCAGCTCCACTCTGCCTGACGACCACAGCAGCTACACTGCTCAGGGCTATGTGGGGCAGGACTTGGATGTAGAC------CTGACTCCAGCGCTGTCACCATGTGTCGTCAGTAGCACTCTCCCCGACTGGCAAATCCCGCTGGAAGTTGTGCCAGATAGCACCAGTGACCTGTACAGCTTCCAGGTGTCACCCATGCCCTCCGCCTCAGAAGCTGCCACGGATGAGGATGAGGAAGGGAAGTTAACTGATGACATCATGAAGCTTTTCGAGCAGTCGGAGTGGCAGCCGACCAGCGTGGATGGGAAGGGGTACCTGCTCAACGAACCTGGAGCCCAGCCCACTTCCATGTACGGAGAGTTCAGCTGCAAAGAGGTGTCAGACATTGACAGTCCTGGCGGGGATATCGGGCTGAGCCTACACCGTGTCTTCACAGATCTGAAGAACATGGACTCCAGC---TGGCTGGACAGCGTGCTG---CCCCCGGTCAGGATG---ACCTCCATCCAGGCCATTCCGTGTGTGCCGTAG

>Horse_ENSECAG00000017794

---------------------------------------------ATGCAGCATCTGCTGCCTAAGTGGGGCATGGTGCTTTACAGACACCATCTCTCTCTGTCACTGGACCAGCAAGAGCGCGCCACTCCTTCGTCGAGGCAGGACGTGCGCCCCAGTCGCGCGGAGCAGAGGCCAGCAGGAGCCGTGCCAAGCCCAGACCAGCGTGCCCAGCAGCCCCGCGACCCAGGCATCCTCTGCCTTCTCCCCGCTCCCACCCGGAATCGCGCTCCCCGCGCCGGCACAGCAGCCCCCGCCGCGCCGGGACCCTCAGGCGCCGCTGCCGGATCGCGCCGCTGCAGAGCCAACATGCCCATCACTCGGATGCGCATGAGACCCTGGCTAGAGATGCAGATTAATTCCAACCAAATCCCAGGGCTGATCTGG------------------------------------------ATTAATAAAGAGGAGATGATCTTCCAGATCCCATGGAAGCACGCTGCCAAGCATGGCTGGGACATCAACAAGGATGCCTGTCTGTTCCGGAGCTGGGCCATTCACACAGGCCGATACAAAGCAGGGGAAAAGGAGCCAGATCCCAAGACATGGAAGGCCAACTTTCGCTGCGCCATGAACTCCCTGCCAGACATTGAGGAGGTGAAGGACCAGAGCAGGAACAAGGGCAGCTCAGCTGTGCGGGTGTACCGGATGCTTCCACCCCTGACCAAGAACCAGAGGAAAGAGAGAAAGTCCAAGTCTAGCCGAGATGCTAAGAGCAAGGCCAAGAGGAAG---TCATGTGGGGACTCCAGCCCTGATACCTTCTCTGATGGACTCAGCAGCTCCACCCTGCCTGATGACCACAGCAGCTACACAGCTCAGAGCTACATGGGGCAGGACTTGGAGATTGAACGGGCCCTTACTCCAGTGCTGTCATCATGTGCTGTAAGTAGCACTCTCCCCGACTGGCACATGCCAGGGGAAATTGTGCCGGACAGCACCAGTGACCTGTACAGCTTCCAAGTGTCGCCTATGCCCTCCACCTCTGAAGCTGCAACAGACGAGGACGAGGAAGGGAAATTAACTGACGACATCATGAAGTTCTTGGAGCAGTCGGGGTGGCAGCCGACAAACGTGGATGGCAAGGGATACCTGCTCAATGAACCTGGGGCCCAGCCCCCCTCTGTCTATGGAGACTTCAGCTGCAAGGAGGAGACAGAAGTCGACAGCCATGGGGGGTATATTGGGCTG------------ATATCTTCAGATCTGAAGAACATGGACACCAGC---TGGCTGGACAGCCTGTTG---ACCCCAGTCAGGCTG---CCCTCCATCCAGGCCATTCCTTGTGCTCCATAG

>Dog_ENSCAFG00845010474

ATGATGGGGCGGCACCGACCAATAGGCGCCCTCGCGGCTCCTCGGGAGGCGGGGCCGGGCCTGGGTTGGGGGAATCCCGCTAAGTGTTTAGATTTCTCGCCGGCGCCGCTGCCCCGGCAGAGCGCGCCACTCCTTCGTCGAGGCAGGACGTGCGCCGGAGCCCGGCCGGGCCGAGGCCGAGGGAGCCGCGCCAAGCCAAGGCCAGCCGTGCCCCCCGCCCCTCGACCCGCGCAGCCTCTGCCTTCGCGCCGCTCCCTGCGGGATCGCGCTCGCCGCAAGGACACCGCCGCGCCAGCCGACCGGGACCCTCGGGCGCCGCGGGTGGATCGCGGCCGCTGCGGAGCCAACATGCCCATCACTCGGATGCGCATGAGACCCTGGCTAGAGATGCAGATTAATTCCAACCAAATCCCAGGGCTGATCTGG------------------------------------------ATTAATAAAGAGGAGATGATTTTCCAGATTCCATGGAAGCATGCTGCCAAGCATGGCTGGGACATCAACAAGGATGCCTGTCTGTTCCGGAGCTGGGCTATTCACACAGGTCGATACAAAGCCGGGGAAAAGGAGCCAGATCCTAAGACATGGAAGGCCAACTTTCGCTGTGCCATGAACTCCCTGCCAGATATTGAGGAAGTGAAGGACCAGAGCAGGAACAAAGGCAGCTCAGCTGTGCGGGTGTACCGGATGCTCCCACCCCTCACCAAGAACCAGAGAAAAGAGAGAAAGTCCAAGTCCAGCCGAGATGCTAAGAGCAAGGCCAAGAGGAAG---TCATGTGGTGACTCCAGTCCTGACACCTTCTCTGATGGACTCAGCACCTCCACCCTGCCCGATGACCACAGCAGCTACACAGCTCACGGCTACATGGGGCAGGATTTGGAGGTTGAGCGGGACCTTACTCCAGCATTGTCACCGTGTGCCGTTACCAGTACTCTCCCTGACTGGCACGTCCCA---GAGATAGTGCCGGACAGCACCAGTGACCTCTACAGCTTCCAGGTGTCACCTATGCCCTCCACTTCTGAAGCCACAACAGATGAGGACGAAGAAGGGAAATTAACTGAGGACATCATGAAGCTCTTGGAGCAGACGGGGTGGCAGCCGACAAACGTGGATGGCAAGGGGTACCTGCTAAATGAACCTGGGACTCAGCCCCCCTCTCTCTATGGAGACTTCAGCTGCAAGGACGAACCAGAAGTTGACAGCCCTGGGGGGTATGTTGGGCTG------------ATCTCTTCAGATCTGAAGAACATGGACACCAGC---TGGCTGGACAGCCTGTTG---ACTCCAGTCAGACTG---CCCTCCATCCAGGCCATTCCTTGTGCACCATAG

>Elephant_ENSLAFG00000007502

------------------------------------------------------------------------------------------------------------------------------------------------------------------------------------------------------------------------------------------------------------------------------------------------------------------------------------------------------------ATGCCCATCACTCGGATGCGCATGAGACCCTGGCTAGAGATGCAGATTAATTCCAACCAAATCCCAGGGCTGGTCTGG------------------------------------------ATTAATAAAGAGGAGATGATCTTCCAGATCCCGTGGAAGCATGCCGCCAAGCATGGCTGGGACATCAACAAGGACGCCTGTCTGTTCAGGAGCTGGGCCATTCACACAGGCCGATACAAAGCAGGGGAAAAGGAGACGGATCCCAAGACATGGAAAGCCAACTTTCGCTGTGCCATGAACTCACTGCCAGACATCGAGGAGGTGAAGGACCAGAGCAGGAACAAGGGCAGCTCAGCTGTGCGGGTGTACCGGATGCTCCCACCCCTCACCAAGAACCAGAGGAAAGAGAGAAAGTCCAAGTCTAGCCAAGATGCTAAGAGCAAGGCCAAGAGGAAG---TCATGTGGGGACTCCAGCCCTGACACCTTCTCTGATGGACTCAGCAGCTCCACCCTGCCCGATGACCACAGCAGCTACACAGCTCAGGGCTACTTGGGGCATGACTTGGAGGTGGAACGGGCCCTCACTCCAGCGCTTTCACCATGTGCCGTCAGTAGCACTCTCCCCGAATGGCGCATCCCAGTGGACATTCTACCTGACAGCACCAGTGACCTGTACAACTTCCAGGTGTCACCCATGCCCTCTACGTCTGAAGCTGCAACAGATGAGGATGAGGAGGGGAAACTCCCTGAGGACATCATGAAGCTCCTGGAGCAGTCGGAGTGGCAGCCGACAAGTGTGGATGGGAAGGGGTACCTGCTCAATGAGCCTGGGGTCCAGCCCTCCTCTGTCTATGGAGACTTCAGCTGCAAGGAAGAGCCCGAAGTTGACAGTGCTGGGGGGGATGTTGGGCTGAGCCTACATCGCGTCTTCACAGACCTGAAGAACATGGACTCCACT---TGGTTGGACAGTCTGCTG---CCCCCAGTCAGGCTG---CCCTCCATCCAGGCCATTCCTTGTGCACCATAA

>Tarsier_ENSTSYG00000001107

------------------------------------------------------------------------------------------------------------------------------------------------------------------------------------------------------------------------------------------------------------------------------------------------------------------------------------------------------------ATGCCCATCACTCGGATGCGCATGAGACCGTGGCTAGAGATGCAGATTAATTCCAACCAAATTCCAGGGCTGATCTGG------------------------------------------ATTAATAAAGAGGAGATGATCTTCCAAATCCCATGGAAGCATGCCGCCAAGCATGGCTGGGACATCAACAAGGACGCCTGTCTGTTCCGGAGCTGGGCCATTCACACAGGGAGATACAAAGCAGGGGAAAAGGAACCAGATCCCAAGACGTGGAAGGCCAACTTTCGTTGTGCCATGAACTCCCTGCCAGATATTGAGGAGGTGAAGGACCAGAGCAGGAACAAGGGCAGTTCAGCCGTGCGGGTGTATCGGATGCTCCCACCTCTCACCAAGAACCAGAGAAAAGAGAGAAAGTCCAAGTCCAGCCGAGATGCTAAGAGCAAGGCCAAGAGGAAG---TCATATGGAGATTCCAGTCCTGATGCTTTCTCTGATGGACTTAGCAGCTCCACCCTGCCCGATGACCACAGCAGCTACACAGCTCAGGGCTACATAGGACAGGACCTGGAGGTGGAGCCAGGCCTGACTCCAGCACTGTCACCGTGTGCCGTCAGTAGCACTCTCCCTGACTGGCACATCCCAGTGGAAGTTGTGCCAGACAGCACCAGTGACCTATACAACTTCCAGGTGTCACCCCTGCCTTCTGCCTCTGAAGCTGCAACAGATGAAGATGAGGAAGGGAAACTACCTGACGACATCATGAAGCTCTTGGAGCAGTCAGAGTGGCAGCCGACAAATGTGGATGGGAAGGGATACCTGCTCAATGAACCTGGGACCCAGCCCACCTCTGTCTATGGAGACTTCAACTGCAAGGAGGAGCCAGAAGTTGACAGTCCTGGGGGGGATATT---GTGAGCCTACACCGCGTCTTCACAGATCTGAAGAACATGGACACCAGC---TGGCTGGACAATCTGCTG---CCCCCAGTCAGGCTG---CCCTCCATCCAAGCCATTCCTTGTGCGCCGTAG

>Gibbon_ENSNLEG00000012720

------------------------------------------------------------------------------------------------------------------------------------------------------------------------------------------------------------------------------------------------------------------------------------------------------------------------------------------------------------ATGCCCATCACTCGGATGCGCATGAGACCCTGGCTAGAGATGCAGATTAATTCCAACCAAATCCCGGGGCTCATCTGG------------------------------------------ATTAATAAAGAGGAGATGATCTTCCAGATCCCATGGAAGCATGCTGCCAAGCATGGCTGGGACATCAACAAGGATGCCTGTTTGTTCCGGAGCTGGGCCATTCACACAGGCCGATACAAAGCAGGGGAAAAGGAGCCAGATCCCAAGACGTGGAAGGCCAACTTTCGCTGTGCCATGAACTCCCTGCCAGATATCGAGGAGGTGAAGGACCAGAGCAGGAACAAGGGCAGCTCAGCTGTGCGAGTGTACCGGATGCTTCCACCTCTCACCAAGAACCAGAGAAAAGGTATCCAAGGACTCTGGGTCCTTGGG---AACTGCTTTCTTCCTCGACAG---TCATGTGGGGATTCCAGCCCTGATACCTTCTCTGATGGACTCAGCAGCTCCACTCTGCCTGATGACCACAGCGGCTACACAGTTCCAGGCTACATG---CAGGACTTGGAGGTGGAGCGGGCCCTGACTCCAGCACTGTCGCCGTGTGCTGTCAGCAGCACTCTCCCTGACTGGCACATCCCAGTGGAAGTTGTGCCGGACAGCACCAGTGATCTGTACAACTTCCAGGTGTCACCCATGCCCTCCACCTCTGAAGCTACAACAGATGAGGATGAGGAAGGGAAATTACCTGAGGACATCATGAAGCTCTTGGAGCAGTCGGAGTGGCAGCCAACAAACGTGGATGGGAAGGGGTACCTACTCAATGAACCTGGAGTCCAGCCCACCTCTGTCTATGGAGACTTTAGCTGTAAGGAGGAGCCAGAAATTGACAGCCCAGGGGGGGATATTGGGCTGAGTCTACAGCGTGTCTTCACAGATCTGAAGAACATGGATGCCACC---TGGCTGGACAGCCTGCTG---ACCCCAGTCCGGTTG---CCCTCCATCCAGGCCATTCCCTGTGCACCGTAG

>MasNightMonkey_ENSANAG00000036604

------------------------------------------------------------------------------------------------------------------------------------------------------------------------------------------------------------------------------------------------------------------------------------------------------------------------------------------------------------ATGCCCATCACTCGGATGCGCATGAGACCCTGGCTAGAGATGCAGATTAATTCCAACCAAATCCCGGGGCTCATCTGG------------------------------------------ATTAATAAAGAGGAGATGATCTTCCAGATCCCATGGAAGCATGCTGCCAAGCATGGCTGGGACATCAACAAGGATGCCTGTTTGTTCCGGAGCTGGGCCATTCACACAGGTCGATACAAAGCAGGGGAAAAGGAGCCAGACCCCAAGACATGGAAGGCCAACTTTCGCTGTGCCATGAACTCCCTGCCAGATATTGAGGAGGTGAAAGACCAGAGCAGGAACAAGGGCAGCTCAGCTGTGCGGGTATACCGGATGCTTCCACCTCTCAACAAGAACCAGAGAAAAGAAAGAAAGTCGAAGTCCAGCCGAGATGCTAAGAGCAAGGCCAAGAGGAAG---TTATGTGGGGATTCCAGCCCTGATACCTTCTCTGATGGACTCAGCAGCTCCACTCTGCCTGATGACCACAGCAGCTATACAACTCCGGGCTACATG---CAGGACTTGGAGGTGGAGCGGGCCCTGACTCCAGCACTGTCGTCCTGTGGTGTCAGCAGCACTCTCCCTGACTGGCACATCCCAGTGGAAGTTGTGCCGGACAGCACCAGTGATCTGTACAACTTCCAGGTGTCACCCATGCCCTCCACCTCTGAAGCTGCAACAGATGAGGATGAGGAAGGGAAATTACCTGAGGACATCATGAAGCTCTTGGAGCAGTCAGAGTGGCAGCCGACAAACGTGGATGGGAAGGGGTACCTACTCAATGAACCTGGAGTCCAGCCCACCTCTGTCTATGGAGACTTTAGCTGCAAGGAGGAGCCAGAAGTTGACAGCCCAGGGGGTGATATTGGGCTGAGTCTACAGCGCGTCTTCACAGATCTGAAGAACATGGATACCACC---TGGCTGGACAGCCTGTTT---ACTCCAGTCCGGTTG---CCCTCCATCCAGGCTATTCCCTGTGCACCGTAG

>Marmoset_ENSCJAG00000013013

------------------------------------------------------------------------------------------------------------------------------------------------------------------------------------------------------------------------------------------------------------------------------------------------------------------------------------------------------------ATGCCCATCACTCGGATGCGCATGAGACCCTGGCTAGAGATGCAGATTAATTCCAACCAAATCCCGGGGCTCATCTGG------------------------------------------ATTAATAAAGAGGAGATGATTTTCCAGATCCCATGGAAGCATGCCGCCAAGCATGGCTGGGACATCAACAAGGATGCCTGTTTGTTCCGGAGCTGGGCCATTCACACAGGCCGATACAAAGCAGGGGAAAAGGAGCCAGACCCCAAAACATGGAAGGCCAACTTTCGCTGTGCCATGAACTCCCTGCCAGATATTGAGGAGGTGAAGGACCAGAGCAGGAACAAGGGTAGTTCAGCTGTGCGGGTGTACCGGATGCTTCCACCTCTCAACAAGAACCAGAGAAAAGAAAGAAAGTCGAAGTCCAGCCGAGAT---AAGAGCAAGGTCAAGAGGAAG---TTATGTGGGGATTCCAGCCCTGATACCTTCTCTGATGGACTCAGCAGCTCCACTCTGCCTGATGACCACAGCAGCTATACAACTCCGGGCTACATG---CAGGACTTGGAGGTGGAGCGGGCCCTGACTCCAGCACTGTCGTCGTGTGGTGTCAGCAGCACTCTCCCCGACTGGCACATCCCAGTGGAAGTTGTGCCGGACAGCACCAGTGATCTGTACAACTTCCAGGTGTCACCTATGCCCTCCACTTCTGAAGCTGCAACAGATGAGGATGAGGAAGGGAAATTACCTGAGGACATCATGAAGCTCTTGGAGCAGTCAGAGTGGCAGCCGACAAACGTGGATGGGAAGGGGTACCTACTCAATGAACCTGGAGTCCAGCCCACCTCTGTCTATGGAGACTTTAGCTGCAAGGAGGAGCCAGAAGTTGACAGCCCAGGGGGTGATATTGGGCTGAGTCTACAGCGTGTCTTCACAGATCTGAAGAACATGGATACCACC---TGGCTGGACAGCCTGTTT---ACTCCAGTCCGGTTG---CCCTCCATTCAGGCTATTCCCTGTGCACCGTAG

>Macaca_ENSMMUG00000008100

------------------------------------------------------------------------------------------------------------------------------------------------------------------------------------------------------------------------------------------------------------------------------------------------------------------------------------------------------------ATGCCCATCACTCGGATGCGCATGAGACCCTGGCTAGAGATGCAGATTAATTCCAACCAAATCCCGGGGCTCATCTGG------------------------------------------ATTAATAAAGAGGAGATGATCTTCCAGATCCCATGGAAGCATGCTGCCAAGCATGGCTGGGACATCAACAAGGATGCCTGTTTGTTCCGGAGCTGGGCCATTCACACAGGCCGATACAAAGCAGGGGAAAAGGAACCAGATCCCAAGACGTGGAAGGCCAACTTTCGCTGTGCCATGAACTCCCTGCCAGATATTGAGGAGGTGAAGGACCAGAGCAGGAACAAGGGCAGCTCAGCTGTGCGAGTGTACAGGATGCTTCCACCTCTCACCAAGAACCAGAGAAAAGAAAGAAAGTCGAAGTCCAGCCGAGATGCTAAGAGCAAGGCCAAGAGGAAG---TCATGTGGGGATTCCAGCCCTGATACCTTCTCTGATGGACTCAGCAGCTCCACTCTGCCTGATGACCACAGCAGCTACACAGCTCCGGGCTACATG---CAGGACTTGGAGGTGGAGCGGGCCCTGACTCCAGCACTGTCGCCGTGTGCTGTCAGCAGCACTCTCCCTGACTGGCACATCCCAGTGGAAGTTGTGCCGGACAGCACCAGTGATCTGTACAACTTCCAGGTGTCACCCATGCCCTCCACCTCTGAAGCTGCAACAGATGAGGATGAGGAAGGGAAATTACCTGAGGACATCATGAAGCTCTTGGAGCAGTCGGAGTGGCAGCCAACAAACGTGGACGGGAAGGGGTACCTACTCAATGAACCTGGAGTCCAGCCCACCTCTGTCTATGGAGACTTTAGCTGTAAGGAGGAGCCAGAAATTGACAGCCCAGGGGGTGATATTGGGCTGAGTCTACAGCGCGTCTTCACAGATCTGAAGAACATGGATGCCACC---TGGCTGGACAGCCTGCTG---ACCCCAGTCCGGTTG---CCCTCCATCCAGGCCATTCCCTGTGCACCATAG

>GoldenSnubNoseMonkey_ENSRROG00000041744

------------------------------------------------------------------------------------------------------------------------------------------------------------------------------------------------------------------------------------------------------------------------------------------------------------------------------------------------------------ATGCCCATCACTCGGATGCGCATGAGACCCTGGCTAGAGATGCAGATTAATTCCAACCAAATCCCGGGGCTCATCTGG------------------------------------------ATTAATAAAGAGGAGATGATCTTCCAGATCCCATGGAAGCATGCTGCCAAGCATGGCTGGGACATCAACAAGGATGCCTGTTTGTTCCGGAGCTGGGCCATTCACACAGGCCGATACAAAGCAGGGGAAAAGGAACCAGATCCCAAGACGTGGAAGGCCAACTTTCGCTGTGCCATGAACTCCTTGCCAGATATTGAGGAGGTGAAGGACCAGAGCAGGAACAAGGGCAGCTCAGCTGTGCGAGTGTACCGGATGCTTCCACCTCTCACCAAGAACCAGAGAAAAGAAAGAAAGTCGAAGTCCAGCCGAGATGCTAAGAGCAAGGCCAAGAGGAAG---TCATGTGGGGATTCCAGCCCTGATACCTTCTCTGATGGACTCAGCAGCTCCACTCTGCCTGATGACCACAGCAGCTACACAGCTCCGGGCTACATG---CAGGACTTGGAGGTGGAGCGGGCCCTGACTCCAGCACTGTCGCCGTGTGCTGTCAGCAGCACTCTCCCCGACTGGCACATCCCAGTGGAAGTTGTGCCGGACAGCACCAGTGATCTGTACAACTTCCAGGTGTCACCCATGCCCTCCACCTCTGAAGCTGCAACAGATGAGGATGAGGAAGGGAAATTACCTGAGGACATCATGAAGCTCTTGGAGCAGTCGGAGTGGCAGCCAACAAACGTGGATGGGAAGGGGTACCTACTCAATGAACCTGGAGTCCAGCCCACCTCTGTCTATGGAGACTTTAGCTGTAAGGAGGAGCCAGAAATTGACAGCCCAGGGGGTGATATTGGGCTGAGTCTACAGCGTGTCTTCACAGATCTGAAGAACATGGATGCCACC---TGGCTGGACAGCCTGCTG---ACCCCAGTCCGGTTG---CCCTCCATCCAGGCCATTCCCTGTGCACCGTAG

>Human_ENSG00000125347

------------------------------------------------------------------------------------------------------------------------------------------------------------------------------------------------------------------------------------------------------------------------------------------------------------------------------------------------------------ATGCCCATCACTCGGATGCGCATGAGACCCTGGCTAGAGATGCAGATTAATTCCAACCAAATCCCGGGGCTCATCTGG------------------------------------------ATTAATAAAGAGGAGATGATCTTCCAGATCCCATGGAAGCATGCTGCCAAGCATGGCTGGGACATCAACAAGGATGCCTGTTTGTTCCGGAGCTGGGCCATTCACACAGGCCGATACAAAGCAGGGGAAAAGGAGCCAGATCCCAAGACGTGGAAGGCCAACTTTCGCTGTGCCATGAACTCCCTGCCAGATATCGAGGAGGTGAAAGACCAGAGCAGGAACAAGGGCAGCTCAGCTGTGCGAGTGTACCGGATGCTTCCACCTCTCACCAAGAACCAGAGAAAAGAAAGAAAGTCGAAGTCCAGCCGAGATGCTAAGAGCAAGGCCAAGAGGAAG---TCATGTGGGGATTCCAGCCCTGATACCTTCTCTGATGGACTCAGCAGCTCCACTCTGCCTGATGACCACAGCAGCTACACAGTTCCAGGCTACATG---CAGGACTTGGAGGTGGAGCAGGCCCTGACTCCAGCACTGTCGCCATGTGCTGTCAGCAGCACTCTCCCCGACTGGCACATCCCAGTGGAAGTTGTGCCGGACAGCACCAGTGATCTGTACAACTTCCAGGTGTCACCCATGCCCTCCACCTCTGAAGCTACAACAGATGAGGATGAGGAAGGGAAATTACCTGAGGACATCATGAAGCTCTTGGAGCAGTCGGAGTGGCAGCCAACAAACGTGGATGGGAAGGGGTACCTACTCAATGAACCTGGAGTCCAGCCCACCTCTGTCTATGGAGACTTTAGCTGTAAGGAGGAGCCAGAAATTGACAGCCCAGGGGGGGATATTGGGCTGAGTCTACAGCGTGTCTTCACAGATCTGAAGAACATGGATGCCACC---TGGCTGGACAGCCTGCTG---ACCCCAGTCCGGTTG---CCCTCCATCCAGGCCATTCCCTGTGCACCGTAG

>Gorilla_ENSGGOG00000013927

------------------------------------------------------------------------------------------------------------------------------------------------------------------------------------------------------------------------------------------------------------------------------------------------------------------------------------------------------------ATGCCCATCACTCGGATGCGCATGAGACCCTGGCTAGAGATGCAGATTAATTCCAACCAAATCCCGGGGCTCATCTGG------------------------------------------ATTAATAAAGAGGAGATGATCTTCCAGATCCCATGGAAGCATGCTGCCAAGCATGGCTGGGACATCAACAAGGATGCCTGTTTGTTCCGGAGCTGGGCCATTCACACAGGCCGCTACAAAGCAGGGGAAAAGGAGCCAGATCCCAAGACATGGAAGGCCAACTTTCGCTGTGCCATGAACTCCCTGCCAGATATCGAGGAGGTGAAAGACCAGAGCAGGAACAAGGGCAGCTCAGCTGTGCGAGTGTACCGGATGCTTCCACCTCTCACCAAGAACCAGAGAAAAGAAAGAAAGTCGAAGTCCAGCCGAGATGCTAAGAGCAAGGCCAAGAGGAAG---TCATGTGGGGATTCCAGCCCTGATACCTTCTCTGATGGACTCAGCAGCTCCACTCTGCCTGATGACCACAGCAGCTACACAGTTCCAGGCTACATG---CAGGACTTGGAGGTGGAGCGGGCCCTGACTCCAGCACTGTCGCCGTGTGCTGTCAGCAGCACTCTCCCCGACTGGCACATCCCAGTGGAAGTTGTGCCGGACAGCACCAGTGATCTGTACAACTTCCAGGTGTCACCCATGCCCTCCACCTCTGAAGCTACAACAGATGAGGATGAGGAAGGGAAATTACCTGAGGACATCATGAAGCTCTTGGAGCAGTCGGAGTGGCAGCCAACAAACGTGGATGGGAAGGGGTACCTACTCAATGAACCTGGAGTCCAGCCCACCTCTGTCTATGGAGACTTTAGCTGTAAGGAGGAGCCAGAAATTGACAGCCCAGGGGGGGATATTGGGCTGAGTCTACAGCGTGTCTTCACAGATCTGAAGAACATGGATGCCACC---TGGCTGGACAGCCTGCTG---ACCCCAGTCCGGTTG---CCCTCCATCCAGGCCATTCCCTGTGCACCGTAG

>Bonobo_ENSPPAG00000036300

------------------------------------------------------------------------------------------------------------------------------------------------------------------------------------------------------------------------------------------------------------------------------------------------------------------------------------------------------------ATGCCCATCACTCGGATGCGCATGAGACCCTGGCTAGAGATGCAGATTAATTCCAACCAAATCCCGGGGCTCATCTGG------------------------------------------ATTAATAAAGAGGAGATGATCTTCCAGATCCCATGGAAGCATGCTGCCAAGCATGGCTGGGACATCAACAAGGATGCCTGTTTGTTCCGGAGCTGGGCCATTCATACAGGCCGATACAAAGCAGGGGAAAAGGAGCCAGATCCCAAGACGTGGAAGGCCAACTTTCGCTGTGCCATGAACTCCCTGCCAGATATCGAGGAGGTGAAAGACCAGAGCAGGAACAAGGGCAGCTCAGCTGTGCGAGTGTACAGGATGCTTCCACCTCTCACCAAGAACCAGAGAAAAGAAAGAAAGTCGAAGTCCAGCCGAGATGCTAAGAGCAAGGCCAAGAGGAAG---TCATGTGGGGATTCCAGCCCTGATACCTTCTCTGATGGACTCAGCAGCTCCACTCTGCCTGATGACCACAGCAGCTACACAGTTCCAGGCTACATG---CAGGACTTGGAGGTGGAGCGGGCCCTGACTCCAGCACTGTCGCCGTGTGCTGTCAGCAGCACTCTCCCCGACTGGCACATCCCAGTGGAAGTTGTGCCGGACAGCACCAGTGATCTGTACAACTTCCAGGTGTCACCCATGCCCTCCACCTCTGAAGCTACAACAGATGAGGATGAGGAAGGGAAATTACCTGAGGACATCATGAAGCTCTTGGAGCAGTCGGAGTGGCAGCCAACAAACGTGGATGGGAAGGGGTACCTACTCAATGAACCTGGAGTCCAGCCCACCTCTGTCTATGGAGACTTTAGCTGTAAGGAGGAGCCAGAAATTGACAGCCCAGGGGGGGATATTGGGCTGAGTCTACAGCGTGTCTTCACAGATCTGAAGAACATGGATGCCACC---TGGCTGGACAGCCTGCTG---ACCCCAGTCCGGTTG---CCCTCCATCCAGGCCATTCCCTGTGCACCGTAG

# IRF2

>Elephant_ENSLAFG00000000088

------------------------------------------------------------------------------------------------------------------------------CAGGCTCCACAGGACTCTGAATCTCTCCCTGGCCACACTGTCAAGATTCAGGAAAAGAAGATTTTTCAGATCCCCTGGATGCATGCGGCTAGACATGGATGGGATGTAGAAAAAGATGCTCCACTCTTTAGGAACTGGGCGATCCACACAGGAAAGCATCAACCAGGCGTAGATAAACCTGATCCAAAAACATGGAAGGCGAATTTCCGATGTGCCATGAACTCCTTGCCCGATATTGAAGAAGTCAAGGATAAAAGCATAAAGAAGGGAAACAATGCCTTCAGAGTGTATCGAATGCTGCCCTTATCTGAACGACCTTCTAAGAAA---------------------------------------------------------------------------------------------------------------------------------------------------------------------------------------------------------------------------------------------------------------------------------GGAAAGAAAACAAAGACAGAAAAAGAAGACAGAGTTAAGCACATCAAGCAAGAACCGGTTGAATCATCTTTGGGGCTTAGTAATGGAGTGAGTGATCTTTCTCCTGAGTACGCCGTCCTGACTTCAGCTATAAAAGATGAAGTGGACAGTACGGTGAACATCATAGTTGTAGGA---CAGTCTCACCTGGGCGGCAACATTGAGGATCAAGAGATTGTCACCAACCCGCCGGACATTTGCCAAGTCGTAGAGGTGACCACTGAAAGTGATGAGCAGCCAGTCAGCATGAGCGAGCTCTACCCTCTGCAGATATCCCCTGTGTCTTCCTACGCAGAAAGCGAAACTACCGACAGTGTGCCCAGCGATGAAGAGAGCACTGAGGCA---------------------------------------------------------------------------------------------------------------------------------------GAAATAGAATGTCCG---------------GAGTGGCCAAAGCAACCA---------------------------------CCAGAAGGAAGC------------------------------------------------------------------------------

>Opossum_ENSMODG00000004393

---------------------------------------------------------------------------------------------------------------------------------------------------------------------------ATGTATGAGAAGAAGATTTTTCAGATTCCTTGGATGCATGCTGCACGACATGGGTGGGATGTTGAGAAAGATGCTCCTCTCTTTAGGAACTGGGCAATTCACACAGGAAAGCATCAACCTGGAGTAGATAAACCGGATCCCAAAACATGGAAGGCGAATTTTCGATGTGCTATGAACTCCTTACCTGATATCGAAGAAGTGAAAGATAAAAGTATAAAGAAAGGAAACAATGCCTTCAGGGTCTATAGGATGCTGCCCTTATCTGAAAGACCTTCTAAGAAAGGTAGAGAAAACTGTTCAGTGATTCACAGGGGACCCCTTAGCCCCGCACGCTCAGCATTAGCACATTCCAGAGCTCCCTTCTGTAGCCACTCCACATATAGAACCCTTCTGCTCTTAACTCACACACGTAAAATCACCTTCACTCAATCTGCCTTGGTATGGGTGAAAGAATTTGTTTGGTCCACTAAGAACTCTCAAACCTATTCAGCAGAGGGGGAATGTCTGTACTTGTATCCTTCTGGGATTATAATAAAATTACGTTTTGTCACTACAGGAAAGAAAACAAAGACAGATAAAGAAGACAGAGTTAAGCACATTAAGCAAGAACCAGTTGAGTCGTCTTTTGGGCTTAGTAATGGAATAAGTGAGCTTTCTCCTGAATACGCGGTCCTGACTTCAACTATAAAAAGCGAAGTGGATAGCACGATAAACATCATAGTTGTAGGA---CAGTCACATCTTGACAGCAACATCGAGGACCAAGTGATTGTCACTAACCCACCCGACATATGCCAAGTTGTTGAGGTGACAACAGAGAGTGATGAACAACCTGTCAGTATGAGTGAGCTGTACCCACTGCAGATTTCCCCCATCTCTTCCTACGCAGAAAGCGAAACAACAGACAGTGTGCCCAGCGATGAAGAAAATACAGAGGGACGACAACACTGGCTGAAGAGAAACATCGAAGGCAAACAGTACCTAAGTAATATGGGGACAAGAAACAGCTACCTGCTCCCCAGCATGGCAACTTTTGTGACCTCCAACAAGCCGGATCTCCAGGTTACCATCAAAGAGGAAAGTTGTCCTATGCCTTATAACAGCTCCTGGCCCATCTTCACCGACCTCCCCTTGGCATCTCCAGTCTCTCCAGCTCCCAGCAGTAGT------AGTCGGGAGACCCGGGCCAGCGTCATCAAGAAAACGTCAGATATCACCCAGTCAAGAGTCAAGAGCTGTTAA

>Cow_ENSBTAG00000010002

------------------------------------------------------------------------------ATGAAAGACACCATGCCGGTGGAAAGGATGCGTATGCGCCCGTGGCTGGAAGAACAGATCAACTCAAATATGATACCAGGGCTGAAGTGGCTTAGCAAGGAAAAGAAGATTTTTCAGATCCCCTGGATGCATGCTGCTAGACATGGGTGGGATGTGGAAAAAGATGCACCCCTCTTTAGAAACTGGGCCATCCATACAGGAAAGCATCAACCAGGAATAGATAAACCTGACCCAAAAACGTGGAAGGCGAATTTCCGATGTGCCATGAACTCTTTGCCTGACATTGAGGAAGTCAAGGACAAAAGCATAAAAAAAGGAAACAATGCCTTCAGAGTCTACCGGATGCTGCCCATGTCTGAGCGGCCTTCTAAGAAA---------------------------------------------------------------------------------------------------------------------------------------------------------------------------------------------------------------------------------------------------------------------------------GGAAAGAAACCAAAGACAGAAAAAGAAGACAGAGTGAAGCACATCAAGCAAGAACCAATTGAGTCACCTTTGGGGCTTAGTAACGGAGTAAGTGATCTTTCTCCTGAGTATGCGGTCCTGACTTCAACTATAAAAACTGAAGTGGATAGTACGGTGAACATCATAGTCGTAGGA---CAGTCACATCTGGACGGCACCTATGAGGATCAAGAGATTGTCGCTAACCCACCGGACATCTGCCAAGTTGTGGAGGTGACCACCGAGAGCGATGAACAGCCGGTCAGCATGAGTGAGCTCTACCCCCTGCAGATCTCCCCCGTGTCTTCCTACGCAGAAAGTGAAACTACGGATAGCGTGCCCAGTGACGAAGAGAGCGCGGAGGGTCGGCCACACTGGCGGAAGAGGAGCGTTGAAGGCAAACAGTACCTCAGCAACATGGGGCCGCGGAACACGTACCTACTGCCCAGCATGGCCACCTTTGTCACGTCCAACAAGCCCGACCTCCAGGTCACCATCAAAGAGGAGAGCTGCCCGGTGCCTTACAACAGCTCCTGGCCCCCGTTCCCCGACCTCCCCATCACCGCCCCCATGAGCCCCACGCCCAGCAGCAGCCGCCCAGACCGGGAGACCCGGGCCAGCGTCATCAAGAAGACGTCAGACATCACCCAGGCCCGCGTCAAGAGCTACTAA

>Rabbit_ENSOCUG00000009229

ATGGCATATGGCAGGATTTGGACAAGCAGGGATGACTTCAGACTGGATAGGAGACCAGGTGTGGAGGCTGGATCTCAGAGGAGAGGTACGATGCCGGTGGAGAGAATGCGAATGCGCCCATGGCTGGAGGAACAGATCAATTCAAACACGATACCAGGGCTAAAGTGGCTTAACAAGGAAAAGAAGATTTTCCAGATCCCCTGGATGCATGCAGCGAGACACGGGTGGGATGTGGAAAAAGATGCGCCACTCTTCAGGAACTGGGCGATCCATACAGGAAAGCATCAACCTGGAGTAGATAAACCCGATCCCAAAACATGGAAGGCAAATTTTCGATGCGCCATGAACTCCTTGCCTGATATTGAAGAAGTTAAGGATAAAAGTATAAAGAAAGGAAACAATGCCTTCAGAGTATACCGGATGCTGCCCTTGTCGGAGCGGCCTTCTAAGAAA---------------------------------------------------------------------------------------------------------------------------------------------------------------------------------------------------------------------------------------------------------------------------------GGAAAGAAACCAAAGACAGAAAAAGAAGACAGAGTTAAGCACATCAAGCAAGAACCAATTGAGTCATCTTTGGGGCTTAGTAATGGAGTAAGTGATCTTTCTTCTGAGTATGCGGTCCTGACTTCAGCTATAAAACATGAAGTGGATAGTACGGTGAACATCATAGTTGTAGGA---CAGTCCCATCTGGACAGCAGCATCGAGGATCAGGAGATTGTCACCAATCCTCCAGACATCTGCCAGGTTGTGGAGGTGACCACCGAGAGCGACGAGCAGCCAGTCAGCATGAGCGAGCTCTACCCTCTGCAGATTTCCCCCGTGTCTTCCTACGCAGAAAGCGAAACCACGGACAGCGTGCCCAGCGACGAGGAGAGCGGCGAGGCGCGACCACACTGGCGGAAGAGGAACATCGAAGGCAAACAGTACCTCAGCAACATGGGGACGCGGAGCACCTACCTGCTGCCCAGCATGGCCACCTTTGTCACCTCCAACAAGCCAGACCTGCAGGTCACCATCAAGGAGGAGAGCTGTCCCGTGCCGTACAACAGCTCCTGGCCCACCTTCCCAGACCTGCCCCTGCCCGCCGCCGTGACGCCCACGCCAGGCAGCAGC---------CGGGAGACCAGGGCCAGCGTCATCAAGAAGACGTCGGATGTCACCCAGGCCCGCGTCAAGAGCTGTTAA

>Mouse_ENSMUSG00000031627

------------------------------------------------------------------------------------------ATGCCGGTGGAACGGATGCGAATGCGCCCGTGGCTGGAGGAGCAGATAAATTCCAATACGATACCAGGGCTAAAGTGGCTGAACAAGGAGAAGAAGATTTTCCAGATCCCCTGGATGCATGCGGCTCGGCACGGATGGGACGTGGAAAAGGATGCTCCGCTCTTCAGAAACTGGGCGATCCATACAGGAAAGCATCAACCAGGAATAGATAAACCAGATCCAAAAACATGGAAAGCAAATTTTCGATGTGCCATGAATTCCCTGCCCGACATTGAGGAAGTGAAGGACAGAAGCATAAAGAAAGGAAACAACGCCTTCAGAGTCTACCGGATGCTGCCCTTATCCGAACGACCTTCCAAGAAA---------------------------------------------------------------------------------------------------------------------------------------------------------------------------------------------------------------------------------------------------------------------------------GGAAAGAAACCAAAGACAGAAAAAGAAGAGAGAGTTAAGCACATCAAGCAAGAACCAGTTGAGTCATCTTTGGGGCTTAGTAATGGAGTAAGTGGCTTTTCTCCTGAGTATGCGGTCCTGACTTCAGCTATAAAAAATGAAGTGGATAGTACGGTGAACATCATAGTTGTAGGA---CAGTCCCATCTGGACAGCAACATTGAAGATCAAGAGATCGTCACTAACCCGCCAGACATCTGCCAGGTTGTAGAAGTGACCACTGAGAGTGATGACCAGCCAGTCAGCATGAGTGAGCTCTACCCTCTACAGATTTCTCCTGTGTCTTCCTACGCAGAAAGCGAAACTACCGACAGTGTGGCCAGTGATGAAGAGAACGCAGAGGGGAGACCACACTGGAGGAAGAGGAGCATCGAAGGCAAGCAGTACCTCAGCAACATGGGGACACGGAACACCTATCTGCTGCCCAGCATGGCGACCTTTGTCACCTCCAACAAGCCAGATCTGCAGGTCACCATCAAAGAGGATAGCTGTCCGATGCCTTACAACAGCTCCTGGCCCCCATTTACAGACCTTCCCCTTCCTGCCCCAGTGACCCCCACGCCCAGCAGCAGTCGGCCAGACCGGGAGACCCGGGCCAGTGTCATCAAGAAGACATCTGATATCACCCAGGCCCGTGTCAAGAGCTGTTAA

>Rat_ENSRNOG00000009824

------------------------------------------------------------------------------------------ATGCCGGTGGAACGGATGCGAATGCGCCCGTGGCTGGAGGAGCAGATAAATTCAAATACGATACCAGGGCTAAAGTGGCTGAACAAGGAAAAGAAGATTTTCCAGATCCCCTGGATGCATGCAGCTAGACACGGTTGGGACGTAGAAAAGGATGCTCCACTCTTTAGAAACTGGGCAATCCATACAGGAAAGCATCAACCAGGAGTAGATAAACCAGATCCGAAAACATGGAAAGCAAATTTTCGATGTGCCATGAATTCCTTGCCCGACATTGAGGAAGTGAAGGACAGAAGCATAAAGAAAGGAAACAACGCCTTCAGAGTCTACCGGATGTTACCCTTATCTGAGCGACCTTCCAAGAAA---------------------------------------------------------------------------------------------------------------------------------------------------------------------------------------------------------------------------------------------------------------------------------GGAAAGAAACCAAAGACAGAGAAAGAAGAGAGAGTTAAGCACATCAAGCAAGAACCAGTTGAGTCATCTTTGGGGCTTAGTAATGGAGTAAGTGGCTTTTCTCCTGAGTATGCGGTCCTGACTTCAGCTATAAAAAATGAAGTGGATAGTACGGTGAACATCATAGTTGTAGGA---CAGTCCCATCTGGACAGCAACATTGAAGATCAAGAGATCGTCACTAACCCGCCAGACATCTGCCAGGTTGTAGAAGTGACCACCGAGAGTGATGACCAGCCAGTCAGCATGAGCGAGCTCTACCCTCTACAGATTTCTCCTGTGTCTTCCTATGCAGAAAGCGAAACTACCGACAGTGTGCCCAGCGATGAGGAGAACGCAGAGGGGAGACCACACTGGCGGAAGAGGAACATCGAAGGCAAGCAGTACCTCAGCAACATGGGGACACGTAACAACTGTCTGCTGCCTAGCATGGCGACCTTTGTCACTTCCAGCAAACCAGATCTGCAGGTCACCATCAAAGAGGAGAGCTGTCCAATGCCTTACAACAGCTCCTGGCCGCCGTTTACAGACATCTCCCTTCCTGCCCCAGTGACCCCCACGCCCAGCAGCAGTCGGCCGGACCGGGAGGCCCGGGCCAGTGTCATCAAGAAGACATCTGATATCACCCAGGCCCGTGTCAAGAGCTGCTAA

>Horse_ENSECAG00000007508

ATGCCGTCGCCACCGGATCCGAAGGAGGGGAGCGGGTCCTTCTCGGTGCCCGGCGGCGGCGCCTCTCTCCAGGCAGCCTCCAGCGGTACCATGCCGGTGGAAAGGATGCGGATGCGGCCATGGCTGGAGGAACAGATAAACTCAAATACGATACCAGGGCTAAAGTGGCTTAACAAGGAGAAGAAGATTTTTCAGATCCCCTGGATGCATGCAGCTAGACATGGGTGGGATGTGGAGAAGGATGCCCCACTCTTTAGAAACTGGGCAATCCATACAGGAAAGCATCAACCGGGAGTAGATAAACCTGACCCAAAAACATGGAAGGCGAATTTTCGATGTGCCATGAACTCCTTGCCTGATATTGAAGAAGTCAAGGACAGAAGTATAAAGAAGGGAAACAATGCCTTCAGGGTGTACCGGATGCTGCCCTTATCTGAGCGACCTTCTAAGAAAGTCACACCAAGGATAGAACATCTGCTCACACTGTGGATGATCATTCTGCTTTACCTGGCTGGGGAAAATGAATCTCAGGTCCCCAAAGGCATTAGAAGAAGCATTTGGAGTTTT---------------------------------------------------------------------------------------------------------------------------------------GACCACATACAGATTAAAGCAACAGGAAAGAAACCAAAGACAGAAAAAGAAGACAGAGTTAAGCACATCAAGCAAGAACCAGTTGAGTCATCTTTGGGGCTTAGTAATGGAGTAAGTGATCTTTCTCCTGAGTATGCGGTCCTGACTTCAGCTATAAAAAATGAAGTGGATAGTACGGTGAACATCATAGTTGTAGGA---CAGTCCCATCTGGACAGCAACATCGAGGATCAAGAGATTGTCACTAATCCGCCAGACATTTGCCAAGTTGTGGAGGTGACCACAGAGAGTGATGAGCAGCCGGTCAGCATGAGTGAGCTCTACCCTCTGCAGATTTCCCCCGTGTCTTCCTACGCAGAAAGTGAAACTACCGATAGTGTGCCCAGTGATGAAGAGAGCACGGAGGGACGCCCACACTGGAGGAAGAGGAACATTGAAGGCAAACAGTACCTCAGCAACATGGGCACCAGAAGCACCTACCTGCTGCCCAGCATGGCGACCTTCGTGACTTCCAACAAGCCGGATCTCCAGGTCACCATCAAAGAGGAGAGCTGTCCGGTGCCTTTCAACAGCTCCTGGCCCCCTTTCCCAGACCTGCCCCTCACTCCCTCCGTGACCCCAGCGTCCAGCAGCAGTCGGCCCGACCGGGAGACTCGGGCCAGCGTCATCAAGAAAACGTCCGATATCACCCAGGCCCGCGTCAAGAGCTGTTAG

>GoldenSnubNoseMonkey_ENSRROG00000002645

------------------------------------------------------------------------------------------ATGCCGGTGGAAAGGATGCGCATGCGCCCGTGGCTGGAGGAGCAGATAAACTCCAACACGATCCCAGGGCTCAAGTGGCTTAACAAGGAAAAGAAGATTTTTCAGATCCCCTGGATGCATGCGGCTAGACATGGGTGGGATGTGGAAAAAGATGCACCACTCTTTAGAAACTGGGCAATCCATACAGGAAAGCATCAACCAGGAGTAGATAAACCTGATCCCAAAACATGGAAGGCGAATTTCAGATGTGCCATGAATTCCTTGCCTGATATTGAAGAAGTCAAGGATAAAAGCATAAAGAAAGGAAACAATGCCTTCAGGGTCTACCGAATGCTGCCCCTATCGGAGCGGCCTTCTAAGAAA---------------------------------------------------------------------------------------------------------------------------------------------------------------------------------------------------------------------------------------------------------------------------------GGAAAGAAACCAAAGACAGAAAAAGAAGACAAAGTGAAGCACATCAAGCAAGAACCAGTTGAGTCATCTCTGGGGCTTAGTAATGGAGTAAGTGATATTTCTCCTGAGTATGCGGTCCTGACTTCAGCTATAAAAAATGAAGTGGATAGTACGGTGAACATCATAGTTGTAGGA---CAGTCCCATCTGGACAGCAACATTGAGGATCAAGAGATTGTCACCAATCCGCCGGACATTTGCCAAGTCGTAGAGGTGACCACTGAGAGCGATGAGCAGCCGGTCAGCATGAGCGAGCTCTACCCTCTGCAGATCTCCCCCGTGTCTTCCTATGCAGAAAGCGAAACGACTGATAGTGTGCCCAGTGATGAAGAGAGCGCTGAGGGGCGGCCACACTGGCGGAAGAGGAATATTGAAGGCAAACAGTACCTCAGCAACATGGGGACTCGAGGCTCCTACCTGCTGCCCGGCATGGCGTCCTTCGTCACTTCCAACAAACCGGACCTCCAGGTCACCATCAAAGAGGAGAGCAATCCGGTGCCTTACAACAGCTCCTGGCCCCCTTTTCAAGACCTCCCCCTTTCTTCCTCCATGACCCCAGCATCCAGCAGCAGTCGGCCAGACCGGGAGACCCGGGCCAGTGTCATCAAGAAGACATCGGATATCACCCAGGCCCGCGTCAAGAGCTGTTAA

>Macaca_ENSMMUG00000015087

---------ATGAAGGAGGGAGTGTCAGAGCCAGTTCCTGGTGGCCGCAAATATCTCACTCAGGGCATGTGTTCCTGTTTCCCAGGCACCATGCCGGTGGAAAGGATGCGCATGCGCCCGTGGCTGGAGGAGCAGATAAACTCCAACACGATCCCAGGGCTCAAGTGGCTTAACAAGGAAAAGAAGATTTTTCAGATCCCCTGGATGCACGCGGCTAGACATGGGTGGGACGTGGAAAAAGACGCACCACTCTTTAGAAACTGGGCAATCCATACAGGAAAGCATCAACCAGGAGTAGATAAACCTGATCCCAAAACATGGAAGGCGAATTTCAGATGTGCCATGAATTCCTTGCCTGATATTGAAGAAGTCAAGGATAAAAGCATAAAGAAAGGAAACAATGCCTTCAGGGTCTACCGAATGCTGCCCCTATCGGAGCGGCCTTCTAAGAAA---------------------------------------------------------------------------------------------------------------------------------------------------------------------------------------------------------------------------------------------------------------------------------GGAAAGAAACCAAAGACAGAAAAAGAAGACAAAGTTAAGCACATCAAGCAAGAACCAGTTGAGTCATCTCTGGGGCTTAGTAATGGAGTAAGTGATCTTTCTCCTGAGTATGCGGTCCTGACTTCAACTATAAAAAATGAAGTGGATAGTACGGTGAACATCATAGTTGTAGGA---CAGTCCCATCTGGACAGCAACATTGAGGATCAGGAGATTGTCACCAATCCGCCGGACATTTGCCAAGTCGTAGAGGTGACCACGGAGAGCGATGAGCAGCCGGTCAGCATGAGCGAGCTCTACCCTCTGCAGATCTCCCCCGTGTCTTCCTATGCAGAAAGCGAAACGACTGATAGTGTGCCCAGCGATGAAGAGAGCGCTGAGGGGCGGCCACACTGGCGGAAGAGGAATATTGAAGGCAAACAGTACCTCAGCAACATGGGGACTCGAGGCTCCTACCTGCTGCCTGGCATGGCGTCCTTCGTCACTTCCAACAAACCGGACCTCCAGGTCACCATCAAAGAGGAGAGCAATCCGGTGCCTTACAACAGCTCCTGGCCCCCTTTTCAAGACCTCCCCCTTTCTTCCTCCATGACCCCAGCATCCAGCAGCAGTCGGCCAGACCGGGAGACCCGGGCCAGTGTCATCAAGAAAACATCGGATATCACCCAGGCCCGCGTCAAGAGCTGTTAA

>Gibbon_ENSNLEG00000010848

------------------------------------------------------------------------------------------ATGCCGGTGGAAAGGATGCGCATGCGCCCGTGGCTGGAGGAGCAGATAAACTCCAACACGATCCCAGGGCTCAAGTGGCTTAACAAGGAAAAGAAGATTTTTCAGATCCCCTGGATGCATGCGGCTAGACATGGGTGGGATGTGGAAAAAGATGCACCACTCTTTAGAAACTGGGCAATCCATACAGGAAAGCATCAACCAGGAGTAGATAAACCTGATCCCAAAACATGGAAGGCGAATTTCAGATGCGCCATGAATTCCTTGCCTGATATTGAAGAAGTCAAGGATAAAAGCATAAAGAAAGGAAACAACGCCTTCAGGGTCTACCGAATGCTGCCCCTGTCGGAGCGGCCTTCTAAGAAA---------------------------------------------------------------------------------------------------------------------------------------------------------------------------------------------------------------------------------------------------------------------------------GGAAAGAAACCAAAGACAGAAAAAGAAGACAAAGTTAAGCACATCAAGCAAGAACCAGTTGAGTCATCTCTGGGGCTTAGTAATGGAGTAAGTGATCTTTCTCCTGAGTATGCGGTCCTGACTTCAACTATAAAAAATGAAGTGGATAGTACGGTGAACATCATAGTTGTAGGA---CAGTCCCATCTGGACAGCAACATTGAGGATCAAGAGATTGTCACCAATCCGCCAGACATTTGCCAAGTTGTAGAGGTGACCACTGAGAGCGATGAGCAGCCGGTCAGCATGAGCGAGCTCTACCCTCTGCAGATCTCCCCTGTGTCTTCCTATGCAGAAAGCGAAACGACTGATAGTGTGCCCAGCGATGAAGAGAGTGCTGAGGGGCGGCCACACTGGCGGAAGAGGAATATTGAAGGCAAACAGTACCTCAGCAACATGGGGACTCGGGGCTCCTACCTGCTGCCTGGCATGGCGTCCTTCGTCACTTCCAACAAACCGGACCTCCAGGTCACCATCAAAGAGGAGAGCAATCCGGTGCCTTACAACAGCTCCTGGCCCCCTTTTCAAGACCTCCCCCTTTCTTCCTCCATGACCCCAGCACCCAGCAGCAGTCGGCCAGACCGGGAGACCCGGGCCAGCGTCATCAAGAAAACATCGGATATCACCCAGGCCCGCGTCAAGAGCTGTTAA

>Human_ENSG00000168310

------------------------------------------------------------------------------------------ATGCCGGTGGAAAGGATGCGCATGCGCCCGTGGCTGGAGGAGCAGATAAACTCCAACACGATCCCGGGGCTCAAGTGGCTTAACAAGGAAAAGAAGATTTTTCAGATCCCCTGGATGCATGCGGCTAGACATGGGTGGGATGTGGAAAAAGATGCACCACTCTTTAGAAACTGGGCAATCCATACAGGAAAGCATCAACCAGGAGTAGATAAACCTGATCCCAAAACATGGAAGGCGAATTTCAGATGCGCCATGAATTCCTTGCCTGATATTGAAGAAGTCAAGGATAAAAGCATAAAGAAAGGAAATAATGCCTTCAGGGTCTACCGAATGCTGCCCCTATCAGAACGGCCTTCTAAGAAA---------------------------------------------------------------------------------------------------------------------------------------------------------------------------------------------------------------------------------------------------------------------------------GGAAAGAAACCAAAGACAGAAAAAGAAGACAAAGTTAAGCACATCAAGCAAGAACCAGTTGAGTCATCTCTGGGGCTTAGTAATGGAGTAAGTGATCTTTCTCCTGAGTATGCGGTCCTGACTTCAACTATAAAAAATGAAGTGGATAGTACGGTGAACATCATAGTTGTAGGA---CAGTCCCATCTGGACAGCAACATTGAGAATCAAGAGATTGTCACCAATCCGCCAGACATTTGCCAAGTTGTAGAGGTGACCACTGAGAGCGACGAGCAGCCGGTCAGCATGAGCGAGCTCTACCCTCTGCAGATCTCCCCCGTGTCTTCCTATGCAGAAAGCGAAACGACTGATAGTGTGCCCAGCGATGAAGAGAGTGCCGAGGGGCGGCCACACTGGCGGAAGAGGAATATTGAAGGCAAACAGTACCTCAGCAACATGGGGACTCGAGGCTCCTACCTGCTGCCCGGCATGGCGTCCTTCGTCACTTCCAACAAACCGGACCTCCAGGTCACCATCAAAGAGGAGAGCAATCCGGTGCCTTACAACAGCTCCTGGCCCCCTTTTCAAGACCTCCCCCTTTCTTCCTCCATGACCCCAGCATCCAGCAGCAGTCGGCCAGACCGGGAGACCCGGGCCAGCGTCATCAAGAAAACATCGGATATCACCCAGGCCCGCGTCAAGAGCTGTTAA

>Gorilla_ENSGGOG00000016559

------------------------------------------------------------------------------------------ATGCCGGTGGAAAGGATGCGCATGCGCCCGTGGCTGGAGGAGCAGATAAACTCCAACACGATCCCGGGGCTCAAGTGGCTTAACAAGGAAAAGAAGATTTTTCAGATCCCCTGGATGCATGCGGCTAGACATGGGTGGGATGTGGAAAAAGATGCACCACTCTTTAGAAACTGGGCAATCCATACAGGAAAGCATCAACCAGGAGTAGATAAACCTGATCCCAAAACATGGAAGGCGAATTTCAGATGCGCCATGAATTCCTTGCCTGATATTGAAGAAGTCAAGGATAAAAGCATAAAGAAAGGAAACAATGCCTTCAGGGTCTACCGAATGCTGCCCCTATCAGAACGGCCTTCTAAGAAA---------------------------------------------------------------------------------------------------------------------------------------------------------------------------------------------------------------------------------------------------------------------------------GGAAAGAAACCAAAGACAGAAAAAGAAGACAAAGTTAAGCACATCAAGCAAGAACCAGTTGAGTCATCTCTGGGGCTTAGTAATGGAGTAAGTGATCTTTCTCCTGAGTATGCGGTCCTGACTTCAACTATAAAAAATGAAGTGGATAGTACGGTGAACATCATAGTTGTAGGA---CAGTCCCATCTGGACAGCAACATTGAGAATCAAGAGATTGTCACCAATCCGCCAGACATTTGCCAAGTTGTAGAGGTGACCACTGAGAGCGACGAGCAGCCAGTCAGCATGAGCGAGCTCTACCCTCTGCAGATCTCCCCCGTGTCTTCCTATGCAGAAAGCGAAACGACTGATAGTGTGCCCAGCGATGAAGAGAGTGCCGAGGGGCGGCCACACTGGCGGAAGAGGAATATTGAAGGCAAACAGTACCTCAGCAACATGGGGACTCGAGGCTCCTACCTGCTGCCCGGCATGGCGTCCTTCGTCACTTCCAACAAACCGGACCTCCAGGTCACCATCAAAGAGGAGAGCAATCCGGTGCCTTACAACAGCTCCTGGCCCCCTTTTCAAGACCTCCCCCTTTCTTCCTCCATGACCCCAGCATCCAGCAGCAGTCGGCCAGACCGGGAGACCCGGGCCAGCGTCATCAAGAAAACATCGGATATCACCCAGGCCCGCGTCAAGAGCTGTTAA

>Bonobo_ENSPPAG00000034580

------------------------------------------------------------------------------------------ATGCCGGTGGAAAGGATGCGCATGCGCCCGTGGCTGGAGGAGCAGATAAACTCCAACACGATCCCAGGGCTCAAGTGGCTTAACAAGGAAAAGAAGATTTTTCAGATCCCCTGGATGCATGCAGCTAGACATGGGTGGGATGTGGAAAAAGATGCACCACTCTTTAGAAACTGGGCAATCCATACAGGAAAGCATCAACCAGGAATAGATAAACCTGATCCCAAAACATGGAAGGCGAATTTCAGATGCGCCATGAATTCCTTGCCTGATATTGAAGAAGTCAAGGATAAAAGCATAAAGAAAGGAAACAATGCCTTCAGGGTCTACCGAATGCTGCCCCTATCTGAACGGCCTTCTAAGAAA---------------------------------------------------------------------------------------------------------------------------------------------------------------------------------------------------------------------------------------------------------------------------------GGAAAGAAACCAAAGACAGAAAAAGAAGACAAAGTTAAGCACATCAAGCAAGAACCAGTTGAGTCATCTCTGGGGCTTAGTAATGGAGTAAGTGATCTTTCTCCTGAGTATGCGGTCCTGACTTCAACTATAAAAAATGAAGTGGATAGTACGGTGAACATCATAGTTGTAGGA---CAGTCCCATCTGGACAGCAACATTGAGAATCAAGAGATTGTCACCAATCCGCCAGACATTTGCCAAGTTGTAGAGGTGACCACTGAGAGCGACGAGCAGCCGGTCAGCATGAGCGAGCTCTACCCTCTGCAGATCTCCCCCGTGTCTTCCTATGCAGAAAGCGAAACGACTGATAGTGTGCCCAGCGATGAAGAGAGTGCCGAGGGGCGGCCACACTGGCGGAAGAGGAATATTGAAGGCAAACAGTACCTCAGCAACATGGGGACTCGAGGCTCCTACCTGCTGCCCGGCATGGCGTCCTTCGTCACTTCCAACAAACCGGACCTCCAGGTCACCATCAAAGAGGAGAGCAATCCGGTGCCTTACAACAGCTCCTGGCCCCCTTTTCAAGACCTCCCCCTGTCTTCCTCCATGACCCCAGCATCCAGCAGCAGTCGGCCAGACCGGGAGACCCGGGCCAGCGTCATCAAGAAAACATCGGATATCACCCAGGCCCGCGTCAAGAGCTGTTAA

>Chimpanzee_ENSPTRG00000050798

------------------------------------------------------------------------------------------ATGCCGGTGGAAAGGATGCGCATGCGCCCGTGGCTGGAGGAGCAGATAAACTCCAACACGATCCCAGGGCTCAAGTGGCTTAACAAGGAAAAGAAGATTTTTCAGATCCCCTGGATGCATGCGGCTAGACATGGGTGGGATGTGGAAAAAGATGCACCACTCTTTAGAAACTGGGCAATCCATACAGGAAAGCATCAACCAGGAATAGATAAACCTGATCCCAAAACATGGAAGGCGAATTTCAGATGCGCCATGAATTCCTTGCCTGATATTGAAGAAGTCAAGGATAAAAGCATAAAGAAAGGAAACAATGCCTTCAGGGTCTACCGAATGCTGCCCCTATCTGAACGGCCTTCTAAGAAA---------------------------------------------------------------------------------------------------------------------------------------------------------------------------------------------------------------------------------------------------------------------------------GGAAAGAAACCAAAGACAGAAAAAGAAGACAAAGTTAAGCACATCAAGCAAGAACCAGTTGAGTCATCTCTGGGGCTTAGTAATGGAGTAAGTGATCTTTCTCCTGAGTATGCGGTCCTGACTTCAACTATAAAAAATGAAGTGGATAGTACGGTGAACATCATAGTTGTAGGA---CAGTCCCATCTGGACAGCAACATTGAGAATCAAGAGATTGTCACCAATCCGCCAGACATTTGCCAAGTTGTAGAGGTGACCACTGAGAGCGACGAGCAGCCGGTCAGCATGAGCGAGCTCTACCCTCTGCAGATCTCCCCCGTGTCTTCCTATGCAGAAAGCGAAACGACTGATAGTGTGCCCAGCGATGAAGAGAGTGCCGAGGGGCGGCCACACTGGCGGAAGAGGAATATTGAAGGCAAACAGTACCTCAGCAACATGGGGACTCGAGGCTCCTACCTGCTGCCCGGCATGGCGTCCTTCGTCACTTCCAACAAACCGGACCTCCAGGTCACCATCAAAGAGGAGAGCAATCCGGTGCCTTACAACAGCTCCTGGCCCCCTTTTCAAGACCTCCCCCTGTCTTCCTCCATGACCCCAGCATCCAGCAGCAGTCGGCCAGACCGGGAGACCCGGGCCAGCGTCATCAAGAAAACATCGGATATCACCCAGGCCCGCGTCAAGAGCTGTTAA

>MasNightMonkey_ENSANAG00000029650

------------------------------------------------------------------------------------------ATGCCGGTGGAAAGGATGCGCATGCGCCCGTGGCTGGAGGAGCAGATAAACTCCAACACGATCCCAGGGCTCAAGTGGCTTAACAAGGAAAAGAAGATTTTTCAGATCCCCTGGATGCACGCGGCTAGACATGGGTGGGACGTGGAGAAAGATGCACCACTCTTTAGAAACTGGGCAATCCACACAGGAAAGCATCAACCAGGAGTAGATAAACCTGATCCCAAAACATGGAAGGCGAATTTCAGATGTGCCATGAACTCCTTGCCTGATATTGAAGAAGTTAAGGATAAAAGCATAAAGAAAGGAAACAATGCGTTCAGAGTCTACCGAATGCTGCCCCTCTCGGAACGGCCTTCTAAGAAA---------------------------------------------------------------------------------------------------------------------------------------------------------------------------------------------------------------------------------------------------------------------------------GGAAAGAAACCAAAGACAGAAAAAGAAGACAGGGTTAAGCACATCAAGCAAGAACCAGTTGAGTCATCTCTGGGGCTTAGTAATGGAGTAAGTGATCTTTCTCCTGAGTATGCGGTCCTGACTTCAGCTATAAAAAATGAAGTGGATAGTACGGTGAACATCATAGTCGTAGGA---CAGTCCCATCTGGACAGCAACATCGAGGATCAAGAGATTGTCACCAATCCGCCGGACATTTGCCAAGTTGTAGAGGTGACCACTGAGAGCGACGAGCAGCCGGTCAGCATGAGCGAGCACTACCCCCTGCAGATCTCCCCCGTGTCTTCCTACGCAGAAAGTGAAACGACTGACAGTGTGCCCAGTGATGAAGAGAGTGCCGAGGGGCGGCCACACTGGCGGAAGAGGAATATTGAAGGCAAACAGTACCTCAGCAACATGGGGATTCGAGGCCCCTACCAGCTGCCGGGGATGGCGAGCTTCGTCACTTCCAGCAAACCGGACCTCCAGGTCACCATCAAAGATGAGAGCCGTCCGGTGCCTTACAACAGCTCCTGGCCCCCTTTTCAAGACCTCCCCCTTTCTTCCTCCGTGACTCAAGCATCCAGCAGCAGTCGGCCAGACCGGGAGACCCGGGCCAGCGTCATCAAGAAAACATCGGATATCACCCAGGCCCGCGTCAAGAGCTGTTAA

>Marmoset_ENSCJAG00000042693

------------------------------------------------------------------------------------------ATGCCGGTGGAAAGGATGCGCATGCGCCCGTGGCTGGAGGAGCAGATAAACTCCAACACGATCCCAGGGCTCAAGTGGCTTAACAAGGAGAAGAAGATTTTTCAGATCCCCTGGATGCATGCTGCTAGACATGGGTGGGACGTGGAGAAAGATGCACCACTCTTTAGAAACTGGGCAATCCACACAGGAAAGCATCAACCAGGAGTAGATAAACCTGATCCCAAAACATGGAAGGCGAATTTCCGATGTGCCATGAACTCCTTGCCTGATATTGAAGAAGTGAAGGATAAAAGCATAAAAAAAGGCAACAACGCGTTCAGAGTCTACCGAATGCTGCCCCTGTCGGAGCGGCCTTCTAAGAAA---------------------------------------------------------------------------------------------------------------------------------------------------------------------------------------------------------------------------------------------------------------------------------GGAAAGAAACCAAAGACAGAGAAAGAAGACAGAGTTAAGCACATCAAGCAAGAACCAGTTGAGTCATCTCTGGGGCTTAGTAATGGAGTAAGTGATCTTTCTCCCGAGTATGCGGTCCTGGCTTCAGCTATAAAAAATGAAGTGGATAGTACGGTGAACATCATAGTCGTAGGA---CAATCCCATCTGGACGGCAACATCGAGGATCAAGAGATTGTCACCAACCCGCCGGACATTTGCCAAGTTGTAGAGGTGACCACTGAGAGCGACGAGCAGCCGGCCAGCATGAGCGAGCTGTACCCTCTGCAGATCTCACCCGTGTCTTCCTACGCGGAAAGTGAAACGACTGACAGTGTGCCCAGCGATGAAGAGAGCACCGAGGGGCGGCCACACTGGCGGAAGAGAAATATTGAAGGCAAACAGTACCTCAGCAACATGGGGATGCGAGGCCCCTACCAGCTGCCAGGGATGGCAAGCTTCGTCACTTCCAGCAAACCGGACCTCCAGGTCACCATCAAAGATGAGAGTCGTCCGGTGCCTTACAACAGCTCCTGGCCCCCTTTTCAAGACCTCCCCCTTTCTTCCTCCATGACTCAAGCATCCAGCAGCAGTCGGCCAGACCGGGAGACCCGGGCCAGCGTCATCAAGAAAACATCGGATATCACCCAGGCCCGCGTCAAGAGCTGTTAA

>MouseLemur_ENSMICG00000015506

------------------------------------------------------------------------------------------ATGCCGGTGGAGAGGATGCGCATGCGCCCGTGGCTGGAGGAGCAGATCAACTCCAACACGATACCGGGGCTGAAGTGGCTGAACAAGGAAAAGAAGATTTTTCAGATCCCCTGGATGCATGCAGCTAGACATGGGTGGGATGTGGAAAAAGACGCACCACTCTTTAGAAACTGGGCAATTCATACAGGAAAGCATCAACCAGGAGTAGATAAACCTGATCCAAAAACATGGAAGGCGAATTTTCGATGTGCCATGAACTCCTTGCCTGATATTGAAGAAGTTAAGGATAAAAGTATAAAGAAAGGAAACAATGCCTTCAGAGTATACCGGATGTTGCCCTTATCTGAGCGACCTTCTAAAAAA---------------------------------------------------------------------------------------------------------------------------------------------------------------------------------------------------------------------------------------------------------------------------------GGAAAGAAACCAAAGACAGAAAAAGAGGACAAAGTTAAGCACATCAAGCAAGAACCAGTTGAGTCATCTCTGGGGCTTAGTAATGGAGTAAGTGATCTTTCTCCCGAGTATGCGGTCCTGACTTCAGCTATAAAAAATGAAGTGGACAGTACGGTGAACATCATAGTTGTAGGACCGTCATCCCATCTGGACAGCAACATTGAGGATCAAGAGATTGTCACTAATCCACCGGACATTTGCCAAGTTGTAGAGGTGACCACTGAGAGCGACGAGCAGCCGGTCAGCATGAGTGAGCTCTACCCTCTGCAGATTTCCCCCGTGTCTTCCTACGCAGAAAGCGAAACGACCGACAGCGTGCCCAGCGACGAAGAGAACGCCGAGGGGCGGCCGCACTGGTGGAAGAGGAACATCGAAGGCAAACAGTACCTCAGCAACACGGGCTCGCGGAGCACGTACCTGCTGCCCAGCATGGCGACCTTCGTCACCTCCAACAAGCCCGACCTCCAGGTCACCATCAAAGAGGAGAGCTGTCCGCTGCCTTACAACAGCTCCTGGCCCCCCTTCGCGGACCTCCCCCTGTCTCCGTCCGTGACCGCCGCCCCCGGCAGCAGTCGGCCGGACCGCGAGACCCGGGCCAGCGTCATCAAGAAGACGTCAGACATCACCCAGGCCCGCGTCAAGAGCTGCTAA

>Dog_ENSCAFG00845015393

---------------------------------ATGCGACCGGGTCCCCCCGCCAGGACGCGCCGGCCCCGGCCCCAGGTCCCGGGTACCATGCCGGTGGAAAGGATGCGGATGCGCCCGTGGCTGGAGGAACAGATAAACTCAAATACGATACCAGGGCTAAAGTGGCTTAACAAGGAAAAGAAGATTTTCCAGATCCCTTGGATGCATGCAGCTAGACATGGGTGGGATGTGGAAAAAGATGCACCACTCTTTAGAAACTGGGCGATCCATACAGGAAAGCATCAACCGGGAGTAGATAAACCTGATCCAAAAACATGGAAGGCGAATTTTCGATGTGCCATGAACTCCTTGCCTGACATTGAGGAAGTCAAGGATAAAAGTATAAAGAAGGGAAACAATGCATTCAGAGTGTACCGGATGTTGCCCTTATCTGAGCGACCTTCTAAGAAA---------------------------------------------------------------------------------------------------------------------------------------------------------------------------------------------------------------------------------------------------------------------------------GGAAAGAAACCAAAGACCGAGAAAGAAGACAGAGTTAAGCACATCAAGCAAGAACCAGTTGAGTCATCTTTGGGGCTTAGTAATGGAGTAAGTGATCTTTCTCCTGAGTATGCCGTCCTGACTTCAGCTATAAAAAATGAAGTGGATAGTACGGTGAACATCATAGTTGTAGGA---CAGTCACATCTGGACAGCAACATCGACGATCAAGAGATTGTCACTAATCCTCCGGACATTTGCCAAGTTGTAGAGGTGACCACCGAGAGTGACGAGCAGCCGGTCAGCATGAGTGAGCTCTACCCTCTGCAGATTTCCCCCGTGTCTTCCTACGCAGAAAGCGAAACTACCGATAGTGTGCCCAGTGACGAAGAGAGCGTCGAGGGACGACCCCACTGGAGGAAGAGGAACATTGAAGGCAAACAGTACCTCAGCAACATGGGGACTCGAAGCACCTACCTGCTGCCCAGCATGGCGACCTTCGTTACTTCCAACAAACCTGACCTCCAGGTCACCATCAAAGAGGAGAGCTGTCCGGTGCCTTACAACAGCTCATGGCCCACTTTACCAGACCTCCCCCTCCCTCCCTCCGTGACCCCAGCGCCCAGCAGCAGTCGGCCAGACCGGGAGACTCGGGCCAGCGTCATCAAGAAAACATCAGATATCACCCAGGCCCGAGTCAAGAGCTGTTAA

# IRF3

>Elephant_ENSLAFG00000028074

---------------------------------------------------------------------------------------------------------------------------------------ATGGCGACCCAAAAGCCGCGGATTCTGCCCTGGCTCATTTCGCTGCTGGACAGGGGGCAGCTGGAGGGCGTGGCCTGGGTGGACGCGAACCGCACGCGCTTCCGCATCCCTTGGAAGCACCGCCTGCGGCAGGATACGCAGCAGGAAGACTTCCGCATCTTC---------------CAG---------------------------------------------------------------------------------------GCGTGGAGCTGGCCCGAGTGCGTGCTTGTGGGTGGAGAATTCTGGGGGCAGGTCTTGGAACTTTGGAGACGAGGCTCAGAGTTCCACAGCTGGTTTAATTACCATCAAGTTACATTCTATTCATTCTCCCATGCCCAGGTCTTGCTAGATCCATGGGTTGGAATTAGCTCCTCCTCCCCC---AAGGCCCAAGCACTTACCCATTTGTGCCACCACGGGGAACAGCTGTTGAAATGCCCGACTCCTTTTCTTCTCCAGGAAGACATTCTGGAGGAGTTACTGGGTGACATGGTCTTGGCCTCAGTCCCAGATGGGGGGCCCTCAGGACTGGCTGTGGCCTCTGAGCAGCCCCCTCAGCTCTTGCTGAGCCCCAACATTGACGTTCTTCCTCCCTGCCCAAACCTGGCATCCTCGGAAAACCCACTGAGGCAGCTGCTGGTCCCCGAGGAAGAGTGGGAGTTTGAGGTGACCGCCTTCTACCGGGGCCGCCAAGTCTTCCAGCAGGCTGTCTTCTGCCCAGGTGGCCTGCGGCTGGTGGGATCAGAAGCAGCAGACAATACACTGCCTGGGCAGCTGATGATGCTGCCTGACCCTGGGGTGGCCCTGACAGACAAGGGGGCGACAAGCTTCGTGAGGCGTGTGCTGAGCTCCCTAGGCGGGGGGCTTGCTCTGTGGAGGGCAGGGCAACAGCTCTGGGCCCGAAGGCTGGGGCACTGCCGTACATACTGGGCTGCGGGTGAGGAGCTGCTCCCTGATGGCACTCACGGGCCAGACGGAGAGGTCCCCAAAGACAGAGAAGGAGTCGTGTTCAACCTGGGACCTTTTGTGGCAGATCTGATTGCC------------TTCATCGAAGGAAGCAAACGCTCACCCCGC------TACACCCTCTGGTTCTGCATTGGGGAGTTATGGCCCCAGGACCAGCCGTGGACCAAGAAGCTGGTGATGGTCAAGGTA---------------GTTCCCACATGCCTCCGGGTCCTGCTAGACATGGCCCGGGTGGAG------------------------------------GGTGCCTCCTCACTGGAGACTACCATGGACCTG---CACATTTCTAACAGCCACCCGCTCTCCCTC---------ACCTCAGACCAGTACAAGGCCTATCTGCAGGACCTGGCCGAGGACATGGATTTCTAA---------------------------------------

>Megabat_ENSPVAG00000010968

---------------------------------------------------------------------------------------------------------------------------------------ATGGCTACCCCAAAGCCGAGGATCCTGCCCTGGCTAGTGTCGCAACTGGACAGTGGGCAGCTGGAGGGCGTGGCATGGCTGGACGAGAGCCACACGCGCTTTCGCATCCCTTGGAAGCACGGCTTGCGGCAGGATGCCCAGCAGGAGGACTTCGGCATCTTC---------------CAG---------------------------------------------------------------------------------------GCCTGGGCCCAGGCCAGCGGTGCTTACACTCCTGGGAAGGATAAGCCTGAC---CTGCCCACCTGGAAGAGG------AATTTCAGGTCTGCCTTGAACCGGAAGGAAGTGTTGCGTTTAGCGGAGGACCGGAGCAAGGATCCCCGTGACCCGCACAAGATCTACGAGTTTGTGTCCTCA---GGAGTTGGGGACTGTCCTGAGCTGGACACTTCTTCAGAAACCGATGGCAGATGCAGTACCTCC---GATACCCAGGAAGACATTCTA---GAGTTACTGAGTGACATGGCCTTGGTCCCAGGTCCAAATGAGGGGCCCTTGAGCCTGGCCATGGCCACTGAACAGCCCCCCCAGCTCTTGCTGAGCCCCAGCTTAGACATCCCTGCTCCCTGCCCAAACTCAGAACCCCCTGAAAACCCACTGAGGCAGCTCCTGGTGCCTGAGGAAGAGTGGGAGTTCGAGGTGACTGCCTTCTACCGGGGCCGCCAAGTCTTCCAGCAGACCATCTTCTGCCCAAAGGGCCTGCGGCTGGTGGGGTCAGAAGCAGGGGACAAGGCACTGCCTGGGCAGCCGATCACACTGCCAGACCCTGGAGTGTCCCTTACAGACAAGGCCGTGACAGGCTACGTGAAGCGTGTGCTGAGCTCCCTCGGGGGGGGCCTAGCTCTGTGGAGGGCAGGGCAGCAGCTCTGGGCCCAGAGGCTGGGGCACTGCCACACCTACTGGGCCATGGGCGAGGAGCTCCTCCCCAGCAGTGGTCACGGGCCTGATGGCGAGGTCCCCAAGGACAGGGAAGGAGGTGTGTTCGACCTGAAGCCCTTCGTGGCAGGCTCCTGGGCCCCCAGATCTGATCGCCTTCATCGAAGGAAGTGGACGCTCGCCACGCTACACCCTCTGGTTCTGCATGGGGGAGTCATGGCCCCA------GGATCAGCCATGGATCAAGAAGCTTGTGATGGTCAA---------------------GGTCGTTCCCACGTGCCTCCGGGCCCTGCTGGACATGGCGCGGGCAGGGGGTGCCTCCTCCCTGGAGAACACGGTGGACCTGCACATTTCTCACAGCCACCCGCTCTCCCTCACGGCAGACCAGTACAAGGCCTACCTCCAGGACCTGGTCGAGGACATGGATTTCTAGGTCACTGGGGACTTCTCCCTCCCCATGTCAACCAGTAA---------------------------------------

>Gorilla_ENSGGOG00000000499

---------------------------------------------------------------------------------------------------------------------------------------ATGGGAACCCCAAAGCCACGGATCCTGCCCTGGCTGGTGTCGCAGCTGGACCTGGGGCAACTGGAGGGCGTGGCCTGGGTGAACAAGAGCCGCACGCGCTTCCGCATCCCTTGGAAGCACGGCCTACGGCAGGATGCACAGCAGGAGGATTTCGGAATCTTC---------------CAG---------------------------------------------------------------------------------------GCCTGGGCCGAGGCCACTGGTGCATATGTTCCCGGGAGGGATAAGCCAGAC---CTGCCAACCTGGAAGAGG------AATTTCCGTTCTGCCCTCAACCGCAAAGAAGGGTTGCGTTTAGCAGAGGACCGGAGCAAGGACCCTCACGACCCACATAAAATCTACGAGTTTGTGAACTCA---GGAGTTGGGCACTTTTCCCAGCCAGACACCTCTCTGGACACCAATGGTGGAGGCAGTACTTCT---GACACCCAGGAAGACATTCTGGATGAGTTACTGGGTAACATGGTGTTGGCCCCACTCCCAGATCCGGGACCCCCAAGCCTGGCTGTAGCCCCTGAGCCCTGCCCTCAGCCCCTGCGGAGCCCCAGCTTGGACAGTCCCACTCCCTTCCCAAACCTGGGGCCCTCTGAGAACCCACTGAAGCGGCTGTTGGTGCCGGGGGAAGAGTGGGAGTTCGAGGTGACAGCCTTCTACCGGGGCCGCCAAGTCTTCCAGCAGACCATCTCCTGCCTGGAGGGCCTGCGGCTGGTGGGGTCCGAAGTGGGAGACAGGACGCTGCCTGGATGGCCAGTCACACTGCCAGACCCTGGCATGTCCCTGACAGACAGGGGAGTGATGAGCTACGTGAGGCATGTGCTGAGCTGCCTGGGTGGGGGACTGGCTCTCTGGCGGGCCGGGCAGTGGCTCTGGGCCCAGCGGCTGGGGCACTGCCACACATACTGGGCAGTGAGCGAGGAGCTGCTCCCCAACAGCGGGCATGGGCCTGACGGCGAGGTCCCCAAGGACAAGGAAGGAGGCGTGTTCAACCTGGGGCCCTTCATTGTAGGCTCCTGGGCCCCCAGATCTGATTACCTTCACGGAAGGAAGCGGACGCTCACCACGCTATGCCCTCTGGTTCTGTGTGGGGGAGTCATGGCCCCA------GGACCAGCCGTGGACCAAGAGGCTCGTGATGGTCAA---------------------GGTTGTGCCCACGTGCCTCAGGGCCTTGGTAGAAATGGCCCGGGTAGGGGGTGCCTCCTCCCTGGAGAACACTGTGGACCTGCACATTTCCAACAGCCACCCACTCTCCCTCACCTCCGACCAGTACAAGGCCTACCTGCAGGACTTGGTGGACGGAATGGATTTCCAGGGCCCTGGGGAGACCTGAGCCCTCGCTCCTCATGGTGTGCCTCCAACCCCCCTGTTCCCCACCATCTCAACCAATAA

>Human_ENSG00000126456

---------------------------------------------------------------------------------------------------------------------------------------ATGGGAACCCCAAAGCCACGGATCCTGCCCTGGCTGGTGTCGCAGCTGGACCTGGGGCAACTGGAGGGCGTGGCCTGGGTGAACAAGAGCCGCACGCGCTTCCGCATCCCTTGGAAGCACGGCCTACGGCAGGATGCACAGCAGGAGGATTTCGGAATCTTC---------------CAG---------------------------------------------------------------------------------------GCCTGGGCCGAGGCCACTGGTGCATATGTTCCCGGGAGGGATAAGCCAGAC---CTGCCAACCTGGAAGAGG------AATTTCCGCTCTGCCCTCAACCGCAAAGAAGGGTTGCGTTTAGCAGAGGACCGGAGCAAGGACCCTCACGACCCACATAAAATCTACGAGTTTGTGAACTCA---GGAGTTGGGGACTTTTCCCAGCCAGACACCTCTCCGGACACCAATGGTGGAGGCAGTACTTCT---GATACCCAGGAAGACATTCTGGATGAGTTACTGGGTAACATGGTGTTGGCCCCACTCCCAGATCCGGGACCCCCAAGCCTGGCTGTAGCCCCTGAGCCCTGCCCTCAGCCCCTGCGGAGCCCCAGCTTGGACAATCCCACTCCCTTCCCAAACCTGGGGCCCTCTGAGAACCCACTGAAGCGGCTGTTGGTGCCGGGGGAAGAGTGGGAGTTCGAGGTGACAGCCTTCTACCGGGGCCGCCAAGTCTTCCAGCAGACCATCTCCTGCCCGGAGGGCCTGCGGCTGGTGGGGTCCGAAGTGGGAGACAGGACGCTGCCTGGATGGCCAGTCACACTGCCAGACCCTGGCATGTCCCTGACAGACAGGGGAGTGATGAGCTACGTGAGGCATGTGCTGAGCTGCCTGGGTGGGGGACTGGCTCTCTGGCGGGCCGGGCAGTGGCTCTGGGCCCAGCGGCTGGGGCACTGCCACACATACTGGGCAGTGAGCGAGGAGCTGCTCCCCAACAGCGGGCATGGGCCTGATGGCGAGGTCCCCAAGGACAAGGAAGGAGGCGTGTTTGACCTGGGGCCCTTCATTGTAGGCTCCTGGGCCCCCAGATCTGATTACCTTCACGGAAGGAAGCGGACGCTCACCACGCTATGCCCTCTGGTTCTGTGTGGGGGAGTCATGGCCCCA------GGACCAGCCGTGGACCAAGAGGCTCGTGATGGTCAA---------------------GGTTGTGCCCACGTGCCTCAGGGCCTTGGTAGAAATGGCCCGGGTAGGGGGTGCCTCCTCCCTGGAGAATACTGTGGACCTGCACATTTCCAACAGCCACCCACTCTCCCTCACCTCCGACCAGTACAAGGCCTACCTGCAGGACTTGGTGGAGGGCATGGATTTCCAGGGCCCTGGGGAGAGCTGAGCCCTCGCTCCTCATGGTGTGCCTCCAACCCCCCTGTTCCCCACCACCTCAACCAATAA

>Chimpanzee_ENSPTRG00000011309

---------------------------------------------------------------------------------------------------------------------------------------ATGGGAACCCCAAAGCCACGGATCCTGCCCTGGCTGGTGTCGCAGCTGGACCTGGGGCAACTGGAGGGCGTGGCCTGGGTGAACAAGAGCCGCACGCGCTTCCGCATCCCTTGGAAGCACGGCCTACGGCAGGATGCACAGCAGGAGGATTTCGGAATCTTC---------------CAG---------------------------------------------------------------------------------------GCCTGGGCCGAGGCCACTGGCGCATATGTTCCCGGGAGGGATAAGCCAGAC---CTGCCAACCTGGAAGAGG------AATTTCCGCTCTGCCCTCAACCGCAAAGAAGGGCTGCGCTTAGCAGAGGACCGGAGCAAGGACCCTCACGACCCACATAAAATCTACGAGTTTGTGAACTCA---GGAGTTGGGGACTTTTCCCAGCCAGACACCTCTCCGGACACCAATGGTGGAGGCAGTACTTCT---GATACCCAGGAAGACATTCTGGATGAGTTACTGGGTAACATGGTGTTGGCCCCACTCCCAGATCCGGGACCCCCAAGCCTGGCTGTAGCCCCTGAGCCCTGCCCTCAGCCCCTGCGGAGCCCCAGCTTGGACAATCCCACTCCCTTCCCAAACCTGGGGCCCTCTGAGAACCCACTGAAGCGGCTGTTGGTACCGGGGGAAGAGTGGGAGTTCGAGGTGACAGCCTTCTACCGGGGCCGCCAAGTCTTCCAGCAGACCATCTCCTGCCCGGAGGGCCTGCGGCTGGTGGGGTCCGAAGTGGGAGACAGGACGCTGCCTGGATGGCCAGTCACACTGCCAGACCCTGGCATGTCCCTGACAGACAGGGGAGTGATGAGCTACGTGAGGCATGTGCTGAGCTGCCTGGGTGGGGGACTGGCTCTCTGGCGGGCCGGGCAGTGGCTCTGGGCCCAGCGGCTGGGGCACTGCCACACATACTGGGCAGTGAGCGAGGAGCTGCTCCCCAACAGCGGGCATGGGCCTGATGGCGAGGTCCCCAAGGACAAGGAAGGAGGCGTGTTTGACCTGGGGCCCTTCATTGTAGGCTCCTGGGCCCCCAGATCTGATTACCTTCACGGAAGGAAGCGGACGCTCACCACGCTATGCCCTCTGGTTCTGTGTGGGGGAATCATGGCCCCA------GGACCAGCCGTGGACCAAGAGGCTCGTGATGGTCAA---------------------GGTTGTGCCCACGTGCCTCAGGGCCTTGGTAGAAATGGCCCGGGTAGGGGGTGCCTCCTCCCTGGAGAACACTGTGGACCTGCACATTTCCAACAGCCACCCACTCTCCCTCACCTCCGACCAGTACAAGGCCTACCTGCAGGACTTGGTGGAGAGCATGGATTTCCAGGGCCCTGGGGAGACCTGAGCCCTCGTTTCTCATGGTGTGCCTCCAACCCCCCTGTTCCCCACCACCTCAACCAATAA

>Bonobo_ENSPPAG00000035759

---------------------------------------------------------------------------------------------------------------------------------------ATGGGAACCCCAAAGCCACGGATCCTGCCCTGGCTGGTGTCGCAGCTGGACCTGGGGCAACTGGAGGGCGTGGCCTGGGTGAACAAGAGCCGCACGCGCTTCCGCATCCCTTGGAAGCACGGCCTACGGCAGGATGCACAGCAGGAGGATTTCGGAATCTTC---------------CAG---------------------------------------------------------------------------------------GCCTGGGCCGAGGCCACTGGCGCATATGTTCCCGGGAGGGATAAGCCAGAC---CTGCCAACCTGGAAGAGG------AATTTCCGCTCTGCCCTCAACCGCAAAGAAGGGCTGCGCTTAGCAGAGGACCGGAGCAAGGACCCTCACGACCCACATAAAATCTACGAGTTTGTGAACTCA---GGAGTTGGGGACTTTTCCCAGCCAGACACCTCTCCGGACACCAATGGTGGAGGCAGTACTTCT---GATACCCAGGAAGACATTCTGGATGAGTTACTGGGTAACATGGTGTTGGCCCCACTCCCAGATCCGGGACCCCCAAGCCTGGCTGTAGCCCCTGAGCCCTGCCCTCAGCCCCTGCGGAGCCCCAGCTTGGACAATCCCACTCCCTTCCCAAACCTGGGGCCCTCTGAGAACCCACTGAAGCGGCTGTTGGTACCGGGGGAAGAGTGGGAGTTCGAGGTGACAGCCTTCTACCGGGGCCGCCAAGTCTTCCAGCAGACCATCTCCTGCCCAGAGGGCCTGCGGCTGGTGGGGTCCGAAGTGGGAGACAGGACGCTGCCTGGATGGCCAGTCACACTGCCAGACCCTGGCATGTCCCTGACAGACAGGGGAGTGATGAGCTACGTGAGGCATGTGCTGAGCTGCCTGGGTGGGGGACTGGCTCTCTGGCGGGCCGGGCAGTGGCTCTGGGCCCAGCGGCTGGGGCACTGCCACACATACTGGGCAGTGAGCGAGGAGCTGCTCCCCAACAGCGGGCATGGGCCTGATGGCGAGGTCCCCAAGGACAAGGAAGGAGGCGTGTTTGACCTGGGGCCCTTCATTGTAGGCTCCTGGGCCCCCAGATCTGATTACCTTCACGGAAGGAAGCGGACGCTCACCACGCTATGCCCTCTGGTTCTGTGTGGGGGAATCATGGCCCCA------GGACCAGCCGTGGACCAAGAGGCTCGTGATGGTCAA---------------------GGTTGTGCCCACGTGCCTCAGGGCCTTGGTAGAAATGGCCCGGGTAGGGGGTGCCTCCTCCCTGGAGAACACTGTGGACCTGCACATTTCCAACAGCCACCCACTCTCCCTCACCTCCGACCAGTACAAGGCCTACCTGCAGGACTTGGTGGAGGGCATGGATTTCCAGGGCCCTGGGGAGACCTGAGCCCTCGTTCCTCATGGTGTGCCTGCAACCCCCCTGTTCCCCACCACCTCAACCAATAA

>Mouse_ENSMUSG00000003184

---------------------------------------------------------------------------------------------------------------------------------------ATGGAAACCCCGAAACCGCGGATTTTGCCCTGGCTGGTGTCACAGCTGGACCTGGGGCAGCTGGAAGGCGTGGCCTGGCTGGACGAGAGCCGAACGAGGTTCAGGATCCCGTGGAAGCATGGCCTACGGCAGGACGCACAGATGGCTGACTTTGGCATCTTC---------------CAG---------------------------------------------------------------------------------------GCCTGGGCAGAAGCCAGTGGTGCCTACACCCCGGGGAAGGATAAGCCGGAC---GTGTCAACCTGGAAGAGG------AATTTCCGGTCAGCCCTGAACCGGAAAGAAGTGTTGCGGTTAGCTGCTGACAATAGCAAGGACCCTTATGACCCTCATAAAGTGTATGAGTTTGTGACTCCA---GGGGCGCGGGACTTCGTACATCTGGGTGCCTCTCCTGACACCAATGGCAAAAGCAGCCTGCCT---CACTCCCAGGAAAAC---CTACCGAAGTTATTTGATGGCCTGATCTTGGGGCCCCTCAAAGATGAGGGGTCCTCAGATCTGGCTATTGTTTCTGATCCT---TCTCAACAACTGCCAAGCCCCAATGTGAACAAC---------TTCCTAAACCCTGCACCCCAAGAAAATCCACTGAAGCAGCTGCTAGCT---GAGGAACAATGGGAGTTCGAGGTGACCGCCTTCTACCGAGGCCGCCAGGTCTTCCAGCAGACACTCTTTTGCCCGGGGGGCCTGCGGCTGGTGGGCAGC---ACAGCTGACATGACACTGCCCTGGCAGCCAGTCACCCTGCCCGATCCTGAGGGGTTTCTGACGGACAAGCTTGTGAAGGAGTACGTGGGGCAGGTGCTCAAAGGGCTGGGCAATGGGCTGGCACTGTGGCAGGCTGGGCAGTGCCTCTGGGCCCAGCGCCTAGGCCACTCCCACGCCTTCTGGGCTCTGGGGGAGGAGCTGCTTCCAGACAGTGGGCGAGGGCCTGATGGAGAGGTCCACAAGGACAAGGACGGAGCCGTGTTCGACCTCAGGCCCTTCGTGGCAGATCTGATTGCC------------TTCATGGAAGGAAGTGGACACTCCCCACGC------TACACTCTGTGGTTCTGCATGGGGGAAATGTGGCCCCAGGACCAGCCATGGGTCAAGAGGCTTGTGATGGTCAAGGTT---------------GTTCCTACATGTCTTAAGGAGCTGTTAGAGATGGCCCGGGAAGGG------------------------------------GGAGCCTCTTCACTGAAA---ACCGTGGACTTG---CACATCTCCAACAGCCAGCCTATCTCCCTT---------ACCTCTGACCAGTACAAGGCCTACCTCCAGGACTTGGTGGAGGACATGGACTTCCAGGCCACTGGAAATATCTGA---------------------

>Rat_ENSRNOG00000043388

ATGCTCCATACTAAGACCCTGTCTCAAAACATCCATAAAAACAATGTAGAAAAGACAAAGCAGAAGAAGCGACAGCAGCTGGGAGAAGACTCTGGAAATGAGCTCAGGAACTGCACTGTATGCGCAGGCTGGATCATGGGAACCCCGAAACCGCTGATTTTGCCTTGGCTGGTGTCTCAGCTGGACCTGGGACAGCTGAAAGGTGTGGCCTGGCTGGACGAGAGCCGTACAAAGTTCAGGATCCCATGGAAGCATGGCTTACGACAGGACGCACAGATGGCTGACTTTGGCATCTTC---------------CAG---------------------------------------------------------------------------------------GCCTGGGCGGAAGCCAGTGGTGCCTACACCCCAGGGAAGGATAAGCCAGAC---CTGTCAACCTGGAAGAGG------AATTTCCGGTCAGCCCTGAACCGGAAAGAAGTGTTGCGATTAGCTGAGGACCGGAGCAAGGACCCTTTTGACCCTCATAAAGTGTATGAGTTTGTGACTCCAGGAGGAGCAAGGGACTTTGTACATCTGGACACCTCCCCTGACACCAATGGCAAAAGCAGTCTGTCT---GATCACCAGGAAGACCTCTTG---GAATTACTCGATCACATGGCCTTGGGACCCCTCCCAGATGAGGGGTCCTCAGACCTGCCTATTGCTTCTGATCCT---TCTCAACCACCACTAAGCCCCATTGTAAACAAC---------TTCCCAAACCCAGCACCCCAGGAAAACCCACTAAGGCAGCTGCTAGCT---GAGGAACAGTGGGAGTTCGAGGTGACTGCCTTCTACCGAGGCCGCCAGGTCTTCCAGCAGACACTCTTTTGCCCCGGGGGCCTGCGGCTGGTGGGCAGCACGTCTGACAACGGGACACTGCCCTGGCAGCCAGTCACCCTGCCAGACCCTGAGGAGTTTCTGACAGACAGGCTTGTGAGGGAGTATGTGAGGCAGGTACTCAAGGGGCTGGGCAAGGGGCTGGTGCTGTGGCGGGCAGGGCAGTGCCTCTGGGCCCAGCGCCTAGGCCACTCGCATTCCTTCTGGGCCCTGGGTGAGGAGCTGCTTCCAGACAGTGGGAGAGGGCCTGATGGAGAGGTCCCCAAGGACAAGAACGGAGTCGTGTTCGACCTCAGGCCCTTTGTGGCAGATCTCATTGCC------------TTCATGGAAGGAAGCAGACATTCCCCACGA------TACACTCTGTGGTTCTGTGTGGGGGAATCGTGGCCCCAGGACCAGCCGTGGGTCAAGAGGCTTGTGATGGTCAAGGTT---------------GTTCCTACATGTCTTAAGGAGCTGTTAGAGATGGCCCGGGAAGGG------------------------------------GGAGCCTCATCACTGAAA---ACCGTGGACTTG---CACATCTCCAACAGCCAGCCGATCTCCCTT---------ACCTCTGACCAGTACAAGGCCTGCCTCCAGGACTTGGTGGAAGACATGGACTTCCAGGCCACTGGAGAAACCTGA---------------------

>Cow_ENSBTAG00000006633

---------------------------------------------------------------------------------------------------------------------------------------ATGGGAACCCAAAAGCCTCGGATACTGCCCTGGCTGATATCTCAGCTGGACCGAGGGGAGTTGGAGGGCGTGGCCTGGCTGGGCGAGAGCCGCACGCGTTTCCGCATCCCTTGGAAGCACGGCTTGCGGCAGGATGCCCAGCAGGAGGATTTCGGCATCTTC---------------CAG---------------------------------------------------------------------------------------GCCTGGGCTGTAGCCAGTGGTGCCTATACTCCTGGGAAGGATAAGCCCGAC---CTGCCGACCTGGAAGAGG------AATTTCCGGTCTGCCCTGAACCGGAAGGAAGTGTTGCGTTTAGCGGAGGACCACAGCAAGGACTCCCAAGACCCACACAAAATCTATGAGTTTGTGAACTCA---GGAGTCAGGGACATCCCTGAGCCAGATACCTCTCAAGAC---AATGGCAGACACAATACCTCT---GATACCCAGGAAGACACTCTGGAGAAGTTACTGAGTGACATGGACTTGAGCCCAGGA---------GGGCCCTCGAATCTGACTATGGCCTCTGAGAAGCCCCCTCAGTTCTTGCAGAGTCCCGACTCAGACATCCCTGCTCTTTGCCCAAACTCGGGACTCTCTGAAAACCCCCTGAAGCAGCTGTTGGCAAACGAGGAAGATTGGGAGTTCGAGGTGACTGCCTTCTACCGGGGCTGCCAAGTCTTCCAGCAGACTGTTTTCTGCCCTGGGGGCCTGCGGCTGGTGGGATCAGAAGCAGGGGACAGGATGCTGCCTGGGCAGCCTATACGACTGCCGGACCCTGCGACGTCCCTGACAGACAAGAGCGTGACAGACTACGTGCAGCGTGTGCTGAGCTGCCTGGGCGGGGGGCTGGCCCTGTGGCGGGCCGGGCAGTGGCTCTGCGCCCAGAGGCTGGGGCACTGCCACGTGTACTGGGCCATAGGCGAGGAGCTCCTCCCCAGCTGTGGCCACAAGCCTGACGGCGAGGTCCCGAAGGACAGGGAAGGAGGTGTGTTCAACCTGGGGCCCTTCATAACAGATCTGATCACC------------TTCATTGAAGGAAGCAGACGTTCACCACTC------TATACCCTCTGGTTCTGTGTGGGGCAGTCATGGCCCCAGGACCAGCCATGGATCAAGAGGCTTGTGATGGTCAAGGTT---------------GTCCCCATGTGTCTCAGGGTTCTTGTAGACATAGCGCGGCAAGGG------------------------------------GGTGCCTCCTCCCTGGAGAACACTGTCGACCTG---CACATTTCCAACAGCGACCCCCTCTCCCTC---------ACCCCAGACCAGTACATGGCCTGCCTCCAGGACCTGGCCGAAGACATGGATTTCTAG---------------------------------------

>Horse_ENSECAG00000018265

---------------------------------------------------------------------------------------------------------------------------------------ATGGGAACCCACAAGCCGCTGATCCTGCCCTGGCTGGTGTCGCAGCTGGACCAGGGCAGGCTGGAGGGCGTGGCCTGGCTGGACAAGGGCCGCACGCGCTTCCGCATCCCTTGGAAGCACGGCCTGCGGCAAGATGCCCAGCAGAAGGACTTCGGCATCTTC---------------CAG---------------------------------------------------------------------------------------GCCTGGGCCGAGGCCAGCGGTGCCTATACTCCAGGGAGAGATAAGCCCGAC---CTGCCGACCTGGAAGAGG------AATTTCCGGTCCGCCCTGAACCGGAAGGAAGTGTTGCGTTTAGTCGACGACCGGAGCAAGGACCCCCAGGACCCGCACAAAGTCTATGAGTTTCTGACCCCA---GCGTTTAGGGACTTGGCTCAGCCCGACACCTCTCCAGACACCGGTGGCAGATGGAGTACCTGT---GACACCCCCAAAGCTACTGTGGAGGAGTTACTGAGTGACATGACTTTAGACCCATTCCTCGATGGGGGGCCCCTGAGCCTGGCTCTGGCTCCTGAGCACCCCCCTCAGCTCTTGCTGAGCCCCAACCTAGACATTCCTGGCCCCTGCCCGAATGTGGAACCCGCTGAAAACCCACTGAGGCAGCTGTTGGTGCCCGAGGATGCGTGGGAGTTCGAGGTGACCGCCTTCTACCGGGGCCGACAAGTGTTCCAGCAGACGGTCTTCTGCCCGGAGGGCCTGCGGCTGGTGGAGCCCGAAGCAGCCGATGGGACACTGCCTGGGCAGCCAATAAGACTGCCAGACCCCGGGGTGTACCTGACAGATGGGGACGTGATGAGCCTTGTGAAGCGCGTACTGAGCAACCTGGGCGGGGGACTTGCCCTCTGGAGGGCAGGGCAGCAGCTCTGGGCCCAAAGGCTGGGGCATAGCCACGTGTACTGGGCCATGGGCGAAGAGCTGCTCCCCGACAGT---CACGGGCCCAGTGGTGAGGTGCCCAAGGACAAGGAAGGAGGTGTGTTTGACCTGGGGCCCTTCGTGGCAGATCTGATTGCC------------TTCCTTGGAGGAAGCAGACGCTCGCCACAC------TACACCCTCTGGTTCTGCTTGGGGGAGTCGTGGCCCCAGGACCAGCCGTGGACCAAGAGGCTCGTGATGGTCAAGGTT---------------GTTCCCACATGCCTCAAGGCCCTGCTAGAGATGGCGCGGATACAG------------------------------------GGTGCCTCCTCAATGGAGAACACTGTGGACCTG---CACATTTCCAACAGCCACCCCCTCTCCCTC---------ACCTCAGACCAGTACAAGGCCTACCTCGAGGACCTGGTGGAGGACATGGATATCGTT---CCTGGGGAGGCCTGG---------------------

>Dog_ENSCAFG00845023315

---------------------------------------------------------------------------------------------------------------------------------------ATGGGAACCCCAAAACCGCGGATCCTGCCCTGGCTGGTGTCACAGCTGGACCTAGGGCAGCTGGAGGGGGTGGCCTGGCTGGACGAGCGCCGCACGCGCTTTCGCATCCCTTGGAAGCATGGCTTGCGGCAGGATGCCCAGCAGGAGGACTTCGGCATCTTC---------------CAG---------------------------------------------------------------------------------------GCGTGGGCCGAGGTCAGCGGTGCCTACACTCCTGGAAAGGATAAACCTGAT---CTGCCCACCTGGAAGAGG------AATTTCCGATCTGCCCTGAACCGGAAGGAGGAGTTGCGTGTAGCTGAAGATCGGAGCAAAGACCCCCACGACCCACACAAGGTCTATGAGTTTGTGATCTCA---GGAGCTGGGAACTTGCCTGAGTTGGACACCTTTCCAGACACCAATGGGAGATACAGTACCTCG---GATACCCAGGAAGACACATTAGAGGAGTTACTGGGTGACATGGTCTTGACCCCATTCCCAGATGAGGGGCCCTCGAGCCTGGTGGTGGTCCCAGAACAGACGCCTCCACTCTTGCTGAGCCCCACCATAGACCTCCCCGCTCCCTGCCCAAACTCAGAACCCCCTGAAAACCCGCTGAAGCGGCTCTTGGTGCCTGATGAAGAGTGGGAGTTCGAGGTGACTGCCTTCTATCGGGGCCGCCAAGTCTTCCAGCAGACAGTCTCCTGCCCAAGGGGCCTGCGGCTGGTGGCAGCAGCAGGAGGGGACACGATGCTGCCTGGACAGCCAATAATACTGCCAGACCCCGGGGTGTTGGTAACAGACAAGACCGTGATGGGCTACGTGAGGCGTGTACTGAGCTGCTTGGGTGGGGGACTAGCTCTGTGGAGGGCAGGACAGCAGCTCTGGGCACGGAGGCTGGGGCACTGCCACACATACTGGGCCCTGGGCGAGGAGCTTCTCCCTGACAGCAGTCCCAGGCCTGGCGGCGAGGTCCCCAAGGATGAAGATGGAGACCTGTTCGACCTGAGGCCCTTCGTGTCAGATCTGATTGCC------------TTCATCAAAGGAAGCCGACACTCACCACGC------TACACCCTCTGGTTCTGTGTGGGGGAGCCATGGCCCCAAGACCAGCCGTGGACCAAGAAGCTGGTGATGCTCAAGGTT---------------GTTCCCACGTGCCTTAGGGCCCTGCTGGAAATGGCACGGTTAGAG------------------------------------GGCGCTTCCTCCCTGGAGAACACTGTGGACCTG---CACATTTCCAACAGCTACCCACTCACTCTC---------ACCTCGGACCAGTACAAGGCGTACCTCCAGGACCTGGTTGAGGACATGGATTTCTGGGTCACTGGGGAGGTCTGA---------------------

>MasNightMonkey_ENSANAG00000032698

---------------------------------------------------------------------------------------------------------------------------------------ATGGGAACCCCAAAGCCACGGATCCTGCCCTGGCTGGAGCTGCAGCTGAATCTGGGGCAGCTGGAGGGTGTGGCCTGGGTGAACGAGAGCCGCACGCGCTTCCGCATCCCTTGGAAACACGGCCTACGGCATGATGCACAGCAGGAGGATTTCGGAATCTTC---------------CAG---------------------------------------------------------------------------------------GCCTGGGCCGAGGCCACTGGTGCGTACGTTCCCGGGAGGGATAAGCCAGAC---CCGCCAACCTGGAAGAGG------AATTTCCGTTCTGCCCTCAACCGCAAAGATGGATTGCGTTTAGCAGATGACCGGAGCAAGGACCCTCACGACCCACATAAGATCTATGAGTTTGTGAATTCA---GGAGTTGGGGACTTTTCCCAGCCAGACACCTCTCCAGACACCAGTGGCGGAGGCAGTACTTCT---GATACCCAGGAAGACATTCTGGATGAGTTACTGGGTAACATGGTCTTGGCCCCACTCCCAGATTCGGGACCACCAGGCCTGGCCGTAGCCCCTGAGCCCTACCCTCAGCCCCTGCTGAACCCCAGCTTGGACAATCCCACGCCCTTCCCAAACCCGGAGCCCTCTGAGAACCCACTGAGGCGGCTGCTGGTGCCCGGGGAAGAGTGGGAGTTCGAGGTGACAGCCTTCTACCGGGGCCGCCAAGTCTTCCAACAGACCATCTCCTGCCCAGGGGGCCTGCGGCTGGTGGGGTCTGAAGTGGCAGACAGGACGCTGCCTGGATGGCCCATCACACTGCCAGACCCTGGCGCGTCCCTGACAGACAAGGGAGTGATGAGCTATGTGAGGCATGTGCTGAGCCGCCTGGGCGGGGGACTGGCTCTGTGGCCGGCTGGACAGCAGCTCTGGGCCCAGCGGCTGGGGCAGTGCCACACGTACTGGGCAGTGAGTGAGGAGTTGCTCCCCAACGGTGGGAATGGGCCTGATGGTGAGGTCCCCAAGGACAAGGAAGGAGGCGTGCTCGACCTGGGACCCTTCATTGTAGATCTGATTACC------------TTCACGGAAGGAAGCGGACGCTCACCACGC------TATACCCTCTGGTTCTGTGTCGGGGAGCAATGGCCCCAGGACCAGCCGTGGACCAAGAGGCTCGTGATGGTCAAGGTT---------------GTGCCCACATGCCTCCGGGCCTTGGTAGAGCTGGCCCGGGCAGGG------------------------------------GGTGCCTCATCCCTGGAGAACACCGTGGAACTG---CACATTTCCAACAGCCACCCACTCTCCCTC---------ACCTCTGACCAGTACAAGGCCTACCTGAAGGACTTGGTGGAGGACATGGATTTCCAGGGCCCTGGGGAGGCCTGA---------------------

>Marmoset_ENSCJAG00000003637

---------------------------------------------------------------------------------------------------------------------------------------ATGGGAACCCCAAAGCCACGGATCCTGCCCTGGCTGGAGTTGCAGCTGAATCTGGGGCAGCTGGAGGGTGTGGCCTGGGTGAATGAGAGCCGCACGCGCTTCCGCATCCCTTGGAAACACGGTCTACGGCATGATGCACAGCAGGAGGATTTCGGAATCTTC---------------CAG---------------------------------------------------------------------------------------GCCTGGGCTGAGGCCACTGGTGCATACGTTCCCGGGAGAGATAAGCCAGAC---CCGCCAACCTGGAAGAGG------AATTTCCGTTCTGCCCTCAACCGCAAAGATGGATTGCGTTTAGCAGATGACCGAAGCAAGGACCCTCACGACCCACATAAGATCTACGAGTTTGTGAACTCA---GCAGTTGGGAACTTTGCCCAGCCAGACACATCTCCAGACACCAGTGGCGGAGGCAGTACTTTT---GATACCCAGGAAGACATTCTGGATGAGTTACTGGGTAACATGGTCTTGGCCCCACTCCCAGATTCGGGACCACCAGGCCTGGCTGTAGCCCCTGAGCCCTACCCTCAGCCCCTGCTGAGCCCCAGCTTGGATAATCCCACGCCCTTCCCAAACCCGGAGCCCTCTGAGAATCCACTGAGGCGGCTGCTGGTGCCGGGGGAAGAGTGGGAGTTCGAGGTGACAGCCTTCTACCGGGGCCGCCAAGTCTTCCAGCAGACCATCTCCTGCCCAGGGGGCCTGCGGCTGGTGGGGTCGGAAGTGGCAGACAGGACGCTGCCTGGATGGCCCATCACACTGCCAGACCCTGGCGCATCCCTGACAGACAAGGGCGTGATGACCTATGTGAGGCACGTGCTGAGCCGCCTGGGTGGGGGACTGGCTCTGTGGCAGGCCGGACAGCAGCTCTGGGCCCAGCGGCTGGGGCAGTGCCACACGTACTGGGCAGTGAGCGAGGAGTTGCTCCCCAACAGTGGGAATGGGCCTGATGGCGAGGTTCCCAAGGACAAGGAAGGAGGCGTGCTCGACCTGGGACCCTTCATTGTAGATCTGATTACC------------TTCACTGAAGGAAGCGGACGCTCACCACGC------TATACCCTCTGGTTCTGTGTTGGGGAGCCATGGCCCCAGGACCAGCCATGGACCAAGAGGCTCGTGATGGTCAAGGTT---------------GTGCCCACATGCCTCAGGGCCTTGGTAGAGCTGGCCCGGGCAGGG------------------------------------GGTGCCTCCTCCCTGGAGAACACAGTGGAACTG---CACATTTCCAACAGCCACCCACTCTCCCTC---------ACCTCTGACCAGTACAAGGCCTACCTGAAGGACTTGGTGGAGGACATGGATTTCCAGGGCCCTGGGGAGGCCTGA---------------------

>Macaca_ENSMMUG00000022735

---------------------------------------------------------------------------------------------------------------------------------------ATGGGAACCCCAAAGCCACGGATCCTGCCCTGGCTGGTGTCGCAGCTGGACCTGGGGCAACTGGAGGGCGTGGCCTGGGTGAACGAGAGCCGCACGCGCTTCCGCATCCCTTGGAAGCACGGCCTACGGCAGGATGCACAGGAGGAGGATTTCAGAATCTTC---------------CAGGTGCGTGAGACGACCGGGGACGCTGGAAACCCTGAGCTGCGCGCGGGGCAGAAAGGACTCCTAGCGGGCCGGGCTGAGACGGCGGAGGCCTGGGCCGAGGCCACTGGTGCGTACGTTCCCGGGAGGGATAAGCCAGAC---CTGCCAACCTGGAAGAGG------AATTTCCGTTCTGCCCTCAACCGCAAAGAT---GTGTGTTTAGCAGAGGACCGGAGCAAGGACCCTCACGACCCACATAAGATCTACGAGTTTGTGAACTCA---GGAGTTGGGGACTTTTCCCAGCCAGACTACTCTCCGGACACCAATGGCGGAGGCAGTACTTCT---GATACCCAGGAAAACATTCTGGATGAGTTACTGGGTAACATGGTGTTGGCCCCACTGCCAGATCCAGGACCCCCAAGCCTGGCTGTAGCC------------CCTCAGCACTTGCAGAGCCCCAGCTTGGACAGTCCCACTCCCTTCCCAAACCTGGAGCCCTCTGAGAACCCACTGAAGCGGCTGTTGGTGCCGGGGGAAGAGTGGGAGTTCGAGGTGACAGCCTTCTACCGGGGCCGCCAAGTCTTCCAGCAGACCATCTCCTGCCCGGAGGGCCTGCGGCTGGTGGGGTCCGAAGTGGGCGACAGGACGCTGCCTGGATGGCCAATCACACTGCCAGACCCTGGCATGTCCCTGACAGACAGGGGAGTGATGAGCTACGTGAGGCACGTGCTGAGCTCCCTGGGTGGGGGACTGGCTCTGCGGCGGGCCGGGCAGCGGCTCTGGGCCCAGCGGCTGGGGCACTGCCACACATACTGGGCAGTGAGCGAGGAGCTGCTC---AACAGCGGGCATGGGCCTGATGGCGAGGTCCCCAAGGACAAGGAAGGAGGCGTGTTCGACCTGGGGCCCTTCATTGTAGATCTGATCACC------------TTCACAGAAGGAAGTGGACGCTCACCACGC------TATGCCCTCTGGTTCTGTGTGGGGGAATCATGGCCCCAGGACCAGCCATGGACCAAGAGGCTCGTGATGGTCAAGGTT---------------GTGCCCACGTGCCTCAGGGCCTTGGTAGAAATCGCCCGGGTAGGG------------------------------------GGTGCCTCCTCCCTGGAGAACACTGTGGACCTG---CACATTTCCAACAGCCACCCACTGTCCCTC---------ACCTCTGACCAGTACAAGGCCTACCTGCAGGACCTGGTGGAGGATATGGATTTCCAGGGCCCTGGGGAGACCTGA---------------------

>Gibbon_ENSNLEG00000005277

---------------------------------------------------------------------------------------------------------------------------------------ATGGGAACCGCAAAGCCACGGATCCTGCCCTGGCTGGTGTCGCAGCTGGACCTGGGGCAACTGGAGGGCGTGGCCTGGGTGAACAAGAGCCCACGG---------------------------CTACGGCAGGATGCACATCATCCTCCCGCCGCTATCCTGACACCACTGTTGCCCCAG---------------------------------------------------------------------------------------GCCTGGGCCGAGGCCACTGGTGCATATGTTCCCGGGAGGGATAAGCCAGAC---CTGCCAACCTGGAAGAGG------AATTTCCGTTCTGCCCTCAACCGCAAAGAAGGGTTGCGTTTAGCAGAGGACCGGAGCAAGGACCCTCACGACCCACATAAAATCTACGAGTTTGTGAACTCA---GGAGTTGGGGACTTTTCCCAGCAAGACACCTCTCCGGACACCAATGGCGGAGGCAGTACTTCT---GATACCCAGGAAGACATTCTGGATGAGTTACTGGGTAACCTGGTGTTGGCCCCACTCCCAGATCCGGGACCCCCAAGCCTGGCTGTAGCACCTGAGCCCTGCCCTCAGCCCCTGCGGAGCCCCAGCTTGGACAATCCCACTCCCTTCCCAAACCTGGGGCCCTCTGAGAACCCACTGAAGCGGCTGTTGGTGCCGGGGGAA---------------------------------------------------------------------------------------------------------------------------------------------------------------------------------------------------------------------------------------------------------------------------------------------------------------------------------------------------------------------------------------------GATCTGATTACC------------TTCACGGAAGGAAGCGGACGCTCACCACGC------TATGCCCTCTGGTTCTGTGTGGGGGAGTCATGGCCCCAGGACCAGCCATGGACCAAGAGGCTCGTGATGGTCAAGGTGCCTGCAGACCTGAGTGTGCCCACGTGCCTCAGGGCCTTGGTAGAAATCGCTCGGGTAGGG------------------------------------GGTGCCTCCTCCCTGGAGAACACTGTGGACCTG---CACATTTCCAACAGCCACCCACTCTCCCTC---------ACCTCTGACCAGTACAAGGCCTACCTGCAGGACTTGGTGGAGGGCATGGATTTCCAGGGCCCTGGGGAGGCTTGA---------------------

>Tarsier_ENSTSYG00000001716

---------------------------------------------------------------------------------------------------------------------------------------ATGGCAACCTCAAAGCCGCGGATCCTGCCCTGGCTGGTGTCGCAGCTGGACCACGGGCAGCTGGAGGGCGTGGCCTGGGTGAACGAGAGCCGCACGCGCTTCCGCATCCCTTGGAAGCACGGCCTGCGGCAGGACGCACAGCAGGAGGACTTCGGAATCTTC---------------CAG---------------------------------------------------------------------------------------GCCTGGGCTGAGGCCACCGGTGCTTACACTCCCGGGAAGGACAAGCCCGAC---CTGCCAACCTGGAAGAGG------AATTTCCGGTCTGCCCTTAATCGGAAAGAAGGGGTGCGTCTAGCAGTGGACCAGAGCAAGGACCCTCACGACCCACATAAGATCTACGAGTTTGTGACCCCA---GGATTTGGGGACTTTTCCCAGTGGGACACCTCTCCAGAAACCAGTGGCCGAGGCAGTACCTCT---GATATCCAGGAAGACATTCTGGATGAGTTACTGGGTAACATGGCCTTGGCCCCACTCCCAGATGGGCCC---TCGGGCCTGACTGTAGCCCCTGAGCCCTGCCCTCAGCTCTTGCTGAGCCCCAGGTCAGACAATCCCGTTCCCTGCTCAAACCTGGGGGCCCCTGAAAATCCACTGAGGCAGCTATTGGTGCCAGAGGAAGCGTGGGAGTTTGAGGTGACGGCCTTCTACCGGGGCCGCCAAGTCTTCCAGCAGACCATCTCACGCCCAGGGGTCCTGCGGCTGGTAGGGCCCGAAGTAGGGGACAAGACGCTGCCTGGATGGCCAATAACACTGCCAGACCCTGGGGTGTCCCTGACAGACAGGGGAGTCATGGGCTACGTGAGGCGTGTGCTGCACTGCCTTGGTGGGGGACTGGCTCTGTGGCGGGCCGGGCAGCAGCTCTGGGCCCAGCGGCTGGGGCACTCCCACACGTACTGGGCCGTGGGCGAGGAGCTGCTCCCCGACAGCGGGCATGGGCCTGATGGTGAGGTTCCCAAGGACAAGGAAGGCAGCGTGTTCGACCTGGGGCCCTTCGTGGCAGATCTGATTACC------------TTCATAGAAGGAAGTGGACGCTCACCACGC------TACACCCTCTGGTTCTGTGTGGGGGAGTCGTGGCCCCAGGACCAGCCATGGATTAAGAGGCTCGTGATGGTCAAGGTG---------------GTGCCCACGTGCCTCAGGGCCCTGTTGGACATGGCCCGACTCGGG------------------------------------GGGGCCTCCTCACTGGAGAACACCGTGGACCTG---CACATTTCCAACAGCCAGCCCCTTTCCCTC---------ACCTCTGACCAGTACAAGGCCTACTTGCAGGACTTGGTAGAGGACATGGATTTTCAGGTCACTGGGGAGGCCTGA---------------------

>MouseLemur_ENSMICG00000001752

---------------------------------------------------------------------------------------------------------------------------------------ATGGCAACCCCAAAGCCACGGATCCTGCCCTGGCTGGTGTCGCAGCTGGACATGGGGCACCTGGAGGGCGTGGCCTGGGTGGACGAGAGCCGCACGCGCTTCCGTATTCCTTGGAAGCACGGCCTGCGGCAGGATGCTCAGCAGGAGGATTTCGGAATCTTC---------------CAG---------------------------------------------------------------------------------------GCCTGGGCCGAGGCCAGCGGTGCCTACACTCCCGGGAAGGATAAGCCGGAC---TTGCCAACCTGGAAGAGG------AATTTCCGGTCCGCCCTGAACCGGAAGGAAGTGGTGCGTTTAGCCGAGGACCGGAGCAAGGACCCTCACGACCCGCATAAAATCTATGAGTTTGTGACTTCA---GGAGTTGGGGACTTTTCCCAGCTGGACACCTCTTCAGAGACCAGTGGTGGAGGCAGTACCTCT---GATACCCAGGAAGACATGCTGGAGGAGTTACTGGACAACATGGTCTTGGCCCCAGCCCCGAACGGGCAGACCCCAAGCCTCGCTGTGGCCCCTGAGCCCCACCCTCAGCTCTTGCTGAGCCCCAGCTTGGACAGTCCCGCTCCCTGCCCAAACCTGGAGCTCCCTGAAAACCCGCTGAGGCTGCTGCTGGTGCCAGAGGAAGAGTGGGAGTTTGAGGTGACAGCCTTCTACCGGGGCCGCCAAGTTTTCCAGCAGACCATCTCATGCCCAGGGGGCCTGCGGCTGGTGGGGTCCGAAGTGGCAGACAGCATGCTCCCTGGACAGCCAATAACACTGCCAGACCCTGGGCTGTTCCTGACAGACAGGGGAGTGACGGGCTACGTGAGGCGTGTGCTCAGCTGCCTGGGCGGGGGGCTGGCTCTGCGGCGGGCCGGGCAGCGGCTCTGGGCCCAGCGCCTGGGGCACTGCCACACATACTGGGCCATGGGCGAGGAGCTGCTCCCCAGCAGTGGGCATGGGGCTGATGGCGAGGTCCCTAAGGACAAGGAAGGAAGCGTGTTCGACTTGGGGCCCTTCGTGGCAGATCTGATTACC------------TTCATTGAAGGAGGCAGTCGCTCACCACGC------TACACCCTCTGGTTCTGCATCGGGGAGCCATGGCCCCAGGACCAGCCATGGACCAAGAGGCTCGTGATGGTTAAGGTG---------------GTTCCCACGTGCCTCAGGGCCCTGTTGGACATGGCCCGGTCAGGG------------------------------------GGTGCCTCCTCCCTGGAGAACACCGTGGACCTG---CACATTTCCAACAGCCACCCGCTCTCCCTC---------ACCTGCGACCAATACAAGGCCTACCTACAGGACCTGGTTGAGGACATGGATTTCGTC---CCTGGGGCAGCCTGA---------------------

# IRF4

>Opossum_ENSMODG00000008383

------------------------------------------------------------------------------------------------------------------------------------------------------------------------------------------------------------------------------------------------------------------------------------------------------------------------------------------------------------------------------------------------------------------------------------------------------------------------------ATGAACTTGGAGGGAGGTGGTGGCAGTGGAGGCGGTGGCAGCAGCAACAGCAGCGAGTGCGGAATGAGTTCTGTGAGTTGCGGCAACGGCAAACTCCGCCAGTGGCTGATCGATCAGATTGACAGTGGCAAGTACCCGGGCCTCGTGTGGGAGAATGAGGAGAAAAGCATCTTCCGCATCCCCTGGAAACACGCCGGCAAGCAAGACTACAACAGAGAGGAAGATGCTGCCCTCTTCAAGGCTTGGGCCCTCTTTAAGGGGAAATTCAGGGAAGGTATTGACAAACCAGACCCTCCAACCTGGAAGACAAGGTTGCGCTGTGCTCTCAACAAGAGTAATGACTTTGAAGAGTTAGTTGACAGAAGCCAATTGGATATCTCAGATCCATATAAAGTTTACAGAATTGTTCCTGAAGGGGCCAAAAAAGGAGCTAAACAACTGGGCTTGGAGGATTCCCAGATAATTATGAACCACCCTTTCCCAATGCCTCCTCACTATACATCACTACAAACT---CAGGTTCCAAACTATATGATGCCC---CATGACCGTAATTGGAGAGATTTTGTTCCGGAGCAGCCTCACCCAGAAATCCCCTATCAATGTACAACTGTCCCTTTTGGACCTCGAAGTCACCATTGGCAGGGCCCTGCTTGTGAGAATGGTTGCCAGGTGACAGGCACCTTTTATGCTTGTGCGCCACCTGAGTCACAGGCTCCTGGAATACCCATAGAGCCAAGCATAAGATCTGCTGAAGCCCTGGCCCTCTCAGATTGCCGGCTCCATATCTGTTTATATTATCGTGAAATACTGGTGAAGGAGATAACCACCTCAAGCCCTGAAGGCTGTAGGATATCTCATGGCCAGAGTTATGATGCCAGTACCCTGGATCAGGTTCTCTTCCCCTATCCAGAGGATAATGTCCAGAGGAAAAACATCGAAAAATTGCTGAGCCATTTGGAAAGGGGAGTCATTCTCTGGATGGCACCCGATGGGCTGTATGCTAAGAGACTTTGTCAAAGCAGGATCTACTGGGATGGACCCTTGGCACTATGCAGTGACCGTCCTAATAAGTTGGAAAGGGACCAGACCTGCAAACTTTTTGACACACAACAATTTCTATCAGAGTTGCAAGTGTTTGCCCATCATGGTCGCCCTTCACCAAGATTCCAAGTAGCTCTGTGCTTTGGAGAAGAATTTCCAGACCCCCAGAGGCAAAGGAAGCTTATCACAGCTCACGTTGAACCATTACTAGCCAAACAGCTCTATTACTTTGCTCAGCAAAATAGTGGACATTTTCTGAGGGGCTTTGATTTACCAGAACATATTACCGGGCCAGAGGATTATCATCGACCAATTCGACATTCTTCCATCCAAGAATGA

>Mouse_ENSMUSG00000021356

------------------------------------------------------------------------------------------------------------------------------------------------------------------------------------------------------------------------------------------------------------------------------------------------------------------------------------------------------------------------------------------------------------------------------------------------------------------------------ATGAACTTGGAGACG---GGCAGCCGGGGCTCA---------------------GAGTTCGGCATGAGCGCAGTGAGCTGCGGCAATGGGAAACTCCGACAGTGGTTGATCGACCAGATCGACAGCGGCAAGTACCCCGGGCTGGTGTGGGAGAACGAGGAGAAGAGCGTCTTCCGCATCCCGTGGAAACACGCGGGCAAGCAGGACTACAATCGTGAGGAGGACGCTGCCCTCTTCAAGGCTTGGGCATTGTTTAAAGGCAAGTTCCGAGAAGGGATCGACAAGCCAGATCCTCCTACTTGGAAGACAAGATTACGATGTGCTCTGAACAAGAGCAATGACTTTGAGGAATTGGTCGAGAGGAGCCAGCTGGATATCTCTGACCCATACAAGGTGTACAGGATTGTTCCAGAGGGAGCCAAAAAAGGAGCAAAGCAGCTCACTTTGGATGACACACAGATGGCCATGGGCCACCCCTACCCCATGACAGCACCTTATGGCTCTCTGCCAGCCCAGCAGGTTCATAACTACATGATGCCACCCCATGACAGGAGCTGGAGGGATTATGCCCCTGACCAGTCACACCCAGAAATCCCATATCAATGT---CCTGTGACGTTTGGCCCACGAGGCCACCACTGGCAAGGCCCATCTTGTGAAAATGGTTGCCAGGTGACAGGAACCTTTTATGCTTGTGCCCCACCTGAGTCCCAGGCTCCTGGAATCCCCATTGAGCCAAGCATAAGGTCTGCTGAAGCCTTGGCGCTCTCAGACTGCCGGCTGCATATCTGCCTGTATTACCGGGACATCCTCGTGAAAGAGCTGACCACGACGAGCCCTGAAGGCTGCCGGATCTCCCACGGACACACCTATGATGTTAGCAACCTGGACCAGGTCCTGTTTCCCTACCCGGACGACAATGGACAGAGGAAGAACATTGAGAAGTTGCTGAGCCACCTGGAGAGGGGACTGGTCCTCTGGATGGCTCCAGATGGGCTTTATGCCAAAAGACTCTGCCAGAGTAGGATCTACTGGGATGGGCCCCTGGCACTGTGCAGCGATCGGCCCAACAAGCTAGAAAGAGACCAGACTTGCAAGCTCTTTGACACACAGCAGTTTCTATCAGAGCTGCAAGTGTTTGCTCACCATGGCCGGCCAGCACCGAGATTCCAGGTGACTCTGTGCTTTGGTGAGGAGTTTCCAGACCCTCAGAGACAGAGGAAGCTCATCACAGCTCATGTGGAACCTCTGCTAGCCAGACAACTGTATTACTTTGCTCAACAAAACACTGGACATTTCCTGAGGGGCTACGAGTTACCTGAACACGTTACCACTCCA---GATTACCACCGCTCCCTCCGTCATTCTTCCATCCAAGAGTGA

>Rat_ENSRNOG00000061070

---------------------------------------------------------------------------------------------------------------------------------------------------------------------------------------------------------------------------------------------------------ATGCTCCCCACACCCTCGTCAAAGTACCTTCTGTTGGGGGACTCCGGAGACACTGGCACTGCACGCGAGCACCTGGCAGCCTCAGAGACTCCGGGGCCTCGTGGGCACTGCAAGAGTTTGGGGACAGGGCTCCCCCAAGAACGATGCGACGGTGGCTGCAGGGAAAGTTTGTCTCCCCGCAGCGCACGAGCGCGGAGGAGGTCAGCACGCGTCATGAACTTGGAGACG---GGCAGCCGGGGCTCA---------------------GAGTTCGGCATGAGCGCGGTGAGCTGCGGCAACGGGAAACTCAGACAGTGGCTGATCGACCAGATCGACAGCGGCAAGTACCCCGGGCTGGTGTGGGAGAACGAGGAGAAGAGCGTCTTCCGCATCCCGTGGAAACACGCGGGCAAACAGGACTACAACCGGGAGGAGGACGCAGCCCTCTTCAAGGCTTGGGCACTATTTAAAGGCAAGTTCCGAGAAGGGATTGACAAGCCAGATCCTCCTACTTGGAAGACAAGATTACGATGCGCTCTGAACAAGAGCAATGACTTTGAGGAACTGGTAGAGAGGAGTCAGCTGGATATCTCCGACCCATACAAGGTCTACAGGATTGTTCCAGAGGGAGCCAAAAAAGGAGCAAAGCAGCTCACTTTGGAAGACACACAGATGGCCATGGGCCACCCCTACCCCATGACAGCACCTTATGGCTCTCTGCCAACCCAGCAGGTTCATAACTACATGATGCCACCCCATGACAGGAGCTGGAGGGATTATGCCCCTGACCAGTCACACCCAGAAATCCCATATCAATGT---CCTGTGACGTTTGGCCCGCGCGGCCACCACTGGCAAAGCCCATCTTGTGAAAATGGTTGCCAGGTGACAGGAACCTTTTATGCTTGTGCCCCACCTGAGTCCCAGGCTCCTGGAATCCCCATTGAGCCAAGCATAAGGTCTGCTGAAGCCTTGGCGCTCTCAGACTGCCGGCTGCATATCTGCCTGTATTACCGGGACATCCTCGTGAAGGAGCTGACCACGTCCAGCCCCGAGGGCTGCCGGATCTCCCACGGACATACCTATGATGTTAGCAACCTGGACCAAGTCCTGTTCCCCTACCCCGACGACAATGGGCAGAGGAAGAACATTGAGAAGTTGCTGAGCCACCTGGAGAGGGGACTGGTCCTCTGGATGGCCCCAGATGGGCTTTATGCCAAAAGACTGTGCCAGAGCAGGATCTACTGGGATGGGCCCCTGGCACTGTCCAGTGATCGGCCCAACAAACTAGAAAGAGACCACACTTGCAAGCTCTTTGACACGCAGCAGTTTCTATCAGAGCTACAAGTGTTTGCTCACCACGGCCGGCCAGCACCGAGATTCCAGGTGACTCTGTGCTTTGGGGAGGAGTTTCCAGACCCTCAGAGACAGAGGAAGCTCATCACAGCTCATGTGGAACCTCTGCTAGCCAGACAACTGTATTTTTTTGCTCAACAAAACAGTGGACATTTTCTGAGGGGCTATGAATTACCTGAACACATTAGCACTCCA---GATTACCACCGGTCCCTCCGTCATTCTTCCATCCAAGAGTGA

>Cow_ENSBTAG00000002929

------------------------------------------------------------------------------------------------------------------------------------------------------------------------------------------------------------------------------------------------------------------------------------------------------------------------------------------------------------------------------------------------------------------------------------------------------------------------------ATGAACCTGGAGGGC---GGCAGCCGAGGCGGC---------------------GAGTTCGGCATGAGCTCCGTGAGCTGCGGCAACGGGAAGCTCCGCCAGTGGCTCATCGACCAGATCGACAGCGGCAAGTACCCGGGGCTGGTGTGGGAGAACGAGGAGAAGAGCATCTTCCGCATCCCCTGGAAGCACGCGGGCAAGCAGGACTACAACCGCGAGGAGGACGCCGCGCTCTTCAAGGCTTGGGCACTGTTTAAAGGGAAGTTTCGAGAAGGCATCGACAAGCCAGACCCTCCCACCTGGAAGACACGCCTGCGGTGTGCTCTGAACAAGAGCAATGACTTCGAGGAGCTGGTGGAGCGGAGCCAGCTGGACATCTCGGACCCCTACAAAGTGTACCGGATCGTCCCCGAGGGAGCCAAGAAAGGAGCGAAGCAGCTGACCCTGGAGGACCCGCAGATGCCCATGAGCCACCCGTACAGCATGCCGACTCCTTACCCCTCACTGCCTGCTCAGCAGGTTCATAACTACATGATCCCGCCCCACGACCGGGGCTGGAGGGAGTTCGTCCCAGACCAGCCGCACGCAGAGATCCCGTACCAGTGT---CCCGTGACCTTCGGACCCCGCGGCCACCACTGGCAAGGCCCAGCCTGTGAAAATGGTTGCCAGGTCACAGGAACCTTTTATGCTTGTGCCCCGCCTGAGTCCCAGGCCCCCGGGATCCCCATAGAGCCAAGCATAAGGTCTGCCGAAGCCTTGGCCCTCTCAGACTGCCGGCTCCACATCTGCCTCTACTACCGGGAAGTCCTGGTCAAGGAGCTGACCACGTCCAGCCCCGAGGGCTGCCGGATCTCCCACGGCCACACCTACGACGCCAGCAGCCTGGACCAGGTCCTGTTTCCCTACCCAGAGGACAGCAGCCAGAGGAAGAACATCGAGAAGCTGCTGAGCCACCTGGAGAGGGGCGTGGTCCTCTGGATGGCCCCCGATGGGCTTTATGCCAAGAGACTGTGCCAGAGCAGAATCTACTGGGACGGGCCCCTGGCGATATGCAGCGACCGGCCCAACAAGCTGGAGAGAGACCAGACCTGCAAGCTGTTTGACACGCAGCAGTTCCTGTCAGAGCTGCAGGCGTTCGCGCACCACGGCCGGCCACTGCCAAGGTTCCAGGTGACTCTGTGTTTCGGGGAGGAGTTCCCAGATCCTCAGAGGCAGCGCAAGCTCATCACTGCCCACGTTGAGCCTCTGTTGGCCAGACAGCTGTATTATTTTGCTCAGCAAAACAGCGGACACTTCCTCAGGGGCTATGACCTGCCCGAGCACGTCGGCGGCCCGGAGGACTTCCACAGGCCCCCCCGCCACTCCTCCATCCAGGAGTGA

>Tarsier_ENSTSYG00000006495

------------------------------------------------------------------------------------------------------------------------------------------------------------------------------------------------------------------------------------------------------------------------------------------------------------------------------------------------------------------------------------------------------------------------------------------------------------------------------------------------------------------------------------------------------------------------------------------------------------------------------------------------------------------------------------------------------------------------------GCCTGGGCACTATTTAAAGGAAAGTTCCGAGAAGGCATTGATAAGCCTGACCCCCCAACCTGGAAGACACGCTTACGATGTGCTCTGAACAAGAGCAACGACTTCGAGGAGCTGGTGGAGAGGAGCCAGCTGGACATCTCAGACCCTTACAAGGTCTATAGGATTGTTCCCGAGGGAGCCAAGAAAGGAGTGAAGCAGCTCACTCTGGAGGATCCACAGATGTCCATGAGCCACTCCTACTCCATGGCAACTCCCTACACCTCCCTCCCTGCTCAGCAGGTTCATAACTATGTGATGCCAACCCTCGATCGCAGCTGGCGGGATTATGTCCCTGATCAGCCGCACCCAGAAATGCCATATCAATGT---CCTATGACGTTTGGACCCCGAAGCCACCACTGGCAAGGCCCGGCTTGTGAAAATGGTTGCCAGGTGACAGGAACCTTTTATGCTTGTGCCCCACCTGAGTCCCAGGCTCCTGGAATCCCCACAGAGCCAAGCATAAGGTCTGCTGAAGCTTTGGCGCTCTCAGACTGCCGGATGCACATCTGTCTGTACTACCGGGAAATCCTGGTGAAGGAGCTGACCACGTCTAGCCCTGAGGGCTGTCGGATCTCCCATGGACACACGTATGATGCCAGCAGCCTGGACCAGGTGCTGTTCCCCTACCCAGAGGACAACAGCCAGCGGAAGAATATCGAGAAGTTGCTGAGCCACATGGAGAGGGGTGTGGTCCTCTGGATGGCCCCTGATGGGCTGTACGCTAAGAGACTGTGCCAGAGCAGGGTCTACTGGGATGGGCCTCTGGCCCTGTGCAGCGACCGACCCAACAAACTGGAGAGAGACCAAACCTGCAAGCTCTTCGACACGCAGCAGTTTTTGTCAGAGCTGCAGGCATTTGCCCACCACAGCCGCCCTCTACCAAGATTCCAGGTGACCTTGTGCTTCGGGGAAGAGTTTCCAGACCCCCAGAGACAAAGGAAGCTCATCACAGCTCACGTGGAACCTCTGCTAGCCAGACAACTATATTATTTTGCTCAACAAAACAGCGGACATTTCCTGAGGAGCTATGATTTACCCGAGCACATTAGCAGTCCAGAGGATTATCACAGGTCTATCCGTCATTCCTCCATTCAAGAATGA

>Horse_ENSECAG00000016808

ATGGGCAAAGAGGAGCGGGAACTACCAGTGAGTATGCAAACGGAGTGCCAAGAGTGGTGGCGGCTGCATTTGGGAGCGTGCGTAAAGGCGAGAGTTGTGTGTCTGGACAGCCCTTGGGCTGCGGGGTGCGTAACAAGAATGGAGAGTCCTGAAGGTGGGGTAGAAGGATGGACTCCACCACGGGCGCGCTCGGGAGGTGGCAGGACTCTGCGAGCCAAGGGGACGAACCCGGGCCGGCTGAGCATGCTGTCTCTCAGGGGCCTGGGGCCACGCCGACTCTGTAGCGCGCCTCGCTGCCTCAGAAACTCCGGGACCTCGTGGTCACTGCCAGAGGGTGTTGAGATGGGGCGCCCCGAGCGCTGTGCCGCGCAGGCTGAAAGGCGGCCCTCCCCTCCCTGCAGCTCAGAGAGGGTGCCAGAGGCGGATCCTGAGCGCGTGAGGGCGACGGTCGGCGCGCTGGGCATGAACCTGGAGGGCGGCGGCGGCCGAGGCGGA---------------------GAGTTCGGCATGAGCTCGGTGAGCTGCGGCAACGGGAAACTCCGCCAGTGGCTGATCGACCAGATCGACAGCGGCAAGTACCCAGGGCTGGTGTGGGAGAACGAGGAGAAGAGCATCTTCCGCATCCCCTGGAAGCACGCGGGCAAACAGGACTACAACCGCGAGGAGGACGCCGCCCTCTTCAAGGCTTGGGCACTATTTAAAGGAAAGTTCCGAGAGGGCATCGATAAGCCAGACCCGCCTACCTGGAAGACACGTTTGCGATGCGCTTTGAACAAGAGCAATGACTTTGAGGAGCTGGTGGAGAGGAGCCAGCTGGACATCTCAGACCCCTACAAAGTATACAGGATTGTTCCCGAAGGAGCCAAAAAAGGAGCAAAGCAGCTGGCGCTGGAGGACCCACAGATGGCCATGAGCCACCCCTACAGCATGACAGCTCCTTACCCCTCACTCCCAGCTCAGCAGGTTCATAACTACATGATGACACCCCACGACCGAAGCTGGAGGGAGTACGTCCCTGATCAGCCACACCCGGAAATCTCGTATCAATGT---CCTGTGCCGTTTGGACCCCGAAGCCACCACTGGCAAGGCCCGGCTTGTGAAAATGGTTGCCAGGTGACAGGAACCTTTTATGCTTGTGCCCCGCCTGAGTCCCAGGCCCCCGGAATCCCCATAGAGCCAAGCATAAGGTCTGGTGAAGCCTTGGCGCTCTCAGACTGCCGGCTTCACATCTCCTTGTACTACCGGGAAATCCTGGTGAAAGAGCTGACCACGTCAAGCCCCGAAGGCTGTCGAATCTCCCATGGGCAAACCTATGACGCCAGTAACCTGGACCAGGTCCTCTTCCCCTATCCGGAGGACAACGGCCAGAGGAAAAACATCGAGAAACTGCTGAGCCACCTGGAAAGGGGCGTGGTCCTCTGGATGGCCCCTGATGGGCTTTATGCCAAAAGACTATGTCAGAGCAGGATCTACTGGGATGGGCCCCTGGCGCTGTGCAGCGACCGGCCCAACAAACTGGAGAGAGACCAGACCTGCAAGCTCTTTGACACACCGCAGTTTTTGTCAGAGCTGCAAGCGTTTGCTCACCATGGCCGTCCCCTGCCAAGGTTCCAAGTAACTCTGTGCTTCGGGGAAGAGTTCCCAGACCCTCAGAGGCAGCGGAAGCTCATCACTGCTCACGTTGAACCTCTACTAGCCAGACAACTGTATTATTTTGCTCAACAAAACAGTGGCCATTTCCTAAGGGGCTACGATGTGCCTGAACACATCAGCAGTCCGGAGGACTATCACAGGTCGATCCGCCATTCTTCCATTCAAGAATGA

>Dog_ENSCAFG00845015916

---------------------------------------------------------------------------------------------------------------------------------------------------------------------------------------------------------------------------------------------------ATGGGGAGGCCGGGCCCTTGGAGCGCACGGACGGCGGGCGCCCCACGTGCCGGGCGTCTGCGCGGGGCTGGGTGCTGGGTGCTGGGTGCTGGGTGCCGGGTGCCGGGGCCAGGGGCCGGGTGCCGGGTGCAGGGCCGGGCGCGGGTCGCGGGGTGCGGCGCTGACGCCGGGTGTCGGGGTGTCGGGGCGCGGGCAGGCGGCGGCGCAGCGCGGCGGGGCATGAACCTGGAGGGC---GGCGGCCGCGGCGGG---------------------GACTTCGGCATGAGCGCCGTGAGCTGCGGCAACGGGAAGCTCCGCCAGTGGCTCATCGACCAGATCGACAGCGGCAAGTACCCGGGGCTCGTGTGGGAGAACGAGGACAAGAGCATCTTCCGCATCCCCTGGAAGCACGCGGGCAAGCAGGACTACAACCGCGAGGAGGACGCGGCGCTCTTCAAGGCTTGGGCACTATTTAAAGGAAAGTTCCGAGAAGGCATAGACAAGCCCGACCCTCCGACCTGGAAGACGCGCTTACGATGCGCTTTGAACAAGAGCAATGACTTTGAGGAGCTGGTGGAGAGGAGCCAGCTGGACATCTCGGACCCCTACAAAGTGTACAGGATTGTGCCCGAGGGCGCCAAAAAAGGAGCAAAGCAGCTCACCCTGGAGGACCCGCAGATGACCATGAGCCACCCCTACACCATGACAGCTCCCTACACCTCGCTGCCAGCGCAGCAGGTTCATAACTACATGATGCCTCCCCACGAGCGAGGCTGGAGGGAGTACATCCCCGATCAGCCCCACGCGGAGATCCCGTATCAGTGT---CCGGTGACGTTTGGATCCCGCGGCCACCACTGGCAGGGCCCGGCTTGTGAAAACGGTTGCCAGGTGACAGGAACCTTTTATGCTTGTGCCCCGCCTGAGTCCCAGGCCCCAGGAATCCCCATAGAGCCAAGCATAAGGTCTGCCGAAGCCTTGGCGCTCTCAGACTGCCGACTGCACATCTGCTTGTACTACCGGGAGATCCTGGTGAAAGAGCTGACCACATCGAGCCCAGAAGGCTGTCGGATCTCCCACGGGCACACTTACGATGCCAGTAACCTGGACCAGGTCCTGTTCCCCTACCCAGAGGACAATGGCCAGAGGAAAAACATCGAGAAACTTCTGAGCCACTTGGAGAGGGGTGTGGTCCTCTGGATGGCCCCTGATGGGCTTTATGCCAAAAGACTGTGCCAGAGCAGGATCTACTGGGATGGGCCCCTGGCACTGTGCAGTGACAGGCCCAACAAACTGGAGAGGGACCAGACCTGCAAGCTCTTCGACACACAGCAGTTTTTAGCAGAGCTGCAAGCTTTTGCTCACCATGGCCGTCCCCTGCCAAGATTCCAGGTAACTCTGTGCTTCGGGGAAGAGTTTCCAGATCCTCAGAGGCAAAGGAAGCTCATCACTGCTCACGTCGAACCTCTACTCGCCAGACAGCTGTACTATTTTGCTCAGCAAAACAGTGGACACTTCCTGCGGGGCTACGACCTGCCCGAGCACATCGGCAGCCCCGAGGACTACCACAGGTCTATTCGCCACTCCTCCATTCAAGAATGA

>Rabbit_ENSOCUG00000009374

------------------------------------------------------------------------------------------------------------------------------------------------------------------------------------------------------------------------------------------------------------------------------------------------------------------------------------------------------------------------------------------------------------------------------------------------------------------------------ATGAACCTGGAGGGC---GGCGGCCGGGGCGCG---------------------GAGTTCGGCATGAGCGCGGTGAGCTGCGGCAACGGGAAGCTCCGCCAGTGGCTGATCGACCAGATCGACAGCGGCAAGTACCCGGGGCTGGTGTGGGAGAACGAGGACAAGAGCGTCTTCCGCATCCCCTGGAAGCACGCGGGCAAGCAGGACTACAACCGCGAGGAGGACGCCGCCCTCTTCAAGGCTTGGGCACTATTTAAAGGCAAGTTCCGAGAAGGCATCGATAAGCCGGACCCTCCCACCTGGAAGACGCGCTTGCGCTGTGCCCTGAACAAGAGCAATGACTTTGAGGAGCTGGTGGAGAGGAGCCAGCTGGACATCTCAGACCCCTACAAAGTGTACAGGATCGTCCCCGAGGGAGCCAAGAAAGGGGCCAAGCAGCTCGCGCTGGAGGACCCACAGATGACCATGGGCCACCCTTACACCATGACGGCTCCCTACACCTCGCTCCCAGCCCAGCAGGTTCATAACTACATGATGCCGCCCCACGACCGCAGCTGGAGGGATTACGTCCCCGATCAGCCACACCCAGAAATCCCCTATCAATGT---CCCGTGACGTTTGGCCCCCGCAGCCACCACTGGCAAGGCCCAACCTGTGAAAACGGTTGCCAGGTGACAGGAACCTTTTATGCTTGTGCCCCACCCGAGTCCCAGGCTCCCGGGATCCCCATTGAGCCAAGCATAAGGTCTGCTGAAGCCCTGGCGCTCTCAGACTGCCGCCTGCACATCTGCCTGTACTACCGGGAAATCTTGGTGAAGGAGCTGACCACGTCCAGCCCCGAGGGCTGCCGGATCTCCCACGGACACACTTACGACGCCAGCAACCTGGACCAGGTCCTGTTCCCTTACCCGGAGGACAATGGGCAGAGGAAAAACATCGAGAAGCTGCTGAGCCACCTGGAGCGGGGCGTGGTCCTCTGGATGGCCCCCGACGGGCTGTACGCCAAAAGACTGTGCCAGAGCAGGATCTACTGGGACGGGCCTCTGGCCCTGTGCAGCGACCGGCCCAACAAACTGGACAGAGACCAGACCTGCAAGCTCTTTGACACACAGCAGTTCCTCTCAGAGCTGCAAGCGTTCGCTCACCATGGCCGTCCACTGCCGAGATTCCAGGTGACCTTGTGCTTCGGGGAGGAGTTTCCAGACCCTCAGAGGCAGAGGAAGCTCATCACGGCTCACGTAGAACCTCTGCTAGCCAGACAACTGTATTACTTTGCTCAGCAAAACAGTGGACATTTCCTGAGGGGCTACGATGTCCCTGAACACATCAGCAGCACAGAGGATTACCACAGATCCATCCGCCACTCTTCCATTCAGGAATGA

>Elephant_ENSLAFG00000017235

------------------------------------------------------------------------------------------------------------------------------------------------------------------------------------------------------------------------------------------------------------------------------------------------------------------------------------------------------------------------------------------------------------------------------------------------------------------------------ATGAACTTGGAGGGC---AGCGGCCGTGGCGGT---------------------GAGTTCGGCATGAGTTCCGTGAGCTGTGGCAACGGGAAACTCCGCCAGTGGCTGATCGACCAGATCGACAGCGGCAAGTACCCAGGGCTGGTGTGGGAGAACGAGGAGAAGAGCATCTTCCGCATCCCCTGGAAGCACGCGGGCAAGCAGGACTACAACCGCGAGGAGGACGCCGCGCTCTTCAAGGCTTGGGCACTATTTAAAGGAAAGTTTCGAGAAGGCATTGATAAACCAGACCCTCCTACCTGGAAGACACGTTTGCGATGTGCTTTGAACAAGAGCAATGATTTTGAGGAATTGGTTGAGAGGAGCCAGCTGGATATCTCAGATCCGTACAAAGTCTACAGGATCGTTCCCGAGGGAGCCAAAAAAGGAGCAAAGCAGCTCACCCTGGAGGACCCCCAGATGGCCATGAGCCACCCGTACACCGTGGCAGCTCCTTACCCTTCACTGCCATCTCAGCAGGTTCATAACTACATGATGCCGCCCCATGACCGAAGCTGGAGGGAGTATGTCCCTGATCAGCCACACCCAGAAATCCCCTACCAATGT---CCTGTGACGTTTGGGCCCCGCAGCCATCACTGGCAAGGCCCCACTTGTGAAAATGGTTGCCAGGTAACAGGAACCTTTTATGCTTGTGCCCCACCTGAGTCCCAGGCCCCTGGAATCCCCATAGAGCCAAGCATAAGGTCTGCTGAAGCCTTGGCGCTCTCAGATTGCCGGCTCCACATTTGCTTGTACTACCGGGAAATCCTGGTGAAGGAGCTGACCACGTCAAGCCCTGAAGGCTGCAGAATCTCCCATGGGCACACCTACGATGCCAGCAACCTGGACCAAGTTCTTTTCCCTTATCCAGAGGACAGTGGCCAGAGGAAAAATATAGAGAAACTGCTGAGCCACCTGGAAAGGGGTGTGGTCCTCTGGATGGCCCCCGATGGGCTCTACGCCAAAAGACTTTGCCAGAGCAGGATCTACTGGGATGGGCCCCTGGCCCTGTGCAGTGACCGGCCCAACAAGCTGGAGAGAGATCAGACCTGCAAGCTTTTTGATACACAGCAGTTCTTATCAGAGCTGCAAGCATTTGCTCATCACGGCCGCTCCCTCCCGAGGTTCCAGGTGACTCTGTGCTTTGGTGAGGAGTTCCCAGACCCTCAGAGACAGAGGAAGCTCATCACAGCTCAGGTTGAACCTCTACTAGCCAGACAGCTATATTATTTTGCTCAACAAAACAGTGGACATTTCCTGAGAGGCTATGACTTACCCGAACACATCAGCAGTCCAGAGGATTATCACAGATCCATTCGCCACTCCTCCATTCAAGAA---

>Megabat_ENSPVAG00000005078

------------------------------------------------------------------------------------------------------------------------------------------------------------------------------------------------------------------------------------------------------------------------------------------------------------------------------------------------------------------------------------------------------------------------------------------------------------------------------ATGAACTTAGAGGGCAGTGGAGGCCGAGGCGGA---------------------GAGTTCGGCATGAGCTCTGTGAGCTGTGGCAATGGGAAACTCCGCCAGTGGCTGATCGACCAGATCGACAGTGGCAAGTATCCAGGGCTGGTGTGGGAGAACGAGGAGAAGAGCATCTTTCGCATCCCCTGGAAGCACGCGGGCAAGCAGGACTACAACCGTGAGGAGGACGCCGCCCTCTTCAAGGCTTGGGCACTATTTAAAGGAAAGTTCCGAGAAGGCATTGATAAGCCAGACCCTCCTACCTGGAAGACGCGTTTACGATGTGCTTTGAACAAAAGCAATGACTTTGAAGAACTGGTTGAGAGGAGCCAGCTGGACATCTCAGACCCCTACAAAGTATACAGGATTGTTCCTGAGGGAGCCAAAAAAGGAGCAAAGCAGATCACCCTGGAGGACTCACAGATGACCATGAGCCACCCCTACACCATGACAACTCCCTATACCTCACTCCCCGCTCAGCAGGTTCATAACTACATGATGCCATCCCACGACCGAAGCTGGAGGGAGTACGTCCCTGATCAGCCACACCCCGAAATCCCATATCAATGT---CCTGTGACATTTGGACCCCGAAGCCACCACTGGCAAGGCCCGGCTTGTGAAAATGGTTGCCAGGTGACAGGAACCTTTTATGCTTGTGCCCCATCTGAGTCCCAGGCCCCTGGAATCCCCATAGAGCCAAGCATAAGGTCTGCCGAAGCTTTGGCACTTTCAGACTGCCGGCTCCACATATGCTTATACTACCGGGAAATCCTGGTGAAAGAGTTGACCACGGCCAGCCCTGAAGGTTGTCGGATCTCCCATGGGCACACTTATGATGCCAGTAACCTGGACCAGGTCCTCTTCCCCTATCCAGAGGACAATGGCCAGAGGAAAAACATTGAGAAACTGCTGAGCCACCTAGAAAGGGGCGTGGTCCTCTGGATGGCTCCTGATGGACTTTATGCCAAAAGACTGTGCCAGAGCAGGATCTACTGGGATGGGCCCTTGGCACTGTGCAACGACCGGCCCAACAAACTAGAGAGAGACCAGACCTGCAAGCTCTTTGACACACAGCAGTTTTTATCAGAGCTGCAAGCATTTGCTCACCATGGCCGTTCCCTGCCAAGATTCCAGGTAACTCTCTGCTTTGGGGAAGAATTTCCAGATCCTCAGAGGCAAAGGAAGCTCATCACTGCTCACGTCGAACCTCTACTAGCCAGACAACTATATTATTTTGCTCAACAAAACAGTGGACATTTCCTAAGGGGCTATGATTTACCTGAACACATTAGCAGTCCAGAGGATTACCACAGATCTATTCGCCACTCCTCCATCCAAGAATGA

>MouseLemur_ENSMICG00000037072

------------------------------------------------------------------------------------------------------------------------------------------------------------------------------------------------------------------------------------------------------------------------------------------------------------------------------------------------------------------------------------------------------------------------------------------------------------------------------ATGAACCTGGACGGC---GGCGGCCGCGGCGGA---------------------GAGTTCGGCATGAGCGCGGTGAGCTGCGGCAACGGGAAGCTGCGCCAGTGGCTGATCGACCAGATCGACAGCGGCAAGTACCCGGGGCTGGTGTGGGAGAACGAGGAGAAGAGCATCTTCCGCATCCCCTGGAAGCACGCGGGCAAGCAGGACTACAACCGCGAGGAGGACGCCGCGCTCTTCAAGGCTTGGGCATTATTTAAAGGAAAGTTTCGAGAAGGCATCGATAAGCCAGACCCCCCTACCTGGAAGACGCGTCTAAGATGTGCTTTGAACAAGAGCAATGACTTTGAGGAATTAGTTGAGAGGAGCCAGCTGGACATCTCGGACCCGTACAAAGTGTACAGAATTGTTCCCGAGGGAGCCAAAAAAGGAGCAAAGCAGCTCACCCTGGAGGACCCACAGATGACCATGAATCACCCCTACCCCATGGCGGCTCCTTACCCCCCACTCCCGGCCCAGCAGGTTCATAACTACGTGATGCCACCCCTCGACCGAAGCTGGAGGGATTACGTCCCTGATCAACCACACCCTGAAATCCCGTACCAATGT---CCCGTGACGTTTGGACACCGTGGCCACCACTGGCAAGGCCCAGCTTGTGAAAATGGTTGCCAGGTGACAGGAACCTTTTATGCTTGTGCCCCACCTGAGTCCCAGGCTCCCGGAATCCCCACAGAGCCAAGCATAAGGTCTGCTGAAGCCTTGGCGCTCTCAGACTGCCGGCTGCACATCTGCCTGTACTACCGGGAAATCCTGGTGAAGGAGCTGACCACGTCCAGCCCGGAGGGCTGCCGGATCTCCCACGGACACACTTACGATGCCAGCAACCTGGACCAGGTGCTGTTCCCCTACCCTGAGGACAATGGCCAGAGGAAAAACATCGAGAAACTGCTGAGCCACCTGGAGAGGGGTGTGGTCCTGTGGATGGCCCCCGACGGGCTTTATGCCAAAAGACTGTGCCAGAGCAGGATCTACTGGGATGGGCCCCTGGCCCTGTGCAGCGACCGGCCCAACAAACTGGAGAGAGACCAGACCTGCAAGCTCTTTGACACCCAGCAGTTTTTGTCAGAGCTGCAAGCATTTGCCCACCACAGCCGCCCCCTGCCAAGATTCCAGGTGACGCTGTGCTTCGGGGAGGAGTTTCCAGACCCCCAGAGGCAACGGAAGCTCATCACGGCTCATGTGGAGCCTCTGCTCGCCAGACAACTGTATTATTTTGCTCAACAAAACAGTGGACATTTCCTGAGGGGCTATGACATGCCCGAACACATTAGCAGTCCGGAGGATTACCACAGATCTATCCGTCACTCCTCCATTCAAGAATGA

>MasNightMonkey_ENSANAG00000035286

------------------------------------------------------------------------------------------------------------------------------------------------------------------------------------------------------------------------------------------------------------------------------------------------------------------------------------------------------------------------------------------------------------------------------------------------------------------------------ATGAACTTGGAGGGC---GGCGGCCGAGGCGGA---------------------GAGTTCGGCATGAGCGCGGTGAGCTGCGGCAACGGGAAGCTCCGCCAGTGGCTGATCGACCAGATCGACAGCGGCAAGTACCCGGGGCTGGTGTGGGAGAACGAGGAGAAGAGCATCTTCCGCATCCCCTGGAAGCACGCGGGCAAGCAGGACTACAACCGCGAGGAGGACGCCGCGCTCTTCAAGGCTTGGGCACTATTTAAAGGAAAGTTCCGAGAAGGCATTGACAAGCCAGACCCTCCCACCTGGAAGACACGTTTACGATGTGCTCTCAACAAGAGCAATGACTTTGAGGAACTGGTTGAACGGAGCCAGCTGGACATCTCAGACCCATACAAAGTGTATAGGATTGTTCCTGAAGGAGCCAAAAAAGGAGCCAAGCAGCCCACTCTGGAGGACCCACAGATGTCCATGAGTCACCCCTATACCATGACAGCACCTTACCCCTCGCTTCCGGCCCAGCAGGTTCACAACTACGTGATGCCACCCCTTGACCGAAGCTGGAGAGACTATGTCCCCGATCAGCCGCACCCGGAAATCCCGTACCAATGT---CCGGTCACATTTGGACACCGCGGCCACCACTGGCAAGGCCCAGCTTGTGAAAATGGTTGCCAGGTGACAGGAACCTTTTATGCTTGTGCCCCACCTGAGTCCCAGGCTCCTGGAATCCCCACAGAGCCAAGCATAAGGTCTGCCGAAGCCTTGGCGCTCTCAGACTGCCGGCTGCACATCTGCCTGTACTACCGGGAAATCCTCGTGAAGGAGCTCACCACGTCCAGCCCCGAGGGCTGCCGGATCTCCCACGGACACACCTACGACCCCAGCAACCTGGACCAGGTCCTGTTCCCCTACCCAGAGGACAATGGCCAGAGGAAAAACATTGAGAAGCTGCTGAGCCACCTGGAGAGGGGCGTGGTCCTCTGGATGGCCCCCGACGGGCTCTATGCGAAAAGACTGTGCCAGAGCAGGATCTACTGGGACGGGCCCCTGGCGCTGTGCAACGACCGGCCCAACAAACTGGAGAGAGACCAGACCTGCAAGCTCTTTGACACGCAGCAGTTTTTGTCAGAGCTGCAAGCGTTTGCTCACCACGGCCGCTCCCTGCCAAGATTCCAGGTGACTCTGTGCTTTGGAGAGGAGTTTCCAGACCCTCAGAGGCAAAGAAAGCTCATCACGGCTCATGTAGAACCTCTGCTAGCCAGACAACTGTATTATTTTGCTCAGCAAAACAGTGGACATTTCCTGAGGGGCTACGATTTACCGGAACACATCAGCAGTCCAGAGGATTACCACAGATCAGTCCGCCATTCCTCCATTCAAGAATGA

>Marmoset_ENSCJAG00000019524

------------------------------------------------------------------------------------------------------------------------------------------------------------------------------------------------------------------------------------------------------------------------------------------------------------------------------------------------------------------------------------------------------------------------------------------------------------------------------ATGAACTTGGAGGGC---GGCGGCCGAGGCGGA---------------------GAGTTCGGCATGAGCGCGGTGAGCTGCGGCAACGGGAAGCTCCGCCAGTGGTTGATCGACCAGATCGACAGTGGCAAGTACCCGGGGCTAGTGTGGGAGAACGAGGAGAAGAGCATCTTCCGCATCCCCTGGAAGCACGCGGGCAAGCAGGACTACAACCGCGAGGAGGACGCCGCGCTCTTCAAGGCTTGGGCACTATTTAAAGGAAAGTTCCGAGAAGGCATCGACAAGCCAGACCCTCCCACCTGGAAGACACGTTTACGATGTGCTCTCAATAAGAGCAATGACTTTGAGGAACTGGTTGAGCGGAGCCAGCTGGACATCTCAGACCCGTACAAAGTGTACAGGATTGTTCCTGAAGGAGCCAAAAAAGGAGCCAAGCAGCTCACCCTAGAGGACCCACAGATGGCCATGAGTCACCCCTACACCATGACAGCGCCTTACCCCTCGCTTCCAGCCCAGCAGGTCCACAACTACGTGATGCCACCCCTTGACCGAAGCTGGAGAGACTATGTCCCTGATCAGCCGCACCCGGAAATCCCGTACCAATGT---CCCGTCACATTTGGTCACCGCGGCCACCACTGGCAAGGCCCAGCTTGTGAAAATGGTTGCCAGGTGACAGGAACCTTTTATGCTTGTGCCCCACCTGAGTCCCAAGCTCCTGGAATCCCCACAGAGCCAAGCATAAGGTCTGCTGAAGCCTTGGCGCTCTCAGACTGCCGGCTGCACATCTGCCTGTACTACCGGGAAATCCTCGTGAAAGAGCTCACCACGTCTAGCCCCGAGGGCTGCCGGATCTCCCACGGACACACCTACGACCCCAGCAACCTGGACCAGGTCCTGTTCCCCTACCCAGAGGACAATGGCCAGAGGAAAAACATTGAGAAGCTGCTGAGCCACCTGGAGAGGGGTGTGGTCCTCTGGATGGCCCCTGACGGGCTCTATGCGAAAAGACTGTGCCAGAGCAGGATCTACTGGGACGGGCCCCTGGCGCTGTGCAACGACCGGCCCAACAAATTGGAGAGAGACCAGACCTGCAAGCTCTTTGACACGCAGCAGTTTTTGTCAGAGCTGCAGGCATTTGCTCACCACGGCCGCTCCCTGCCAAGATTCCAGGTGACTCTGTGCTTTGGAGAGGAGTTTCCAGACCCTCAGAGGCAAAGAAAGCTCATCACAGCTCACGTAGAACCTCTGCTAGCCAGACAACTGTATTATTTTGCTCAACAAAACAGTGGACATTTCCTGAGGGGCTACGATTTACCGGAACACATCAGCAGTCCAGAGGATTACCACAGATCTATCCGCCATTCCTCCATTCAAGAATGA

>Macaca_ENSMMUG00000018513

------------------------------------------------------------------------------------------------------------------------------------------------------------------------------------------------------------------------------------------------------------------------------------------------------------------------------------------------------------------------------------------------------------------------------------------------------------------------------ATGAACCTGGAGGGC---AGCGGCCGAGGCGCA---------------------GAGTTCGGCATGAGCGCGGTGAGCTGCGGCAACGGGAAGCTCCGCCAGTGGCTGATCGACCAGATCGACAGCGGCAAGTACCCCGGGCTGGTGTGGGAGAACGAGGAGAAGAGCATCTTCCGCATCCCCTGGAAGCACGCGGGCAAGCAGGACTACAACCGCGAGGAGGACGCCGCGCTCTTCAAGGCTTGGGCGCTATTTAAAGGAAAGTTCCGAGAAGGCATCGACAAGCCGGACCCTCCTACCTGGAAGACTCGTTTACGATGTGCTCTGAACAAGAGCAATGACTTTGAGGAACTGGTTGAGCGGAGCCAGCTGGACATCTCGGACCCGTACAAAGTGTACAGGATTGTTCCTGAGGGAGCCAAAAAAGGAGCCAAGCAGCTCACCCTGGAGGACCCGCAGATGTCCATGAGCCACCCCTACACCATGACAACGCCTTACCCCTCGCTCCCAGCCCAGCAGGTTCACAACTACGTGATGCCATCCCTTGACCGAAGCTGGAGGGACTATGTCCCAGATCAGCCGCACCCGGAAATCCCGTACCAATGT---CCCGTAACGTTCGGACCCCGCGGCCACCACTGGCAAGGCCCAGCTTGTGAAAATGGTTGCCAGGTGACAGGAACCTTTTATGCTTGTGCCCCACCTGAGTCCCAGGCTCCAGGAATCCCCACAGAGCCAAGCATAAGGTCTGCCGAAGCCTTGGCGCTCTCAGACTGCCGGCTGCACATCTGCCTGTACTACCGGGAAATCCTCGTGAAGGAGCTGACCACGTCCAGCCCCGAGGGCTGCCGGATCTCCCATGGACATACTTACGACGCCAGCAACCTGGACCAGGTCCTGTTCCCCTACCCAGAGGACAATGGCCAGAGGAAGAACATTGAGAAGCTGCTGGGCCACCTGGAGAGGGGCGTGGTCCTCTGGATGGCCCCCGACGGGCTCTACGCGAAAAGACTCTGCCAGAGCCGGATCTACTGGGACGGGCCCCTGGCGCTGTGCAGCGACCGGCCCAACAAGCTGGAGAGAGACCAGACCTGCAAGCTCTTTGACACGCAGCAGTTTTTGTCAGAGTTGCAAGCGTTTGCTCACCACGGCCGCTCCCTGCCACGATTCCAGGTGACTCTGTGCTTCGGAGAGGAGTTTCCAGACCCTCAGAGGCAAAGGAAGCTCATCACAGCTCACGTAGAACCTCTGCTCGCCAGACAATTATATTATTTTGCTCAACAAAACAGTGGACATTTCCTGAGGGGCTACGATTTACCGGAACACATCAGCAATCCAGAAGATTACCACAGATCTATCCGCCATTCCTCCATTCAAGAATGA

>GoldenSnubNoseMonkey_ENSRROG00000035766

------------------------------------------------------------------------------------------------------------------------------------------------------------------------------------------------------------------------------------------------------------------------------------------------------------------------------------------------------------------------------------------------------------------------------------------------------------------------------ATGAACCTGGAGGGC---AGCGGCCGAGGCGGA---------------------GAGTTCGGCATGAGCGCGGTGAGCTGCGGCAACGGGAAGCTCCGCCAGTGGCTGATCGACCAGATCGACAGCGGCAAGTACCCCGGGCTGGTGTGGGAGAACGAGGAGAAGAGCATCTTCCGCATCCCCTGGAAGCACGCGGGCAAGCAGGACTACAACCGCGAGGAGGACGCCGCGCTCTTCAAGGCTTGGGCACTATTTAAAGGAAAGTTCCGAGAAGGCATCGACAAGCCAGACCCTCCTACCTGGAAGACTCGTTTACGATGCGCTCTGAACAAGAGCAATGACTTTGAGGAACTGGTCGAGCGGAGCCAGCTGGACATCTCGGACCCGTACAAAGTGTACAGGATTGTTCCTGAGGGAGCCAAAAAAGGAGCCAAGCAGCTC---ATGGAGGACCCGCAGATGTCCATGAGCCACCCCTACACCATGACAACGCCTTACCCCTCGCTCCCAGCCCAGCAGGTTCACAACTACGTGATGCCACCCCTTGACCGAAGCTGGAGGGACTATGTCCCAGATCAGCCGCACCCGGAAATCCCGTACCAATGT---CCCGTAACGTTCGGACCTCGCGGCCACCACTGGCAAGGCCCAGCTTGTGAAAATGGTTGCCAGGTGACAGGAACCTTTTATGCTTGTGCCCCACCTGAGTCCCAGGCTCCCGGCATCCCCACAGAGCCAAGCATAAGGTCTGCTGAAGCCTTGGCGCTCTCAGACTGCCGGCTGCACATCTGCCTGTACTACCGGGAAATCCTCGTGAAGGAGCTGACCACGTCCAGCCCCGAGGGCTGCCGAATCTCCCATGGACATTCCTACGACGCCAGCAACCTGGACCAGGTCCTGTTCCCCTACCCAGAGGACAACGGCCAGAGGAAAAACATTGAGAAGCTGCTGGGCCACCTGGAGAGGGGCGTGGTCCTCTGGATGGCCCCCGACGGGCTCTATGCAAAAAGACTCTGCCAGAGCCGGATCTACTGGGACGGGCCCCTGGCGCTGTGCAGCGACCGGCCCAACAAACTGGAGAGAGACCAGACCTGCAAGCTCTTTGACACACAGCAGTTTTTGTCAGAGTTGCAAGCGTTTGCTCACCACGGCCGCTCCCTGCCGAGATTCCAGGTGACTCTATGCTTCGGAGAGGAGTTTCCAGACCCTCAGAGGCAAAGGAAGCTCATCACAGCTCACGTAGAACCTCTGCTCGCCAGACAACTGTATTATTTCGCTCAGCAAAACAGTGGACATTTCCTGAGGGGCTACGATTTACCGGAGCACATCAGCAATCCGGAAGATTACCACAGATCTATCCGCCATTCCTCCATTCAAGAATGA

>Gibbon_ENSNLEG00000010631

------------------------------------------------------------------------------------------------------------------------------------------------------------------------------------------------------------------------------------------------------------------------------------------------------------------------------------------------------------------------------------------------------------------------------------------------------------------------------ATGAACCTGGAGGGC---AGCGGCCGAGGCGGA---------------------GAGTTCGGCATGAGCGCGGTGAGCTGCGGCAACGGGAAGCTGCGCCAGTGGCTGATCGACCAGATCGACAGCGGCAAGTACCCCGGGCTGGTGTGGGAGAACGAGGAGAAGAGCATCTTCCGCATCCCCTGGAAGCACGCGGGCAAGCAGGACTACAACCGCGAGGAGGACGCCGCGCTCTTCAAGGCTTGGGCACTGTTTAAAGGAAAGTTCCGAGAAGGCATCGACAAGCCAGACCCTCCTACCTGGAAGACACGTTTACGATGTGCTCTGAACAAGAGCAATGACTTCGAGGAACTGGTTGAGCGGAGCCAGCTGGACATCTCAGACCCGTACAAAGTGTACAGGATTGTTCCTGAGGGAGCCAAGAAAGGAGCCAAGCAGCTCACCCTGGAGGACCCGCAGATGTCCATGAGCCACCCCTACACCATGACAACGCCTTACCCCTCACTCCCAGCCCAGCAGGTTCACAACTACATGATGCCACCCCTCGACCGAAGCTGGAGGGACTACGTCCCGGATCAGCCACACCCGGAAATCCCGTACCAATGT---CCCGTAACGTTTGGACCCCGCGGCCACCACTGGCAAGGCCCAGCTTGTGAAAATGGTTGCCAGGTGACAGGAACCTTTTATGCTTGTGCCCCACCTGAGTCCCAGGCTCCCGGAATCCCCACAGAGCCAAGCATAAGGTCTGCCGAAGCCTTGGCGCTCTCAGACTGCCGGCTGCACATCTGCCTGTACTACCGGGAAATCCTCGTGAAGGAGCTGACCACGTCCAGCCCCGAGGGCTGCCGGATCGCCCATGGACATACCTATGAGGCCAGCAACCTGGACCAGGTCCTGTTCCCCTACCCAGAGGACAATGGCCAGAGGAAGAACATTGAGAAGCTGCTGAGCCACCTGGAGAGGGGCGTGGTCCTCTGGATGGCCCCCGACGGGCTCTATGCAAAAAGACTGTGCCAGAGCAGGATCTACTGGGATGGGCCCCTGGCGCTGTGCAGCGACCGACCCAACAAACTGGAGAGAGACCAGACCTGCAAGCTCTTTGACACACAGCAGTTTTTGTCAGAGCTGCAAGCGTTTGCTCACCACGGCCGCTCCCTGCCAAGATTCCAGGTGACTCTGTGCTTTGGAGAGGAGTTTCCAGACCCTCAGAGGCAAAGAAAGCTCATCACAGCTCACGTAGAACCTCTGCTAGCCAGACAACTATATTATTTTGCTCAACAAAACAGTGGACATTTCCTGAGGGGCTACGATTTAGCAGAACACATCAGCAATCCAGAAGATTACCACAGATCTATCCGCCATTCCTCCATTCAAGAATGA

>Gorilla_ENSGGOG00000012342

------------------------------------------------------------------------------------------------------------------------------------------------------------------------------------------------------------------------------------------------------------------------------------------------------------------------------------------------------------------------------------------------------------------------------------------------------------------------------ATGAACCTGGAGGGC---GGCGGCCGAGGCGGA---------------------GAGTTCGGCATGAGCGCGGTGAGCTGCGGCAACGGGAAGCTCCGCCAGTGGCTGATCGACCAGATCGACAGCGGCAAGTACCCCGGGCTGGTGTGGGAGAACGAGGAGAAGAGCATCTTCCGCATCCCCTGGAAGCACGCGGGCAAGCAGGACTACAACCGCGAGGAGGACGCCGCGCTCTTCAAGGCTTGGGCACTGTTTAAAGGAAAGTTCCGAGAAGGCATCGACAAGCCGGACCCTCCCACCTGGAAGACGCGCCTGCGGTGCGCTTTGAACAAGAGCAATGACTTTGAGGAACTGGTTGAGCGGAGCCAGCTGGACATCTCAGACCCGTACAAAGTGTACAGGATTGTTCCTGAGGGAGCCAAAAAAGGAGCCAAGCAGCTCACCCTGGAGGACCCGCAGATGTCCATGAGCCACCCCTACACCATGACAACGCCTTACCCTTCACTCCCAGCCCAGCAGGTTCACAACTACATGATGCCACCCCTCGACCGAAGCTGGAGGGACTACGTCCCGGATCAGCCACACCCGGAAATCCCGTACCAATGT---CCCGTGACGTTTGGACCCCGCGGCCACCACTGGCAAGGCCCAGCTTGTGAAAATGGTTGCCAGGTGACAGGAACCTTTTATGCTTGTGCCCCACCTGAGTCCCAGGCTCCCGGAATCCCCACAGAGCCAAGCATAAGGTCTGCCGAAGCCTTGGCGCTCTCAGACTGCCGGCTGCACATCTGCCTGTACTACCGGGAAATCCTCGTGAAGGAGCTGACCACGTCCAGCCCCGAGGGCTGCCGGATCTCCCATGGACATACGTATGATGCCAGCAACCTGGACCAGGTCCTGTTCCCCTACCCAGAGGACAATGGCCAGAGGAAAAACATTGAGAAGCTGCTGAGCCACCTGGAGAGGGGCGTGGTCCTCTGGATGGCCCCCGATGGGCTCTATGCGAAAAGACTGTGCCAGAGCAGGATCTACTGGGACGGGCCCCTGGCGCTGTGCAACGACCGGCCCAACAAACTGGAGAGAGACCAGACCTGCAAGCTCTTTGACACACAGCAGTTCTTATCAGAGCTGCAAGCATTTGCTCACCACGGCCGCTCCCTGCCAAGATTCCAGGTGACTCTATGCTTTGGAGAGGAGTTTCCAGACCCTCAGAGGCAAAGAAAGCTCATCACAGCTCACGTAGAACCTCTGCTAGCCAGACAACTATATTATTTTGCTCAACAAAACAGTGGACATTTCCTGAGGGGCTACGATTTACCAGAACACATCAGCAATCCAGAAGATTACCACAGATCTATCCGCCATTCCTCTATTCAAGAATGA

>Human_ENSG00000137265

------------------------------------------------------------------------------------------------------------------------------------------------------------------------------------------------------------------------------------------------------------------------------------------------------------------------------------------------------------------------------------------------------------------------------------------------------------------------------ATGAACCTGGAGGGC---GGCGGCCGAGGCGGA---------------------GAGTTCGGCATGAGCGCGGTGAGCTGCGGCAACGGGAAGCTCCGCCAGTGGCTGATCGACCAGATCGACAGCGGCAAGTACCCCGGGCTGGTGTGGGAGAACGAGGAGAAGAGCATCTTCCGCATCCCCTGGAAGCACGCGGGCAAGCAGGACTACAACCGCGAGGAGGACGCCGCGCTCTTCAAGGCTTGGGCACTGTTTAAAGGAAAGTTCCGAGAAGGCATCGACAAGCCGGACCCTCCCACCTGGAAGACGCGCCTGCGGTGCGCTTTGAACAAGAGCAATGACTTTGAGGAACTGGTTGAGCGGAGCCAGCTGGACATCTCAGACCCGTACAAAGTGTACAGGATTGTTCCTGAGGGAGCCAAAAAAGGAGCCAAGCAGCTCACCCTGGAGGACCCGCAGATGTCCATGAGCCACCCCTACACCATGACAACGCCTTACCCTTCGCTCCCAGCCCAGCAGGTTCACAACTACATGATGCCACCCCTCGACCGAAGCTGGAGGGACTACGTCCCGGATCAGCCACACCCGGAAATCCCGTACCAATGT---CCCATGACGTTTGGACCCCGCGGCCACCACTGGCAAGGCCCAGCTTGTGAAAATGGTTGCCAGGTGACAGGAACCTTTTATGCTTGTGCCCCACCTGAGTCCCAGGCTCCCGGAGTCCCCACAGAGCCAAGCATAAGGTCTGCCGAAGCCTTGGCGTTCTCAGACTGCCGGCTGCACATCTGCCTGTACTACCGGGAAATCCTCGTGAAGGAGCTGACCACGTCCAGCCCCGAGGGCTGCCGGATCTCCCATGGACATACGTATGACGCCAGCAACCTGGACCAGGTCCTGTTCCCCTACCCAGAGGACAATGGCCAGAGGAAAAACATTGAGAAGCTGCTGAGCCACCTGGAGAGGGGCGTGGTCCTCTGGATGGCCCCCGACGGGCTCTATGCGAAAAGACTGTGCCAGAGCAGGATCTACTGGGACGGGCCCCTGGCGCTGTGCAACGACCGGCCCAACAAACTGGAGAGAGACCAGACCTGCAAGCTCTTTGACACACAGCAGTTCTTGTCAGAGCTGCAAGCGTTTGCTCACCACGGCCGCTCCCTGCCAAGATTCCAGGTGACTCTATGCTTTGGAGAGGAGTTTCCAGACCCTCAGAGGCAAAGAAAGCTCATCACAGCTCACGTAGAACCTCTGCTAGCCAGACAACTATATTATTTTGCTCAACAAAACAGTGGACATTTCCTGAGGGGCTACGATTTACCAGAACACATCAGCAATCCAGAAGATTACCACAGATCTATCCGCCATTCCTCTATTCAAGAATGA

>Bonobo_ENSPPAG00000040992

---------------------------------------------------------------------------------------------------------------------------------------------------------------------------------------------------------------------------------------------------------------------------------------------------------------------------------------------------------------------------------------------------------------------------------------------------------------------------------------------------------------------------------------------------------------------------------------------------------------------------------------------------------------------------------------CAGGACTACAACCGCGAGGAGGACGCCGCGCTCTTCAAGGCTTGGGCACTGTTTAAAGGAAAGTTCCGAGAAGGCATCGACAAGCCGGACCCTCCCACCTGGAAGACGCGCCTGCGGTGCGCTTTGAACAAGAGCAATGACTTTGAGGAACTGGTTGAGCGGAGCCAGCTGGACATCTCAGACCCGTACAAAGTGTACAGGATTGTTCCTGAGGGAGCCAAAAAAGGAGCCAAGCAGCTCACCCTGGAGGACCCGCAGATGTCCATGAGCCACCCCTACACCATGACAACGCCTTACCCTTCGCTCCCAGCCCAGCAGGTTCACAACTACATGATGCCACCCCTCGACCGAAGCTGGAGGGACTACGTCCCGGATCAGCCACACCCGGAAATCCCGTACCAATGT---CCCGTGACGTTTGGACCCCGCGGCCACCACTGGCAAGGCCCAGCTTGTGAAAATGGTTGCCAGGTGACAGGAACCTTTTATGCTTGTGCCCCACCTGAGTCCCAGGCTCCCGGAGTCCCCACAGAGCCAAGCATAAGGTCTGCCGAAGCCTTGGCGTTCTCAGACTGCCGGCTGCACATCTGCCTGTACTACCGGGAAATCCTCGTGAAGGAGCTGACCACGTCCAGCCCCGAGGGCTGCCGGATCTCCCATGGACATACGTATGACGCCAGCAACCTGGACCAGGTCCTGTTCCCCTACCCAGAGGACAATGGCCAGAGGAAAAACATTGAGAAGCTGCTGAGCCACCTGGAGAGGGGCGTGGTCCTCTGGATGGCCCCCGACGGGCTCTATGCGAAAAGACTGTGCCAGAGCAGGATCTACTGGGACGGGCCCCTGGCGCTGTGCAACGACCGGCCCAACAAACTGGAGAGAGACCAGACCTGCAAGCTCTTTGACACACAGCAGTTCTTGTCAGAGCTGCAAGCGTTTGCTCACCACGGCCGCTCCCTGCCAAGATTCCAGGTGACTCTATGCTTTGGAGAGGAGTTTCCAGACCCTCAGAGGCAAAGAAAGCTCATCACAGCTCATGTAGAACCTCTGCTAGCCAGACAACTATATTATTTTGCTCAACAAAACAGTGGACATTTCCTGAGGGGCTACGATTTACCAGAACACATCAGCAATCCAGAAGATTACCACAGATCTATCCGCCATTCCTCTATTCAAGAATGA

>Chimpanzee_ENSPTRG00000017656

------------------------------------------------------------------------------------------------------------------------------------------------------------------------------------------------------------------------------------------------------------------------------------------------------------------------------------------------------------------------------------------------------------------------------------------------------------------------------ATGAACCTGGAGGGC---GGCGGCCGAGGCGGA---------------------GAGTTCGGCATGAGCGCGGTGAGCTGCGGCAACGGGAAGCTCCGCCAGTGGCTGATCGACCAGATCGACAGCGGCAAGTACCCCGGGCTGGTGTGGGAGAACGAGGAGAAGAGCATCTTCCGCATCCCCTGGAAGCACGCGGGCAAGCAGGACTACAACCGCGAGGAGGACGCCGCGCTCTTCAAGGCTTGGGCACTGTTTAAAGGAAAGTTCCGAGAAGGCATCGACAAGCCGGACCCTCCCACCTGGAAGACGCGCCTGCGGTGCGCTTTGAACAAGAGCAATGACTTTGAGGAACTGGTTGAGCGGAGCCAGCTGGACATCTCAGACCCATACAAAGTGTACAGGATTGTTCCTGAGGGAGCCAAAAAAGGAGCCAAGCAGCTCACCCTGGAGGACCCGCAGATGTCCATGAGCCACCCCTACACCATGACAACGCCTTACCCTTCGCTCCCAGCCCAGCAGGTTCACAACTACATGATGCCACCCCTCGACCGAAGCTGGAGGGACTATGTCCCGGATCAGCCACACCCGGAAATCCCGTACCAATGT---CCCGTGACGTTTGGACCCCGCGGCCACCACTGGCAAGGCCCAGCTTGTGAAAATGGTTGCCAGGTGACAGGAACCTTTTATGCTTGTGCCCCACCTGAGTCCCAGGCTCCCGGAGTCCCCACAGAGCCAAGCATAAGGTCTGCCGAAGCCTTGGCGTTCTCAGACTGCCGGCTGCACATCTGCCTGTACTACCGGGAAATCCTCGTGAAGGAGCTGACCACGTCCAGCCCCGAGGGCTGCCGGATCTCCCATGGACATACGTATGATGCCAGCAACCTGGACCAGGTCCTGTTCCCCTACCCAGAGGACAATGGCCAGAGGAAAAACATTGAGAAGCTGCTGAGCCACCTGGAGAGGGGCGTGGTCCTCTGGATGGCCCCCGACGGGCTCTATGCGAAAAGACTGTGCCAGAGCAGGATCTACTGGGACGGGCCCCTGGCGCTGTGCAACGACCGGCCCAACAAACTGGAGAGAGACCAGACCTGCAAGCTCTTTGACACACAGCAGTTCTTGTCAGAGCTGCAAGCGTTTGCTCACCACGGCCGCTCCCTGCCAAGATTCCAGGTGACTCTATGCTTTGGAGAGGAGTTTCCAGACCCTCAGAGGCAAAGAAAGCTCATCACAGCTCATGTAGAACCTCTGCTAGCCAGACAACTATATTATTTTGCTCAACAAAACAGTGGACATTTCCTGAGGGGCTACGATTTACCAGAACACATCAGCAATCCAGAAGATTACCACAGATCTATCCGCCATTCCTCTATTCAAGAATGA

# IRF5

>Horse_ENSECAG00000009902

ATGGCCTGTGTTTGCACCGATCCTGCTCCCAGAGGGCCCGGGCTGGGGCAGCTCCCGGGCCCCGCCAAGCACTACGGGGAAGTGGGGGGCTGGGGTTGTCGAATGACTATTTTGCAGTTCCAGATTGCCGAAGGAACCCAGTCGTCGGTCCAGGCCATCACGAGGCCGGGGGTCTCCAGAGCCCCGGAGGCTCTGGGCTGCAGGGGTGCCCGACCTGGCCCGACGCTCAACCGGGGCCCACGGTCGCCAGATCAATGCGGGGGCCCCAGGGTGGCCCCGCGGGGCAGGGCGGGGCGCCGTCCGCTCATCCAGGCTCTGCGGCCTCGGCCCGGGGGCGGGGGCGAGGGCGAGGGCGGGGGCGGGCGACGCGGAAGTGCCCGGCGTGCGGACCGGCTGGAGGCGCAACTTCGGGAGAGCGCAGCTCGGCCCCGCCGCCCGGCCCGTGCTCCTGGGCGCAGCCACGCGGGCGCACCGCAGACAGACCCCTTTGCCATGAACCAGCCTGCTCCCACTGCCGCCCCTCCACCCCGCCGCGTGCGGCTGAAGCCCTGGCTGGTGGCCCAGGTGAACAGCTGCCAGTACCCAGGGCTTCAGTGGGTCAACGGGGAGAGGAAACTCTTCTACATCCCCTGGCGCCATGCCACACGGCATGGACCCAGCCAGGATGGAGATAACACCATCTTCAAGGCCTGGGCCAAGGAGACGGGGAAGTACACTGAGGGGGTGGACGAGGCCGATCCGGCCAAGTGGAAGGCCAACCTGCGCTGTGCCCTTAACAAGAGCCGGGACTTCCGCCTCATCTATGATGGGCCCCGGGACATGCCGCCTCAGCCCTACAAGATCTACGAGGTCTGCTCCAATGGCCCCGCTCCCGCAGAGTCACAGCCCAGTGAGGATTATGCCCCCATTGGTACAGGAGAGGAGGAGGAGGAGGAAGAGGAAGAG---------------------------------------------CTCCAGAGGATGTTACCAAGCCTGAGCCTCACAGAACCTGTGCAGCCCCGCCCCCCCATGCCACCCTACTCTTTACCCAAAGAGGATGTCAAGTGG---------------------------------------------CCACCCACTCTCCAGCCACCTGTGGTGATTGGCCCGCTTGCTCCAGATCCCAGCCTCCTGGGCCCTGCCCCTGGCAATGCAGCTGGCTTTGGGGAC------CTTCTCCCGGAGGTCCTGCCGAGC------CTGCAGCCTGGGGGGCCTCTGGTTGCCAGCCTACCCCCCACGGGCGAACAACTCCTGCCC------------------------------------------GACCTGCTGATC---------AGCCCCCACATGCTGCCT---TTGACTGACCTGGAGATCAAGTTCCAGTACCGGGGGCGGCCACCCCGGGCCCTCACCATCAGCAACCCACATGGCTGCCGGCTCTTCTACAGCCAGCTAGAGGCCACTCAGGAGCAGGTGGAGCTCTTCGGTCCTGTGAGCCTGGAGCAAGTACGCTTCCCCAGCCCTGAGGACATCCCCAGCGACAAGCAGCGCTTCTACACAAACCAGCTGCTGGATGTCCTGGACCGCGGACTCATCCTCCAGCTACAAGGCCAGGATCTGTATGCCATCCGCCTGTGCCAGTGCAAGGTGTTCTGGAGTGGGCCCTGTGCCTCAGCCCACGGCTCACACCCCAACCCCATCCAGCGGGAGGTCAAGACCAAGCTTTTCAGCCTGGAGCATTTTCTCAACGAGCTCATCCTGTTCCAGAAGGGCCAGACCAACACCCCACCACCATTCGAGATCTTCTTCTGCTTTGGGGAGGAGTGGCCTGACCGCAAACCCCGAGAGAAGAAGCTCATCACAGTACAG---------------------------------TTCTTTTCCAATTCTGAGTGTGCTGTGGGAACCAGGGACGAAACATCAATGGAGAGCCAACAGGCAGAGGCCACCCACTCCAAACCAACCCGAAAATTTTCACCT------CAGTCTGGTTCAGTATTAGAGTTGGAGGGAGAACTTACCTGTGTGACTAGTGGTTGCAGCAAGGCTGCAGATCCTTTGCCTACACAGCGAACAGCAAAGCGGAGGCACCTGCAACAACGCTCTACAATCCGATTATCAGCCCGGTGCTTATTTAGAGTTTCAGATAAAACTGGCCATATCTGA

>Rabbit_ENSOCUG00000010101

------------------------------------------------------------------------------------------------------------------------------------------------------------------------------------------------------------------------------------------------------------------------------------------------------------------------------------------------------------------------------------------------------------------------------------------------------------------------------------------------------------ATGAACCAGCCGGCCCCCGGGGCTCCCCCGCCGCCCCGCCGCGTGAGGCTCAAGCCCTGGCTGGTGGCCCAGGTGAACAGCTGCCAGTACCCGGGGCTTCAGTGGGTCAATGGGGAGAGGAGGTTCTTCTACATCCCCTGGCGCCATGCCACTCGGCACGGTCCCAGCCAGGATGGAGACAACACCATCTTCAAGGCCTGGGCCAAGGAGACGGGCAAGTACACCGAGGGTGTGGACGAGGCTGATCCGGCCAAGTGGAAAGCCAACCTGCGCTGTGCCCTCAACAAGAGCCGCGACTTCCGCCTCATCTATGACGGGCCCCGGGACATGCCGCCTCAGCCCTACAAGATCTACGAGGTCTGCTCCAACGGCCCCACTGCCACAGAGTCGCAGCCCGCTGATGAGTACTAT---GTGGGTCCAGGAGAGGAAGAGGAGGAGGAAGATGAAGAG---------------------------------------------CTCCAGAGGATGCTACCAGGCCTGTGCATCACAGACCCAGTGCCTTCCGGCCCCGCCCCGCCACCCTATTCCTTCCCCAAAGAGGACATCAAGTGG---------------------------------------------CCCCCTGCTGTGCAGCCACCTGTGCTACCGGGCCCCCTCCCTCCAGACGACGGCCTCCTGGTCCCTCCCTCGGGTACCACTGCTGGCTTTGAGGAGCTGCAGCTTCTTCCTGAGGTCCTGCAAAGC------GTGCAGCCT---GGG------------------CCCCCTGCAGGCGAACAGTTCCTGCCC------------------------------------------GACCTACTCGTC---------AACCCCCACATGCTGCCC---CTGACGGACCTGGAGATCAAGTTCCAGTACCGGGGGCGACCGCCCCGCGCCCTCACCATCAGCAACCCGCACGGCTGCCGGCTCTTCTACAGCCAGCTGGAGGCCACCCAGGAGCAGGTGGAGCTGTTCGGCCCCGTGAGCCTGGAGCAAGTGCGCTTCCCCAGCCCCGAGGACATCCCCAGTGACAAGCAGCGCTTCTACACGAACCAGCTGCTGGACGTCCTGGACCGCGGGCTCATCCTGCAGCTCCAGGGCCAGGACCTGTACGCCATCCGCCTGTGCCAGTGCAAGGTGTTCTGGAGCGGGCCCTGCGCCTCAGCCCACGGCTCGCGCCCCAACCCCATCCAGCGGGAGGTCAAGACCAAGCTCTTCAGCCTGGAGTACTTTCTTAATGAGCTCATCCTGTTCCAGAAGGGCCAGACCCACGCCCCTCCACCCTTCGAGATCTTCTTCTGCTTCGGGGAGGAGTGGCCTGACCGCAAGCCCCGAGAGAAGAAACTCATCACCGTCCAGGTGGTGCCGGTTGCAGCGCGCCTGCTGTTGGAGATGTTCTCGGGGGAGCTGTCTTGGTCGGCGGACAGCATCCGGCTACAGATCTCGAACCCGGACCTCAAAGACCGCATGGTGGAGCAGTTCAAGGAGCTCCATCACATCTGGCAGTCCCAGCAGCGCTTGCAGCCCGTGCCCCAGGCCCCTGCTGTGGCCGCGGGCCTTGGTGCTGGCCAGGCGCCCTGGCCCATGCATCCCGTCGGCATGCAGTGA------------------------------------------------------------------------------

>Mouse_ENSMUSG00000029771

------------------------------------------------------------------------------------------------------------------------------------------------------------------------------------------------------------------------------------------------------------------------------------------------------------------------------------------------------------------------------------------------------------------------------------------------------------------------------------------------------------ATGAACCACTCAGCCCCAGGGATTCCCCCACCACCCCGCCGTGTGAGGCTGAAGCCCTGGTTGGTGGCCCAGGTGAACAGCTGCCAGTACCCAGGGCTTCAGTGGGTCAACGGGGAAAAGAAACTCTTCTATATACCCTGGCGCCATGCCACGAGGCATGGTCCCAGCCAGGATGGGGACAACACCATCTTCAAGGCCTGGGCTAAAGAGACAGGGAAGTACACTGAAGGGGTGGATGAGGCTGACCCAGCCAAGTGGAAGGCCAACCTGCGCTGTGCCCTTAACAAAAGCCGTGACTTCCAGCTGTTCTATGATGGCCCTCGGGACATGCCACCTCAGCCGTACAAGATCTACGAGGTCTGCTCCAACGGCCCTGCTCCCACAGAGAGCCAACCCACTGATGATTACGTT---------CTGGGAGAAGAGGAGGAGGAGGAAGAGGAAGAG---------------------------------------------CTCCAGAGAATGCTACCAGGCCTGAGCATCACAGAGCCTGCGCTACCTGGGCCTCCCAACGCACCCTATTCCTTACCCAAAGAAGACACCAAGTGG---------------------------------------------CCACCTGCTCTCCAGCCACCTGTAGGGCTGGGTCCCCCTGTCCCAGACCCAAATCTCCTGGCCCCTCCCTCTGGAAATCCTGCTGGCTTCAGGCAG------CTTCTCCCTGAGGTC---------------CTGGAGCCT---GGACCTCTGGCTTCCAGCCAGCCCCCTACA---GAACCACTCTTGCCT------------------------------------------GACCTGCTGATC---------AGCCCCCACATGTTGCCT---TTGACGGACCTAGAGATCAAGTTCCAGTACCGGGGACGCGCACCCCGGACCCTCACCATCAGCAACCCACAAGGCTGCAGGCTCTTCTACAGCCAGCTAGAGGCTACCCAGGAGCAAGTGGAACTCTTTGGCCCTGTGACCCTGGAGCAAGTGCGCTTCCCTAGCCCAGAGGACATCCCCAGTGACAAGCAGCGTTTCTATACGAACCAGCTGCTAGATGTCCTGGACCGTGGGCTCATCCTGCAGCTGCAGGGCCAGGACCTGTACGCCATCCGTCTGTGCCAGTGTAAGGTGTTCTGGAGTGGGCCCTGCGCCTTGGCCCATGGCTCCTGCCCCAACCCCATTCAGCGGGAAGTCAAGACGAAGCTCTTTAGCCTAGAGCAGTTTCTCAATGAGCTCATCCTGTTCCAGAAGGGCCAGACTAATACCCCACCACCTTTTGAGATCTTCTTTTGCTTTGGAGAAGAATGGCCTGATGTCAAACCCCGAGAGAAGAAGCTCATTACTGTACAGGTGGTACCTGTTGCAGCCCGGTTGCTGCTGGAGATGTTCTCAGGGGAGCTTTCTTGGTCGGCAGACAGCATCCGACTGCAGATCTCAAACCCGGATCTCAAAGACCACATGGTAGAGCAGTTTAAAGAGCTTCATCACCTCTGGCAGTCCCAGCAGCAATTGCAGCCCATGGTCCAGGCCCCTCCT---GTGGCAGGCCTCGATGCAAGCCAGGGGCCCTGGCCCATGCACCCAGTTGGCATGCAATAA------------------------------------------------------------------------------

>Rat_ENSRNOG00000007437

---------------------------------------------------------------------------------------------------------------------------------------------------------------------------------------------------------------------------------------------------------------------------------------------------------------------------------------------------------------------------------------------------ATGGACTGGGGAGCTCGGTCACACAGCCTCTCCACCCAGGGCACGGGGAATCTTGCTATCAATTTGCAGAGCTGTGCTTTGCTTTCTCCCACAGAGCCCTTTGCCATGAACCAGTCAGCCCCAGGGATTCCCACACCACCCCGCCGTGTGAGGCTGAAGCCCTGGCTGGTGGCCCAGGTGAACAGCTGCCAGTACCCAGGGCTCCAGTGGGTCAACGGGGAAAAGAAACTCTTCTATATCCCCTGGCGCCATGCTACAAGGCACGGTCCCAGCCAGGATGGAGACAACACCATCTTCAAGGCCTGGGCTAAAGAGACAGGGAAGTACACGGAGGGCGTGGATGAGGCTGACCCAGCCAAATGGAAGGCCAACCTGCGCTGTGCCCTTAACAAAAGCCGTGACTTCCAGCTGTACTATGATGGCCCTCGAGACATGCCACCTCAGCCCTACAAGATCTACGAGGTCTGCTCCAATGGCCCTGCTCCCACAGAGTCCCAGCCCACAGATGATTACGTT---------CTGGGAGAAGAGGAGGAAGAGGAAGATGAAGAGGCTTCCCTTCAG---------------------------------CTCCAGAGAATGCTACCAGGCCTGAGTATCACAGAACCTGTGCTACCTGGGCCTCCCATAGCACCCTATTCCTTACCCAAAGAAGACACCAAGTGG---------------------------------------------CCACCTGCTCTCCAGCCACCTGTAGGGCTGGGTCCCCCTCCCCCAGACCCAAATCTCCTGGCCCCTCCCTCTGGAGATTCTGCTGGCTTTAGGCAG------CTTCTCCCTGAGGTC---------------CTGGAGCCT---GGACCCCTGGCTTCCAACCAGCCCCCTACA---GAACAACTCTTGCCT------------------------------------------GACCTGCTCATC---------AGCCCCCACATGCTGCCT---TTGACTGACCTAGAGATCAAGTTCCAGTACCGGGGACGTCCACCGCGGGCCCTCACCATCAGCAACCCACAAGGCTGCAGGCTCTTCTATAGCCAGCTAGAGGCTACCCAGGAGCAAGTGGAACTCTTTGGCCCTGTGACCCTGGAGCAAGTTCGCTTCCCTAGCCCAGAGGATATCCCCAGTGACAAGCAGCGCTTCTATACGAACCAGCTGCTGGATGTCCTGGACCGAGGGCTCATCCTGCAGCTGCAGGGCCAGGACCTGTATGCCATCCGTCTGTGCCAGTGTAAGGTGTTCTGGAGTGGGCCCTGTGCCTTGGCCCATGGTTCCTGCCCCAACCCCATCCAGCGGGAAGTCAAGACAAAGCTCTTTAGCCTAGAGCAGTTTCTCAATGAACTCATCCTGTTCCAGAAGGGCCAAACCAATACCCCACCACCTTTTGAGATCTTCTTTTGCTTTGGAGAAGAATGGCCTGACCTCAAACCCCGAGAGAAGAAGCTCATTACTGTACAGGTGGTACCTGTCGCAGCCCGGTTGCTGCTGGAGATGTTCTCAGGAGAGCTTTCTTGGTCAGCAGACAGCATTCGGCTGCAGATCTCAAACCCGGATCTCAAAGACCACATGGTAGAGCAGTTCAAAGAGCTCCATCACATCTGGCAGTCCCAGCAGCGCTTGCAGCCCATGGTCCAGGCCCCTCCT---GTGGCAGGCCTTGATGCTGGCCAGGGGCCCTGGCCCATGCACCCAGTTGGCATGCAATAG------------------------------------------------------------------------------

>Elephant_ENSLAFG00000021568

------------------------------------------------------------------------------------------------------------------------------------------------------------------------------------------------------------------------------------------------------------------------------------------------------------------------------------------------------------------------------------------------------------------------------------------------------------------------------------------------------------ATGAACCAGCCTGCCCCCGGGGCGCCCCCCGCACCTCGCCGGGTGCGGCTGAAGCCCTGGCTGGTGGCCCAGGTGAACAGCTGCCAGTACCCAGGGCTCCAGTGGGTCAACGGGGAGAGGAGATTCTTCTACATCCCCTGGCGCCACGCCACGCGACATGGCCCCAGCCAGGATGGGGACAACACCATCTTCAAGGCCTGGGCCAAGGAGACTGGGAAATACACTGAGGGTGTGGATGAGGCTGACCCGGCCAAGTGGAAGGCCAACCTGCGCTGTGCCCTTAACAAGAGCCGAGACTTCCGGCTCATCTACGATGGGCCCCGGGACATGCCACCTCAGCCCTACAAGATCTACGAGGTCTGCTCCCATGGTCCCGCGCCTACAGAAGCACAGCCCAGTGAGGACTATGCT---ATTGGGCCAGGAGAGGAGGAAGAGGAGGATGAGGAAGAG---------------------------------------------CTCCAGAGGATGTTACCAAGCCTGAGTCTCACAGAAGCAGTGCAGCCTGGCCCCTCCATGGGACCCTATTCTGTACCCAAAGAAGAGCTCAAGTGG---------------------------------------------CCCCCCACCCTCCAGGCGCCTGTGGTGCTGGGTCCCCCTGCTCCAGAGCCCACCCTCCTGGCCCCTCCCCCCAACAACCCTGCTAGCTTTGGGGAG------CTGCTCCCTGAAGTCCTG------------CTGGAGCCT---GGGCCCCTGGCCGTCAGCTTGCCCCCTGTAGGAGAACAACTCCTGCCT------------------------------------------GACCTGCTGATC---------AGCCCCCACATGCTGCCC---TTGACCGACCTGGAGATCAAGTTCCAGTACCGCGGCCGGCCGCCCCGCTCCCTAACCATCAGCAACCCGCATGGCTGCCGGCTCTTCTACAGCCAGCTGGAGGCCACCCAGGAGCAGGTGGAGCTCTTTGGCCCGGTGAGCCTGGAGCAGGTGCGCTTCCCCAGCCCTGAGGACATCCCCAGCGAGAAGCAGCGCTTCTACACCAATCAGCTGCTGGACGTGCTGGACCGCGGGCTCATCCTGCAGCTGCAGGGCCAGGACCTCTATGCCGTCCGCCTGTGCCAGTGCAAAGTGTTCTGGAGTGGGCCCTGTGCCGAGGCCCACGCTGCGTGCCCCAACCCCATCCAGCGGGAGGTGAAGACCAAGCTCTTTAGCCTCGAGCAGTTTCTCCACGAGCTCATTCTGTTCCAGAAGGGCCAGACCAACACCCCACCACCGTTTGAGATCTTCTTCTGCTTTGGGGAGGAGTGGCCCGACCGCAAACCCCGCGAGAAGAAGCTCATCACTGTACAGGTGGTGCCTGTGGCAGCTCGGTTGCTGCTGGAGATGTTCTCAGGGGAGCTTTCCTGGTCGGCTGACAGTATCCGGCTTCAGATCTCAAACCCAGACCTCAAGGACCGCATGGTGGAGCAGTTTAAGGAGCTCCATCACCTCTGGCAGTCCCAGCAGCGGGTGCACCCCGTGGCTCAGGCCCCTCCT---GGGCCAGGCCTCGGGGCTGACCAGGGGCCCTGGGCCATGCACCCAGTTGGCATGCAGTAA------------------------------------------------------------------------------

>Cow_ENSBTAG00000004989

------------------------------------------------------------------------------------------------------------------------------------------------------------------------------------------------------------------------------------------------------------------------------------------------------------------------------------------------------------------------------------------------------------------------------------------------------------------------------------------------------------ATGAACCAGCCGGCCCCCGCCGCCCTGCTTCCGCCCCGGCGCGTGCGGCTGAAGCCCTGGCTGGTGGCCCAGGTCAACAGCTGCCAGTACCCAGGGCTTCAGTGGGTCAACGGGGAAAAGAAATTGTTTTACATCCCCTGGCGCCATGCCACGCGGCACGGCCCTAGCCATGATGGAGATAACACCATCTTCAAGGCCTGGGCCAAGGAGACGGGGAAGTACACCGAGGGGGTGGACGAGGCCGATCCGGCCAAGTGGAAGGCCAACCTGCGCTGTGCCCTGAACAAGAGCCGTGACTTCCGCCTCATCTATGATGGGCCCCGGGACATGCCACCTCAGCCCTACAAGGTCTACGAGGTCTGCTCCAATGGCCCGGCTCCCGCAGAGTCACAGCCCAGTGAGGATAACGCT---------------------GAGGAAGAAGAAGAGGAAGAG---------------------------------------------CTCCAGAAGATGTTACCAGGCCTGAGCATCACAGAAGCAGTGCAGCCCGGCCCTGCCATGGCACCCTATTCTTTACCCAAAGAGGATGTCAAGTGG---------------------------------------------CCACCCACTCTCCAGCCACCTGTGGTGTTGGCCCCCCCTGCTCCAGGCCCCAACCTGCTGGTCCCTGCTCCTGGCAATGCCGCTGACTTTGGGGAG------GTGTTTTCTGAGGTTCTGCCGAGCTCGCAACCGCAGCCT---GGGTCCCTGTCTACCAGCCTGGCCCCCACAGGCGAACAACTCCTGCCC------------------------------------------GACCTGTTGATC---------AGCCCCCACATGCTGCCT---CTGACCGACCTGGAGATCAAGTTCCAGTACCGGGGACGGCCACCCCGGGCTCTCACCATCAGCAACCCTCAAGGCTGCCGGCTCTTCTACAGCCAACTGGAAGCCACCCAGGAGCAGGTGGAGCTCTTTGGCCCGGTGAGCCTGGAGCAAGTGCGCTTCCCCAGCCCCGAGGACATCCCCAGCGAGAAGCAACGCTTTTATACCAACCAGCTGCTGGACGTCCTGGACCGTGGGCTCATCCTTCAGCTACAAGGCCAGGATCTGTATGCCATCCGCCTGTGCCAGTGCAAGGTGTTCTGGAGCGGGCCCTGCGCCTCAGCCCAGGGCTCACACCCCAACCCCATCCAGCGGGAGGTCAAGACCAAGCTCTTCAGCCTGGAGGACTTCCTCAATGAGCTCATCCTATTCCAGAAGGGCCAGACCAACACCCCACCTCCATTCGAGATCTTCTTCTGCTTTGGGGAGGAGTGGCCTGACTGCAAACCCCGGGAGAAGAAGCTCATCACTGTACAGGTGGTACCCGTAGCAGCCCGGATGTTGCTGGAGATGTTCTCGGGGGAGCTTTCTTGGTCAGCTGATAGCATCCGGCTACAGATCTCAAACCCAGACCTCAAAGACCGCATGGTGGAACAGTTCAAGGAGCTCCATCACATCTGGCTGTCCCAGCAGCATCTGCAGCCTGTAGCCCAAACCCCTGCC---ATGCCAGGCCTCAGTGCTGCACAGGGACCCTGGCCCATGCACCCAGTTGGCATGCAGTAA------------------------------------------------------------------------------

>Megabat_ENSPVAG00000015756

------------------------------------------------------------------------------------------------------------------------------------------------------------------------------------------------------------------------------------------------------------------------------------------------------------------------------------------------------------------------------------------------------------------------------------------------------------------------------------------------------------ATGAACCAGCCTGCCCCAGGTGCCCTCCCGCCGCCCCGCCGTGTGCGGCTGAAGCCCTGGCTGGTGGCCCAGGTGAACAGCTGCCAGTACCCAGGGCTTCAGTGGGTCAATGGTGAAAGGAAATTCTTCTACATCCCCTGGCGACATGCCACACGGCACGGCCCAAGCCAGGATGGAGATAACACCATCTTCAAGGCG---------------GGGAAGTAC---GAGGGGGTGGACGAGGCCGATCCGGCCAAGTGGAAGGCCAAC---CGCTGTGCC---AACAAGAGCCGTGACTTCCGC---ATCTATGACGGGCCCCGGGACATG---CCACAGCCCTACAAGATCTACGAGGTCTGCTCCAATGGCCCTGCTCCCGCAGAGTCACAGCCCAGCGAGGACCACACG---CGTGGTGCAGGAGAGGAGGAGGAGGAGGAGGAGGAA------------------------------------------------CTCCAGAGGATGTTACCGAGCCTGAGCCTCACAGAAGCAGCGCAGCCTGGTCCCCCCATGGCACCGTATTCTTTACCCAAAGAGGATGTCAAGTGG---------------------------------------------CCACCTACTCTGCAGCCTCCTGCAGTGCTGGGCCCCCCTGCTCCAGGACCCAACCTCCTGGGCCCTGCCCCCGCC------------TATGGGGAG------CTTCTCCCTGAGGTC---------------CTGGAGCCT---GGGGCCCTGGCTGCCAGCCTGCCCCCTGCAAGCGAACAACTCCTGCCC------------------------------------------GACCTGCTGATC---------AGCCCTCACATGCTGCCT---CTGACTGACCTGGAGATCAAGTTCCAGTACCGTGGGCGGCCACCCCGTGCCCTCACCATCAGCAACCCACATGGCTGCCGGCTCTTCTACAGCCAGCTAGAGGCCACCCAGGAGCAGGTGGAGCTCTTTGGCCCTGTGAGCTTGGAGCAAGTGCGCTTCCCCAGCCCTGAGGACATCCCCAGTGACAAGCAGCGCTTCTACACGAACCAGCTGCTGGATGTCCTGGACCGCGGGCTCATCCTCCAGTTGCAAGGTCAGGATCTGTATGCCATCCGCCTGTGCCAGTGCAAGGTGTTCTGGAGTGGGCCCTGTGCCTTTGCTCATGGGTCACACCCCAACCCCATCCAGCGAGAGGTCAAGACCAAGCTCTTCAGCCTGGAGAATTTTCTCAATGGGCTCATCCTGTTCCAGAAGGGCCAGACCAACACCCCACCACCGTTTGAAATCTTCTTCTGCTTTGGGGAGGAGTGGCCTGACCGCAAACCCCGAGAGAAGAAGCTCATCACTGTACAGGTGGTGCCCGTAGCAGCTCGGTTGTTGCTGGAGATGTTCTCAGGGGAGCTTTCCTGGTCGGCTGATAGTATCCGGCTACAGATCTCAAACCCAGACCTCAAAGACCACATGGTGGAACAGTTCAAGGAGCTCCATCACATCTGGCAGTCTCAGCAGCAGTTGCAGCCAGTGGCCCAGGCCCCTCCT---------------------GCTGGTGGGGCCTGGCCCATGCACCCAGCCGGCATGCAA---------------------------------------------------------------------------------

>Dog_ENSCAFG00845021435

---------------------------------------------------------------------------------------------------ATGTTCACAGTGAGTCAGATTTCTGAGTTGTCTGGTCTGGCCACTTTCATTTCCCCTGGGTCTGGGTGGAGTTTGGTTCAGAAAGGGGAACTGAGCCACCAGTCTTTTGAGGCCTGGGGACTGGGCTGGGGGGTCTCTTACTCTTCCTGTCGCAGCCCGGATCTGGGCACTGCTCTGAAGAGAGGCCTGGCCTATGGAGACTTTTTAGCAGCGTGGAAGTGGCGCTTAACAACTACAACCCAAAAAGACCCCTTGGCC---------------------------------------------------------------------------------------------------------------------------------------ATGAACCAGCCTGCCCCCAGGGCACCCCCTCCGCCCCGCCGCGTGCGACTGAAGCCCTGGCTGGTGGCCCAGGTGAACAGCTGCCAGTACCCAGGGCTTCAGTGGGTCAATGGGGAAAGGAAATTCTTCTGCATCCCCTGGCGCCATGCCACGCGACATGGCCCCAGCCAGGAGGGGGATAATACCATCTTCAAGGCCTGGGCCAAGGAGACAGGAAAGTACACTGAGGGGGTGGACGAGGCGGACCCAGCCAAGTGGAAGGCCAACCTGCGCTGTGCCCTTAACAAGAGCCGTGACTTCCGCCTCATCTATGATGGACCCCGGGACATGCCGCCTCAGCCCTACAAGATCTACGAGGTCTGCTCTAATGGCTCCGCTCCCGCAGAGTCCCAGCCTAGTGAGGATTACACT---TTTGGTGCAGGAGAGGAGGAGGAGGAAGAAGAGGAAGACGTGAGCTCGGGGCCATTCCTGGCAGCGTCGGCCTGCACTGACGTGCTCCAGAGGATGTTACCAAGCCTGAGCATCACAGAAGCAGTGCAACCTGGCCCCCCCATGGCACCCTATTCTTTACCCAAAGAGGATGTCAAGTGG---------------------------------------------CCGCCCACCCTCCAGCCGCCTGTGGTGCTGGGCCCTCCTGCACCAGACCCCAGCCTGCTGGGCCCCACCCCTGGCAACCCTGCTGGCTTTGGGGAG------CTTCTCCCTGAAGTCCTGCCGAGC------CTGCAGCCT---GGGCCCCTGGCTGCCAGCCTGCCCCCCACAGGCGAACAACTCCTGCCC------------------------------------------GACCTGCTAATC---------AGCCCCCACATGCTGCCT---CTGACGGACCTGGAGATCAAGTTCCAGTACCGGGGGCGGCCACCCCGTGCCCTCACCATCAGCAACCCCCAGGGGTGCCGGCTCTTCTACAGTCAGCTGGAGCCCACCCAGGACCAGGTAGAGCTCTTCGGCCCCGTGAGCCTGGAGCAAGTGCGCTTCCCCAGCCCTGAGGACATCCCCAGCGACAAGCAGCGCTTCTACACAAACCAGCTGCTGGATGTCCTGGACCGCGGCCTCATACTCCAGCTGCAGGGCCAAGATCTGTATGCCATCCGCCTGTGCCAGTGCAAAGTGTTCTGGAGCGGGCCCTGCGCCTCGGCCCACGGCTCACACCCCAACCCCATCCAGCGGGAGGTCAAAACCAAGCTTTTCAGCCTGGAGCATTTTCTCAATGAGCTCATCCTGTTCCAGAAGGGCCAGACTAACACCCCACCGCCATTTGAGATCTTCTTCTGCTTTGGGGAGGAGTGGCCTGACCGCAAACCCCGAGAGAAGAAGCTCATCACTGTACAGGTGGTGCCTGTAGCAGCTCGGCTGTTGCTGGAGATGTTCTCAGGGGAGCTTTCTTGGTCAGCTGATAGTATCCGGCTACAGATCTCAAACCCAGACCTCAAAGACCGCATGGTAGAGCAGTTCAAGGAGCTCCATCACCTCTGGCAGTCCCAGCAGCGGTTGCAGCCTGTGGCCCAGGCCCCCGCT---GTGGCAGGCCTCAGTGCTGGCCAGGGGGCCTGGCCCATGCACTCAGTTGGCATGCAACAATGA---------------------------------------------------------------------------

>MouseLemur_ENSMICG00000000138

------------------------------------------------------------------------------------------------------------------------------------------------------------------------------------------------------------------------------------------------------------------------------------------------------------------------------------------------------------------------------------------------------------------------------------------------------------------------------------------------------------ATGAACCAGCCCACCTCTGGCGCCCCTGCCCCGCCCCGCCGCGTGCGGCTGAAGCCCTGGCTGGTGGCCCAGGTGAACAGCTGCCAGTATCCAGGGCTTCAGTGGGTCAATGGGGAAAAGAAATTCTTCTACATCCCCTGGAGGCACGCCACGAGGCACGGCCCCAGCCAGGACGGGGATAACACCATCTTCAAGGCCTGGGCCACGGAGACGGGGAAGTACACCGAAGGGGTGGATGAGGCCGATCCAGCCAAGTGGAAGGCCAACCTGCGCTGTGCCCTTAACAAGAGCCGCGACTTCCGTCTCATCTATGATGGGCCCCGGGACATGCCGCCCCAGCCCTACAAGATCTACGAGGTCTGCTCCAACGGCCCCGCTCCCGCAGAGTCCCAGCCTGCTGAAGATTACGCT---GTTGGAGCAGGAGAGGAGGAGGAGGAGGAAGAGGAAGAG---------------------------------------------CTTCAGAGGATGTTACCAAACCTGAGCATCACAGAAGCAGTGCCGCCTGGCCCCGCCATGGCCCCCTATTCTTTACCTAAAGAGGATGTCAAGTGG---------------------------------------------CCACCCACCCTGCAGCCACCTGTAGTGATGGGCCCCCCTGCTCCGGACCCCAGGCTCATGGCCACTCCTCCTGGTGTCCCTGCTGGCTTCAGGGAG------CTTCTGCCAAGTGTG------------------GAGCCT---GGGCCCCTGGCTGCCAGCCTGCCCCCTGCAAGCGAGCAGCTCTTGCCC------------------------------------------GAACTGCTGATC---------AGCCCCCACATGCTGCCA---CTGACCGACCTGGAGATCAAGTTCCAGTACAGGGGACGGCCACCCCAGGCGGTGATCATTAGCAACCCGCACGGCTGCCGACTCTTCTACAGCCAGCTGGAGGCCACCCAGGAACAGGTGGAACTCTTCGGCCCCGTGAGCCTGGAGCAAGTGCGTTTCCCCAGCCCCGAGGACATCCTCAGCGACAAGCAGCGCTTCTACACAAACCAGCTACTGGATGTCCTGGACCGTGGGCTCATCCTCCAGCTGCAGGGCCAGGACCTATATGCCATCCGCCTGTGCCAGTGCAAGGTGTTCTGGAGCGGGCCCTGCGCCTCAGCCCATGGCTTGCGCCCCAACCCCATCCAGCGGGAAGTCAAGACCAAGCTCTTCAGCCTGGAGCATTTTCTCAATGAGCTCATCCTGTTCCAGAAAGGCCAGACCAATACCCCACCGCCCTTCGAGATCTTCTTCTGCTTTGGGGAGGAGTGGCCTGACTCCAAACCTCGAGAAAAGAAGCTGATTACCGTACAGGTGGTGCCCGTAGCAGCTCGGTTGCTGCTGGAGATGTTCTCAGGGGAGCTCTCTTGGTCAGCTGACAGTATCCGGCTACAGATCTCCAACCCAGACCTCAAAGACCGCATGGTGGAGCAGTTCAAGGAGCTCCATCACATCTGGCAGTCCCAGCAGCGGTTGCAGCCTGTGGCCCAGGCCCCTCCT---GTGGCAGGCCTTGGTGCTGGCCAGGGGCCCTGGCCCATGCACCCAGTTGGTATGCAGTAA------------------------------------------------------------------------------

>Tarsier_ENSTSYG00000032877

------------------------------------------------------------------------------------------------------------------------------------------------------------------------------------------------------------------------------------------------------------------------------------------------------------------------------------------------------------------------------------------------------------------------------------------------------------------------------------------------------------ATGAACCAGGCCCTCTCCGGGGCTGCTGCCCCACCTCGCCGAGTGAGGCTAAAGCCCTGGCTGGTGGCCCAGGTGAACAGCGGCCAGTACCCAGGGCTCCAGTGGGTCAACGGGGAGAAGAAATTCTTCTACATCCCCTGGAGACACGCCACGAGGCATGGTCCCAGCCAGGATGGAGATAACACCATCTTCAAGGCCTGGGCCAAGGAAACCGGGAAGTACACTGAAGGGGTGGATGAGGCCGACCCCGCCAAATGGAAGGCCAACCTGCGCTGCGCCCTCAACAAGAGCCGCGACTTCCGCCTCATCTATGACGGGCCCCGGGACATGCCGCCCCAGCCCTACAAGATCTACGAGGTCTGCTCCAATGGCCCCACTCCCACAGAGTCCCAGCCCCCCGAGGATTACTCC---TTTGCTGCAGGAGAGGAGGAAGAGGAGGAGGAGGAAGAG---------------------------------------------CTCCAGAGGATGCTGCCAAGCCTGAGCATCACAGAGGCAGTGCAGCCCGTCCCTCCCATGGCACCGTATTCTCTACCCAAAGAGGAGGTCAAGTGG---------------------------------------------CCACCCACCCTGCAGCCGCCCGTGGGGCTGGGACCCCCTGCTCCCGACCCCAGCCTCCTGGCCCCTCCCCCTGGCAACGCCGCCGGCTTCCAGGAG------CTTCTCTCTGGGGTCCTGCCAAAC------CTGGAGGCA---GGGCCCCTGGCTGCCAGCCTACCCCTTGCAGGCGAACAACTCCTGCCC------------------------------------------GACCTGCTCATC---------AGTCCCCACATGCTGCCC---CTGACTGACCTGGAGATCAAGTTCCAGTACCGGGGGCGGCCACCCCGGGCCCTGACCATCAGCAACCCGCACGGCTGCCGGCTCTTCTATAGCCAGCTGGAGGCCACGCAGGACCAGGTGGAGCTCTTCGGCCCCGTGAGTCTTGAGCAGGTGCGCTTTCCCAGCCCCGAGGACATCCCCAGCGACAAGCAGCGCTTCTACACAAACCAGCTGCTGGACGTCCTGGACCGCGGGCTCATCCTCCAGCTGCAGGGCCAGGACCTGTACGCCATCCGCCTGTGCCAGTGCAAGGTGTTCTGGAGCGGGCCCTGCGCCTCGGCCTTGGGCTCCCACCCCAACCCCATCCAGCGGGAGGTCAAGACCAAGCTCTTCAGTCTGGAGCACTTCCTCAACGAGCTCATCCTGTTCCAGAAGGGCCAGACCAACACCCCACCGCCCTTCGAGATCTTCTTCTGCTTTGGGGAGGAGTGGCCTGACCACAAACCCCGAGAGAAGAAGCTCATTACTGTACAGGTGGTGCCCGTGGCAGCCCGGCTGCTGCTGGAGATGTTCTCAGGGGAGCTATCTTGGTCGGCAGACAGTATCCGACTTCAGATCTCAAACCCGGACCTCAAGGACCGCATGGTGGAGCAGTTCAAGGAGCTCCATCACATCTGGCAGTCCCAGCAGCGGCTGCAGCCTGTGGTCCAGGCCCCTCCT---GGAACAGGCCTTGGC---GGCCAGGGGCCCTGGCCTATGCACCTGGTTGGCATGCAGTAA------------------------------------------------------------------------------

>Gibbon_ENSNLEG00000012454

------------------------------------------------------------------------------------------------------------------------------------------------------------------------------------------------------------------------------------------------------------------------------------------------------------------------------------------------------------------------------------------------------------------------------------------------------------------------------------------------------------ATGAACCAGTCCATCCCAGTGGCTCCCACCCCACCCCGCCGCGTGAGGCTGAAGCCCTGGCTGGTGGCCCAGGTGAACAGCTGCCAGTACCCAGGGCTTCAATGGGTCAACGGGGAAAAGAAATTATTCTGCATCCCCTGGAGGCATGCCACGAGGCATGGTCCCAGCCAGGACGGAGATAACACCATCTTCAAGGCCTGGGCCAAGGAGACAGGGAAATACACCGAAGGCGTGGATGAAGCCGATCCGGCCAAGTGGAAGGCCAACCTGCGCTGTGCCCTTAACAAGAGCCGAGACTTCCGCCTCATCTACGACGGGCCCCGGGACATGCCACCTCAGCCCTACAAGATTTACGAGGTCTGCTCCAATGGCCCCGCTCCCACAGACTCCCAGCCCCCTGAGGATTACTCT---TTTGGTGCAGGAGAGGAGGAGGAGGAGGAAGAGGAAGAG---------------------------------------------GTGAGTGTGGGCTGTCCACACGCACTCTCTGTAGATGCAGTGCAGTCTGGCCCCCACGTGGCACCCTATTCTTTACTCAAAGAGGATGTCAAGTGG---------------------------------------------CCGCCCACTCTGCAGCCGCCCGTGGTGCTGGGTCCCCCTGCTCCGGACCCCAGTCCCCTGGCTCCTCCCCCTGGCAACCCT---------------------------------GTC---------------CTGGAGCCT---GGGCCCCTGGCTGCCAGCTTGCCCCCTGCAGGCGAACAGCTCCTGAAGGTGGAGGGTGCTGGACTCCCTTGGGTGGGGAAAGTGGGAGAGCAGATGGGGCTGGGACGGGATGGGCCTGCCTTCTGCCCCACAGTGACCGACCTGGAGATCAAGTTTCAGTACCGGGGGCGGCCACCCCGGGCCCTCACCATCAGCAACCCCCATGGCTGCCGGCTCTTCTACAGCCAGCTAGAGGCCACCCAGGAGCAGGTGGAACTCTTTGGCCCCATAAGCCTGGAACAAGTGCGCTTCCCCAGCCCTGAGGACATCCCCAGCGACAAGCAGCGCTTCTACACGAACCAGCTGCTGGATGTCCTGGACCGCGGGCTCATCCTCCAGCTACAAGGCCAGGACCTTTATGCCATCCGCCTGTGTCAGTGCAAGGTGTTCTGGAGCGGGCCTTGTGCCTCAGCCCATGACTCATGCCCCAACCCCATCCAGCGGGAGGTCAAGACCAAACTTTTCAGCCTGGAGCATTTTCTCAATGAGCTCATCCTGTTCCAGAAGGGCCAGACCAACACCCCACCACCCTTCGAGATCTTCTTCTGCTTCGGGGAAGAATGGCCTGACCGCAAACCCCGAGAGAAGAAGCTCATTACTGTACAGGTAGTGCCTGTAGCAGCTCGACTGCTGCTGGAGATGTTCTCAGGGGAGCTATCTTGGTCAGCTGATAGTATCCGGCTACAGATCTCAAACCCAGACCTCAAAGACCGCATGGTAGAGCAATTCAAGGAGCTCCATCACATCTGGCAGTCCCAGCAGCGGTTGCAGCCTGTGGCCCAGGCCCCTCCT---GGGGCAGGCCTTGGTGTTGGCCAGGGGCCCTGGCCTATGCACCCAGCTGGCATGCAATAA------------------------------------------------------------------------------

>MasNightMonkey_ENSANAG00000038213

------------------------------------------------------------------------------------------------------------------------------------------------------------------------------------------------------------------------------------------------------------------------------------------------------------------------------------------------------------------------------------------------------------------------------------------------------------------------------------------------------------ATGAACCAGTCCATCCCGGGGGCTCCCACCCCGCCCCGCCGCGTGCGGCTGAAGCCCTGGCTGGTGGCCCAGGTGAACAGCTGCCAGTACCCAGGGCTTCAATGGGTCAACGGGGAAAAGAAATTCTTCTATATCCCCTGGAGGCATGCCACAAGGCATGGTCCCAGCCAGGACGGAGATAACACCATCTTCAAGGCCTGGGCCAAGGAGACAGGGAAATACACTGAAGGGGTGGATGAAGCTGATCCAGCCAAGTGGAAGGCCAACCTGCGCTGCGCCCTTAACAAGAGCCGAGACTTCCGCCTCATCTACGACGGGCCTCGGGACATGCCACCTCAGCCCTACAAGATCTACGAGGTCTGCTCCAATGGCCCCACTCCCACAGAGTCCCAGCCCCCTGAGGATTACTCT---ATT---CCAGGAAAGGAGGAGGAGGAGGAAGAGGAAGAG---------------------------------------------CTGCAGAGGATGTTGCCAAGTCTGAGCCTCACAGATGCAGTGCCGCCGGGCCCCGCCATGGCACCCTATTCTTTACCCAAAGAGGATGTCAAGTGG------------------------------CCACCCACTCTGCAGCCGCCCACTCTGCAGCCACCCGTGGTGCTGGGTCCCCCTGCTCCGGACCCCAGTCCTCTGGCTCCTCCCCTTGGCAACCCTGCTGGCTTCAGGCAG------CTTCTCCCTGAGGTC---------------CTGGAGCCT---GGGCCCCTGGCTGCCAGCCTGCCCCCTGCAGGAGAACAGCTCCTGCCC------------------------------------------GACCTGCTGATC---------AGCCCCCATATGCTGCCT---CTGACGGACCTGGAGATCAAGTTCCAGTACCGGGGGCGGCCACCCCGGGCCCTCACCATCAGCAACCCCCACGGCTGCCGGCTCTTCTACAGCCAGCTGGAGGCCACCCAGGAGCAGGTGGAACTCTTCGGCCCCATAAGCCTGGAGCAAGTGCGCTTCCCCAGCCCTGAGGACATCCCCAGCGACAAGCAGCGTTTCTACACGAACCAGCTGCTGGATGTACTGGACCGCGGGCTCATCCTCCAGCTCCAGGGTCAGGACCTGTATGCCATCCGCCTGTGTCAGTGCAAGGTGTTCTGGAGCGGGCCTTGTGCCTCAGCCCATGCCTCATGCCCCAACCCCATCCAGCGGGAGGTCAAGACCAAGCTCTTCAGCCTGGAGCACTTTCTCAATGAGCTCATCCTGTTCCAGAAGGGGCAGACCAACACCCCACCACCCTTCGAGATCTTCTTCTGCTTTGGGGAAGAATGGCCTGACCGCAAACCCCGAGAGAAGAAGCTCATTACTGTACAGGTGGTGCCTGTAGCAGCTCGAATGCTGCTGGAGATGTTCTCAGGGGAGCTATCTTGGTCAGCAGATAGTATCCGGCTACAGATCTCAAACCCAGACCTCAAAGACCGCATGGTGGAGCAGTTCAAGGAGCTTCATCACATCTGGCAGTCTCAGCAGCGGTTGCAGTCTGTGGCCCAGGCCCCTCCT---GGGGCAGGCCTTGGTGCTGGCCAGGGGCCCTGGACTATGCACCCAGCTGGCATGCAGTAA------------------------------------------------------------------------------

>Marmoset_ENSCJAG00000018061

------------------------------------------------------------------------------------------------------------------------------------------------------------------------------------------------------------------------------------------------------------------------------------------------------------------------------------------------------------------------------------------------------------------------------------------------------------------------------------------------------------ATGAACCAGTCCATCCCAGGGGCTCCCACCCCGCCCCGCCGCGTGCGGCTGAAGCCCTGGCTGGTGGCCCAGGTGAACAGCTGCCAGTACCCAGGGCTTCAATGGGTCAACGGGGAAAAGAAATTCTTCTACATCCCCTGGAGGCATGCCACAAGGCATGGTCCCAGCCAGGACGGAGATAACACCATCTTCAAGGCCTGGGCCAAGGAGACAGGGAAATACACTGAAGGGGTGGATGAAGCCGATCCAGCCAAGTGGAAGGCCAACCTGCGCTGCGCCCTTAACAAGAGCCGAGACTTCCGCCTCATCTATGACGGGCCCCGGGACATGCCACCTCAGCCCTACAAGATCTACGAGGTCTGCTCCAATGGCCCCGCTCCTACAGAGTCCCAGCCCTCTGAGGATTACTCT---ATT---CCAGGAGAAGAGGAGGAGGAGGAAGAGGAAGAA---------------------------------------------CTGCAGAGAATGTTGCCAAGTCTGAGCCTCACAGATGCAGTGCCGCCGGGCCCTGCCATGCCACCCTATTCTTTACCCAAAGAGAATGTCAAGTGG------------------------------CCACCCACTTTGCAGCTGCCCACTCTGCAGCCACCCGTGGTGCTGGGTCCCCCTGCTCCGGACCCCAGTCCCCTGGCTCCTCCCCTTAGCAACCCTGCTGGCTTCAGGGAG------CTTCTCCCTGAGGTC---------------CTGGAGCCT---GGGCCCCTGGATGCCAGCCTGCCCCCTGCAGACGAACAGCTCCTGCCT------------------------------------------GACCTACTGATC---------AGTCCCCACATGCTGCCT---TTGACTGACCTGGAGATCAAGTTCCAGTACCGGGGGCGGCCACCCCGGGCCCTCACCATCAGCAACCCCCATGGCTGCCGGCTCTTCTACAGCCAGCTGGAAGCCACCCAGGAGCAGGTGGAACTCTTTGGCCCCATAAGCCTGGAGCAAGTGCGCTTCCCCAGCCCTGAGGACATCCCCAGTGACAAGCAGCGCTTCTATACGAACCAGCTGCTGGATGTCCTGGACCGCGGGCTCATCCTCCAGCTCCAGGGCCAGGACCTGTATGCCATCCGCCTGTGTCAGTGCAAGGTGTTCTGGAGCGGGCCTTGTGCCTCAGCCGATGACTCATGCCCCAACCCCATCCAGCGGGAGGTCAAGACCAAGCTCTTCAGTCTGGAGCACTTTCTCAATGAGCTCATCCTGTTCCAGAAGGGGCAGACCAACACCCCACCACCCTTTGAGATCTTCTTCTGCTTTGGGGAAGAATGGCCTGACCGCAAACCCCGGGAGAAGAAGCTCATTACTGTACAGGTGGTGCCTGTAGCAGCTCGAATGCTGCTGGAGATGTTCTCAGGGGAGCTATCTTGGTCAGCAGATAGTATCCGGCTACAGATCTCAAACCCAGACCTCAAAGACCGCATGGTGGAGCAGTTCAAGGAGCTTCATCACATCTGGCAGTCTCAGCAGCGGTTGCAGCCTGTGGCCCAGGCCCCTCCT---GGGGCAGGCCTTGGTGCTGGCCAGGGGCCCTGGACTATGCACCCAGCTGGTATGCAGTAA------------------------------------------------------------------------------

>Gorilla_ENSGGOG00000012013

------------------------------------------------------------------------------------------------------------------------------------------------------------------------------------------------------------------------------------------------------------------------------------------------------------------------------------------------------------------------------------------------------------------------------------------------------------------------------------------------------------ATGAACCAGTCCATCCCAGTGGCTCCCACCCCACCCCGCCGCGTGCGGCTGAAGCCCTGGCTGGTGGCCCAGGTGAACAGCTGCCAGTACCCAGGGCTTCAATGGGTCAACGGGGAAAAGAAATTATTCTGCATCCCCTGGAGGCATGCCACGAGGCATGGTCCCAGCCAGGACGGAGATAACACCATCTTCAAGGCCTGGGCCAAGGAAACAGGGAAATACACCGAAGGCGTGGATGAAGCCGATCCGGCCAAGTGGAAGGCCAACCTGCGCTGTGCCCTTAACAAGAGCCGGGACTTCCGCCTCATCTACGACGGGCCCCGGGACATGCCACCTCAGCCCTACAAGATCTACGAGGTCTGCTCCAATGGCCCTGCTCCCACAGACTCCCAGCCCCCTGAGGATTACTCT---TTTGGTGCAGGAGAGGAGGAGGAAGAAGAGGAAGAG------------------------------------------------CTGCAGAGGATGTTGCCAAGCCTGAGCCTCACAGATGCAGTGCAGTCTGGCCCCCACATGACACCCTATTCTTTACTCAAAGAGGATGTCAAGTGG---------------------------------------------CCGCCCACTCTGCAGCTGCCCGTGGTGCTGGGTCCCCCTGCTCCAGACCCCAGCCCCCAGGCTCCTCCCCCTGGCAACCCTGCTGGCTTCAGGGAG------CTTCTCCCTGAGGTC---------------CTGGAGCCT---GGGCCCCTGGCTGCCAGCCTGCCCCCTGCAGGCGAACAGCTCCTGCCA------------------------------------------GACCTTCTGATC---------AGCCCCCACATGCTGCCT---CTGACCGACCTGGAGATCAAGTTTCAGTACCGGGGGCGGCCACCCCGGGCCCTCACCATCAGCAACCCCCATGGCTGCCGGCTCTTCTACAGCCAGCTGGAGGCCACCCAGGAGCAGGTGGAACTCTTCGGCCCCATAAGCCTGGAGCAAGTGCGCTTCCCCAGCCCTGAGGACATCCCCAGTGACAAGCAGCGCTTCTACACGAACCAGCTGCTGGATGTCCTGGACCGCGGGCTCATCCTCCAGCTACAGGGCCAGGACCTTTATGCCATCCGCCTGTGTCAGTGCAAGGTGTTCTGGAGCGGGCCTTGTGCCTCAGCCCATGACTCATATCCCAACCCCATCCAGCGGGAGGTCAAGACCAAGCTTTTCAGCCTGGAGCATTTTCTCAATGAGCTCATCCTGTTCCAAAAGGGCCAGACCAACACCCCACCACCCTTCGAGATCTTCTTCTGCTTTGGGGAAGAATGGCCTGACCGCAAACCCCGAGAGAAGAAGCTCATTACTGTACAGGTGGTGCCTGTAGCAGCTCGACTGCTGCTGGAGATGTTCTCAGGGGAGCTATCTTGGTCAGCTGATAGTATCCGGCTACAGATCTCAAACCCAGACCTCAAAGACCGCATGGTGGAGCAATTCAAGGAGCTCCATCACATCTGGCAGTCCCAGCAGCGGTTGCAGCCTGTGGCCCAGGCCCCTCCT---GGAGCAGGCCTTGGTGTTGGCCAGGGGCCCTGGCCTATGCACCCAGCTGGCATGCAATAA------------------------------------------------------------------------------

>Human_ENSG00000128604

------------------------------------------------------------------------------------------------------------------------------------------------------------------------------------------------------------------------------------------------------------------------------------------------------------------------------------------------------------------------------------------------------------------------------------------------------------------------------------------------------------ATGAACCAGTCCATCCCAGTGGCTCCCACCCCACCCCGCCGCGTGCGGCTGAAGCCCTGGCTGGTGGCCCAGGTGAACAGCTGCCAGTACCCAGGGCTTCAATGGGTCAACGGGGAAAAGAAATTATTCTGCATCCCCTGGAGGCATGCCACAAGGCATGGTCCCAGCCAGGACGGAGATAACACCATCTTCAAGGCCTGGGCCAAGGAGACAGGGAAATACACCGAAGGCGTGGATGAAGCCGATCCGGCCAAGTGGAAGGCCAACCTGCGCTGTGCCCTTAACAAGAGCCGGGACTTCCGCCTCATCTACGACGGGCCCCGGGACATGCCACCTCAGCCCTACAAGATCTACGAGGTCTGCTCCAATGGCCCTGCTCCCACAGACTCCCAGCCCCCTGAGGATTACTCT---TTTGGTGCAGGAGAGGAGGAGGAAGAAGAGGAAGAG------------------------------------------------CTGCAGAGGATGTTGCCAAGCCTGAGCCTCACAGATGCAGTGCAGTCTGGCCCCCACATGACACCCTATTCTTTACTCAAAGAGGATGTCAAGTGGCCGCCCACTCTGCAGCCGCCCACTCTGCGGCCGCCTACTCTGCAGCCGCCCACTCTGCAGCCGCCCGTGGTGCTGGGTCCCCCTGCTCCAGACCCCAGCCCCCTGGCTCCTCCCCCTGGCAACCCTGCTGGCTTCAGGGAG------CTTCTCTCTGAGGTC---------------CTGGAGCCT---GGGCCCCTGCCTGCCAGCCTGCCCCCTGCAGGCGAACAGCTCCTGCCA------------------------------------------GACCTGCTGATC---------AGCCCCCACATGCTGCCT---CTGACCGACCTGGAGATCAAGTTTCAGTACCGGGGGCGGCCACCCCGGGCCCTCACCATCAGCAACCCCCATGGCTGCCGGCTCTTCTACAGCCAGCTGGAGGCCACCCAGGAGCAGGTGGAACTCTTCGGCCCCATAAGCCTGGAGCAAGTGCGCTTCCCCAGCCCTGAGGACATCCCCAGTGACAAGCAGCGCTTCTACACGAACCAGCTGCTGGATGTCCTGGACCGCGGGCTCATCCTCCAGCTACAGGGCCAGGACCTTTATGCCATCCGCCTGTGTCAGTGCAAGGTGTTCTGGAGCGGGCCTTGTGCCTCAGCCCATGACTCATGCCCCAACCCCATCCAGCGGGAGGTCAAGACCAAGCTTTTCAGCCTGGAGCATTTTCTCAATGAGCTCATCCTGTTCCAAAAGGGCCAGACCAACACCCCACCACCCTTCGAGATCTTCTTCTGCTTTGGGGAAGAATGGCCTGACCGCAAACCCCGAGAGAAGAAGCTCATTACTGTACAGGTGGTGCCTGTAGCAGCTCGACTGCTGCTGGAGATGTTCTCAGGGGAGCTATCTTGGTCAGCTGATAGTATCCGGCTACAGATCTCAAACCCAGACCTCAAAGACCGCATGGTGGAGCAATTCAAGGAGCTCCATCACATCTGGCAGTCCCAGCAGCGGTTGCAGCCTGTGGCCCAGGCCCCTCCT---GGAGCAGGCCTTGGTGTTGGCCAGGGGCCCTGGCCTATGCACCCAGCTGGCATGCAATAA------------------------------------------------------------------------------

>Bonobo_ENSPPAG00000031382

------------------------------------------------------------------------------------------------------------------------------------------------------------------------------------------------------------------------------------------------------------------------------------------------------------------------------------------------------------------------------------------------------------------------------------------------------------------------------------------------------------ATGAACCAGTCCATCCCAGTGGCTCCCACCCCACCCCGCCGTGTGCGGCTGAAGCCCTGGCTGGTGGCCCAGGTGAACAGCTGCCAGTACCCAGGGCTTCAATGGGTCAACGGGGAAAAGAAATTATTCTGCATCCCCTGGAGGCATGCCACAAGGCATGGTCCCAGCCAGGACGGAGATAACACCATCTTCAAGGCCTGGGCCAAGGAGACAGGGAAATACACCGAAGGCGTGGATGAAGCCGATCCGGCCAAGTGGAAGGCCAACCTGCGCTGTGCCCTTAACAAGAGCCGGGACTTCCGCCTCATCTACGACGGGCCCCGGGACATGCCACCTCAGCCCTACAAGATCTACGAGGTCTGCTCCAATGGCCCTGCTCCCACAGACTCCCAGCCCCCTGAGGATTACTCT---TTTGGTGCAGGAGAGGAGGAGGAAGAAGAGGAAGAG------------------------------------------------CTGCAGAGGATGTTGCCAAGCCTGAGCCTCACAGATGCAGTGCAGTCTGGCCCCCACATGACACCCTATTCTTTACTCAAAGAGGATGTCAAGTGGCCGCCCACTCTGCAGCCGCCCACTCTGCGGCCGCCTACTCTGCAGCCGCCCACTCTGCAGCCGCCCGTGGTGCTGGGTCCCCCTGCTCCAGACCCCAGCCCCCTGGCTCCTCCCCCTGGCAACCCTGCTGGCTTCAGGGAG------CTTCTCCCTGAGGTC---------------CTGGAGCCT---GGGCCCCTGCCTGCCAGCCTGCCCCCTGCAGGCGAACAGCTTCTGCCA------------------------------------------GACCTGCTGATC---------AGCCCCCACATGCTGCCT---CTGACCGACCTGGAGATCAAGTTTCAGTACCGGGGACGGCCACCCCGGGCCCTCACCATCAGCAACCCCCATGGCTGCCGGCTCTTCTACAGCCAGCTGGAGGCCACCCAGGAGCAGGTGGAACTCTTCGGCCCCATAAGCCTGGAGCAAGTGCGCTTCCCCAGCCCTGAGGACATCCCCAGTGACAAGCAGCGCTTCTACACGAACCAGCTGCTGGATGTCCTGGACCGCGGGCTCATCCTCCAGCTACAGGGCCAGGACCTTTATGCCATCCGCCTGTGTCAGTGCAAGGTGTTCTGGAGCGGGCCTTGTGCCTCAGCCCATGACTCATGCCCCAACCCTATCCAGCGGGAGGTCAAGACCAAGCTTTTCAGCCTGGAGCATTTTCTCAATGAGCTCATCCTGTTCCAAAAGGGCCAGACCAACACCCCACCACCCTTCGAGATCTTCTTCTGCTTTGGGGAAGAATGGCCTGACCGCAAACCCCGAGAGAAGAAGCTCATTACTGTACAGGTGGTGCCTGTAGCAGCTCGACTGCTGCTGGAGATGTTCTCAGGGGAGCTATCTTGGTCAGCTGATAGTATCCGGCTACAGATCTCAAACCCAGACCTCAAAGACCGCATGGTGGAGCAATTCAAGGAGCTCCATCACATCTGGCAGTCCCAGCAGCGGTTGCAGCCTGTGGCCCAGGCCCCTCCTGCTGGAGCAGGCCTTGGTGTTGGCCAGGGGCCCTGGCCTATGCACCCAGCTGGCATGCAATAA------------------------------------------------------------------------------

>Chimpanzee_ENSPTRG00000019675

------------------------------------------------------------------------------------------------------------------------------------------------------------------------------------------------------------------------------------------------------------------------------------------------------------------------------------------------------------------------------------------------------------------------------------------------------------------------------------------------------------ATGAACCAGTCCATCCCAGTGGCTCCCACCCCACCCCGCCGTGTGCGGCTGAAGCCCTGGCTGGTGGCCCAGGTGAACAGCTGCCAGTACCCAGGGCTTCAATGGGTCAACGGGGAAAAGAAATTATTCTGCATCCCCTGGAGGCATGCCACAAGGCATGGTCCCAGCCAGGACGGAGATAACACCATCTTCAAGGCCTGGGCCAAGGAGACAGGGAAATACACCGAAGGCGTGGATGAAGCCGATCCGGCCAAGTGGAAGGCCAACCTGCGCTGTGCCCTTAACAAGAGCCGGGACTTCCGCCTCATCTACGACGGGCCCCGGGACATGCCACCTCAGCCCTACAAGATCTACGAGGTCTGCTCCAATGGCCCTGCTCCCACAGACTCCCAGCCCCCTGAGGATTACTCT---TTTGGTGCAGGAGAGGAGGAGGAAGAAGAGGAAGAG------------------------------------------------CTGCAGAGGATGTTGCCAAGCCTGAGCCTCACAGATGCAGTGCAGTCTGGCCCCCACATGACACCCTATTCTTTACTCAAAGAGGATGTCAAGTGGCCGCCCACTCTGCAGCCGCCCACTCTGCGGCCGCCTACTCTGCAGCCGCCCACTCTGCAGCCGCCCGTGGTGCTGGGTCCCCCTGCTCCAGACCCCAGCCCCCTGGCTCCTCCCCCTGGCAACCCTGCTGGCTTCAGGGAG------CTTCTCCCTGAGGTC---------------CTGGAGCCT---GGGCCCCTGCCTGCCAGCCTGCCCCCTGCAGGCGAACAGCTCCTGCCA------------------------------------------GACCTGCTGATC---------AGCCCCCACATGCTGCCT---CTGACCGACCTGGAGATCAAGTTTCAGTACCGGGGGCGGCCACCCCGGGCCCTCACCATCAGCAACCCCCATGGCTGCCGGCTCTTCTACAGCCAGCTGGAGGCCACCCAGGAGCAGGTGGAACTCTTCGGCCCCATAAGCCTGGAGCAAGTGCGCTTCCCCAGCCCTGAGGACATCCCCAGTGACAAGCAGCGCTTCTACACGAACCAGCTGCTGGATGTCCTGGACCGCGGGCTCATCCTCCAGCTACAGGGCCAGGACCTTTATGCTATCCGCCTGTGTCAGTGCAAGGTGTTCTGGAGCGGGCCTTGTGCCTCAGCCCATGACTCATGCCCCAACCCTATCCAGCGGGAGGTCAAGACCAAGCTTTTCAGCCTGGAGCATTTTCTCAATGAGCTCATCCTGTTCCAAAAGGGCCAGACCAACACCCCACCACCCTTCGAGATCTTCTTCTGCTTTGGGGAAGAATGGCCTGACCGCAAACCCCGAGAGAAGAAGCTCATTACTGTACAGGTGGTGCCTGTAGCAGCTCGACTGCTGCTGGAGATGTTCTCAGGGGAGCTATCTTGGTCAGCTGATAGTATCCGGCTACAGATCTCAAACCCAGACCTCAAAGACCGCATGGTGGAGCAATTCAAGGAGCTCCATCACATCTGGCAGTCCCAGCAGCGGTTGCAGCCTGTGGCCCAGGCCCCTCCTGCTGGAGCAGGCCTTGGTGTTGGCCAGGGGCCCTGGCCTATGCACCCAGCTGGCATGCAATAA------------------------------------------------------------------------------

>Macaca_ENSMMUG00000018478

---------------------------------------------------------------------------------------------------ATGCACAAAGACTGCCTGCAGGTGTGGGGTGTGGGGAAGAGCTCTGGGTGGGAGGTTGGAAATTTGGTCTGGGGGACCCACTCGACTCCCTCCGTCAGCCCACAGTCAGTTTGGTTTCTGAGTTGTCCCAGTCCAGCCACTTTCGTTTCCCCTGGGGCTGGGTGGAGGCTGGGGCAGAAAGCGGAACTGAGCCCCCATGTTCTGAGGTCGGGGCAGGGCTGGAGCGTTCTGAACACCTCCCAGTCCCAGCCCCTGGGCCAGGCAAGGCCGGCCGGCCTTACGTCTCCTGGGTTGGTGGCAGCCGAGCTGGGCTCTGAGGGAGGCCTACAATGTGAGACACTAGCAGCTCAGAGGCAGCACGAGGCAGGTGCAAACCCAAAAGACCCTTCTGCCATGAACCAGTCCATCCCAGGCGCTCCCACCCCGCCCCGCCGCGTGCGGCTGAAGCCCTGGCTGGTGGCCCAGGTGAACAGCTGCCAGTACCCAGGGCTTCAATGGGTCAACGGGGAAAAGAAATTATTCTACATCCCCTGGAGGCATGCCACGAGGCATGGTCCCAGCCAGGACGGAGATAACACCATCTTCAAGGCCTGGGCCAAGGAGACTGGGAAATACACTGAAGGCGTGGATGAAGCTGATCCGGCCAAATGGAAGGCCAACTTGCGCTGTGCCCTTAACAAGAGCCGGGACTTCCGCCTCTTCTACGATGGGCCCCGGGACATGCCACCTCAGCCCTACAAGATCTACGAGGTCTGCTCCAATGGCCCTGCTCCCACAGACTCCCAGCCCCCTGAGGATTACTCT---TTTGGTGCAGGAGAGGAGGAGGAGGAGGAAGAGGAAGAG---------------------------------------------CTGCAGAGGATGTTGCCAAGCCTGAGCCTCACAGATGCAGTGCAGTCTGGCCCCCACATGGCACCCTATTCTTTACTCAAAGATGATGTCAAGTGG---------------------------------------------CCGCCCACTCTGCAGCCGCCCGTGGTGCTGGGTCCCCCTGCTCCAGACCCCAGACCCCTGGCTCCTCCCCCTGGCAACCCTGCTGGCTTCAGGGAG------CTTTTCCCTGAGGTC---------------CTGGAGCCT---GGGCCCCTGGCTGCCAGCCTGCCCCCTGCAAGCGAACAGCTCCTGCCT------------------------------------------GACCTGCTGATC---------AGCCCCCACATGCTGCCT---CTGACTGACCTGGAGATCAAGTTTCAGTACCGGGGGCGGCCACCCCGGGCCCTCACTATCAGCAACCCCCATGGCTGCCGGCTCTTCTACAGCCAGCTGGAGGCCACCCAGGAGCAGGTGGAACTCTTTGGCCCCATAAGCCTGGAGCAAGTGCGCTTCCCCAGCCCTGAGGACATCCCCAGCGACAAGCAGCGCTTCTACACGAACCAGCTGCTGGATGTCCTGGACCGCGGGCTCATCCTCCAGTTACAGGGCCAGGACCTGTATGCCATCCGCCTGTGTCAGTGCAAGGTGTTCTGGAGTGGGCCTTGTGCTTCAGCCCATGACTCATGCCCCAACCCCATCCAGCGGGAGGTCAAGACCAAGCTCTTCAGCCTGGAGCATTTTCTCAATGAGCTCATCCTGTTCCAGAAGGGCCAGACCAACGCCCCACCACCCTTCGAGATCTTCTTCTGCTTCGGGGAAGAATGGCCTGACCGCAAACCCCGAGAGAAGAAGCTCATTACTGTACAGGTAGTGCCTGTAGCAGCTCGATTGCTGCTGGAGATGTTCTCAGGGGAGCTATCTTGGTCAGCTGATAGTATCCGGCTACAGATCTCAAACCCAGACCTCAAAGACCACATGGTGGAGCAGTTCAAGGAGCTCCATCACATCTGGCAGTCCCAGCAGCGGTTGCAGCCTGTGGCCCAGGCCCCTCCT---GGGGCAGGCCTCGGTGCTGGCCAGGGGCCCTGGCCCATGCACCCAGCTGGCATGCAGTAA------------------------------------------------------------------------------

>GoldenSnubNoseMonkey_ENSRROG00000030142

------------------------------------------------------------------------------------------------------------------------------------------------------------------------------------------------------------------------------------------------------------------------------------------------------------------------------------------------------------------------------------------------------------------------------------------------------------------------------------------------------------ATGAACCAGTCCATCCCAGGGGCTCCCACCCCGCCCCGCCGCGTGCGGCTGAAGCCCTGGCTGGTGGCCCAGGTGAACAGCTGCCAGTACCCAGGGCTTCAATGGGTCAACGGGGAAAAGAAATTATTCTATATCCCCTGGAGGCATGCCACGAGGCATGGTCCCAGCCAGGACGGAGATAACACCATCTTCAAGGCCTGGGCCAAGGAGACTGGGAAATACACTGAAGGCGTGGATGAAGCTGATCCGGCCAAGTGGAAGGCCAACCTGCGCTGTGCCCTTAACAAGAGCCGGGACTTCCGCCTCTTCTACGATGGGCCCCGAGACATGCCACCTCAGCCCTACAAGATCTACGAGGTCTGCTCCAATGGCCCTGCTCCCACAGACTCCCAGCCCCCTGAGGATTACTCT---TATGGCCCAGGAGAGGAGGAGGAGGAGGAAGAGGAAGAG---------------------------------------------CTGCAGAGGATGTTGCCAAGCCTGAGCCTCACAGATGCAGTGCAGTCTGGCCCCCACATGGCACCCTATTCTTTACTCAAAGACGATGTCAAGTGG---------------------------------------------CCGCCCACTCTGCAGCCGCCCGTGGTGCTGGGTCCGCCTGCTCCAGACCCCAGACCCCTGGCTCCTCCCCCTGGCAACCCTGCTGGCTTCAGGGAG------CTTTTCCCTGAGGTC---------------CTGGAGCCT---GGGCCCCTGGCTGCCAGCCTGCCCCCTGCAAGCGAACAGCTCCTGCCT------------------------------------------GACTTGCTGATC---------AGCCCCCATATGCTGCCT---CTGACTGACCTGGAGATCAAGTTTCAGTACCGGGGGCGGCCACCCCGGGCCCTCACCATCAGCAACCCCCACGGCTGCCGGCTCTTCTACAGCCAGCTGGAGGCCACCCAGGAGCAGGTGGAACTCTTTGGCCCCATAAGCCTGGAGCAAGTGCGCTTCCCCAGCCCTGAGGACATCCCCAGTGACAAGCAGCGCTTCTACACGAACCAGCTGCTGGATGTCCTGGACCGCGGGCTCATCCTCCAGTTACAGGGCCAGGACCTGTATGCCATCCGCCTGTGTCAGTGCAAGGTGTTCTGGAGTGGGCCTTGTGCCTCGGTCCATGACTCATGCCCCAACCCCATCCAGCGGGAGGTCAAGACCAAGCTCTTCAGCCTGGAGCATTTTCTCAATGAGCTCATCCTGTTCCAGAAGGGCCAGACCAACACCCCACCACCCTTCGAGATCTTCTTCTGCTTCGGGGAAGAATGGCCTGACCGCAAACCCCGAGAGAAGAAGCTCATTACTGTACAGGTAGTGCCTGTAGCAGCTCGATTGCTGCTGGAGATGTTCTCAGGGGAGCTATCTTGGTCAGCTGATAGTATCCGGCTACAGATCTCAAACCCAGACCTCAAAGACCACATGGTGGAGCAGTTCAAGGAGCTCCATCACATCTGGCAGTCCCAGCAGCGGTTGCAGCCTGTGGCCCAGGCCCCTCCT---GGGGCAGGCCTCGGTGCTGGCCAGGGGCCCTGGCCTATGCACCCAGCTGGCATGCAGTAA------------------------------------------------------------------------------

# IRF6

>Megabat_ENSPVAG00000004865

------------------------------------------------------------------------------------------------------------------------------------------------------------------------------------------------------------------ATGGCCCTCCACCCCCGCAGAGTTCGGCTGAAGCCCTGGCTGGTGGCCCAGGTGGATAGCGGCCTGTACCCTGGGCTCATCTGGCTACACAGGGACTCCAAACGCTTCCAGATTCCCTGGAAACATGCCACCCGGCATAGCCCCCAGCAAGAGGAGGAAAACACCATTTTCAAGGCCTGGGCGGTGGAGACAGGAAAGTACCAGGAAGGGGTGGATGACCCTGACCCGGCCAAATGGAAGGCCCAGCTCCGCTGTGCTCTCAACAAGAGCAGGGAATTCAACCTGATGTATGATGGCACCAAGGAGGTGCCCATGAACCCAGTGAAGATATATCAAGTGTGTGACATCCCGCAGCCCCAGGGCTCAATCATTAATCCA------GGATCCACTGGATCTGCTCCTTGGGATGAGAAGGATAATGATGTGGATGAAGACGATGAGGAAGATGAGCTTGATCAGTCACAGCATCACGTTCCCATCCAGGACACCTTCCCCTTCCTGAACATCAATGGTTCCCCCATGGCACCAGCCAGCGTGGGCAATTGCAGTGTAGGCAACTGCAGTGTGGGCAACTGCAGTCCTGAAGCTGTGTGGCCCAAAACGGAACCTCTGGAGATGGAAGTACCCCAGGCACCTATACAG---CCCTTCTTTAGCTCTCCAGAGCTGTGGATCAGCTCTCTTCCGATGACTGACCTGGACATCAAGTTTCAGTACCGTGGGAAGGAGTACGGGCAGACTATGACCGTGAGCAACCCCCAGGGCTGCCGGCTCTTCTATGGGGACCTAGGTCCCATGCCTGACCAGGAGGAGCTCTTTGGTCCTGTCAGCCTGGAACAGGTCAAGTTTCCAGGTCCAGAGCACATCACCAATGAAAAGCAGAAGCTGTTCACTAGCAAGCTGCTAGACGTCATGGACAGAGGACTGATCCTGGAGGTCAGCGGTCACGCCATTTATGCCATCAGGCTGTGCCAGTGCAAGGTGTACTGGTCTGGACCATGCGCCCCATCGCTTGTTGCCCCCAACCTGATTGAGAGACAAAAGAAGGTCAAACTATTTTGTCTGGAAACATTCCTTAGCGNNNNNNNNNNNNNNNNNNNNNNNNNNNNNNNNNNNNNNNNNNNNNNNNNNNNNNNNNNNNNNNNNNNNNNNNNNNNNNNNNNNNNNNNNNNNNNNNNNNNNNNNNNNNNNNNNNNNNNNGTCATTCCAGTGGTGGCTCGGATGATCTATGAGATGTTTTCTGGCGATTTCACACGATCCTTTGACAGTGGCAGTGTCCGCCTGCAGATCTCAACTCCAGACATCAAAGATAACATTGTCGCTCAGCTGAAGCAGCTGTACCGCATCCTCCAAACCCAGGAAAGCTGGCAGCCCATGCAGCCCACCCCCAATATGCAACTGCCCCCTGCCCTGCCAGCTCAGTAG---------------------------------------------------------------------------------

>Opossum_ENSMODG00000002539

ATGGGAACCGAACCTGGAGTTTCCACCCGATCAAATTCTTCCACCAAAGTCTTCCGACGGCCTGGAGCGCTGTGGTGGCGGATC------------------------------------------------------------------------------------------------------------------------------ATGGCCCTCCAACCCCGAAGAGTTCGGCTGAAGCCCTGGCTTGTGGCCCAAGTAGACAGTGGGATGTACCCTGGGCTTGTCTGGCTACACAGAGAATCTAAACGCTTCCAGATTCCTTGGAAACATGCAACCCGACATAGCCCCCAACACGAGGAGGAGAATACTATCTTTAAGGCCTGGGCTGTGGAAACTGGGAAATACCAGGAAGGGGTTGATGACCCCGACCCAGCCAAATGGAAAGCCCAACTCCGATGTGCGCTGAACAAGAGCCGAGAGTTCAACCTGATGTATGATGGCACCAAGGAGGTGCCCATGAACCCGGTGAAGATCTATGAAGTCTGTGATATCCCCCAGCCCCAGGGCTCAATCATTAACCCA------GGGTCCACAGGATCTGCTCCATGGGATGAGAAGGATAATGAACTAGATGAGGAA---GAGGAAGATGAACTAGATCAATCTCAGCCCCATATCCCCATCCAGGAAGCCTTCCCTTTCCTGAACATCAACGGCTCTCCTATGGCGCCAGCC---------------AGTGTGGGCAATTGCAGCGTGGGAAATTGCAGCCCTGAAGCAGTGTGGCCCAAAACAGAACCCCTGGAAATGGAAGTGCCCCCAGCTCCCATGCAGCATGACTTCTTTAGTTCTCCAGAACTTTGGATCAGCTCTCTCCCAATGACTGATTTGGACATTAAATTCCAGTACCGTGGGAAGGAGCTGGGGCAGACG---ACGGTGAGCAACCCTCAGGGCTGCCGACTCTATTACGGTGATCTGGGGCCCATGCCAGACCAGGAGGAGCTTTTTGGTCCTGTTAGCCTGGAGCAGGTGAAGTTCCCTGGGCCAGATCAGATCACTAATGAAAAGCAGAAGCTGTTTACCAGCAGGTTGCTGGATGTCATGGACCGGGGACTGATCTTGGAAGTCAGTGGCCATGCCATTTATGCCATCAGATTATGCCAGTGCAAGGTATACTGGTCTGGGCCCTGTGCCCCTTCGCTGGTAGCCCCCAACTTGATCGAACGGCAAAAGAAGGTCAAGCTATTCTGCCTGGAAACTTTCCTCAGCGAACTCATTGCCCGCCAGAAAGGACAACTAGAGAAACAACCACCTTTTGAGATCTATTTGTGCTTTGGGGAAGAATGGCCTGATGGGAAACCCCGAGAGAGGAAGCTCATTGTGGTTCAGGTTATTCCAGTGGTTGCAAGGATGATCTACGAAATGTTTTCTGGAGATTTTACCCGGTCCTTTGACAGTGGCAGTGTCCGCCTGCAGATCTCAACTCCGGATATTAAAGATAATATTGTTGCCCATTTGAAGCAGCTGTATCGCCTTCTCCAGACCCAGGAAAGCTGGCCTCCCATGAACCCTCCCCCCAACATGCATATGACCCCTGCCCTGCCAGCCCAGTGA---------------------------------------------------------------------------------

>Rabbit_ENSOCUG00000004035

ATGAGGGCCACTGCCCAGTGTTATCAACTATGCCCGTGTATTTGCATGAGAATCAATTGCAGACTTTACCCAGCCTGGGACCCATTGCCTCCCTGCAGCCTCTGGGGAGATAAACAGACCCGGAGTCTCCCGGTGGCACAGAACAAGGAAAGAGGACTTGAAGCCTGGCTCTTCAGATCTCCGTGGACACATTGCCGGCTCAGCTGTATCATGGCCCTCCACCCCCGCAGAGTTCGGCTGAAGCCCTGGCTGGTGGCCCAGGTGGATAGTGGCCTGTATCCTGGGCTCATCTGGCTACACAGGGACTCCAAACGCTTCCAGATTCCCTGGAAACATGCCACAAGGCATAGCCCCCAACAAGAGGAGGAAAATACCATTTTTAAGGCTTGGGCTGTAGAGACAGGGAAGTACCAGGAAGGAGTGGATGACCCTGACCCCGCTAAATGGAAGGCCCAGCTCCGCTGTGCTCTCAACAAGAGCAGGGAATTCAACTTGATGTATGATGGCACCAAGGAGGTGCCCATGAACCCAGTGAAGATATATCAAGTGTGTGACATCCCCCAGCCCCAGGGCTCAATCATTAACCCA------GGATCTACAGGATCTGCTCCCTGGGATGAGAAAGATAATGATGTGGATGAAGATGATGAGGAAGATGAGCTTGATCAGTCACAGCACCATGTTCCCATCCAGGACACCTTCCCATTCCTGAACATTAATGGTTCTCCCATGGCACCAGCC---------------AGTGTGGGCAACTGTAGTGTAGGCAATTGCAGCCCTGAAACAGTATGGCCCAAAACGGAACCTCTGGAGATGGAAGTACCCCAGGCACCTATCCAG---CCATTTTACAGCTCTCCAGAGCTATGGATCAGCTCTCTCCCAATGACTGACCTGGACATCAAATTTCAGTATCGGGGGAAGGAGTATGGGCAGACTATGACCGTGAGCAACCCCCAGGGCTGCCGGCTCTTCTATGGGGACCTGGGCCCCATGCCTGACCAGGAGGAGCTCTTTGGTCCTGTCAGCCTGGAGCAGGTCAAATTCCCAGGTCCGGAGCATATCACCAATGAGAAGCAGAAGCTGTTCACAAGCAAACTGCTGGACGTCATGGACAGAGGACTGATCCTGGAGGTCAGCGGTCATGCCATTTATGCCATCAGGCTGTGCCAATGCAAGGTGTACTGGTCTGGGCCGTGTGCCCCATCACTTGTTGCCCCCAATCTGATCGAGAGACAAAAGAAGGTCAAGCTGTTTTGCCTGGAAACATTCCTTAGTGATCTCATTGCCCACCAGAAAGGGCAGATAGAAAAGCAGCCACCATTTGAGATCTATTTATGCTTTGGGGAAGAATGGCCAGATGGGAAACCCCAGGACAGGAAACTCATCTTGGTTCAGGTAATCCCAGTGGTGGCGCGGATGATCTATGAGATGTTTTCTGGTGACTTCACACGATCCTTTGACAGTGGCAGTGTCCGCCTGCAGATCTCAACTCCAGACATCAAAGATAACATCGTTGCTCAGCTGAAACAACTCTACCGCATTCTGCAAACCCAGGAAAGCTGGCAGCCCATGCAGCCCACCCCCAGCATGCAACTGCCCCCAGCCTGCCGGCCCAGTAATCACGAATGCCATCTTCTCCTTCTCTTTTTTATGATATTGTATATAGGGCTTTTTATTATTATTATTATTATTCAGACTTAA

>Cow_ENSBTAG00000002849

------------------------------------------------------------------------------------------------------------------------------------------------------------------------------------------------------------------ATGGCCCTCCACCCCCGCAGAGTTCGGCTGAAGCCCTGGCTGGTGGCCCAAGTGGATAGTGGTCTGTACCCAGGGCTCATCTGGCTACACAGGGACTCCAAACGCTTCCAGATCCCCTGGAAACATGCCACCCGGCACAGCCCCCAGCAAGAGGAGGAAAACACCATTTTCAAGGCCTGGGCTGTGGAGACAGGGAAGTACCAGGAAGGGGTAGATGACCCCGACCCAGCTAAATGGAAGGCCCAGCTCCGCTGTGCTCTCAACAAGAGCAGAGAATTCAACCTGATGTATGATGGCACCAAGGAGGTGCCCATGAACCCAGTGAAGATATATCAAGTGTGTGACATCCCCCAGCCCCAGGGCTCGATCATTAACCCA------GGATCCACTGGGTCTGCTCCTTGGGATGAGAAAGACAATGATGTGGATGAAGAAGATGAGGAAGATGAGCTGGACCAGTCACAGCACCACGTCCCCATTCAGGACACC---CCCTTCCTGAACATCAATGGTTCTCCCATAGCACCAGCC---------------AGTGTGGGCAACTGCAGTGTGGGCAACTGCAGCCCTGAAGCAGTGTGGCCCAAAACGGAGCCTCTGGAGATGGAAGTGCCTCAGGCGCCTATACAG---CCTTTCTATAGCTCTCCAGAGCTGTGGATCAGCTCACTCCCAATGACTGACCTGGACATCAAGTTTCAGTACCGTGGGAAGGAATATGGGCAGACCATGACCGTGAGCAACCCCCAAGGCTGCCGGCTCTTCTATGGGGACCTGGGTCCCATGCCTGACCAGGAGGAGCTCTTTGGTCCCGTCAGCCTGGAGCAGGTCAAGTTCCCGGGGCCAGAGCACATCACCAATGAGAAGCAGAAGCTGTTCACCAGCAAGCTGCTAGACGTCATGGATAGAGGACTGATCCTGGAGGTCAGCGGGCACGCCATCTACGCCATCAGGCTGTGCCAGTGCAAGGTGTACTGGTCTGGGCCATGTGCCCCATCACTTGTTGCCCCCAACCTGATTGAGAGACAAAAGAAGGTCAAACTGTTTTGTCTGGAAACGTTCCTGAGTGATCTCATTGCCCACCAGAAAGGACAGATAGAGAAGCAGCCACCATTTGAAATCTACTTATGCTTCGGGGAAGAATGGCCAGATGGGAAACCCCAGGAAAGGAAACTCATCTTGGTTCAGGTCATTCCGGTGGTGGCTCGGATGATCTATGAGATGTTTTCTGGTGACTTCACACGATCCTTTGACAGTGGCAGTGTCCGCCTGCAGATCTCAACTCCAGACATCAAGGATAACATCGTTGCTCAGCTGAAGCAGCTGTACCGCATCCTGCAAACCCAGGAAAGCTGGCAGCCTATGCAGCCCGCCCCCAGCATGCAATTGCCCACTACCCTGCCAGCCCAGTAA---------------------------------------------------------------------------------

>Elephant_ENSLAFG00000001374

------------------------------------------------------------------------------------------------------------------------------------------------------------------------------------------------------------------ATGGCCCTCCACCCCCGTAGAGTCCGGCTGAAGCCCTGGCTGGTGGCCCAGGTGGACAGTGGCCTCTACCCTGGGCTCATCTGGCTGCACAGGGATGCCAAACGCTTCCAGATTCCCTGGAAACATGCCACCCGGCATAGTCCCCAACAGGAGGAAGAAAATACCATTTTCAAGGCCTGGGCAGTGGAGACGGGAAAGTACCAGGAAGGGGTGGATGATCCTGACCCAGCTAAATGGAAGGCCCCGCTTCGCTGTGCTCTCAACAAGAGCAGAGAATTCACCCTGATGTACGATGGCACCAAGGAGGTGCCTATGAACCCAGTGAAGATCTATCAAGTGTGTGACATCCCCCAGCCCCAGGGCTCGATCATTAACCCAGGGGTAGGATCCACAGGGTCTGCTCCCTGGGATGAGAAGGATAATGATGTAGATGAAGAGGAGGAGGAAGATGAGCTAGATCAGTCACAGCATCATGTTCCTATCCAGGACACCTTCCCCTTCCTGAACATCAATGGCTCTCCCATGGCACCAGCCAGCGTGGGCAACTGCAGTGTAGGCAATTGCAGTGTGGGCAACTGCAGCCCTGAAGCAGTGTGGCCCAAAAGTGAACCTCTGGAAATGGAAGTACCCCAGACACCTATCCAG---CCCTTCTATAGCTCTCCAGAGCTGTGGATCAGCTCTCTCCCAATGACTGACCTGGACATCAAATTTCAGTACCGTGGGAAGGAGTACGGGCAGACCATGACTGTGAGCAACCCCCAGGGCTGCCGGCTTTTCTACGGGGACCTGGGTCCCATGCCTGACCAGGAGGAGCTCTTTGGTCCTGTCAGCCTAGAGCAGGTCAAGTTCCCAGGTCCGGAACATATCACCAATGAGAAGCAGAAGCTTTTCACCAGCAAGCTGCTGGATGTCATGGACAGAGGACTGATCCTGGAGGTCAGCGGTCATGCCATTTATGCAATCAGGCTGTGCCAGTGCAAAGTGTACTGGTCTGGGCCATGTGCCCCATCGCTTGTCGCCCCCAACTTGATTGAGAGACAAAAGAAGGTCAAGTTATTTTGCCTGGAAACATTCCTTAGTGACCTTATTGCCCATCAGAAAGGACAGATAGAGAAGCAGCCACCATTTGAGATCTACTTATGCTTTGGGGAAGAATGGCCAGATGGGAAGCCTCTGGAAAGGAAACTCATCTTGGTTCAGGTTATTCCGGTGGTGGCTCGGATGATCTACGAGATGTTTTCTGGTGATTTCACACGATCCTTTGATAGTGGCAGTGTTCGCCTGCAGATCTCAACTCCAGACATCAAAGATAACATCGTCGCTCAGTTAAAGCAGCTGTACCGCATCCTGCAAACCCAGGAAAGCTGGCAGCCCATGCAGCCCACCCCTAGCATGCAACTGCCCACTGCCCTGCCAGCCCAGTAA---------------------------------------------------------------------------------

>Dog_ENSCAFG00845002898

------------------------------------------------------------------------------------------------------------------------------------------------------------------------------------------------------------------ATGGCCCTCCACCCCCGCAGAGTTCGGCTGAAGCCCTGGCTGGTGGCCCAGGTGGATAGTGGCCTGTACCCTGGGCTCATCTGGCTACACAGGGACTCCAAACGCTTCCAGATTCCCTGGAAACATGCCACCCGGCATAGCCCCCAACAAGAGGAAGAAAATACCATTTTCAAGGCGTGGGCTGTGGAGACAGGGAAGTACCAGGAAGGGGTGGATGATCCTGACCCAGCTAAATGGAAGGCCCAGCTTCGCTGTGCTCTCAACAAGAGCAGGGAATTCAACCTGATGTACGATGGCACCAAGGAAGTACCCATGAACCCAGTGAAGATATATCAAGTGTGTGACATCCCCCAGCCTCAGGGGTCGATCATTAACCCA------GGATCCACTGGATCTGCTCCTTGGGATGAGAAGGATAATGATGTAGATGAAGAAGATGAAGAAGATGAGCTCGATCAGTCACAACACCATGTTCCCATCCAGGACACCTTCCCCTTCCTGAACATCAATGGTTCTCCCATGGCACCAGCC------------------------------AGTGTGGGTAACTGCAGCCCTGAAGCAGTGTGGCCCAAAACTGAACCTCTGGAAATAGATGTACCCCAGACACCTATACAG---CCCTTTTATAGCTCTCCAGAGCTGTGGATCAGCTCTCTCCCAATGACTGACCTGGACATCAAGTTTCAGTACCGTGGGAAGGAATACGGGCAAACCATGACTGTGAGCAACCCCCAGGGCTGCCGGCTCTTCTATGGCGACCTGGGTCCCATGCCTGACCAAGAAGAGCTCTTTGGTCCTGTCAGCCTGGAACAGGTCAAGTTCCCAGGGCCAGAGCATATCACCAATGAGAAGCAGAAGCTGTTCACCAGTAAGCTGCTAGATGTCATGGACAGAGGACTGATCCTGGAGGTCAGCGGTCATGCAATTTATGCCATCAGGCTGTGCCAGTGCAAGGTGTACTGGTCTGGGCCATGTGCCCCATCACTTGTTGCCCCCAACCTGATTGAGAGGCAAAAGAAGGTCAAACTATTTTGTCTGGAAACATTCCTTAGTGATCTCATTGCCCACCAGAAAGGACAGATAGAGAAGCAGCCACCATTTGAGATCTACTTATGCTTTGGGGAAGAATGGCCAGATGGGAAACCCCTGGAAAGGAAACTCATCTTGGTTCAGGTCATTCCAGTGGTAGCTCGGATGATCTACGAGATGTTTTCTGGTGATTTTACACGGTCCTTTGACAGTGGCAGTGTCCGCCTGCAGATCTCAACTCCAGATATCAAAGATAACATCGTTGCTCAACTGAAGCAGCTGTACCGCATCCTCCAAACCCAAGAAAGCTGGCAGCCCATGCAGCCCACCCCCAGCATGCAACTACCCTCTGCTCTGCCAGCCCAGTAA---------------------------------------------------------------------------------

>Tarsier_ENSTSYG00000010982

------------------------------------------------------------------------------------------------------------------------------------------------------------------------------------------------------------------ATGGCCCTCCACCCCCGCAGAGTCCGGCTAAAGCCCTGGCTGGTGGCCCAAGTGGATAGTGGCCTGTACCCGGGACTCATCTGGCTACACAGGGACTCCAAACGCTTCCAGATTCCCTGGAAACATGCCACCCGGCATAGCCCCCAACAAGAGGAGGAAAATACCATTTTTAAGGCCTGGGCTGTAGAGACGGGAAAGTACCAGGAAGGAGTAGATGACCCTGACCCAGCTAAATGGAAGGCCCAGCTACGCTGTGCTCTCAATAAGAGCAGGGAATTCAACCTAATGTATGATGGCACAAAGGAGGTGCCCATGAACCCAGTGAAGATATATCAAGTGTGTGACATACCCCAGCCCCAAGGCTCAATCATTAACCCA------GGATCCACAGGGTCTGCTCCTTGGGATGAGAAGGATAATGATGTGGATGAAGAAGATGAGGAAGATGAGCTTGATCAGTCGCAGCACCATGTTCCCATCCAGGACACCTTCCCCTTCCTGAACATCAATGGTTCTCCCATGGCACCAGCC---------------AGTGTGGGCAACTGCAGTGTGGGCAACTGCAGCCCTGAGGCAGTGTGGCCCAAAACTGAACCCCTGGAGATGGAAGTACCTCAGGCACCTATACAG---CCCTTTTACAGTTCTCCAGAGCTGTGGATCAGCTCTCTCCCAATGACTGATCTGGACATCAAGTTTCAGTACCGTGGGAAGGAGTACGGACAGACCATGACTGTAAGCAACCCTCAGGGCTGCCGGCTCTTCTATGGGGACCTGGGTCCCATGCCTGACCAGGAGGAGCTCTTTGGTCCCGTCAGCCTGGAGCAGGTCAAATTCCCAGGTCCAGAGCATATCACCAATGAGAAGCAGAAGCTGTTCACCAGCAAGCTGCTGGACGTCATGGACAGAGGACTGATCCTGGAGGTCAGCGGTCACGCCATTTATGCCATAAGGCTATGCCAGTGCAAGGTGTACTGGTCTGGGCCATGTGCCCCATCACTTGTTGCCCCCAACCTGATTGAGAGACAAAAGAAGGTCAAGCTATTTTGTCTGGAAACATTCCTTAGTGATCTCATTGCCCACCAAAAAGGACAGATAGAGAAGCAGCCACCGTTTGAGATTTACTTATGCTTTGGGGAAGAATGGCCAGATGGGAAACCCTTGGAAAGGAAACTCATCTTGGTTCAGGTCATTCCGGTGGTGGCGCGGATGATCTATGAGATGTTTTCTGGTGATTTCACACGATCCTTTGACAGTGGCAGTGTTCGCCTACAGATCTCAACTCCAGACATCAAAGATAACATCGTTGCTCAGCTGAAGCAGCTGTACCGGATTCTTCAAACCCAGGAAAGCTGGCAGCCTATGCAGTCTACCCCTAACATGCAACTGCCCCCTGCCCTGCCGGCCCAGTAA---------------------------------------------------------------------------------

>Horse_ENSECAG00000007482

------------------------------------------------------------------------------------------------------------------------------------------------------------------------------------------------------------------ATGGCCCTCCACCCCCGCAGAGTTCGGCTGAAGCCCTGGCTGGTGGCCCAGGTGGATAGTGGCCTGTACCCTGGGCTCATCTGGCTACACAGGGACTCCAAACGCTTCCAGATTCCCTGGAAGCATGCCACCCGGCACAGCCCCCAACAAGAGGAGGAAAATACCATTTTCAAGGCCTGGGCTGTGGAGACAGGGAAGTACCAGGAAGGGGTGGATGACCCTGACCCAGCTAAATGGAAGGCCCAGCTCCGTTGTGCTCTCAACAAGAGCAGGGAATTCAACCTGATGTACGATGGCACCAAGGAGGTGCCCATGAACCCAGTGAAGATATATCAAGTGTGTGATATCCCCCAGCCTCAGGGGTCGATCATTAACCCA------GGATCCACTGGGTCTGCTCCTTGGGATGAGAAGGATAATGATGTGGATGAAGAAGATGAGGAAGATGAGCTTGATCAGTCACAGCACCATGTTCCCATCCAGGACACCTTCCCCTTCTTGAACATCAATGGTTCTCCCATGGCACCAGCC---------------AGTGTGGGCAACTGCAGTGTGGGCAACTGCAGCCCTGAAGCAGTGTGGCCCAAAACTGAACCCCTGGAGATGGAAGTACCCCAGACACCTATACAG---CCCTTCTATAGCTCTCCAGAGCTGTGGATCAGCTCTCTCCCAATGACTGACCTGGACATCAAGTTTCAGTATCGTGGGAAGGAGTATGGGCAGACCATGACTGTGAGCAACCCTCAGGGCTGCCGGCTCTTCTATGGGGACCTAGGTCCCATGCCTGACCAGGAGGAGCTCTTTGGTCCCGTCAGCCTGGAGCAGGTCAAGTTCCCAGGTCCAGAGCATATCACCAATGAGAAGCAGAAGCTGTTCACCAGCAAGCTGCTAGATGTCATGGACAGAGGACTGATCCTGGAGGTCAGCGGTCACGCCATTTATGCCATCAGGCTGTGCCAGTGCAAGGTGTATTGGTCTGGGCCATGTGCCCCATCGCTTGTTGCCCCCAACCTGATTGAGAGACAAAAGAAGGTCAAACTATTCTGTCTGGAAACATTCCTTAGTGATCTCATTGCCCACCAGAAAGGACAGATAGAGAAGCAGCCACCATTTGAGATCTACTTATGCTTTGGAGAAGAATGGCCAGATGGGAAACCCCTGGAAAGGAAACTCATCTTGGTTCAGGTCATTCCAGTGGTGGCTCGGATGATCTACGAGATGTTTTCTGGCGATTTCACACGATCCTTTGACAGTGGCAGTGTCCGCCTGCAGATCTCAACTCCAGACATCAAAGATAACATTGTTGCCCAGCTGAAGCAACTGTACCGCATCCTCCAAACCCAGGAAAGCTGGCAGCCCATGCAGCCCACCCCCAGCATGCAGCTGCCCCCTGCCCTGCCGGCCCAGTAG---------------------------------------------------------------------------------

>Human_ENSG00000117595

------------------------------------------------------------------------------------------------------------------------------------------------------------------------------------------------------------------ATGGCCCTCCACCCCCGCAGAGTCCGGCTAAAGCCCTGGCTGGTGGCCCAGGTGGATAGTGGCCTCTACCCTGGGCTCATCTGGCTACACAGGGACTCTAAACGCTTCCAGATTCCCTGGAAACATGCCACCCGGCATAGCCCTCAACAAGAAGAGGAAAATACCATTTTTAAGGCCTGGGCTGTAGAGACAGGGAAGTACCAGGAAGGGGTGGATGACCCTGACCCAGCTAAATGGAAGGCCCAGCTGCGCTGTGCTCTCAATAAGAGCAGAGAATTCAACCTGATGTATGATGGCACCAAGGAGGTGCCCATGAACCCAGTGAAGATATATCAAGTGTGTGACATCCCTCAGCCCCAGGGCTCGATCATTAACCCA------GGATCCACAGGGTCTGCTCCCTGGGATGAGAAGGATAATGATGTGGATGAAGAAGATGAGGAAGATGAGCTGGATCAGTCGCAGCACCATGTTCCCATCCAGGACACCTTCCCCTTCCTGAACATCAATGGTTCTCCCATGGCGCCAGCC---------------AGTGTGGGCAATTGCAGTGTGGGCAACTGCAGCCCGGAGGCAGTGTGGCCCAAAACTGAACCCCTGGAGATGGAAGTACCCCAGGCACCTATACAG---CCCTTCTATAGCTCTCCAGAACTGTGGATCAGCTCTCTCCCAATGACTGACCTGGACATCAAGTTTCAGTACCGTGGGAAGGAGTACGGGCAGACCATGACCGTGAGCAACCCTCAGGGCTGCCGACTCTTCTATGGGGACCTGGGTCCCATGCCTGACCAGGAGGAGCTCTTTGGTCCCGTCAGCCTGGAGCAGGTCAAATTCCCAGGTCCTGAGCATATTACCAATGAGAAGCAGAAGCTGTTCACTAGCAAGCTGCTGGACGTCATGGACAGAGGACTGATCCTGGAGGTCAGCGGTCATGCCATTTATGCCATCAGGCTGTGCCAGTGCAAGGTGTACTGGTCTGGGCCATGTGCCCCATCACTTGTTGCTCCCAACCTGATTGAGAGACAAAAGAAGGTCAAGCTATTTTGTCTGGAAACATTCCTTAGCGATCTCATTGCCCACCAGAAAGGACAGATAGAGAAGCAGCCACCGTTTGAGATCTACTTATGCTTTGGGGAAGAATGGCCAGATGGGAAACCATTGGAAAGGAAACTCATCTTGGTTCAGGTCATTCCAGTAGTGGCTCGGATGATCTACGAGATGTTTTCTGGTGATTTCACACGATCCTTTGATAGTGGCAGTGTCCGCCTGCAGATCTCAACCCCAGACATCAAGGATAACATCGTTGCTCAGCTGAAGCAGCTGTACCGCATCCTTCAAACCCAGGAGAGCTGGCAGCCCATGCAGCCCACCCCCAGCATGCAACTGCCCCCTGCCCTGCCTCCCCAGTAA---------------------------------------------------------------------------------

>Gorilla_ENSGGOG00000006214

------------------------------------------------------------------------------------------------------------------------------------------------------------------------------------------------------------------ATGGCCCTCCACCCCCGCAGAGTCCGGCTAAAGCCCTGGCTGGTGGCCCAGGTGGATAGTGGCCTCTACCCTGGGCTCATCTGGCTACACAGGGACTCTAAACGCTTCCAGATTCCCTGGAAACATGCCACCCGGCATAGCCCTCAACAAGAAGAGGAAAATACCATTTTTAAGGCCTGGGCTGTAGAGACAGGGAAGTACCAGGAAGGGGTGGATGACCCTGACCCAGCTAAATGGAAGGCCCAGCTGCGCTGTGCTCTCAATAAGAGCAGAGAATTCAACTTGATGTATGATGGCACCAAGGAGGTGCCCATGAACCCAGTGAAGATATATCAAGTGTGTGACATCCCTCAGCCCCAGGGCTCGATCATTAACCCA------GGATCCACAGGGTCTGCTCCCTGGGATGAGAAGGATAATGATGTGGATGAAGAAGATGAGGAAGATGAGCTGGATCAGTCGCAGCACCATGTTCCCATCCAGGACACCTTCCCCTTCCTGAACATCAATGGTTCTCCCATGGCGCCAGCC---------------AGTGTGGGCAATTGCAGTGTGGGCAACTGCAGCCCGGAGGCAGTGTGGCCCAAAACTGAACCCCTGGAGATGGAAGTACCCCAGGCACCTATACAG---CCCTTCTATAGCTCTCCAGAACTGTGGATCAGCTCTCTCCCAATGACTGACCTGGACATCAAGTTTCAGTACCGTGGGAAGGAGTACGGGCAGACCATGACCGTGAGCAACCCCCAGGGCTGCCGACTCTTCTATGGGGACCTGGGTCCCATGCCTGACCAGGAGGAGCTCTTTGGTCCCGTCAGCCTGGAGCAGGTCAAATTCCCAGGTCCTGAGCATATTACCAATGAGAAGCAGAAGCTGTTCACTAGCAAGCTGCTGGACGTCATGGACAGAGGACTGATCCTGGAGGTCAGCGGTCATGCCATTTATGCCATCAGGCTGTGCCAGTGCAAGGTGTACTGGTCTGGGCCATGTGCCCCATCACTTGTTGCTCCCAACCTGATTGAGAGACAAAAGAAGGTCAAGCTATTTTGTCTGGAAACATTCCTTAGCGATCTCATTGCCCACCAGAAAGGACAGATAGAGAAGCAGCCACCGTTTGAGATCTACTTATGCTTTGGGGAAGAATGGCCAGATGGGAAACCACTGGAAAGGAAACTCATCTTGGTTCAGGTCATTCCAGTAGTGGCTCGGATGATCTACGAGATGTTTTCTGGTGATTTCACACGATCCTTTGATAGTGGCAGTGTCCGCCTGCAGATCTCAACCCCAGACATCAAGGATAACATCGTTGCTCAGCTGAAGCAGCTGTACCGCATCCTTCAAACCCAGGAGAGCTGGCAGCCCATGCAGCCCACCCCCAGCATGCAACTGCCCCCTGCCCTGCCTCCCCAGTAA---------------------------------------------------------------------------------

>Macaca_ENSMMUG00000022065

------------------------------------------------------------------------------------------------------------------------------------------------------------------------------------------------------------------ATGGCCCTCCACCCCCGCAGAGTCCGACTGAAGCCCTGGCTGGTGGCCCAGGTGGATAGTGGCCTCTACCCTGGGCTCATCTGGCTACACAGGGACTCTAAACGCTTCCAGATTCCCTGGAAACATGCCACCCGGCATAGCCCTCAACAAGAAGAGGAAAATACCATTTTTAAGGCCTGGGCTGTAGAGACAGGGAAGTACCAGGAAGGGGTGGATGACCCTGATCCAGCTAAATGGAAGGCCCAGCTGCGCTGTGCTCTCAATAAGAGCAGAGAATTCAACCTAATGTATGATGGCACCAAGGAGGTGCCCATGAACCCAGTGAAGATATATCAAGTGTGTGACATCCCTCAGCCCCAGGGCTCGATCATTAACCCA------GGATCCACAGGGTCTGCTCCCTGGGATGAGAAGGATAATGATGTGGATGAAGAAGATGAGGAAGATGAGCTGGATCAGTCGCAGCACCATGTTCCCATCCAGGACACCTTCCCCTTCCTGAACATCAATGGTTCTCCCATGGCGCCAGCC---------------AGTGTGGGTAATTGCAGTGTGGGCAACTGCAGCCCTGAGGCAGTGTGGCCCAAAACTGAACCCCTGGAGATGGAAGTACCCCAGGCACCTATACAG---CCCTTCTATAGCTCTCCAGAACTGTGGATCAGCTCTCTCCCAATGACTGACCTGGACATCAAGTTTCAGTACCGTGGGAAGGAGTACGGGCAGACCATGACCGTGAGCAACCCCCAGGGCTGCCGACTCTTCTATGGGGACCTGGGTCCCATGCCTGACCAGGAGGAGCTCTTTGGTCCTGTCAGCCTGGAGCAGGTCAAATTCCCAGGTCCTGAGCATATTACCAATGAGAAGCAGAAGCTCTTCACTAGCAAGCTGCTGGACGTCATGGACAGAGGACTGATCCTGGAGGTCAGCGGTCACGCCATTTATGCCATCAGGCTGTGCCAGTGCAAGGTGTACTGGTCTGGGCCATGTGCCCCATCACTTGTTGCTCCCAACCTGATTGAGAGACAAAAGAAGGTCAAGCTATTTTGTCTGGAAACATTCCTTAGCGATCTCATTGCCCACCAGAAAGGACAGATAGAAAAGCAGCCACCGTTTGAGATCTACTTATGCTTTGGGGAAGAATGGCCAGATGGGAAACCCTTGGAAAGGAAACTCATCTTGGTTCAGGTCATTCCAGTAGTAGCTCGGATGATCTACGAGATGTTTTCTGGTGATTTCACACGATCCTTTGATAGTGGCAGTGTCCGCCTCCAGATCTCAACCCCAGACATCAAAGATAACATCGTTGCTCAGCTGAAGCAGCTGTACCGCATCCTTCAAACCCAGGAGAGCTGGCAGCCCATGCAGCCCACCCCCAGCATGCAACTCCCCCCTGCCCTGCCTCCTCAATAA---------------------------------------------------------------------------------

>Gibbon_ENSNLEG00000001962

------------------------------------------------------------------------------------------------------------------------------------------------------------------------------------------------------------------ATGGCCCTCCACCCCCGCAGAGTCCGGCTAAAGCCCTGGCTGGTGGCCCAGGTGGATAGTGGCCTCTACCCTGGGCTCATCTGGCTACACAGGGACTCTAAACGCTTCCAGATTCCCTGGAAACATGCCACCCGGCATAGCCCTCAACAAGAAGAGGAAAATACCATTTTTAAGGCCTGGGCTGTAGAGACAGGGAAGTACCAGGAAGGGGTGGATGACCCTGACCCAGCTAAATGGAAGGCCCAGCTACGCTGTGCTCTCAATAAGAGCAGAGAATTCAACCTGATGTATGATGGCACCAAGGAGGTGCCCATGAACCCAGTGAAGATATATCAAGTGTGTGACATCCCTCAGCCCCAGGGCTCGATCATTAACCCA------GGATCCACAGGGTCTGCTCCCTGGGATGAGAAGGATAATGATGTGGATGAAGAAGATGAGGAAGATGAGCTGGATCAGTCGCAGCACCATGTTCCCATCCAGGACACCTTCCCCTTCCTGAACATCAATGGTTCTCCCATGGCGCCAGCC---------------AGTGTGGGCAATTGCAGTGTGGGCAACTGCAGCCCGGAGGCAGTGTGGCCCAAAACTGAACCCCTGGAGATGGAAGTACCCCAGGCACCTATACAG---CCCTTCTATAGCTCTCCAGAACTGTGGATCAGCTCTCTCCCAATGACTGACCTGGACATCAAGTTTCAGTACCGTGGGAAGGAGTATGGGCAGACCATGACCGTGAGCAACCCCCAGGGCTGCCGACTCTTCTATGGGGACCTGGGTCCCATGCCTGACCAGGAGGAGCTCTTTGGTCCTGTCAGCCTGGAGCAGGTCAAATTCCCAGGTCCTGAGCATATTACCAATGAGAAGCAGAAGCTGTTCACTAGCAAGCTGCTGGACGTCATGGACAGAGGACTGATCCTGGAGGTCAGCGGTCATGCCATTTATGCCATCAGGCTGTGCCAGTGCAAGGTGTACTGGTCTGGGCCATGTGCCCCATCACTTGTTGCTCCCAACCTGATTGAGAGACAAAAGAAGGTCAAGCTATTTTGTCTGGAAACATTCCTTAGCGATCTCATTGCCCACCAGAAAGGACAGATAGAGAAGCAGCCACCATTTGAGATCTACTTATGCTTTGGGGAAGAATGGCCAGATGGGAAACCCTTGGAAAGGAAACTCATCTTGGTTCAGGTCATTCCAGTAGTGGCTCGGATGATCTACGAGATGTTTTCTGGTGATTTCACACGATCCTTTGATAGTGGCAGTGTCCGCCTGCAGATCTCAACCCCAGACATCAAGGATAACATCGTTGCTCAGCTGAAGCAGCTGTACCGCATCCTTCAAACCCAGGAGAGCTGGCAGCCCATGCAGCCCACCCCCAGCATGCAACTGCCCCCTGCCCTGCCTCCCCAGTAA---------------------------------------------------------------------------------

>Bonobo_ENSPPAG00000032756

------------------------------------------------------------------------------------------------------------------------------------------------------------------------------------------------------------------ATGGCCCTCCACCCCCGCAGAGTCCGGCTAAAGCCCTGGCTGGTGGCCCAGGTGGATAGTGGCCTCTACCCTGGGCTCATCTGGCTACACAGGGACTCTAAACGCTTCCAGATTCCCTGGAAACATGCCACCCGGCATAGCCCTCAACAAGAAGAGGAAAATACCATTTTTAAGGCCTGGGCTGTAGAGACAGGGAAGTACCAGGAAGGGGTGGATGACCCTGACCCAGCTAAATGGAAGGCCCAGCTGCGCTGTGCTCTCAATAAGAGCAGAGAATTCAACCTGATGTATGATGGCACCAAGGAGGTGCCCATGAACCCAGTGAAGATATATCAAGTGTGTGACATCCCTCAGCCCCAGGGCTCAATCATTAACCCA------GGATCCACAGGGTCTGCTCCCTGGGATGAGAAGGATAATGATGTGGATGAAGAAGATGAGGAAGATGAGCTGGATCAGTCGCAGCACCATGTTCCCATCCAGGACACCTTCCCCTTCCTGAACATCAATGGTTCTCCCATGGCGCCAGCC---------------AGTGTGGGCAATTGCAGTGTGGGCAACTGCAGCCCGGAGGCAGTGTGGCCCAAAACTGAACCCCTGGAGATGGAAGTACCCCAGGCACCTATACAG---CCCTTCTATAGCTCTCCAGAACTGTGGATCAGCTCTCTCCCAATGACTGACCTGGACATCAAGTTTCAGTACCGTGGGAAGGAGTACGGGCAGACCATGACCGTGAGCAACCCCCAGGGCTGCCGACTCTTCTATGGGGACCTGGGTCCCATGCCTGACCAGGAGGAGCTCTTTGGTCCAGTCAGCCTGGAGCAGGTCAAATTCCCGGGTCCTGAGCATATTACCAATGAGAAGCAGAAGCTGTTCACTAGCAAGCTGCTGGACGTCATGGACAGAGGACTGATCCTGGAGGTCAGCGGTCATGCCATTTATGCCATCAGGCTGTGCCAGTGCAAGGTGTACTGGTCTGGGCCATGTGCCCCATCACTTGTTGCTCCCAACCTGATTGAGAGACAAAAGAAGGTCAAGCTATTTTGTCTGGAAACATTCCTTAGCGATCTCATTGCCCACCAGAAAGGACAGATAGAGAAGCAGCCACCGTTTGAGATCTACTTATGCTTTGGGGAAGAATGGCCAGATGGGAAACCATTGGAAAGGAAACTCATCTTGGTTCAGGTCATTCCAGTAGTGGCTCGGATGATCTACGAGATGTTTTCTGGTGATTTCACACGATCCTTTGATAGTGGCAGTGTCCGCCTGCAGATCTCAACCCCAGACATCAAGGATAACATCGTTGCTCAGCTGAAGCAGCTGTACCGCATCCTTCAAACCCAGGAGAGCTGGCAGCCCATGCAGCCCACCCCCAGCATGCAACTGCCCCCTGCCCTGCCTCCCCAGTAA---------------------------------------------------------------------------------

>Chimpanzee_ENSPTRG00000001941

------------------------------------------------------------------------------------------------------------------------------------------------------------------------------------------------------------------ATGGCCCTCCACCCCCGCAGAGTCCGGCTAAAGCCCTGGCTGGTGGCCCAGGTGGATAGTGGCCTCTACCCTGGGCTCATCTGGCTACACAGGGACTCTAAACGCTTCCAGATTCCCTGGAAACATGCCACCCGGCATAGCCCTCAACAAGAAGAGGAAAATACCATTTTTAAGGCCTGGGCTGTAGAGACAGGGAAGTACCAGGAAGGGGTGGATGACCCTGACCCAGCTAAATGGAAGGCCCAGCTGCGCTGTGCTCTCAATAAGAGCAGAGAATTCAACCTGATGTATGATGGCACCAAGGAGGTGCCCATGAACCCAGTGAAGATATATCAAGTGTGTGACATCCCTCAGCCCCAGGGCTCAATCATTAACCCA------GGATCCACAGGGTCTGCTCCCTGGGATGAGAAGGATAATGATGTGGATGAAGAAGATGAGGAAGATGAGCTGGATCAGTCGCAGCACCATGTTCCCATCCAGGACACCTTCCCCTTCCTGAACATCAATGGTTCTCCCATGGCACCAGCC---------------AGTGTGGGCAATTGCAGTGTGGGCAACTGCAGCCCGGAGGCAGTGTGGCCCAAAACTGAACCCCTGGAGATGGAAGTACCCCAGGCACCTATACAG---CCCTTCTATAGCTCTCCAGAACTGTGGATCAGCTCTCTCCCAATGACTGACCTGGACATCAAGTTTCAGTACCGTGGGAAGGAGTACGGGCAGACCATGACCGTGAGCAACCCCCAGGGCTGCCGACTCTTCTATGGGGACCTGGGTCCCATGCCTGACCAGGAGGAGCTCTTTGGTCCAGTCAGCCTGGAGCAGGTCAAATTCCCGGGTCCTGAGCATATTACCAATGAGAAGCAGAAGCTGTTCACTAGCAAGCTGCTGGACGTCATGGACAGAGGACTGATCCTGGAGGTCAGCGGTCATGCCATTTATGCCATCAGGCTGTGCCAGTGCAAGGTGTACTGGTCTGGGCCATGTGCCCCATCACTTGTTGCTCCCAACCTGATTGAGAGACAAAAGAAGGTCAAGCTATTTTGTCTGGAAACATTCCTTAGCGATCTCATTGCCCACCAGAAAGGACAGATAGAGAAGCAGCCACCGTTTGAGATCTACTTATGCTTTGGGGAAGAATGGCCAGATGGGAAACCACTGGAAAGGAAACTCATCTTGGTTCAGGTCATTCCAGTAGTGGCTCGGATGATCTACGAGATGTTTTCTGGTGATTTCACACGATCCTTTGATAGTGGCAGTGTCCGCCTGCAGATCTCAACCCCAGACATCAAGGATAACATCGTTGCTCAGCTGAAGCAGCTGTACCGCATCCTTCAAACCCAGGAGAGCTGGCAGCCCATGCAGCCCACCCCCAGCATGCAACTGCCCCCTGCCCTGCCTCCCCAGTAA---------------------------------------------------------------------------------

>GoldenSnubNoseMonkey_ENSRROG00000033255

------------------------------------------------------------------------------------------------------------------------------------------------------------------------------------------------------------------ATGGCCCTCCACCCCCGCAGAGTCCGACTGAAGCCCTGGCTGGTGGCCCAGGTGGATAGTGGCCTCTACCCTGGGCTCATCTGGCTACACAGGGACTCTAAACGCTTCCAGATTCCCTGGAAACATGCCACCCGGCATAGCCCTCAACAAGAAGAGGAAAATACTATTTTTAAGGCCTGGGCTGTAGAGACAGGGAAGTACCAGGAAGGGGTGGATGACCCTGATCCAGCTAAATGGAAGGCCCAGCTGCGCTGTGCTCTCAATAAGAGCAGAGAATTCAACCTAATGTATGATGGCACCAAGGAGGTGCCCATGAACCCAGTGAAGATATATCAAGTGTGTGACATCCCTCAGCCCCAGGGCTCGATCATTAACCCA------GGATCCACAGGGTCTGCTCCCTGGGATGAGAAGGATAATGATGTGGATGAAGAAGATGAGGAAGATGAGCTGGATCAGTCGCAGCACCATGTTCCCATCCAGGACACCTTCCCCTTCCTGAACATCAATGGTTCTCCCATGGCGCCAGCC---------------AGTGTGGGCAATTGCAGTGTGGGCAACTGCAGCCCTGAGGCAGTGTGGCCCAAAACTGAACCCCTGGAGATGGAAGTACCCCAGGCACCTATACAG---CCCTTCTATAGCTCTCCAGAACTGTGGATCAGCTCTCTCCCAATGACTGACCTGGACATCAAGTTTCAGTACCGTGGGAAGGAGTACGGGCAGACCATGACCGTGAGCAACCCCCAGGGCTGCCGACTCTTCTATGGGGACCTGGGTCCCATGCCTGACCAGGAGGAGCTCTTTGGTCCTGTCAGCCTGGAGCAGGTCAAATTCCCAGGTCCTGAGCATATTACCAATGAGAAGCAGAAGCTCTTCACTAGCAAGCTGCTGGACGTCATGGACAGAGGACTGATCCTGGAGGTCAGCGGTCACGCCATTTATGCCATCAGGCTGTGCCAGTGCAAGGTGTACTGGTCTGGGCCATGTGCCCCATCACTTGTTGCTCCCAACCTGATTGAGAGACAAAAAAAGGTCAAGCTATTTTGTCTGGAAACATTCCTTAGCGATCTCATTGCCCACCAGAAAGGACAGATAGAAAAGCAGCCACCGTTTGAGATCTACTTATGCTTTGGGGAAGAATGGCCAGATGGGAAACCCTTGGAAAGGAAACTCATCTTGGTTCAGGTCATTCCAGTAGTGGCTCGGATGATCTACGAGATGTTTTCTGGTGATTTCACACGATCCTTTGATAGTGGCAGTGTCCGCCTGCAGATCTCAACCCCAGACATCAAAGATAACATCGTTGCTCAGCTGAAGCAGCTGTACCGCATCCTTCAAACCCAGGAGAGCTGGCAGTCCATGCAGCCCACCCCCAGCATGCAACTCCCCCCTGCCCTGCCTCCTCAATAA---------------------------------------------------------------------------------

>Marmoset_ENSCJAG00000015032

------------------------------------------------------------------------------------------------------------------------------------------------------------------------------------------------------------------ATGGCCCTCCACCCCCGCAGAGTCCGGCTGAAGCCCTGGCTGGTGGCCCAGGTGGATAGTGGCCTCTACCCTGGGCTCATCTGGCTACACAGGGACTCTAAGCGCTTCCAGATTCCCTGGAAACATGCCACCCGGCATAGCCCTCAACAAGAAGAGGAAAATACCATTTTTAAGGCCTGGGCTGTAGAGACAGGGAAGTACCAGGAAGGGGTGGATGACCCTGACCCAGCTAAATGGAAGGCCCAGCTGCGCTGTGCTCTCAATAAGAGCAGAGAATTCAACCTGATGTATGATGGCACCAAGGAGGTGCCCATGAACCCAGTAAAGATATATCAAGTGTGTGACATCCCTCAGCCCCAGGGCTCGATCATTAACCCA------GGATCCACAGGGTCTGCTCCCTGGGATGAGAAGGACAATGATGTCGATGAAGAAGATGAGGAAGATGAGCTGGATCAGTCGCAGCACCACGTTCCCATCCAGGACACGTTCCCCTTCCTGAACATCAATGGTTCTCCCATGGCGCCAGCC------------------------------AGTGTGGGCAACTGCAGCCCTGAAGCAGTGTGGCCCAAAACTGAACCTCTGGAGATGGAAGTACCCCAGCCACCTATACAG---CCCTTCTATAGCTCTCCAGAACTGTGGATCAGCTCTCTCCCAATGACTGACCTGGACATCAAGTTTCAGTACCGTGGGAAGGAGTATGGGCAGACTATGACTGTGAGCAACCCCCAGGGCTGCCGACTCTTCTATGGAGATCTGGGTCCCATGCCTGACCAGGAGGAGCTCTTTGGTCCCATCAGCCTGGAGCAGGTCAAATTCCCAGGTCCTGAGCATATTACCAATGAGAAGCAGAAGCTGTTCACTAGCAAGCTGCTGGACGTCATGGACAGAGGACTGATCCTGGAGGTCAGCGGTCACGCCATTTATGCTATCAGGCTGTGCCAGTGCAAGGTGTACTGGTCTGGGCCATGTGCCCCATCACTTGTTGCTCCCAACCTGATTGAGAGACAAAAGAAGGTGAAGCTGTTTTGTCTGGAAACATTCCTCAGTGATCTCATTGCCCACCAGAAAGGACAGATAGAGAAGCAGCCACCGTTTGAGATCTACTTATGCTTTGGGGAAGAATGGCCAGATGGGAAACCCTTGGAAAGGAAACTCATCTTGGTTCAGGTGATTCCAGTAGTGGCTCGGATGATATACGAGATGTTTTCTGGTGATTTCACACGATCCTTTGATAGTGGCAGTGTCCGCCTGCAGATCTCAACCCCAGACATCAAAGATAACATCGTTGCTCAGCTGAAGCAGCTGTACCGCATCCTTCAAACCCAGGAAAGCTGGCAGCCCATGCAGCCCAACCCCAGCATGCAACTTCCCCCTGCCCTGCCGCCCCAGTGA---------------------------------------------------------------------------------

>MasNightMonkey_ENSANAG00000021723

------------------------------------------------------------------------------------------------------------------------------------------------------------------------------------------------------------------ATGGCCCTCCACCCCCGCAGAGTCCGGCTGAAGCCCTGGCTGGTGGCCCAGGTGGATAGTGGCCTCTACCCTGGGCTCATCTGGCTCCACAGGGACTCTAAGCGCTTCCAGATTCCCTGGAAACATGCCACCCGGCATAGCCCTCAACAAGAAGAGGAAAATACCATTTTTAAGGCTTGGGCCGTAGAGACAGGGAAGTACCAGGAAGGGGTGGATGACCCTGACCCAGCTAAATGGAAGGCCCAGCTGCGCTGTGCTCTCAATAAGAGCAGAGAATTCAACCTGATGTATGATGGCACCAAGGAGGTGCCCATGAACCCAGTGAAGATATATCAAGTGTGTGACATCCCTCAGCCCCAGGGCTCGATCATCAACCCA------GGATCCACAGGGTCTGCTCCCTGGGATGAGAAGGACAATGATGTCGATGAAGAAGATGAGGAAGACGAGCTGGATCAGTCACAGCACCATGTTCCCATCCAGGACACGTTCCCCTTCCTGAACATCAATGGTTCTCCCATGGCGCCAGCC------------------------------AGTGTGGGCAACTGCAGCCCTGAAGCAGTGTGGCCCAAAACTGAACCCCTGGAGATGGAAGTACCCCAGGCACCTATCCAG---CCCTTCTATAGCTCTCCAGAACTGTGGATCAGCTCTCTCCCAATGACTGACCTGGACATCAAGTTTCAGTACCGTGGGAAGGAGTACGGGCAGACTATGACCGTGAGCAACCCCCAGGGCTGCCGACTCTTCTATGGGGATCTGGGTCCCATGCCTGACCAGGAGGAGCTCTTTGGTCCCATCAGCCTGGAGCAGGTCAAATTCCCAGGTCCTGAGCATATTACCAATGAGAAGCAGAAGCTGTTCACTAGCAAGCTGCTGGACGTCATGGACAGAGGACTGATCCTGGAGGTCAGCGGTCACGCCATTTATGCCATCAGGCTGTGCCAGTGCAAGGTGTACTGGTCTGGGCCATGTGCCCCGTCACTTGTTGCTCCCAACCTGATTGAGAGACAAAAGAAGGTCAAGCTGTTTTGTCTGGAAACATTCCTCAGTGATCTCATTGCCCACCAGAAAGGACAGATAGAGAAGCAGCCACCATTTGAGATCTACTTATGCTTTGGAGAAGAATGGCCAGATGGGAAACCCTTGGAAAGGAAACTCATCTTGGTTCAGGTGATTCCAGTAGTGGCTCGGATGATCTACGAGATGTTTTCTGGTGATTTCACACGATCCTTTGATAGTGGCAGTGTCCGCCTGCAGATCTCAACCCCAGACATCAAAGATAACATCGTTGCTCAGCTGAAGCAGCTGTACCGCATCCTTCAAACCCAGGAGAGCTGGCAGCCCATGCAGCCCAGCCCCAGCATGCAACTGCCCCCTGCCCTGCCGCCCCAGTAA---------------------------------------------------------------------------------

>MouseLemur_ENSMICG00000001233

------------------------------------------------------------------------------------------------------------------------------------------------------------------------------------------------------------------ATGGCCCTGCACCCCCGCAGAGTCCGGCTGAAGCCCTGGCTGGTGGCCCAGGTGGATAGTGGCTTGTACCCTGGGCTCATCTGGCTACACAGGGACTCCAAACGCTTCCAGATTCCCTGGAAACATGCCACCCGGCATAGCCCCCAACAAGAGGAGGAAAATACCATTTTTAAGGCCTGGGCTGTAGAGACGGGGAAGTACCAGGAAGGGGTAGATGACCCTGATCCAGCTAAATGGAAGGCCCAGCTCCGCTGTGCTCTCAACAAGAGCAGAGAATTCAACCTGATGTATGATGGCACTAAGGAGGTGCCCATGAACCCAGTGAAGATATATCAAGTCTGTGACATCCCCCAGCCCCAGGGCTCAATCATTAACCCA------GGATCCACGGGGTCTGCTCCCTGGGATGAGAAGGATAATGATGTGGATGAAGATGATGAGGAAGATGAGCTGGACCAGTCGCAGCACCATGTTCCCATCCAGGACACCTTCCCCTTCCTGAACATTAATGGTTCTCCCATGGCGCCAGCC---------------AGTGTGGGAAACTGCGGTGTGGGTAACTGCAGCCCTGAAGCAGTGTGGCCCAAAACTGAACCTCTGGAGATGGAAGTGCCCCAGGCACCTATACAG---CCCTTCTTCAGCTCTCCAGAGCTGTGGATCAGCTCTCTCCCAATGACTGACCTGGACATCAAGTTTCACTACCGTGGGAAGGAGTATGGGCAGACCATGACCGTGAGCAACCCCCAGGGCTGCCGGCTCTTCTATGGGGATCTGGGTCCCATGCCTGACCAGGAAGAGCTCTTCGGTCCCGTCAGCCTGGAGCAAGTCAAATTCCCGGGTCCGGAGCATATCACCAATGAAAAACAGAAGCTGTTCACCAGCAAGCTGCTTGATGTCATGGACAGGGGACTGATCCTGGAGGTCAGCGGTCACGCCATTTATGCCATCAGGCTGTGCCAGTGCAAGGTGTACTGGTCTGGGCCATGTGCCCCATCGCTTGTTGCCCCCAACCTGATTGAGAGACAAAAGAAGGTCAAGCTGTTTTGTCTGGAAACATTCCTTAGTGAGCTCATTGCTCACCAGAAAGGACAGATAGAGAAGCAGCCACCATTTGAGATCTACTTGTGCTTTGGGGAAGAATGGCCAGATGGGAAACCCTTGGAAAGGAAACTCATCTTGGTTCAGGTCATTCCGGTGGTGGCTCGGATGATCTACGAGATGTTTTCTGGTGATTTCACACGATCCTTTGACAGTGGCAGTGTTCGCCTGCAGATCTCCACTCCAGACATAAAAGATAACATTGTTGCTCAGCTGAAGCAGCTGTACCGCATCCTTCAAACCCAGGAAAGCTGGCAGCCCATGCAGCCTGCCCCCAGCATGCAGCTGCCCCCTGCGCTGCCAGCCCAGTAA---------------------------------------------------------------------------------

>Mouse_ENSMUSG00000026638

------------------------------------------------------------------------------------------------------------------------------------------------------------------------------------------------------------------ATGGCCCTCCACCCTCGAAGAGTCCGGCTGAAGCCCTGGCTGGTGGCCCAGGTGGACAGTGGCCTCTACCCTGGTCTCATCTGGCTACACAGAGATTCCAAACGCTTCCAGATCCCCTGGAAACATGCCACGCGGCACAGCCCCCAACAAGAGGAAGAAAACACCATTTTTAAGGCTTGGGCTGTGGAGACCGGAAAGTACCAGGAAGGGGTAGACGATCCTGACCCAGCTAAATGGAAGGCTCAGCTCCGCTGTGCTCTCAACAAAAGCAGGGAGTTCAACTTGATGTACGATGGCACCAAGGAAGTGCCCATGAATCCTGTGAAGATCTATCAAGTGTGTGACATCCCCCAGACCCAGGGCTCTGTCATTAATCCA------GGATCCACAGGGTCTGCTCCTTGGGATGAGAAAGATAATGATGTGGATGAAGATGAGGAGGAGGATGAACTTGAGCAGTCACAGCACCATGTCCCCATCCAGGACACCTTCCCCTTCCTGAACATCAACGGTTCTCCCATGGCGCCAGCC---------------AGCGTGGGCAACTGCAGTGTGGGAAACTGCAGCCCCGAATCAGTGTGGCCCAAAACAGAACCTCTGGAGATGGAAGTACCCCAGGCACCCATTCAA---CCCTTCTACAGTTCTCCAGAGCTATGGATCAGCTCTCTCCCCATGACTGACTTGGACATCAAATTTCAGTATCGTGGGAAGGAGTATGGGCAAACTATGACGGTGAGCAACCCCCAGGGCTGCCGGCTCTTCTATGGGGACCTGGGCCCCATGCCCGACCAGGAGGAGCTCTTTGGTCCTGTCAGCCTGGAGCAGGTCAAGTTCCCAGGTCCAGAGCATATCACCAACGAGAAGCAGAAGCTGTTCACCAGCAAGCTGTTGGATGTCATGGACAGGGGACTGATCCTGGAGGTCAGCGGACATGCCATTTATGCCATCAGGCTGTGCCAGTGCAAAGTGTACTGGTCAGGGCCATGTGCTCCCTCGCTTGCTGCTCCTAACCTGATTGAGAGGCAGAAGAAGGTCAAGTTGTTTTGTCTGGAAACCTTCCTGAGTGAGCTCATTGCCCACCAGAAAGGACAGATAGAGAAGCAGCCGCCTTTTGAGATCTACTTATGCTTTGGGGAAGAGTGGCCAGATGGAAAGCCCCTGGAGAGGAAGCTCATCTTGGTCCAGGTCATCCCAGTGGTGGCTCGCATGATCTATGAGATGTTTTCTGGTGATTTTACCCGGTCCTTTGACAGTGGCAGCGTTCGCCTGCAGATCTCCACTCCAGACATCAAAGATAACATCGTTGCTCAGCTCAAGCAGCTGTACCGCATCCTTCAAACCCAGGAGAGCTGGCAGCCCATGCAGCCTGCCCCCAGCATGCAGCTGCCACAGGCTCTGCCTGCCCAGTGA---------------------------------------------------------------------------------

>Rat_ENSRNOG00000005082

------------------------------------------------------------------------------------------------------------------------------------------------------------------------------------------------------------------ATGGCCCTCCACCCTCGAAGAGTCCGGCTGAAGCCCTGGCTGGTGGCCCAGGTGGACAGTGGCCTCTACCCTGGTCTCATCTGGCTACATAGAGATTCCAAACGCTTCCAGATCCCCTGGAAACATGCCACGCGGCACAGCCCCCAACAAGAGGAAGAAAACACCATTTTTAAGGCTTGGGCTGTGGAGACTGGAAAGTACCAGGAAGGGGTGGACGATCCTGACCCAGCTAAATGGAAGGCTCAGCTCCGATGTGCTCTCAACAAAAGCCGGGAATTCAACTTGATGTATGACGGCACCAAGGAAGTGCCCATGAATCCTGTGAAGATCTATCAAGTGTGTGACATCCCCCAGCCCCAGGGCTCTGTCATTAACCCA------GGATCCACAGGGTCTGCTCCTTGGGATGAGAAAGATAATGATGTGGATGAAGATGACGAGGAAGATGAACTTGAACAGTCACAGCACCACGTCCCGATCCAGGACACCTTCCCCTTCCTGAACATCAATGGTTCTCCCATGGCACCAGCC---------------AGCGTGGGCAACTGCAGTGTGGGAAACTGCAGCCCTGAATCAGTGTGGCCCAAAACAGAACCTCTGGAAATGGAAGTACCGCAGGCACCCATTCAG---CCCTTCTATAGTTCTCCAGAGCTGTGGATCAGCTCCCTCCCCATGACTGACTTGGACATCAAGTTTCAGTATCGTGGGAAGGAGTATGGGCAAACCATGACGGTGAGCAACCCCCAGGGCTGCCGGCTCTTCTATGGGGACCTGGGCCCCATGCCTGACCAGGAGGAGCTCTTTGGTCCTGTCAGCCTGGAGCAGGTCAAGTTCCCGGGGCCAGAGCATATCACCAATGAGAAGCAGAAGCTGTTCACCAGTAAGCTGTTGGATGTCATGGACAGGGGACTGATCCTGGAGGTCAGCGGACATGCCATTTATGCCATCCGGCTGTGCCAGTGCAAAGTGTACTGGTCGGGGCCGTGTGCCCCCTCACTTGCTGCTCCTAACCTGATTGAGAGGCAGAAGAAGGTCAAGTTGTTTTGTCTGGAAACATTCCTGAGTGAGCTCATTGCCCATCAGAAAGGGCAGATAGAGAAGCAGCCACCTTTCGAGATTTACTTATGCTTCGGGGAAGAATGGCCAGATGGAAAGCCCCTGGAGAGGAAGCTCATCTTGGTCCAGGTCATTCCAGTGGTGGCTCGCATGATCTATGAGATGTTTTCTGGTGATTTCACCCGGTCCTTTGACAGTGGCAGTGTTCGCCTGCAGATTTCCACTCCAGACATCAAAGATAACATCGTCGCTCAGCTCAAGCAGCTGTACCGCATCCTTCAAACCCAGGAGAGCTGGCAGCCCATGCAGCCTGCCCCCAGCATGCAGCTGCCGCCTGCCCTGCCTGCCCAGTGA---------------------------------------------------------------------------------

# IRF7

>Mouse_ENSMUSG00000025498

------------------ATGGCTGAAGTG---------------------------AGGGGGGTCCAGCGAGTGCTGTTTGGAGACTGGCTATTGGGGGAGGTCAGCAGCGGCCAGTACGAGGGGCTGCAGTGGCTGAACGAGGCTCGCACAGTCTTCCGCGTACCCTGGAAGCATTTCGGTCGTAGGGATCTGGATGAAGAAGATGCACAGATCTTCAAGGCCTGGGCTGTGGCCCGAGGGAGGTGGCCACCTAGT------GGAGTTAACCTGCCACCCCCAGAGGCTGAGGCTGCTGAGCGAAGAGAGCGAAGAGGCTGGAAGACCAACTTCCGCTGTGCACTCCACAGCACAGGGCGTTTTATCTTGCGCCAAGACAATTCAGGGGATCCAGTTGATCCGCATAAGGTGTACGAACTTAGCCGGGAGCTTGGATCTACTGTGGGCCCAGCCACGGAAAATAGGGAAGAAGTGAGCCTCAGCAATGCTCTGCCCACACAGGGTGTGTCCCCAGGATCATTTCTGGCAAGAGAAAATGCTGGG---------------------------------------CTCCAAACCCCA---------------AGCCCTCTG------CTTTCTAGTGATGCCGGGGACCTCTTGCTTCAGGTTCTGCAGTACAGC------------CACATACTGGAATCCGAGTCTGGGGCAGACCCCGTCCCACCACAGGCTCCTGGCCAGGAGCAAGACCGTGTT---------------------------TACGAGGAACCCTATGCAGCA------------------------------------------------------------------------TGGCAGGTGGAAGCTGTCCCCAGTCCCAGGCCTCAACAGCCA------GCTCTC------------------------------------------------------------------------------------------------------------------------------------------ACCGAGCGCAGCCTTGGGTTCCTGGATGTGACCATCATGTACAAGGGCCGCACAGTGCTACAGGCAGTGGTGGGGCACCCCAGATGCGTGTTCCTGTACAGCCCCATGGCCCCAGCAGTAAGAACTTCAGAGCCCCAGCCG---------GTGATCTTTCCCAGTCCTGCTGAGCTCCCAGATCAGAAGCAGCTGCACTACACAGAGACGCTTCTCCAGCATGTGTCTCCCGGCCTTCAGCTGGAGCTTCGAGGACCGTCACTGTGGGCCCTGCGTATGGGCAAGTGCAAGGTGTACTGGGAGGTAGGCAGCCCTATGGGCACTACCGGCCCCTCCACCCCACCCCAGCTGCTGGAGCGCAACCGCCACACCCCCATCTTCGACTTCAGCACTTTCTTCCGAGAACTGGAGGAGTTTCGGGCTCGGAGGCGGCAAGGGTCACCACACTACACCATCTACCTGGGTTTTGGGCAAGACTTGTCAGCAGGGAGGCCCAAGGAGAAGACCCTGATCCTGGTGAAGCTGGAGCCATGGGTATGCAAGGCATACCTGGAGGGCGTGCAGCGTGAGGGTGTGTCCTCCCTGGACAGCAGCAGTCTCGGCTTGTGCTTGTCT------------------AGCACCAACAGTCTCTACGAAGACATCGAACACTTCCTC------------ATGGACCTG------GGTCAGTGGCCTTGA------------------------------------

>Rat_ENSRNOG00000017414

------------------ATGGCCGAAGTG---------------------------AGGGGGGTCCAGCGAGTGCTGTTCGGAGACTGGCTCTTGGGTGAGGTCAGTAGTGGCCGGTACGAGGGGCTGCAGTGGCTGAATGAGGCTCACACAGTCTTCCGTGTGCCCTGGAAGCATTTCAGCCGTAGGGATCTGGATGAAGCTGATGCACAGATCTTCAAGGCCTGGGCTGTGGCCCGAGGGAGGTGGCCACCTAGT------GGAGTTAACTTACCACCCCCAGAGGCTGAGGCTGCT---------GAGCGAAGAGGCTGGAAGACCAACTTCCGATGTGCACTCCGCAGCACAGGGCGCTTTATCTTGCGTCAAGACAACTCAGGGGACCCTGCTGATCCGCACAAGGTGTATGAACTTAGCCCGGAGCTTGGATCTACAACTGGCCCAGCTCTGGAGAACAGGGAAGAAGTGACCCTCAGCAGTGCCCTACCCACACAGGGTATGTCCCTAGGATCATTCCTGGCAAGAGGAAATGCTGGG---------------------------------------TTGCAAACCCCA---------------AGCCCTCTG------CTTTCTGGTGATGCTGGGGACCTCTTGCTTCAGGTTCTGCAATACAGC------------CACCTACTGGACTCTGAGTCTAGGGCGGACTCAGTCCCACCACAGGCTTCTGGCCAGGAGCAAGAACACCTT---------------------------CCTGAGGAGCCCCATGCAGCA------------------------------------------------------------------------TGGCAGATGGAAGCTACCTCCAGCCCCAGGCTTCAACACCCA------GTTCTGATGACTGAGCGCAAC---------------------------------------------------------------------------------------------------------------------------------------CTTGGGTCCCTGGATGTGACCATCATGTACAAGGGCCGCACAGTGCTACAGGCAATGGTGGGGCACCCCAGATGCGTGTTCCTGTATAGCCCCGTGGGCTCAGCAATAAGAACTTCAGAGCCCCAGCCG---------GTGATTTTTCCGAGCCCCGCTGAGCTCCCAGATCAGAAGCAGCTGCACTACACAGAGACTCTCCTACAGCATGTGTCTCCAGGCCTTCAGCTGGAGCTTCGGGGGCCATCACTATGGGCCCTGCGAATGGGCAAGTGCAAGGTGTACTGGGAGGTGGGCAGCCCTATGGGCTCCACCAACCCCTCCACCCCAGCCCAGCTGCTGGAGCGCAACTGCCATACTCCCATCTTTGACTTCAGCACTTTCTTCCGAGAACTGGAGGAGTTCCGGGCCCGGAGGCGACAAGGATCACCACATTACACCATCTACCTGGGTTTTGGGCAAGACTTGTCAGCAGGGAGGCCCAAGGAGAAGAGCCTGATCCTGGTGAAGCTGGAGCCATGGCTGTGCAAGACACATCTGGAAGGCGTGCAGCGTGAGGGTGTGTCCTCTCTGGACAGCAGCAGTCTTGGCCTGTGCTTGTCT------------------AGCACCAATAGTCTCTACGATGACATTGAACACTTCCTC------------ATGGACCTG------GGTCAGTGGGCTTGA------------------------------------

>Horse_ENSECAG00000009744

------------------ATGGCTGTGGCTCCCGAC---------------------AGGGTAGCCCCGCGCGTGCTGTTCGGAGACTGGCTTTTGGGCGAGGTCAGCAGCGGCCGCTACGAGGGGCTGGAGTGGCTGGACGCAGCCCGCACACACTTCCGTGTCCCCTGGAAGCACTTCGGGCGGAGGGACCTGGGCGAGGCGGACTCACGCATCTTCAAGGCCTGGGCCGTAGCCCGCGGCAGGTGGCCGCCCAGCAGCAGCAGAAGTGACCCTCAGACCCCC------GAGAGCGCC---------CTCCGCGCAGGCTGGAAAACCAACTTCCGCTGCGCATTGCGCAGCACGCGGCGCTTCGTGATGCTGCAAGACAACTCTGGGGACCTCACGGACCCGCATAAGGTGTATGCGCTCAGCCCCGAGGTGGGGTGGGCAGAAGATGCAGGCATTAACCAGGGGAAGGATGAGGCCCTT---GATGTCCCCCCCAGGATGGGCGGGTTCCTCAGGCCAGCCCTGGCA---GGAGATGCTGGTGAGAGGCGGGGGCACCAGCTGGCTGGGCCGAACCCTGAGCCCTGCGCCCCA---------------AGCCCTCTTTCCGGCCCAGCTGACGATGTGGAGGACCTCTTGCTCCAGGCTCTGCAGCAGAGCAGCCTGGAGGACCACCTGCTGGAAGCTACATGGGGGGTGGATCCAATCTCCCCAGAGGCTCCTGGC---------------------------------------------CCAGAGCTCCCTGCGGGGCAGCTGTACCTGCCAAAGCCCCAGGCCCAGACGACAGGTGAGAGGAACCTGGGGACATGTGGAGGAGGGAGGTGCTGGGGGACCAGGTGCCAACACAGCCCT---------------------TCCCTGGACGCAGGCCTAGCCCCAGCACCCTGGCAGCCCCCTCAAGAGGCAGAGCCCTGCACTGCGCTGCCCCCCAGTGCCTGTCCCCCGGTGGCAGGTGAGCAGGTCCCAGCCAGGCCTGGCTACTCACAGCTCAGTCTGCACACAGAACCCAGCCTGGGCACCCTGGACGTGACCATCATGTACAAGGGCCGAACGGTGCTGCAGGAGGTGGTGGGCCGCCCAAGATGTGTGTTCCTATACGGGCCTCCCAGCCTACCTGTTGAGGCCACAGAACTCCAGTGC---------GTGGCCTTCCCCAGCCCTGCTGAGCTCCCCGACCAGAAACAGCTTCACTACACAGAGAAGCTGCTGCAGCACGTGGCCCCTGGCCTGCAGCTGGAGCTCCGGGGGCCTGGGCTGTGGGCCCGGCGCCTGGGCAAGTGCAAGGTCTTCTGGGAGGTGGGAGGCCCCCTGGGCTCTGCCAGCCCCTCTAGCCCGGCCCGCCTGCTGCCCAGGAACTGCGACACTCCCATCTTCGACTTCAGCACCTTCTTCCGAGAGCTTGTGGAGTTCCGGGCTCGCCAGCGCCAGGGCTCCCCACACTACACCATCTACCTGGCCTTTGGGCAAGACCTGTCAGCCAGGAGACCCAAGGAGAAGAGCCTGGTCCTGGTGAAGCTGGAGCCGTGGCTGTGCCGGGCGCACCTGGAGGGTGTGCAGCGTGAAGGCGTGTCCTCCCTGGACAGCGGCAGCCTAGGCCTCTGCCTGTCC------------------AGCTCCAACAGCCTCTACAACGACCTCGAGCATTTCCTTGACCACTTCTTCATGGAGGTG------GAGCAGCCCGCCTAG------------------------------------

>Megabat_ENSPVAG00000006873

GCGCCCGCCCCCGCGTGCCCTGCGGCCCGCCCTGAC------CGGCTGGTCGCCCGCAGGGGGGCCCCGCGCGTGCTGTTCGCAGACTGGCTTCTGGGCGAGGTCAGCAGCGGCCGCTACGAGGGGCTGCGGTGGCTGGACGAGGCTCGCACACGCTTCCGAGTGCCCTGGAAGCACTTTTCGCGGAAGAACCTGGGCGAGGCCGACTCGCTCATCTTCAAGGCCTGGGCCATCGCCCGCGGCAGGTGGCCGCTCAGCAGCGGCCCAGGCAACCCGCCCTCC---------GAAAGTGCA---------CTCCGAGCCGGCTGGAAAACCAACTTCCGCTGCGCACTGCGCAGCACTCAGCGCTTCGTCATGCTGCACGACAATTCCGCGGACCCCGCCGACCCGCATAAAGTGTATGAGCTCAGCTCCGAACCGCCGTGGAGAGAAAGTCCAGGGATTAACCAGGGGGAGCACAAGGCCCTTGAGGATGCCTCATCCTGGAGGGGTGGGCTCCCTAGGCCACATCTGGTAGACGCAGAGGCTGGG---------------------------------------GCCTGCGCCCAG---------------TGCCCCAGCCCCGGCCTGGCAGGCTCCACGGGGGACCTTCTGCTCCAGGCTCTGCAGCAGAGCCACCTGGAGGACCATCTGCTGACGGTGCAG---GAGGTGGACCCAGTCCCCCTAGAGGCTCCTGGTGAGAGG---------------------GTTGGATGC---------CCAGAGCTCCCTGCTGAGGAACCATACCTGCCG------------------------------------------------------------TGGGCCATGGAGGTGGCCGCCAGCCCCAGGTCACAGCCCCAG------GCCGCAATGACAGGTCCCGCCCCAGAGCCCTGG---------------------------------------------------------CCC---------------------------------------------------TACAAGTCTGGCCTGGGGACCCTGGACGTGACCATCATGTACAAGGGCCGAACAGTGCTGCAGGAGGTGGTGGGGCGCCCGCGCTGTGTGCTGCTCTACGGGCCCCCTGGTCTAGCCAGCGAGGCCAGAGAGCCCCAAGAGCCTCAGATGGTGGCCTTCCCCAGCCCAGCCGAGCTCCCTGACCAGAAGCAGCTGCACTACACAGAGAAGCTGCTGCGGCACGTGGCCCCGGGCCTGCAGCTTGAGCTCCGGGGGCCTGGGCTGTGGGCCCGGCGCCTGGGCAAGTGCAAGGTCTACTGGGAGGTGGGCGGCCCGCTGGGCTCCGACAACCCCTCCACACCGGCCCGCCTGCTGCAAAGGAACTGTGACACCCCCATCTTTGATTTTGGCACCTTCTTCCGAGAGCTGGTGGAGTTCCGGACTCGGCAGCGCCGAGGCTCTCCACACTACACCATCTACCTGGGCTTCGGGCAGGACCTGTCGGCTGGGAGGCCCAAGGACAGGAGCCTGGTTCTGGTGAAGCTGGAGCCGTGGCTGTGCCGCGCATACCTGGAGAGCGTGCAGCGGGAAGGCGTGTCCTCCCTGGACAGCAGCAGCTTCAGCTCTGCTCTGTCC------------------AGCTTCAACAGCCTGTATGAGGACCTGGAGCACTTCCTG------------GAGCACTTC---------------------------------------------------------

>Elephant_ENSLAFG00000032120

------------------CGTTCCACCCAC---------------------------AGAGGGACCCCACGCGTGCTGTTCGGAGAGTGGCTTCTGCACGAGATCAGCAGCGGCCGCTACGAGGGGCTGCGGTGGCTGGATGCGGCCCGTACGCGCTTCCGCGTGCCCTGGAAACACTTCGCACGCAAGGATCTGGAAGAGGCTGACGCGCACATCTTCAAGGCGTGGGCTGTGGCCCGCGGCAGGTGGCCACCCAGCAACTGCGCGGGCACCCAGCCGCCCCCC------GAAGCACCA---------GAGCGCGCCGGCTGGAAAACTAACTTCCGCTGCGCCCTGCACAGCACGCGGCGCTTCGTGATGCTGCAGGACAACTCGGGAGACCCCGCGGATCCGCACAAGGTGTTTGCGGTCAGCCCCGCGCCGGGAAGAAGAGAAGGCCCAGGCATCGACAGAACAGAA---GAGGCCCCTGCAGATGGCTGGCCCATGAGGGGTGAGCCTCCTGGGCCATTCCTGGGAGGAGAAGCTGCAACT---------------------------------------AGGCAGAGCCCTGAGCCCTGTACCTCAAACCCTCTTCCCGCCCGGGCTGGTGACACCAGGGACCTTCTGCACCAGGCTCTGCAGCAGAGCCACCTTGGAGACGATTTGCTGGAAGCAGCATGGAGGATGGATCCGGCCCCCCAGCAGCCTTCCGGTAAGAGACAGGAGGGA---------------------------------GGAGGGGCAGGTGAGGAGCTATACTTGCCG------------------------------------------------------------TGGGCAGTGGAGGCGGCCCCCAACCCCCCACCACAGCCCCCCGACCAGGCCCTGATGACAGGTAGG------------------------------------------------------------------------------------------------------------------------------------------AGAGGGGCTGGGAACANGACCATCCTGTACAAGGGCCGCCTGGTGCTGCAGGAAGTAGTGGGC---ACGAGCTGCGTGCTCCTGTACCAGCCCCCTGGCCCCGCTGCCGAGGCCACAGACGTGCAGCTG---------GTGGCCTTCCCCAGCCCAGCCGAGCTCCCCGACCAGAAGCAGCTTCGCTACACGGAGGAGCTGCTGCGACACGTGGATCCGGGGCTGCAGCTGGAGCTGCGCGGAGGCAGCCTGTGGGCGCGGCGCCTGGGCAAATGTAAGGTGTACTGGGAGGTGGGCGGCCCCCTGGGCTCTGCCAGCCCCTCCAGCCACGCCTGCCTGCTGCATAGGAACTTCGACACTCCCATCTTCGACTTCGGAGTCTTCTTCAGGGAGCTGGTGGAATTCCGGGAACGGCGGCGCCAGGGCTCCCCGCACTATACCATCTACCTGGGCTTTGGGCAGGACCTGTCTGCTGGGAGGCCGAAGGAGAAGAGCCTGGTCCTGGTGAAGCTGGAGCCATGGCTCTGCAGGGCGTATCATGAAGGCGCGCAGCGTGAGGGCACGTCCTCCCTGGACAGCAGCAGCCTCGGCCTCTGCCTGTCC------------------AGCTCCAACAGCCTGTACGATGACATCAACCACTTCCTC------------ATGGAGCTG------GGGCAGCCT------------------------------------------

>Rabbit_ENSOCUG00000002339

------------------ATGGCCTCCGCTCCCGAG---------------------GGGCGGCCTCCGCGCGTGCTCTTCGGGGAGTGGCTCCTGCAACAGGTCAGCAGCGGCCGCTACGAGGGGCTGTGCTGGCTGAACGAGGCCCGCACGCGCTTCCGCGTGCCCTGGAAGCACTTCGCGCGCAGGGACCTGAGCGAAGCCGACGCGCGCATCTTCAAGGCCTGGGCGGTGGCCCGCGGTAGGTGGCCGCCCAGCCGCCTCGAAGGTGACCAGCTGCCCCCA------GAAGCGGCT---------GAGCGCGCTGGCTGGAAAACCAACTTCCGCTGCGCGCTGAAGAGCACGCGACGCTTCGTGATGCTGCGCGATAACTCCACGGACCCCGCAGACCCGCACAAGGTGTTCGAGCTGAGCCCCACGCCGCGCGGGAGAGAAGACCCAGGCGTGGAG------GAGGAAGATGCTCTCCAAGATGCCCCACCCACACAGGGCGGGGCCCCCACGCCATTCCTGGCCGGAGGAGCAGCGGCC---------------------------------------------GCCCCC------------------------------------------TGCGGGGACATCCTGCTGGAGGCTCTGCAG---AGCTGCCTGGGGGAA---------------GAGTGGGGGGCAGACCCAGCCCCTCCACAGGCTCTGGGC---------------------------------------------CCAGCGCTCCCTGTTGAAGTGTCCCCAGCG---------------------------------------------------------------TGCACAGCAGAGGGCGTTCTGAGCCCAGAGCCCCCGGCCCGG------GCCCTG------------------------------------------------------------------------------------ATG---------------------------------------------------ACAGAGCCCAGCCCAGGCGTCCTGGACGTGACCATCATGTACAAGGGGCGCACGGTGCTGCAGGAGGTGGTGGGGCGCCCGAGCTGCGTGTTCCTGTACGGGCCCCCCAGCCCCGCGGTGGCAGGGGCAGGGCCACAGCAG---------GTGGCCTTCCCCAGCCCGGCTGAGCTGGCGGACCAGCAGCAGCTGCGGTACACGGAGGAGCTGCTGCGGCACGTGGCGCCCGGGCTGCAGCTGGAGCTGCGGGGCTGGGGGCTGTGGGCCCTGCGCATGGGCAAGTGCAAGGTGTACTGGGAGGTGGGCGGCCCCCTGGGCTGCGACCGCCCCTGCAGCCCCGCCCGGCCGCTGGCGCGCAACTGCCACACCCCCATCTTCGACTTCAGCACCTTCTTCCAAGAGCTGGCAGAGTTCCGGGCACGGCGGCGCAAGGACTCCCCACGCTACACCATCTACCTGGGCTTCGGGCAGGACCTGTCGGCCAGCAGGCCCAAAGACAAGAGCCTGGTCCTGGTCAAGCTGGAGCCGTGGCTGTGCCGTGCACACCTGGAGGGGGTGCAGCGGGAGGGCGTGTCCTCCCTGGACGGCGGCAGCCTGAGCCTGTGCCTGTCC------------------AGCACCAACAGCCTGTACGACGACATTGAGCGCTTCCTC------------ATGGAGCTG---------------GGGTAG------------------------------------

>Marmoset_ENSCJAG00000012124

------------------ATGGCCTTGGCTTCGCAG---------------------AGGGCAGCCCCACGCGTGCTGTTCGGAGAGTGGCTCCTTGGGGAGGTCAGCAGCGGCCGCTACGAGGGGCTGCGGTGGCTGGACGAGGCACGCACCCGCTTCCGCGTGCCCTGGAAGCACTTCGGGCGCAAGGACCTGAGCGAGGCGGACGCGCGCATCTTCAAGGCCTGGGCCGTGGCTCGCGGCAGGTGGCCGCCCAGCAGCAGCGGAGGTGACCAGCCGCCC------TGCGAGGCTGCG---------GAGCGCGCCGGCTGGAAAACCAACTTCCGCTGCGCCCTGCACAGCACGCGTCGCTTCGTGATGCTACGGGACAACTCGGGGGACCCTGCGGACCCCCACAAGGTGTACACGCTCAGCCAGGAGCTGGGCTGCCGAGAAGGCCCAGGCACGGACCGGACAGAGGCAGAGGCCCCCATGGCTGACCCACCCTCCCAGGCTGGGACCCCAGGGCCATTCCTAGCACAAGGAGATGCTGAA---------------------------------------GTCCAAGTCCCA---------------AGCCTCCTCCCTGCCCCAGCCGGGGACCAGCAGGACCTCCTGCTTCAGGCGATGTGGCAGAGCTGCCTGGGGGACAATCTGCTGACAGCATCATGGGGGGCAGATCCAGGCCCAGCCAAGGCTCCTGGAGAGGGACTGAACGCGCTTTCCCCGACTGGGGGCTATGCTAGAGACTCAGGGCTCCCTGCTGGGGAGCTGTACAAA---------------------------------------------------------------TGGGCAGCAGAGGGGACCCCAAGCCCCAGGACCCAGCCCCCG------GCCCTAATGGCAGGCGAGGCCACGGCCCCA---GAGCCCCTGCACCAGGCAGAGCCATACCTGGCCCGCTCCCCAGGTGCCTGCACTGTGGTG---------------------------------------------------GGAGAGCCCGGCCCAGGGGTGCTGGACGTGACCATCACCTACAAGGGCCGCACGGTCCTGCAGCAGGTAGTGGAGCGCCCGAGCTGTGTGTTCCTGTATGGCCCCCAGGGCCCAGCCATCTGGGCCACAGACCCTCAGCCG---------GTGGCATTCCCCAGCCCTGCTGAGCTTCCGGACCAGAAGCAGCTGCGCTACACGGAAGAGCTGCTGCGGCATGTGGCCCCCGGGCTGCAGCTGGAGCTTCGGGGGCCGCAGCTGTGGGCTCGGCGCATGGGCAAGTGCAAGGTATACTGGGAGGTTGGCGGCCCCCCAGGCTCCGCCAGCCCCTCCACCCCAGCCTGCCTGCTGCCTCGCAACTGTGACACCCCCATCTTCGACTTCAGAGTCTTCTTCCAAGAGCTGGTGGAATTTAGGGCACGGCGCCGCTGCAACTCCCCACACTGCACCATCTACCTGGGCTTCGGCCAGGACTTGTCAGCCGGGAGACCCAAGGAGAAGAGCCTGGTCCTGGTGAAGCTGGAGCCCTGGCTGTGCCGAGTGTACCTGGAGGGCACGCAGCGCGAGGGGGTGTCGTCCCTGGACAGCAGCAGCCTGAGCCTCTGCCTGTCC------------------AGCGCCAACAGCCTCTACGATGTCATCGAGGGCTTCCTT------------ATGGAGCTG------GAGCAGCCTGCCTAG------------------------------------

>Gibbon_ENSNLEG00000008419

------------------ATGGCCTTGGCTCCTGAG---------------------AGGGCGGCCCCACGCGTGCTGTTCGGAGAGTGGCTCCTTGGAGAGATCAGCAGCGGCTGCTATGAGGGGCTGCAGTGGCTGGACGAGGCCCGCACCTGCTTCCGCGTGCCCTGGAAGCACTTCGCTCGCAAGGACCTGAGCGAGGCCGACGCGCGCATCTTCAAGGCCTGGGCCGTGGCCCGCGGCAGGTGGCCGCCTGGCAGCAGGGGAGGTGACCCGCCGCCCCCAGAGGCTGAGGCTGCG---------GAGCGCGCGGGCTGGAAAACCAACTTCCGCTGCGCACTGCGCAGCACGCGTCGCTTCGTGATGCTGCGAGATAACTCGGGCGACCCGGCCGACCCGCACAAGGTGTACGCGCTGAGCCGGGAGCCCTGCTGGCGAGAAGGCCCAGGCACGGACCAGACTGAGGCAGAGGCCCCCGCGGCCGTCCCGCCACCACAGGGTGGGCCCCCAGGGCCGTTCCTGGCACACAGAGATGCGGGA---------------------------------------CTCCAAGCCCCA---------------GGCCCCCTCCCTGCCCCAGCTGGTGACAAGGGGGACCTCCTGCTCCAGGCAGTGCAACAGAGCTGCCTGGCGGGCCATCTGCTGACAGCGTCATGGGGGGCAGATCCAGTCCCAGCCGAGGCTCCTGGAGAGGGACAAGAAGGGCTTCCCCTGACTGGGGCCTGTGCTGGAGGCCCAGGGCTCCCTGCTGGGGAGCTGTTCGGG---------------------------------------------------------------TGGGCAGTAGAGGCGACCCCCAGCCCCGGGCCCCAGCCCGCG------GCACTAATGACAGGCGAGGCCACGGCCCCA---GAGCCCCCGCACCAGGCAGAGCCGTACCTGGCACCCTCCCCAAGCGTCTGCACCGCGGTG---------------------------------------------------CAAGAGCCCAGCCCAGGGGCGCTGGACGTGACCATCATGTACAAGGGCCGCACGGTGCTGCAGAAAGTGGTGGGACACCCGAGCTGCACGTTCCTATACGGCCCCCCAGACCCAGCTGTCCGGGCCACAGACCCCCAGCAG---------GTAGCATTCCCCAGCCCCGCTGAGCTCCCCGACCAGAAACAGCTGCGCTACACGGAGGAACTGCTGCGGCACGTGGCCCCTGGGTTGCACCTGCAGCTTCGGGGGCCACAGCTGTGGGCGCGGCGCATGGGCAAGTGCAAGGTGTACTGGGAGGTGGGCGGCCCCCCAGGCTCCGCCAGCCCCTCCACCCCAGCCTGCCTGCTGCCTCGGAACTGTGACACCCCCATCTTCGACTTCAGAGTCTTCTTCCGAGAGCTGGTGGAATTCCGGGCACGGCAGCGCCGCGGCTCCCCACGATATACCATCTACCTGGGCTTCGGGCAGGACCTGTCGGCTGGGAGGCCCAAGGAGAAGAGCCTGGTCCTGGTGAAGCTGGAGCCCTGGCTATGCCGAGTGCACCTGGAGGGCACGCAGCGTGAGGGTGTGTCTTCCTGGATAGCAGCAGCCTCAGCTCTGCCTGTCCAGTGTCAGCGCAAGAACCTGACGGCAGGCATGCTGATGCCCCCCATTCCCCGCCACTCC------------CGACAGCTGCCCGGGCATGGCCCCGTTTCCCGCCACTCCCGACAGCTCCCTGGGCATGGCCCGTGA

>Gorilla_ENSGGOG00000008341

ATGCCAGTCCCCGAGCGCCCTGCGGCCGGCCCTGACTCTCCGCGGCCGGGCACCCGCAGGGCGGCCCCACGCGTGCTGTTTGGAGAGTGGCTCCTTGGAGAGATCAGCAGCGGCTGCTACGAGGGGCTGCAGTGGCTGGACGAGGCCCGCACCTGCTTCCGCGTACCCTGGAAGCACTTCGCGCGCAAGGACCTGAGTGAGGCCGACGCGCGCATCTTCAAGGCCTGGGCTGTGGCCCGCGGCAGGTGGCCGCCTAGCAACAGGGGAGGTGAC---CCGCCCCCCGAGGCTGAGACTGCG---------GAGCGCGCCGGCTGGAAAACCAACTTCCGCTGCGCACTGCGCAGCACGCGTCGCTTCGTGATGCTGCGAGATAACTCGGGGGACCCGGCCGACCCGCACAAGGTGTACGCGCTCAGCCGGGAGCTGTGCTGGCGAGAAGGCCCAGGCACGGACCAGACTGAGGCAGAGGCCCCCGCAGCTGTCCCACCACCACAGGGTGGGCCCCCAGGGCCATTCCTGGCACACACAGATGCTGGA---------------------------------------CTCCAAGCCCCA---------------GGCCCCCTCCCTGCCCCAGCTGGTGACAAGGAGGACCTCCTGCTCCAAGCAGTGCAACAGAGCTGCCTGGCGGACCATCTGCTGACAGCGTCATGGGGGGCACATCCAGTGCCAGCCAAGGCTCCTGGAGAGGGACAAGAAGGGCTTCCCCTGACTGGGACCTGTGCTGGAGGCCCAGAGCTCCCTGCTGGGGAGCTGTACGGG---------------------------------------------------------------TGGACAGTAGAGACGACCCCCAGCCCCGGGCCCCAGCCCGCG------GCACTAATGACAGGCGAGGCCACGGCCCCA---GAGTCCCCGCACCAGGCAGAGCCGTACCTGGCACCCTCCCCAAGCGCCTGCACCGCGGTG---------------------------------------------------CAAGAGCCCAGCCCAGAGGCGCTGGACGTGACCATCATGTACAAGGGCCGCACGGTGCTGCAGAAGGTGGTGGGACACCGGAGCTGCACGTTCCTGTACGGCCCCCCAGACACAGCTGTCCGGGCCACAGACCCCCAGCAG---------GTAGCATTCCCCAGCCCTGCTGAGCTCCCGGACCAGAAGCAGCTGCGCTACACGGAGGAACTGCTGCGGCATGTGGCCCCTGGGTTGCACCTGGAGCTTCGGGGGCCACAGCTGTGGGCCCGGCGCATGGGCAAGTGCAAGGTGTACTGGGAGGTGGGCGGCCCCCCAGGCTCCGCCAGCCCCTCCACCCCAGCCTGCCTGCTGCCTCGGAACTGTGACACCCCCATCTTCGACTTCAGAGTCTTCTTCCGAGAGCTGGTGGAATTCCGGGCACGGCAGCGCCGCGGCTCCCCACGCTATACCATCTACCTGGGCTTCGGGCAGGACCTGTCAGCTGGGAGGCCCAAGGAGAAGAGCCTGGTCCTGGTGAAGCTGGAACCCTGGCTGTGCCGAGTGCACCTAGAGGGCACGCAGCGTGAGGGTGTGTCTTCCCTGGATAGCAGCAGCCTCAGCCTCTGCCTGTCC------------------AGCGCCAACAGCCTCTATGACGACATCGAGTGCTTCCTT------------ATGGAGCTG------GAGCAGCCCACCTAG------------------------------------

>Human_ENSG00000185507

ATGCCAGTCCCCGAGCGCCCTGCAGCCGGCCCTGACTCTCCGCGGCCGGGCACCCGCAGGGCAGCCCCACGCGTGCTGTTCGGAGAGTGGCTCCTTGGAGAGATCAGCAGCGGCTGCTATGAGGGGCTGCAGTGGCTGGACGAGGCCCGCACCTGTTTCCGCGTGCCCTGGAAGCACTTCGCGCGCAAGGACCTGAGCGAGGCCGACGCGCGCATCTTCAAGGCCTGGGCTGTGGCCCGCGGCAGGTGGCCGCCTAGCAGCAGGGGAGGTGGC---CCGCCCCCCGAGGCTGAGACTGCG---------GAGCGCGCCGGCTGGAAAACCAACTTCCGCTGCGCACTGCGCAGCACGCGTCGCTTCGTGATGCTGCGGGATAACTCGGGGGACCCGGCCGACCCGCACAAGGTGTACGCGCTCAGCCGGGAGCTGTGCTGGCGAGAAGGCCCAGGCACGGACCAGACTGAGGCAGAGGCCCCCGCAGCTGTCCCACCACCACAGGGTGGGCCCCCAGGGCCATTCCTGGCACACACACATGCTGGA---------------------------------------CTCCAAGCCCCA---------------GGCCCCCTCCCTGCCCCAGCTGGTGACAAGGGGGACCTCCTGCTCCAGGCAGTGCAACAGAGCTGCCTGGCAGACCATCTGCTGACAGCGTCATGGGGGGCAGATCCAGTCCCAACCAAGGCTCCTGGAGAGGGACAAGAAGGGCTTCCCCTGACTGGGGCCTGTGCTGGAGGCCCAGGGCTCCCTGCTGGGGAGCTGTACGGG---------------------------------------------------------------TGGGCAGTAGAGACGACCCCCAGCCCCGGGCCCCAGCCCGCG------GCACTAACGACAGGCGAGGCCGCGGCCCCA---GAGTCCCCGCACCAGGCAGAGCCGTACCTGTCACCCTCCCCAAGCGCCTGCACCGCGGTG---------------------------------------------------CAAGAGCCCAGCCCAGGGGCGCTGGACGTGACCATCATGTACAAGGGCCGCACGGTGCTGCAGAAGGTGGTGGGACACCCGAGCTGCACGTTCCTATACGGCCCCCCAGACCCAGCTGTCCGGGCCACAGACCCCCAGCAG---------GTAGCATTCCCCAGCCCTGCCGAGCTCCCGGACCAGAAGCAGCTGCGCTACACGGAGGAACTGCTGCGGCACGTGGCCCCTGGGTTGCACCTGGAGCTTCGGGGGCCACAGCTGTGGGCCCGGCGCATGGGCAAGTGCAAGGTGTACTGGGAGGTGGGCGGACCCCCAGGCTCCGCCAGCCCCTCCACCCCAGCCTGCCTGCTGCCTCGGAACTGTGACACCCCCATCTTCGACTTCAGAGTCTTCTTCCAAGAGCTGGTGGAATTCCGGGCACGGCAGCGCCGTGGCTCCCCACGCTATACCATCTACCTGGGCTTCGGGCAGGACCTGTCAGCTGGGAGGCCCAAGGAGAAGAGCCTGGTCCTGGTGAAGCTGGAACCCTGGCTGTGCCGAGTGCACCTAGAGGGCACGCAGCGTGAGGGTGTGTCTTCCCTGGATAGCAGCAGCCTCAGCCTCTGCCTGTCC------------------AGCGCCAACAGCCTCTATGACGACATCGAGTGCTTCCTT------------ATGGAGCTG------GAGCAGCCCGCCTAG------------------------------------

>Bonobo_ENSPPAG00000030184

ATGCCAGTCCCCGAGCGCCCTGCAGCCGGCCCTGACTCTCCGCGGCCGGACACCCGCAGGGCAGCCCCACGCGTGCTGTTCGGAGAGTGGCTCCTTGGAGAGATCAGCAGCGGCTGCTATGAGGGGCTGCAGTGGCTGGACGAGGCCCGCACCTGTTTCCGCGTGCCCTGGAAGCACTTCGCGCGCAAAGACCTGAGCGAGGCCGACGCGCGCATCTTCAAGGCCTGGGCTGTGGCCCGCGGCAGGTGGCCGCCTAGCAGCAGGGGAGGTGAC---CCGCCCCCCGAGGCTGAGACTGCG---------GAGCGCGCCGGCTGGAAAACCAACTTCCGCTGCGCACTGCGCAGCACGCGTCGCTTCGTGATGCTGCGAGATAACTCGGGGGACCCGGCCGACCCGCACAAGGTGTACGCGCTCAGCCGGGAGCTGTGCTGGCGAGAAGGCCCAGGCACGGACCAGACTGAGGCAGAGGCCCCCGCAGCTGTCCCACCACCACAGGGTGGGCCCCCAGGGCCATTCCTGGCACACACAGATGCTGGA---------------------------------------CTCCAAGCCCCA---------------GGCCCCCTCCCTGCCCCGGCTGGTGACAAGGGGGACCTCCTGCTCCAGGCAGTGCAACAGAGCTGCCTGGCGGACCATCTGCTGACAGCGTCATGGGGGGCAGATCCAGTCCCAACCAAGGCTCCTGGAGAGGGACAAGAAGGGCTTCCCCTGACTGGGGCCTGTGCTGGAGGCCCAGGGCTCCCTGCTGGGGAGCTGTACGGG---------------------------------------------------------------TGGGCAGTAGAGACGACCCCCAGCCCCGGGCCCCAGCCCGCG------GCACTAACGACAGGCGAGGCCGCGGCCCCA---GAGTCCCCGCACCAGGCAGAGCCGTACCTGTCACCCTCCCCAAGCGCCCGCACCGCGGTG---------------------------------------------------CAAGAGCCCAGCCCAGGGACGCTGGACGTGACCATCATGTACAAGGGCCGCACGGTGCTGCAGAAGGTGGTGGGACACCCTAGCTGCACGTTCCTATACGGGCCCCCAGACCCAGCTGTCCGGGCCACAGACCCCCAGCAG---------GTAGCATTCCCCAGCCCTGCCGAGCTCCCGGACCAGAAGCAGCTGCGCTACACGGAGGAACTGCTGCGGCATGTGGCCCCTGGGTTGCACCTGGAGCTTCGGGGGCCACAGCTGTGGGCCCGGCGCATGGGCAAGTGCAAGGTGTACTGGGAGGTGGGCGGCCCCCCAGGCTCCGCCAGCCCCTCCACCCCAGCCTGCCTGCTGCCTCGGAACTGTGACACCCCCATCTTCGACTTCAGAGTCTTCTTCGGAGAGCTGGTGGAATTCCGGGCACGGCAGCGCCGTGGCTCCCCACGCTATACCATCTACCTGGGCTTCGGGCAGGACCTGTCAGCTGGGAGGCCCAAGGAGAAGAGCCTGGTCCTGGTGAAGCTGGAACCCTGGCTGTGCCGAGTGCACCTAGAGGGCACGCAGCGTGAGGGTGTGTCTTCCCTGGATAGCAGCAGCCTCAGCCTCTGCCTGTCC------------------AGCACCAACAGCCTCTATGACGACATCGAGTGCTTCCTT------------ATGGAGCTG------GAGCAGCCCGCCTAG------------------------------------

>Chimpanzee_ENSPTRG00000003126

ATGCCAGTCCCCGAGCGCCCTGCAGCCGGCCCTGACTCTCCGCGGCCGGACACCCGCAGGGCAGCCCCACGCGTGCTGTTCGGAGAGTGGCTCCTTGGAGAGATCAGCAGCGGCTGCTATGAGGGGCTGCAGTGGCTGGACGAGGCCCGCACCTGTTTCCGCGTGCCCTGGAAGCACTTCGCGCGCAAAGACCTGAGCGAGGCCGACGCGCGCATCTTCAAGGCCTGGGCTGTGGCCCGCGGCAGGTGGCCGCCTAGCAGCAGGGGAGGTGAC---CCGCCCCCCGAGGCTGAGACTGCG---------GAGCGCGCCGGCTGGAAAACCAACTTCCGCTGCGCACTGCGCAGCACGCGTCGCTTCGTGATGCTGCGAGATAACTCGGGGGACCCGGCCGACCCGCACAAGGTGTACGCGCTCAGCCGGGAGCTGTGCTGGCGAGAAGGCCCAGGCACGGACCAGACTGAGGCAGAGGCCCCCGCAGCTGTCCCACCACCACAGGGTGGGCCCCCAGGGCCATTCCTGGCACACACAGATGCTGGA---------------------------------------CTCCAAGCCCCA---------------GGCCCCCTCCCTGCCCCGGCTGGTGACAAGGGGGACCTCCTGCTCCAGGCAGTGCAACAGAGCTGCCTGGCGGACCATCTGCTGACAGCGTCATGGGGGGCAGATCCAGTCCCAACCAAGGCTCCTGGAGAGGGACAAGAAGGGCTTCCCCTGACTGGGGCCTGTGCTGGAGGCCCAGGGCTCCCTGCTGGGGAGCTGTACGGG---------------------------------------------------------------TGGGCAGTAGAGACGACCCCCAGCCCCGGGCCCCAGCCCGCG------GCACTAACGACAGGCGAGGCCGCGGCCCCA---GAGTCCCCGCACCAGGCAGAGCCGTACCTGTCACCCTCCCCAAGCGCCCGCACCGCGGTG---------------------------------------------------CAAGAGCCCAGCCCAGGGGCGCTGGACGTGACCATCATGTACAAGGGCCGCACGGTGCTGCAGAAGGTGGTGGGACACCCTAGCTGCACGTTCCTATACGGGCCCCCAGACCCAGCTGTCCGAGCCACAGACCCCCAGCAG---------GTAGCATTCCCCAGCCCTGCCGAGCTCCCGGACCAGAAGCAGCTGCGCTACACGGAGGAACTGCTGCGGCATGTGGCCCCTGGGTTGCACCTGGAGCTTCGGGGGCCACAGCTGTGGGCCCGGCGCATGGGCAAGTGCAAGGTGTACTGGGAGGTGGGCGGCCCCCCAGGCTCCGCCAGCCCCTCCACCCCAGCCTGCCTGCTGCCTCGGAACTGTGACACCCCCATCTTCGACTTCAGAGTCTTCTTCGGAGAGCTGGTGGAATTCCGGGCACGGCAGCGCCGTGGCTCCCCACGCTATACCATCTACCTGGGCTTCGGGCAGGACCTGTCAGCTGGGAGGCCCAAGGAGAAGAGCCTGGTCCTGGTGAAGCTGGAACCCTGGCTGTGCCGAGTGCACCTAGAGGGCACGCAGCGTGAGGGTGTGTCTTCCCTGGATAGCAGCAGCCTCAGCCTCTGCCTGTCC------------------AGCACCAACAGCCTCTATGACGACATCGAGTGCTTCCTT------------ATGGAGCTG------GAGCAGCCCGCCTAG------------------------------------

>Macaca_ENSMMUG00000004014

------------------ATGGCCTTGGCTCCTGAG---------------------AGGGCGGCCCCACGCGTGCTGTTCGGAGAGTGGCTCCTTGGAGAGATCAGCAGCGGCTGCTATGAGGGGCTGCAGTGGCTGGACGAGGCCCGCACCTGCTTCCGCGTGCCCTGGAAGCACTTCGCGCGCAAGGACCTGAGCGAGGCTGACGCGCGCATCTTCAAGGCCTGGGCCGTGGCCCGCGGCAGGTGGCCGCCTAGCAGCAGAGGAGGTGACCCGCCGCCCCCCGAGGCTGAGGCTGCG---------GAGCGCGCCGGCTGGAAAACCAACTTCCGCTGCGCACTGCGCAGCACGCGCCGCTTCGTGATGCTGCGAGATAACTCGGGGGACCCGGCCGACCCGCACAAGGTGTATGCGCTGAGCCCGGAGCTGGGCTGGCGAGAAGGCCCAGGCACGGACCAGACTGAGGCAGAGGGCCCCGCGGCTGTCCGGCCACCGCAGGGCAGGCCCCCAGGGCCATTCCTGGCACACAGAGATGCTGGA---------------------------------------CTCCAAGCCCCA---------------GGCCCCTTCCCTGCCCCAGCTGGTGACAAGGGGGACCTCCTGCTCCAGGCAGTGCAACAGAGCTGCCTGGCGGACCATCTGCTGACAGCGTCATGGGGGGCAGACCCAGTCCCAGCCCAGGCTCCTGGAGAGGGACAAGAGGGTCTTCCCCTGACTGGGGCCTGTGCTGGAGGTCCAGGGCTCCCTGCTGGGGAGCTGTGCACA---------------------------------------------------------------TGGGCAGTAGAAGCAACCCCTAGCCCCGGGCCCCAGCCCACG------GCGCTAATGACAGGCGAGGCCACGGCCCCA---GAGCCCCCGCACCAGGCAGAGCCATACCTGGCACCCTCCCCAAGTGCCTGCACTGTGGTG---------------------------------------------------CAAGAGCCCAGCCCAGGGGCGCTGGACGTGACCATCATGTACAAGGGCCGCACAGTGCTGCAGAAGGTGGTGGGGCACCCGAGCTGCATGTTCCTGTACGGCCCCCCAGACCCAGCTGTCCGGGCCACAGACCCCCAGCAG---------GTAGCATTCCCCAGCCCTGCTGAGCTCCCCGACCAGAAGCAGCTGCGCTACACGGAGGAACTGCTGAGGCACGTGGCCCCTGGGCTGCAGCTGGAGCTTCGGGGGCCACAGCTGTGGGCCCGGCGCATGGGCAAGTGCAAGGTGTACTGGGAGGTGGGTGGCCCCCCGGGCTCCGCCAGCCCCTCCACCCCAGCCTGCCTGCTGCCTCGGAACTGCGACACCCCCATCTTTGACTTCAGAGTCTTCTTCCAAGAGCTGGTGGAATTCCGGGCACGGCAGCGCCGCGGCTCCCCCTGCTATACCATCTACCTGGGCTTCGGGCAGGACCTGTCAGCCAGGAGGCCCAAGGAGAAGAGCCTGGTCCTGGTGAAGCTGGAGCCCTGGCTGTGCCGAGTGCACCTGGAGGGCACGCAACGTGAGGGTGTGTCTTCCCTGGATAGCAGCAGCCTCAGCCTCTGTCTGTCC------------------AGCACCAACAGCCTCTATGATGACATTGAGTGCTTGCTC------------ATGGAGCTG------GAGCAGCCCGTCTAG------------------------------------

>GoldenSnubNoseMonkey_ENSRROG00000029652

------------------ATGGCCTTGGCTCCTGAG---------------------AGGGCGGCCCCACGCGTGCTGTTCGGAGAGTGGCTCCTTGGAGAGATCAGCAGCGGCTGCTACGAGGGGCTGCAGTGGCTGGACGAGGCCCGCACCTGCTTCCGCGTGCCCTGGAAGCACTTCGCGCGCAAGGACCTGAGCGAGGCTGACGCGCGCATCTTCAAGGCCTGGGCCGTGGCCCGCGGCAGGTGGCCGCCTAGCAGCAGAGGCGGTGACCCGCCGCCCCCCGAGGCTGAGGCTGCG---------GAGCGCGCTGGCTGGAAAACCAACTTCCGCTGCGCACTGCACAGCACGCGCCGCTTCGTGATGCTGCGAGACAACTCGGGGGACCCGGCCGACCCGCACAAGGTGTATGCGCTGGGCCCGGAGCTGTGCTGGCGA---GGCCCAGGCACGGACCAGACTGAGGCAGAGGCCCCCGCGGCTGTCCGGCCACCGCAGGGCAGGCCCCCAGACCCATTCCTGGCACACAGAGATGCTGGA---------------------------------------CTCCAAGCCCCA---------------GGCCCCCTCCCTGCCCCAGCTGGTGACAAGGGGGACCTCCTGCTCCAGGCAGTGCAACAGAGCTGCCTGGCGGACCATCTGCTGACAGCGTCATGGGGGGCAGATCCAGTCCCAGCCCAGGCTCCTGGAGAGGGACAAGAGGGTCTTCCCCTGACTGGGGCCTGTGCTGGAGGCCCAGGTTTCCCTGCCGGGGAGCTGTGCGCA---------------------------------------------------------------TGGTCAGTAGAGGCAACCACCAGCCCCGGGCCCCAGCCCGTG------GCCCTTATGACAGGCGAGGCCACGGCCCCA---GAGCCCCCGCACCAGGCAGAGCCGTACCTGGCACCCTCCCCAAGTGCCTGCACTGCAGTG---------------------------------------------------CAAGAGCCCAGTCCAGGGGCACTGGACGTGACCATCATGTACAAGGGCCGCACAGTGCTGCAGAAGGTGGTGGGGCACGCGAGCTGCATGTTCCTGTATGGCCCCCCCGACCCAGCTGTCCAGGCCGCAGACCCCCAGCAG---------GTAGCATTCCCCAGCCCTGCTGAGCTCCCCGACCAGAAGCAGCTGCGCTACACGGAGGAACTGCTGCGGCACGTGGCCCCTGGGCTGCAGCTGGAGCTTCGGGGGCCACAGCTGTGGGCCCGGCGCATGGGCAAGTGCAAGGTGTACTGGGAGGTGGGTGGCCCGCCGGGCTCTGCCAGCCCCTCCACCCCAGCCTGCCTGCTGCCTCGGAACTGCGACACCCCCATCTTTGACTTCAGAGTCTTCTTCCGAGAGCTGGTGGAATTCCGGGCACGGCAGCGCCGCGGCTCCCCATGCTATACCATCTACCTGGGCTTCGGGCAGGACCTGTCAGCCAGGAGGCCCAAGGAGAAGAGCCTGGTCCTTGTGAAGCTGGAGCCCTGGCTGTGCCGAGTGCACCTGGAGGGCACGCAACGTGAGGGTGTGTCTTCCCTGGATAGCAGCAGCCTCAGCCTCTGTCTGTCC------------------AGCACCAACAGCCTCTATGATGACATTGAGTGCTTGCTC------------ATGGAGCTG------GAGCACCCCGTCTAG------------------------------------

# IRF8

>Rabbit_ENSOCUG00000000536

---------------------------------------------------------------------------------------------------------------------------------------------------------------------------------------------------ATGTGTGACCGGAACGGCGGCCGGCGGCTTCGGCAGTGGCTGATCGAGCAGATTGACAGCAACATGTACCCGGGGCTGATCTGGGAAAACGACGAGAAGAGCATGTTCCGGATCCCCTGGAAGCATGCCGGCAAGCAGGACTACAACCAGGAGGTGGACGCGTCCATCTTCAAGGCCTGGGCAGTCTTCAAGGGGAAGTTCAAGGAAGGGGACAAGGCCGAGCCGGCCACTTGGAAGACGAGGCTGCGCTGTGCTCTGAACAAGAGCCCGGATTTTGAAGAAGTGACCGACCGATCCCAGCTGGACATTTCTGAGCCGTACAAAGTTTACCGAATCGTCCCCGAGGAGGAGCAAAAATGCAAACTCGGCGTGGTGCCTCCCGGCGGCGTGAACGAGGTCCCGGAGATGGAGTGCGGGCGCTCGGACATCCAGGAGCTCATCAAGGAGCCGTCCGTGGACGACTACATGGGCATGATCAAGAGGAGCCCCTCCCCGCCGGAGGCCTGCAGGAGCCAGCTGCTCCCAGACTGGTGGGCGCAGCAGCCCAGCGCA---GGCTTGCCGCTGGTGACGGGCTACCCCGCCTATGACGCGCCCCACTCAGCCTTTTCCCAGATGGTGATCAGCTTCTACTACGGGGGCAAGCTCATGGGCCAGACCACCACCACGTGCCCCGAGGGCTGCCGCCTGTCCCTGAGCCAGCCGGGGCTGCCCGGCGGCAAGATGTACGGGCCCGAGGGCCTGGAGCTGGTGCGCTTCCCGCCGGCCGACGCCATCCCCAGCGAGCGGCAGCGGCAGGTGACGCGGAAGCTGTTCGGGCACCTGGAGCGCGGGGTGCTGCTGCACGGCAGCCGGCAGGGCGTGTTCGTCAGGCGCCAGTGCCAGGGCCGCGTCTTCTGCAGCGGCAAC---GCCGTGCCCTGCAAGGGCAGGCCCAACAAGCTGGAGCGCGACGAGGTGGTGCAGGTCTTCGACACGAGCCAGTTCCTGCGAGAGCTGCAGCAGTTCTACAGCAGCCAGAGCCGGCTCCCCGACGGCCGCGTGGTGCTGTGCTTCGGGGAGGAGTTCCCGGACACGGCGCCGCTGCGCTCCAAGCTCATCCTCGTGCAGGTGAGAGCAGCCGCCGGGCCTGTGTCCGGGCTCCCAGACCCCTGCTGGGGTCAGGCTATCCTGGGGCTGGAG---------GATGGGGGATTCCTGCCACCTCCAGCCGTCCTCTGGTGGCCAGGACTCATTTATGCTTGG---TTTACTCTTCAAAATCAAAGTCTCTTCTCCGGAAGGATTCCACCTGTTGCCTTGCCATCGTGTCTGAGGACAGGCAAGGCCGTGGCAAGCCCTTCCTTAACGGTAGCCGAAGGCAAGAGACTCTGGGACTGA---------------------------

>Marmoset_ENSCJAG00000017331

---------------------------------------------------------------------------------------------------------------------------------------------------------------------------------------------------ATGTGTGACCGGAATGGCGGTCGGCGGCTCCGACAGTGGCTGATCGAGCAGATCGACAGTAGCATGTATCCAGGACTGATCTGGGAGAATGACGAGAAGGGCATGTTCCGGATCCCTTGGAAGCACGCTGGCAAGCAAGATTACAATCAGGAAGTGGATGCCTCCATCTTTAAGGCCTGGGCAGTTTTTAAAGGGAAGTTTAAAGAAGGAGACAAAGCTGAACCAGCCACTTGGAAGACGAGGTTACGCTGTGCTCTGAATAAGAGCCCAGATTTTGAGGAAGTGACGGACCGGTCCCAGCTGGACATTTCCGAGCCTTACAAAGTTTACCGAATTGTCCCTGAGGAAGAGCAAAAATGCAAACTAGGCGTGGCAGCTGCTGGCTGCGTGAATGAAGTCACAGAGATGGAGTGCGGTCGCTCTGACATCGAAGAGCTGATCAAGGAGCCTTCTGTGGACGATTACATGGGGATGATCAAAAGGAGCCCTTCCCCGCCAGAGGCCTGTCGGAGCCAGCTCCTTCCAGACTGGTGGGCGCAGCAGCCCAGCACA---GGCGTGCCGCTGGTGATGGGGTACAGTGCCTACGACGCGCACCATTCAGGGTTCTCCCAAATGGTGATCAGCTTCTACTACGGGGGCAAGCTGGTGGGCCAGGCCACCACCACCTGCCCCAAGGGCTGCCTCCTGTCCCTGAGCCAGCCGGGGCTGCCCAGTGCCAAGCTGTGTGGGCCTGAGGGCCTGGAGCTGGTGTGCTTCCCGCCGGCCGACGCCATCCCCAGCGAGCGACAGAGGCAGGTGACACGGAAGCTGTTCGGGCACCTGGAGCGCGGGGTGGTGCTGTACGGCAGCCGGCACGGTGTGTTCGTCAAGCGGCAGTGCCAGGGCCGCGTGTTCTGCAGCGGCAAT---GCCGTGGTGTGCAAGGGCAGACCCAACAAGCTGGAGCGCGACGAGATGGTCCAGGTCTTCGACACCAGCCAGTTCTTCCGAGAGCTGCAGCAGTTCTATAACAGCCAGGGCCGGCTTCCCGACAGCAGGGTGGTGCTGTGCTTTGGGGAAGAGTTTCCGGATATGGCCCCCTTGCGCTCCAAACTCATTCTTGTGCAGGTAAGT------------------------------------GTGGGCAGC---TGTGAGCCCTGCAGCCTG---------GTCTCCTGGCCCCCTCGTGTCAGGATCAAGTTTTGGGCTGGAGAAGAGGCCACAAGGATGGGGTTT------------------------------------CCACCTGTGTGGGAGTCAGGCCTTCAATATGGGTTAAGTAGATTCTTGCCATCTCCGAGCTATCTTCTGGTGTCTGGGACGCACCTAATCTCTGTATGTGTTTTCTCTTTTCTTTTATAA

>GoldenSnubNoseMonkey_ENSRROG00000040814

---------------------------------------------------------------------------------------------------------------------------------------------------------------------------------------------------ATGTGTGACCGAAATGGTGGTCGGCGGCTTCGACAGTGGCTGATCGAGCAGATTGACAGTAGCATGTATCCAGGACTGATTTGGGAGAATGACGAGAAGAGCATGTTCCGGATCCCTTGGAAACACGCTGGCAAGCAAGATTATAATCAGGAAGTGGATGCCTCCATTTTCAAGGCCTGGGCAGTTTTTAAAGGGAAGTTTAAAGAAGGGGACAAAGCTGAACCAGCCACTTGGAAGACGAGGTTACGCTGTGCTTTGAATAAGAGCCCAGATTTTGAGGAAGTGACGGACCGGTCCCAGCTGGACATTTCCGAGCCATACAAAGTTTACCGAATTGTTCCTGAGGAAGAGCAAAAATGCAAACTAGGCGTAGCAACTGCTGGCTGCGTGAATGAAGTCACAGAGATGGAGTGCGGTCGCTCTGAAATCGACGAGCTAATCAAGGAGCCTTCTGTGGACGATTACATGGGGATGATCAAAAGGAGCCCTTCCCCGCCGGAGGCCTGTCGGAGTCAGCTCCTTCCAGACTGGTGGGCGCAGCAGCCCAGCACA---GGCGTGTCGCTGGTGACGGGGTACACCGCCTACGACGCACACCATTCAGCGTTCTCCCAGATGGTGATCAGCTTCTACTATGGGGGCAAGCTGGTGGGCCAGGCCACCACCACCTGCCCCGAGGGCTGCCGCTTGTCCCTGAGCCAGCCTGGGCTACCTGGCACCAAGCTGTATGGGCCCGAGGGCCTGGAGCTGGTGCGCTTCCCACCGGCCGACGCCATCCCCAGCGAGCGACAGAGGCAGGTGACGCGGAAGCTGTTCGGGCACCTGGAGCGCGGGGTGCTGCTGCACAGCAGCCGGCAGGGCGTATTCGTCAAGCGGCTGTGCCAGGGCCGCGTGTTCTGCAGCGGCAAC---GCCGTGGTGTGCAAGGGCAGGCCCAACAAGCTGGAGCGCGACGAGGTGGTCCAGGTCTTCGACACCAGCCAGTTCTTCCGAGAGCTGCAGCAGTTTTATAACAGCCAGGGCCGGCTTCCCGATGGCAGAGTGGTGCTGTGTTTTGGGGAAGAATTTCCAGATATGGCCCCCTTGCGCTCCAAACTCATTCTTGTGCAGGTAAGTATGGGCAGCTTTTTTTTTTTTTTTTTTGAGATGGAGTCTTGCTCTGTTGCCCAAGCTGGAGTGCAATGGCATGACCTTGGCTCACTGCAACCTCCACCT---------------CCCGGG------TTCAAGCGA---TTC------------------------------------TCCTGCCTCAGCCTCCCGAGTTGCTGGAACTACAGGCCCGTGCCACCACGCTCAGCTAATTTTTTGTATTTCTAG---------------------------------------------

>Mouse_ENSMUSG00000041515

---------------------------------------------------------------------------------------------------------------------------------------------------------------------------------------------------ATGTGTGACCGGAACGGCGGGCGGCGGCTGCGGCAGTGGCTGATCGAACAGATCGACAGCAGCATGTACCCGGGGCTGATCTGGGAAAATGATGAGAAGACCATGTTCCGTATCCCCTGGAAGCATGCCGGCAAGCAGGATTACAATCAGGAGGTGGATGCTTCCATCTTCAAGGCCTGGGCAGTTTTTAAAGGGAAGTTTAAAGAGGGAGACAAAGCTGAACCAGCCACGTGGAAGACGAGGTTACGCTGTGCTCTGAACAAGAGCCCAGATTTTGAAGAAGTGACTGACCGGTCCCAGCTGGACATTTCTGAGCCATATAAAGTTTACCGAATTGTCCCCGAGGAAGAACAAAAATGCAAGCTGGGCGTGGCACCTGCAGGCTGCATGAGCGAAGTTCCTGAGATGGAGTGTGGCCGCTCAGAGATTGAGGAGCTGATCAAGGAACCTTCTGTGGATGAGTACATGGGTATGACCAAGAGGAGCCCATCCCCACCAGAGGCCTGCAGGAGCCAGATCCTCCCTGACTGGTGGGTCCAGCAGCCCAGTGCA---GGCCTGCCACTGGTGACCGGATATGCCGCCTATGACACACACCATTCAGCTTTCTCCCAGATGGTCATCAGCTTCTACTACGGGGGCAAGCTGGTGGGCCAGGCCACCACCACCTGCCTTGAAGGCTGCCGTCTCTCCCTGAGCCAGCCGGGGCTGCCT------AAGTTGTATGGGCCGGATGGCCTGGAACCCGTGTGCTTTCCGACGGCCGACACCATCCCCAGTGAGCGGCAGAGGCAGGTGACCCGGAAGCTGTTTGGGCACCTGGAACGTGGCGTGCTACTGCACAGCAACCGCAAGGGCGTGTTCGTGAAGCGGCTGTGCCAGGGCCGCGTGTTCTGCAGCGGCAAC---GCGGTGGTGTGCAAGGGCAGGCCCAACAAGCTGGAGCGGGACGAGGTGGTGCAGGTCTTTGACACCAACCAGTTCATCCGAGAGCTGCAGCAATTCTACGCCACCCAGAGCCGCCTACCTGACAGCAGGGTGGTCCTGTGCTTCGGGGAGGAGTTTCCGGACACTGTGCCCTTGCGCTCCAAACTCATTCTGGTGCAGGTAGAG------CAGCTGTATGCCAGGCAGCTGGTGGAGGAAGCGGGCAAGAGCTGCGGTGCTGGCTCCCTG---------ATGCCAGCCCTGGAGGAGCCCCAG---------------CCGGACCAGGCTTTCCGCATG---TTT------------------------------------CCGGATATC------------TGTACCTCACACCAGAGACCCTTTTTTAGAGAAAATCAACAGATCACCGTCTAA---------------------------------------------

>Rat_ENSRNOG00000017869

---------------------------------------------------------------------ATGTCGCTGGCCCAGATGTCTCCCAAGCCGGCGCCTTTGTGTGCACCTCTGATCTTCCTGTCTGTCGGGTCCCGGATTGCTCTGACTCACAGAGACGTTATACCGAAGTCTGTTTTCTTTCCGAGGATGTGTGACCGGAATGGCGGGCGGCGGCTGCGACAGTGGCTGATCGAACAGATTGACAGCAGCATGTACCCGGGGCTGATCTGGGAGAGTGAGGAGAAGACCCTGTTCCGCATCCCCTGGAAACACGCAGGCAAGCAAGACTACAACCAGGAAGTGGACGCTTCAATCTTCAAGGCCTGGGCAGTTTTTAAAGGGAAGTTTAAAGAGGGAGACAAAGCTGAGCCAGCCACGTGGAAGACACGGTTACGCTGCGCTCTGAACAAGAGCCCGGATTTTGAAGAGGTGACCGACCGGTCCCAGCTGGACATTTCCGAGCCATACAAAGTTTACCGAATCGTCCCCGAGGAAGAGCAAAAATGCAAGCTGGGCGTGGCACCTGCGGGCTGCATGAGCGACGTCACGGAGATGGAGTGTGGCCGTTCAGAGATCGAGGAGCTGATCAAGGAGCCTTCTGTGGATGAATACATGGGAATAGCCAAAAGGAGCCCATCCCCTCCAGAGGCCTGCAGGAGCCAGATCCTCCCTGACTGGTGGGCCCAGCAGCCCAACGCA---GGCCTGCCACTGGTGACTGGGTATACTGCCTATGACGCACACCATTCAGCCTTATCCCAGATGGTGATCAACTTTTACTACGGGGGCAAGCTGGTGGGCCAGGCCACCACCACCTCCCTTGAGGGCTGCCGCCTATCCCTGAGCCAGCCGGGGCTGCCG------AAGCTGTATGGGCCGGACGGCCTGGAGCCCGTGTGCTTCCCGGCGGCGGACACCATCCCCAGTGAGCGGCAGAGGCAGGTGACCCGGAAGCTGTTTGGACACCTGGAGCGCGGCGTGCTCCTGCACAGCAACCGCAAGGGTGTGTTCGTGAAGCGGCTGTGCCAGGGCCGCGTGTTCTGCAGCGGCAAC---GCGGTGGTGTGCAAGGGTAGACCCAACAAGCTGGAGCGGGATGAGGTGGTGCAGGTCTTTGACACCAACATGTTCCTCCGAGACCTGCAGCAGTTCTACGCCACCCAGAGCCGCCTGCCTGATAGCAGGGTGGTTCTGTGCTTTGGGGAGGAGTTTCCAGACACAGCGCCCTTGCGCTCCAAACTCATTCTGGTGCAGGTAGAG------CAGCTGTATGCCAGGCAGATGGTGGAGGAAGCGGGCAAGAGCTGTGGGGCAGGCTCCCTG---------ATGCCAGCCCTGGAGGAACCCCAG---------------CCAGACCAGGCCTTTCGGATG---TTT------------------------------------CCAGATATC------------TGTACCTCACACCAGAGACCCTTTTTCAGAGAAAATCAACAGATCACCGTCTAA---------------------------------------------

>Cow_ENSBTAG00000017824

---------------------------------------------------------------------------------------------------------------------------------------------------------------------------------------------------ATGTGTGACCGGAACGGCGGCCGACGGCTGCGGCAGTGGCTGATTGAGCAGATCGACAGCACCATGTACCCGGGGCTGATCTGGGAGAACGACGAGAAGACCATGTTCCGGATCCCCTGGAAACACGCCGGCAAGCAGGACTACAACCAGGAAGTGGACGCCTCTATCTTCAAGGCCTGGGCCGTTTTTAAAGGGAAGTTTAAGGAAGGGGACAAGGCTGAGCCAGCCACGTGGAAGACGAGGCTACGCTGCGCTTTGAACAAGAGCCCAGATTTTGAGGAAGTGACAGACCGGTCCCAGCTGGACATTTCTGAGCCGTATAAAGTTTACCGCATCGTCCCCGAGGAGGAGCAGAAATGCAAATTAGGTGTGGCGGCCCCGGGCTGTGTGAGCGAGGCTGTGGAGATGGAGTGTGGGCGATCTGAGATCGATGAGCTGATCAAGGAGCCTCCCGGGGATGACTACATGGGGATGGTGAAGAGGAGCCCCTCACCGCCCGAGGCCTGCCGGAGCCAGCTCCTCCCGGACTGGTGGGTGCAGCAGCCCAGCGCAGCAGGCCTGCCACTGGTGCCGGGGTACAGCGCCTATGACGCCCACCACTCAGCCTTCTCCCAGATGGTCATCAGCTTCTACTACGGGGGCAAGCTGGTGGGCCAGACTACCACCACGTGCCCCGAGGGCTGCCGCCTGTCGCTGGGTCAGCCTGGCCTGCTGGGTGGGAAGCTGTACGGGCCGGAGGGCCTGGAGCTGGTGCGCTTCCCACCGGCTGACACCATCCCCAGCGAGCGGCAGCGGCAGGTGACGCGGAAGCTGTTCGGGCACCTGGAGCGCGGCATCCTCCTGCACAGCAGCAGGCAGGGCGTGCTGGTCAAGCGGCTGTGCCAGGGCCGCGTGTTCTACAGCGGGAAC---GCCGCGCCCGCCCGCGACCGGCCCAACAAGCTGGAGCGCGACGAGGTGGTCAGGGTCTTCGACACCAGCCAGTTCTTCCGAGAGCTGCAGCAGTTCTACAACAGCCAGAGCCGGCTGCCCGACAGCAGAGTGGTGCTATGCTTCGGAGAGGAGTTTCCGGACACGACCCCACTGCGCTCCAAGCTCATCCTTGTCCAGGTGGAG------CAGCTATACGTCCGGCAGCTGGTGGAAGAAGCGGGGAAGGGCTGCGGCCCCGGATCCTTG---------ATGCAGGCCCCCGAGGAGCCCCCA---------------CCAGACCAGGTCTTCCGGATG---TTT------------------------------------CCAGAGATC------------TGTGCCTCCCACCAGAGACCTTTCTTCAGAGAAAACCAACAGATCACAGTTTAA---------------------------------------------

>Horse_ENSECAG00000015449

ATGCCCATACTGGACAAAATTTTGCTTTTTACGTCAGGAGCCTCCTGGCGCGGGGACTGTAGTTTACGAACCTGTGCTAGGGGCTCATCGAGATGCTGCGGAGCAGAAACGGCTCGTTTGATTGACAGATCCGCAGACCCCAATCCCCTGTTGTGTTCTGGCCCCTCTCGCCCTCCACACAGCTCGTTCCTCAAGATGTGTGACAGGAATGGCGGCCGGCGGCTTCGGCAGTGGCTGATCGAGCAAATCGACAGCAACATGTACCCAGGGCTGATTTGGGAAAATGATGAGAAGAGCATGTTCCGGATCCCTTGGAAACACGCCGGCAAACAAGATTATAATCAGGAGGTGGACGCCTCCATTTTCAAGGCCTGGGCAGTTTTTAAAGGGAAGTTTAAGGAAGGGGACAAAGCTGAACCAGCCACTTGGAAGACGAGGCTCCGCTGTGCTTTGAACAAGAGCCCAGATTTTGAGGAAGTAACAGACCGGTCCCAGCTGGACATTTCCGAGCCATACAAAGTTTACCGCATCGTCCCCGAGGAAGAGCAAAAATGCAAATTAGGCACGGCGACCCCTGGCTGTGTGAATGAAGTCCCGGAGATGGAGTGCGGCCGCTCCGAAATCGACGAGCTGATCAAGGAGCCGTCGGTGGATGAGTACATGGGGATGGTGAAGAGGAGTCCTTCCCCGCCGGAGGCCTGCCGGAGTCAGCTCTTCCCAGACTGGTGGGCGCAGCAGACCAGCGCAGGAGGCTTGCCCCTGGTGCCGGGCTACACCACCTATGACCCACACCATACAGCCTTCTCCCAGATGGTCATCAGCTTCTACTACGGGGGCAAGCTGGTGGGCCAGACCACCACCACCTGCCCGGACGGCTGCCGCCTGTCCCTGAGCCAGGCCGGCCTGCCCAGCACCAAGCTGTACGGACCCGAGGGCCTGGAGCTGGTGCGCTTCCCGCCGGCTGATGCCATCCCCAGCGAGCGGCAGAGGCAGGTGACGCGGAAGCTGTTCGGGCACCTGGAGCGCGGCGTGCTTCTGCACAGCAGCCGGCAGGGCGTGCTGGTCAAGCGGCTGTGCCAGGGCCGTGTGTTTGCCAGCGGCAAC---GCCGTGGTGTGCAAGGACAGGCCCAACAAGCTGGAGCGCGACGTGGTGGTCAAGGTCTTCGACACCAGCCAGTTCTTCCGAGACCTGCAACACTTCTACAACGGCCAGGGCCGGCTCCCCGACAGCAGGGCAGTGCTGTGCTTTGGCGAGGAGTTCCCCGACATGACGCCCTTGCGCTCCAAACTCATCCTCGTGCAGATCGAG------CAGGTCTATGCCCGGCAGCTGGTGGAAGAAGCTGGGAAGAGCTGCGGGGCGGGCTCCATG---------ATGCAGGCTCCTGAGGAGCCCCAG---------------CCGGACCAGGTCTTCCGGATG---TTT------------------------------------CCAGATATT------------TGTGCCTCACACCAGAGACCCTTTTTCAGAGAAAACCAGCAGATCACCGTTTAA---------------------------------------------

>Dog_ENSCAFG00845004258

---------------------------------------------------------------------------------------------ATGGGGCTCAGTCAATGGCAGGCTGGGATGCCCTGCTTCCCTGGCTGGGACGCCCCAGCAAGGTTGGCACCTGCCAAGGTGGAGGCCATGGCTCTCTGGAGGATGTGTGACCGGAACGGTGGCCGGCGGCTTCGGCAGTGGTTGATTGAGCAGATTGACAGCAACATGTACCCGGGGCTGATTTGGGAAAATGATGAGAAGAGCATGTTCAGGATCCCTTGGAAACATGCTGGCAAGCAAGATTATAACCAGGAAGTGGATGCATCCATTTTTAAGGCCTGGGCAGTTTTCAAAGGGAAATTTAAGGAAGGAGACAAAGCTGAACCAGCCACTTGGAAGACAAGGCTACGCTGTGCTTTGAACAAGAGCCCAGATTTTGAGGAAGTGACAGACCGGTCCCAGCTGGACATCTCTGAGCCATACAAAGTTTACCGGATTGTCCCCGAGGAGGAGCAAAAATGCAAATTAGGCATGGCGACTCCTGGCTGTGTGAGCGAGATCACTGAGATGGAGTGTGGTCGTTCTGAAATCGATGAGCTCATCAAAGAGCCCTCTGTGGACGATTACATGGGGGTGGTCAAAAGGAGCCCTTCCCCGCCGGAGGCCTGTAGGAACCAGCTCCTCTCAGACTGGTGGGTGCAGCAGCCCAGTGCA---GGCCTCCCACTAGTGACGGGGTACACTGCCTATGATGCGCACCATTCAGCTTTCTCCCAGATGGTCATCAGCTTCTACTACGGGGGCAAGCTGGTGGGCCAGACCACCACCACCTGCCCCGAGGGCTGCCGCCTGTCCCTGAGCCAGCCAGGCCTGCCCAGTGCCAAGCTGTACAGTCCCGAGGGCCTGGAGCTGGTGCGCTTCCCACCAGCGGACGCCATCCCCAGCGAGCGGCAGCGGCAGGTGACGCGGAAGCTGTTCGGGCACCTGGAGCGCGGCGTGCTCCTGCACAGCAGCCGGCAGGGCGTGCTTGTCAGGCGGCTGTGTCAGGGCCGCGTGTTCTGCAGCGGCAAC---GCCGTGGTGTGCAAGGACAGGCCCAACAAGCTGGAGCGTGACGAGGTGGTCAAGGTCTTCGACACCAGCCAGTTCTTCCGAGAGCTGCAGCAGTTCTATAACCACCAGAGTCGGCTTCCCGACAGCAGGGTGGTGCTGTGCTTCGGAGAAGAGTTTCCAGATATGACCCCCTTGCGCTCCAAACTCATTCTGGTGCAGATCGAG------CAGCTCTACGCCCGGCAGCTGGTGGAAGAAGCTGGGAAGAGCTGTGGCGCCAGCTCGATG---------GTGCAGCCTCCCGAGGAATCCCAG---------------CCAGACCAGGTCTTCCGGATG---TTT------------------------------------CCAGACATC------------TGTGCCTCACACCAGAGACCTTTTTTCAGAGAAAACCAGCAGATCACGGTTTAA---------------------------------------------

>Megabat_ENSPVAG00000003758

---------------------------------------------------------------------------------------------------------------------------------------------------------------------------------------------------ATGTGTGACCGGAACGGGGGCCGACGGCTTCGGCAGTGGCTGATTGAGCAAATTGACAGCAACATGTACCCAGGGCTGATTTGGGAAAATGATGAGAAGAGTATGTTCCGGATCCCTTGGAAACATGCTGGCAAACAAGATTATAATCAGGAAGTGGACGCCTCCATTTTCAAGGCCTGGGCAGTTTTTAAAGGGAAATTTAAGGAAGGTGACAAAGCTGAACCAGCCACGTGGAAAACAAGGCTACGCTGTGCTTTGAACAAGAGTCCGGATTTTGAGGAAGTAACAGACCGGTCCCAGCTGGACATTTCTGAGCCATACAAAGTTTACCGCATCGTCCCCGAGGAAGAGCAAAAATGCAAATTAGGCATGGCGACTCCTGGCTGTGTGAATGAAGTCACGGACATGGAGTGTGGGCGCTCTGAAATTGATGAGCTGATCAAGGAACCTTCTGTGGACGACTACATGGGGATGGTCAAAAGGAGCCCTTCCCCACCTGAGGCCTGCAGGAGTCAGCTCCTCCCAGACTGGTGGATGCAGCAGCCCAGCGCA---GGCTTGCCGCTGGTGACAGGATAC---GGCTACGACACGCACCATTCAGCCTTCTCTCAGATGGTCATCAGCTTCTACTATGGGGGCAAGCTGGTGGGCCAGACCACCACCACCTGCCCCGAGGGCTGCCGCCTGTCCCTGAGCCAGCCGGGTCTGCCCAGTGCCAAGATGTACGGGCCCGAGGGCCTGGAGCTGGTGCGCTTCCCGCCGGCTGACACCATTCCCAGCGAGCGGCAGAGGCAGGTGACTCGGAAGCTGTTTGGGCACCTGGAGCGTGGCGTGCTGCTGCACAGCAGCCGGCAGGGCGTGCTGGTCAAGCGGCTGTGCCAGGGCCGCGTGTTCTGCAGTGGCAAC---GCTGTGCTGTGCAAGGACAGGCCCAACAAGCTGGAGCGTGACGAGGTGGTCAAGGTCTTCGACACCAGCCACTTTTTGCGAGAGCTGCAGCAGTTCTATGGCAACCAGAGCCGGCTGCCAGACAGCAGGGTCATGCTGTGCTTCGGAGAAGAGTTCCCAGACATGACCCCCTTGCGCTCCAAACTCATTCTCGTGCAGATTGAG------CAACTCTACGTCCGGCAGCTGGTGGAAGAAGCTGGGAAGAACTGTGGTGCTGGGACCATG---------ATGCAGGCTCCCGAGGAGCCTCAG---------------CCAGACCAGGTCTTCCGGATG---TTT------------------------------------CCAGATATT------------TGTGCCTCCCACCAGAGATCCTTTTTCAGAGAAAACCAACAAATCACAGTTTAA---------------------------------------------

>Elephant_ENSLAFG00000010973

---------------------------------------------------------------------------------------------------------------------------------------------------------------------------------------------------ATGTGTGACCGTAATGGCGGGAGGCGGCTGCGGCAGTGGCTCGTCGAACAAATCGACAGCAACATGTATCCAGGGCTGATTTGGGAAAATGACGAGAAGAGCATGTTCCGGATCCCTTGGAAGCACGCGGGCAAACAAGATTACAATCAGGAAGTTGACGCCTCCATTTTCAAGGCCTGGGCAGTTTTCAAAGGGAAGTTTAAGGAAGGGGACAAAGCTGAACCAGCTACTTGGAAGACGAGGTTACGCTGTGCCTTGAATAAGAGTCCAGACTTTGAGGAAGTGACAGAGCGGTCCCAGCTGGACATTTCTGAGCCATATAAAGTTTACCGGATCGTCCCCGAGGAGGAGCAGAAATGCAAATTAGGCATGGCTGCCCCTGGCTGTGTGAACGAAGTCACGGAGATGGAATGTGGCCGCTCAGAAATCGATGACCTGATTAAGGAGCCTTCTGTGGATGATTATATGGGCATGATCAAAAGGAGCCCTTCCCCGCCGGAGGCCTGCAGGAGCCAGCTCCTTCCAGACTGGTGGACACAGCAAGCCGGCCCA---GCTTTACCACTGGTGACCGGGTATGCCGCCTATGATGCGCACCACTCAGCCTTCGCCCAGATGGTGATCAGCTTCTACTATGGTGGCAAGCTGGTGGGCCAGACCACGACCAGCTGCCCTGAGGGCTGCCGCCTTTCCCTGGGCCAGCCAGGCCTGCCTGGTGCCAAGTTGTACGGGCCCGAGGGCCTGGAGATTGTCCGCTTCCCGCCAGCCGATGCCATTCCCAGCGAGCGGCAGCGGCAGGTGACGCGGAAGCTGTTCGGGCACCTGGAGCGGGGTGTCCTGCTGCACAGCAGCCGACAGGGCATCCTGGTCAAGCGGCTGTGCCAGGGCCGTGTGTTCTGCAGTGGCAAC---GCCGTGGCCTGCAAGGACAGGCCCAACAAGCTGGAGCGTGACGAAGTGGTCAAGGTCTTTGACACCAGTCAGTTCTTCCGAGAGCTGCAGCAGTTCTATAACAACCAGAGCCGGCTCCCTGACAGCAGGGTGGTGCTATGCTTTGGAGAAGAGTTTCCAGATATGGCCCCTTTGCGCTCCAAACTCATTCTTGTGCAGATTGAG------CAGCTCTACGTTCGGCAGCTGGTCGAAGAAGCTGGGAAAAGCTGTGGTGCCGGCTCCGCG---------ATGCAGGTTCCTGAGGAGCCCCAG---------------CCGGACCAGGTCTTCCGGATG---TTT------------------------------------CCGGATATT------------TGTGCTTCGCACCAGAGACCGTTTTTCAGAGAAAACCAACAGATCACTGTCTAA---------------------------------------------

>Tarsier_ENSTSYG00000037522

---------------------------------------------------------------------------------------------------------------------------------------------------------------------------------------------------ATGTGCGACCGGAATGGCGGGCGTCGGCTTCGACAGTGGCTCATTGAACAGATCGACAGTAACATGTACCCAGGCCTGATCTGGGAGAACGATGAGAAGAGCTTGTTCCGGATCCCGTGGAAACATGCTGGAAAGCAAGATTATAACCAAGAAGTGGACGCCTCCATCTTCAAGGCCTGGGCAGTTTTTAAAGGGAAGTTTAAAGAAGGGGACAAAGCTGAACCAGCCACTTGGAAGACGAGGTTACGCTGTGCTTTGAACAAGAGCCCTGATTTTGAGGAAGTGACAGACCGGTCCCAGCTGGACATTTCCGAGCCATACAAAGTGTACCGAATCGTCCCCGAGGAAGAGCAAAAATGCAAACTAGGCGTGGTGACTCCTGGCTGCATGAACGAAGTCATAGATATGGAATGTGGGCGCTCTGAGGTCGACGAGCTGGTCAAGGAGCCTCCTGTGGATGATTACATGGGG---ATCAAAAGGAGCCCCTCCCCGCCTGAGGCCTGTCGGAGTCAGCTCCTTCCAGACTGGTGGGCACAGCAGCCCAGCGCA---GGCTTGTCGCTGGTGACGGGGTACACAGCCTACGACACGCACCATTCAGCCTTCTCCCAGATGGTGATCAGCTTCTACTACGGGGGCAAGCTGGTAGGCCAGGCCACCACCACCTGCCCCGAGGGCTGCCGCCTGTCCCTGAGCCAGCCGGGGCTGCCGGGTGCCAAGCTGTACGGGCCTGAGGGCCTGGAGCTGGTGCGCTTCCCGCCGGCCGACACCATCCCCAGCGAGCGGCAGAGGCAGGTGACGCGGAAGCTGTTCGGGCACCTGGAGCGCGGGGTGCTGCTACACAGCAGCCGGCAGGGTGTGTTCATCAAGCGGCTGTGCCAGGGCCGCGTGTTCTGCAGCGGCAACGCGGCCGCCGCATGCAAGGGCAGGCCCAACAAGCTGGAGCGCGACGAGGTGGTCCAGGTCTTCGACACCAGCCAGTTCTTCCGAGAGTTGCAGCAGTTCTATAACAGCCAGAGCCGGCTTCCAGACAGCCGGGTGGTGTTGTGCTTCGGGGAAGAGTTTCCAGACACAGTCCCCCTGCGCTCCAAG---------------ATTGAG------CAACTCTACGTCCGGCAGCTGATGGAGGAAGCCGGGAAGAGCTGCGGCGCCGGCTCCCTG---------ATGCAGGTGCCCGAGGAGCCCCCG---------------CCGGACCAGGTCTTCCGGATG---TTT------------------------------------CCGGACATT------------TGTGCCTCACACCAGAGACCCTTTTTCAGAGAAAACCAACAGATCACCGTCTGA---------------------------------------------

>MasNightMonkey_ENSANAG00000036367

---------------------------------------------------------------------------------------------------------------------------------------------------------------------------------------------------ATGTGTGACCGGAATGGCGGTCGGCGGCTCCGACAGTGGCTGATCGAGCAGATTGACAGTAGCATGTATCCAGGACTGATTTGGGAGAATGACGAGAAGAGCATGTTCCGGATCCCTTGGAAACATGCTGGCAAGCAAGATTATAATCAGGAAGTGGATGCCTCCATTTTCAAGGCCTGGGCAGTTTTTAAAGGGAAGTTTAAAGAAGGAGACAAAGCTGAACCAGCCACTTGGAAGACGAGGTTACGCTGTGCTTTGAATAAGAGCCCAGATTTTGAGGAAGTGACGGACCGGTCCCAGCTGGACATTTCCGAGCCTTACAAAGTTTACCGAATTGTCCCTGAGGAAGAGCAAAAATGCAAACTAGGCGTGGCAGCTGCTGGCTGCGTGAATGAAGTCACAGAGATGGAGTGCGGTCGCTCTGACATCGAAGAGCTGATCAAGGAGCCTACTGTGGACGATTACATGGGGATGATCAAAAGGAGCCCTTCCCCGCCAGAGGCCTGTCGGAGCCAGCTCCTTCCAGACTGGTGGGCGCAGCAGCCCAGCACA---GGTGTGCCGCTGGTGACGGGGTACACTGCCTACGACGCACACCATTCAGCGTTCTCCCAGATGGTAATCAGCTTCTACTACGGGGGCAAGCTGGTGGGCCAGGCCACCACCACCTGCCCCGAGGGCTGCCGCCTGTCCCTGAGCCAGCCGGGGCTGCCCAATGCCAAGCTGTGCGGGCCCGAGGGCCTGGAGCTGGTGCGCTTCCCGCCGGCCGACGCCATCCCCAGCGAGCGACAGAGGCAGGTGACGCGGAAGCTGTTCGGGCACCTGGAGCGCGGGGTGCTGCTGTACAGCAACCGGCAGGGTGTGTTCGTCAGGCGGCTGTGCCAGGGCCGCGTGTTCTGCAGTGGCAAT---GCCGTGGTGTGCAAGGGCAGACCCAACAAGCTGGAGCGCGACGAGATGGTCCAGGTCTTCGACACCAGCCAGTTCTTCCGAGAGCTGCAGCAGTTCTACAACAGCCAGAGCCGGTTTCCCGACAGCAGGGTTGTGCTGTGCTTTGGGGAAGAGTTTCCGGATATGGCCCCCTTGCGCTCCAAACTCATTCTTGTGCAGATCGAG------CAGCTCTACGTCCGGCAGCTGGCAGAAGAGGCCGGGAAGAGCTGCGGCGCCGGCTCCGTG---------ATACAGGCCCCCGAGGAGTCCCTG---------------CCGGACCAGGTCTTCCGGATG---TTT------------------------------------CCAGATATC------------TGTGCCTCACACCAGAGACCGTTTTTCAGAGAAAACCAGCAGATCACCGTCTAA---------------------------------------------

>Gibbon_ENSNLEG00000011429

---------------------------------------------------------------------------------------------------------------------------------------------------------------------------------------------------ATGTGTGACCGGAATGGTGGTCGGCGGCTTCGGCAGTGGCTGATCGAGCAGATTGACAGTAGCATGTATCCAGGACTGATTTGGGAGAATGACGAGAAGAGCATGTTCCGGATCCCTTGGAAGCACGCTGGCAAGCAAGATTATAATCAGGAAGTGGACGCCTCCATTTTTAAGGCCTGGGCAGTTTTTAAAGGGAAGTTTAAAGAAGGGGACAAAGCTGAACCAGCCACTTGGAAGACGAGGTTACGCTGTGCTTTGAATAAGAGCCCAGATTTTGAGGAAGTGACGGACCGGTCCCAACTGGACATTTCCGAGCCATACAAAGTTTACCGAATTGTTCCTGAGGAAGAGCAAAAATGCAAACTAGGCATGGCAACTGCTGGCTGCGTGAATGAAGTCACAGAGATGGAGTGCGGTCGCTCTGAAATCGACGACCTGATCAAGGAGCCTTCTGTGGATGATTACATGGGGATGATCAAAAGGAGCCCTTCTCCGCCGGAGGCCTGTCGGAGTCAGCTCCTTCCAGACTGGTGGGCACAGCAGCCCAGCACA---GGCGTGCCGCTGGTGACGGGGTACACAGCCTACGACGCGCACCATTCAGCATTCTCCCAGATGGTGATCAGCTTCTACTATGGGGGCAAGCTGGTGGGCCAGGCCACCACCACCTGCCCCGAGGGCTGCCGCCTGTCCCTGAGCCAGCCCGGGCTGCCCGGCACCAAGCTGTATGGGCCTGAGGGCCTGGAGCTGGTGCGCTTCCCGCCGGCCGACGCCATCCCCAGCGAGCGACAGAGGCAGGTGACGCGGAAGCTGTTCGGGCACCTGGAGCGCGGGGTGCTGCTGCACAGCAGCCGGCAGGGAGTGTTCGTCAAGCGGCTGTGCCAGGGCCGCGTGTTCTGCAGCGGCAAC---GCCGTGGTGTGCAAGGGCAGGCCCAACAAGCTGGAGCGTGATGAGGTGGTCCAGGTCTTCGACACCAGCCAGTTCTTCCGAGAGCTGCAGCAGTTCTATAACAGCCAGGGCCGGCTTCCTGACGGCAGGGTGGTGCTGTGTTTCGGGGAAGAGTTTCCGGATATGGCCCCCTTGCGCTCCAAACTCATTCTCGTGCAGATTGAG------CAGCTCTATGTCCGGCAACTGGCAGAAGAGGCTGGGAAGAGCTGCAGAGCCGGCTCTGTG---------ATGCAGGCCCCCGAGGAGCCGCCG---------------CCAGACCAGATCTTCCGGATG---TTT------------------------------------CCAGATATT------------TGTGCCTCACACCAGAGACCGTTTTTCAGAGAAAACCAACAGATCACCGTCTAA---------------------------------------------

>Macaca_ENSMMUG00000008125

---------------------------------------------------------------------------------------------------------------------------------------------------------------------ATGGCAGGTGTCCCGGAGTCCCTGAATCTGATGTGTGACCGAAATGGTGGTCGGCGGCTTCGACAGTGGCTGATCGAGCAGATTGACAGTAGCATGTATCCAGGACTGATTTGGGAGAATGACGAGAAGAGCATGTTCCGGATCCCTTGGAAACACGCCGGCAAGCAAGATTATAATCAGGAAGTGGATGCCTCCATTTTCAAGGCCTGGGCAGTTTTTAAAGGGAAGTTTAAAGAAGGGGACAAAGCTGAACCAGCCACTTGGAAGACGAGGTTACGCTGTGCTTTGAATAAGAGCCCAGATTTTGAGGAAGTGACGGACCGGTCCCAGCTGGACATTTCCGAGCCATACAAAGTTTACCGAATTGTTCCTGAGGAAGAGCAAAAATGCAAACTAGGCGTGGCAACTGCTGGCTGCGTGAATGAAGTCACGGAGATGGAGTGCGGTCGCTCTGAAATCGACGAGCTGATCAAGGAGCCTTCTGTGGACGATTACATGGGGATGATCAAAAGGAGCCCTTCCCCGCCGGAGGCCTGTCGGAGTCAGCTCCTTCCAGACTGGTGGGCGCAGCAGCCCAGCACA---GGCGTGTCGCTGGTGACGGGGTACACCGCCTACGACGCGCACCATTCAGCATTCTCCCAGATGGTGATCAGCTTCTACTATGGGGGCAAGCTGGTGGGCCAGGCCACCACCACCTGCCCCGAGGGCTGCCGCCTGTCCCTGAGCCAGCCTGGGCTACCTGGCACCAAGCTGTATGGGCCCGAGGGCCTGGAGCTGGTGCGCTTCCCACCGGCCGACGCCATCCCCAGCGAGCGACAGAGGCAGGTGACGCGGAAGCTGTTCGGGCACCTGGAGCGCGGGGTGCTGCTGCACAGCAGCCGGCAGGGCGTGTTCGTCAAGCGGCTGTGCCAGGGCCGCGTGTTCTGCAGCGGCAAC---GCCGTGGTGTGCAAGGGCAGGCCCAACAAGCTGGAGCGCGACGAGGTGGTCCAGGTCTTCGACACCAGCCAGTTCTTCCGAGAGCTGCAGCAGTTTTATAACAGCCAGGGCCGGCTTCCCGACGGCAGGGTGGTGCTGTGCTTTGGGGAAGAGTTTCCGGATATGGCCCCCTTGCGCTCCAAACTCATTCTCGTGCAGATTGAG------CAGCTCTATGTCCGGCAGCTGGCAGAAGAGGCTGGGAAGAGCTGTGGAGCCGGCTCCGTG---------ATGCAGGCCCCCGAGGAGCCGCCG---------------CCAGACCACGTCTTCCGGATG---TTT------------------------------------CCAGATATT------------TGTGCCTCACACCAGAGACCGTTTTTCAGAGAAAACCAACAGATCACCGTCTAA---------------------------------------------

>Gorilla_ENSGGOG00000028251

---------------------------------------------------------------------------------------------------------------------------------------------------------------------------------------------------ATGTGTGACCGGAATGGTGGTCGGCGGCTTCGACAGTGGCTGATCGAGCAGATTGACAGTAGCATGTATCCAGGACTGATTTGGGAGAATGACGAGAAGAGCATGTTCCGGATCCCTTGGAAACATGCTGGCAAGCAAGATTATAATCAGGAAGTGGATGCCTCCATTTTTAAGGCCTGGGCAGTTTTTAAAGGGAAGTTTAAAGAAGGGGACAAAGCTGAACCAGCCACTTGGAAGACGAGATTACGCTGTGCTTTGAATAAGAGCCCAGATTTTGAGGAAGTGACGGACCGGTCCCAACTGGACATTTCCGAGCCATACAAAGTTTACCGAATTGTTCCTGAGGAAGAGCAAAAATGCAAACTAGGCGTGGCAACTGCTGGCTGCGTGAATGAAGTTACAGAGATGGAGTGTGGTCGCTCTGAAATCGACGAGCTGATCAAGGAGCCTTCTGTGGACGATTACATGGGGATGATCAAAAGGAGCCCTTCCCCGCCGGAGGCCTGTCGGAGTCAGCTCCTTCCAGACTGGTGGGCGCAGCAGCCCAGCACA---GGTGTGCCACTGGTGACGGGGTACACCACCTACGACGCGCACCATTCAGCGTTCTCCCAGATGGTGATCAGCTTCTACTATGGGGGCAAGCTGGTGGGCCAGGCCACCACCACCTGCCCCGAGGGCTGCCGCCTGTCCCTGAGCCAGCCTGGGCTGCCCGGCACCAAGCTGTATGGGCCCGAGGGCCTGGAGCTGGTGCGCTTCCCGCCGGCCGACGCCATCCCCAGCGAGCGACAGAGGCAGGTGACGCGGAAGCTGTTCGGGCACCTGGAGCGCGGGGTGCTGCTGCACAGCAGCCGGCAGGGCGTGTTCGTCAAGCGGCTGTGCCAGGGCCGCGTGTTCTGCAGCGGCAAT---GCCGTGGTGTGCAAAGGCAGGCCCAACAAGCTGGAGCGTGATGAGGTGGTCCAGGTCTTCGACACCAGCCAGTTCTTCCGAGAGCTGCAGCAGTTCTATAACAGCCAGGGCCGGCTTCCTGACGGCAGGGTGGTGCTGTGCTTTGGGGAAGAGTTTCCGGATATGGCCCCCTTGCGCTCCAAACTCATTCTCGTGCAGATTGAG------CAGCTGTATGTCCGGCAACTGGCAGAAGAGGCTGGGAAGAGCTGTGGAGCCGGCTCTGTG---------ATGCAGGCCCCCGAGGAGCCGCCG---------------CCAGACCAGGTCTTCCGGATG---TTT------------------------------------CCAGATGTT------------TGTGCCTCACACCAGAGATCGTTTTTCAGAGAAAACCAACAGATCACCGTCTAA---------------------------------------------

>Bonobo_ENSPPAG00000035056

---------------------------------------------------------------------------------------------------------------------------------------------------------------------------------------------------ATGTGTGACCGGAATGGTGGTCGGCGGCTTCGACAGTGGCTGATCGAGCAGATTGACAGTAGCATGTATCCAGGACTGATTTGGGAGAATGACGAGAAGAGCATGTTCCGGATCCCTTGGAAACACGCTGGCAAGCAAGATTATAATCAGGAAGTGGATGCCTCCATTTTTAAGGCCTGGGCAGTTTTTAAAGGGAAGTTTAAAGAAGGGGACAAAGCTGAACCAGCCACTTGGAAGACGAGGTTACGCTGTGCTTTGAATAAGAGCCCAGATTTTGAGGAAGTGACGGACCGGTCCCAACTGGACATTTCCGAGCCATACAAAGTTTACCGAATTGTTCCTGAGGAAGAGCAAAAATGCAAACTAGGCGTGGCAACTGCTGGCTGCGTGAATGAAGTTACAGAGATGGAGTGCGGTCGCTCTGAAATCGACGAGCTGATCAAGGAGCCTTCTGTGGACGATTACATGGGGATGATCAAAAGGAGCCCTTCCCCGCCGGAGGCCTGTCGGAGTCAGCTCCTTCCAGACTGGTGGGCGCAGCAGCCCAGCACA---GGCGTGCCGCTGGTGACGGGGTACACCACCTACGACGCGCACCATTCAGCGTTCTCCCAGATGGTGATCAGCTTCTACTATGGGGGCAAGCTGGTGGGCCAGGCCACCACCACCTGCCCCGAGGGCTGCCGCCTGTCCCTGAGCCAGCCCGGGCTGCCCGGCACCAAGCTGTATGGGCCCGAGGGCCTGGAGCTGGTGCGCTTCCCACCGGCCGACGCCATCCCCAGCGAGCGACAGAGGCAGGTGACGCGGAAGCTGTTCGGGCACCTGGAGCGCGGGGTGCTGCTGCACAGCAGCCGGCAGGGTGTGTTCGTCAAGCGGCTGTGCCAGGGCCGCGTGTTCTGCAGCGGCAAC---GCCGTGGTGTGCAAAGGCAGGCCCAACAAGCTGGAGCGTGATGAGGTGGTCCAGGTCTTCGACACCAGCCAGTTCTTTCGAGAGCTGCAGCAGTTCTATAACAGCCAGGGCCGGCTTCCTGACGGCAGGGTGGTGCTGTGCTTTGGGGAAGAGTTTCCGGATATGGCCCCCTTGCGCTCCAAACTCATTCTCGTGCAGATTGAG------CAGCTGTATGTCCGGCAACTGGCAGAAGAGGCTGGGAAGAGCTGTGGAGCCGGCTCTGTG---------ATGCAGGCCCCCGAGGAGCCGCCG---------------CCAGACCAGGTCTTCCGGATG---TTT------------------------------------CCAGATATT------------TGTGCCTCACACCAGAGATCGTTTTTCAGAGAAAACCAACAGATCACCGTCTAA---------------------------------------------

>Chimpanzee_ENSPTRG00000008435

---------------------------------------------------------------------------------------------------------------------------------------------------------------------------------------------------ATGTGTGACCGGAATGGTGGTCGGCGGCTTCGACAGTGGCTGATCGAGCAGATTGACAGTAGCATGTATCCAGGACTGATCTGGGAGAATGACGAGAAGAGCATGTTCCGGATCCCTTGGAAACACGCTGGCAAGCAAGATTATAATCAGGAAGTGGATGCCTCCATTTTTAAGGCCTGGGCAGTTTTTAAAGGGAAGTTTAAAGAAGGGGACAAAGCTGAACCAGCCACTTGGAAGACGAGGTTACGCTGTGCTTTGAATAAGAGCCCAGATTTTGAGGAAGTGACGGACCGGTCCCAACTGGACATTTCCGAGCCATACAAAGTTTACCGAATTGTTCCTGAGGAAGAGCAAAAATGCAAACTAGGCGTGGCAACTGCTGGCTGCGTGAATGAAGTTACAGAGATGGAGTGCGGTCGCTCTGAAATCGACGAGCTGATCAAGGAGCCTTCTGTGGACGATTACATGGGGATGATCAAAAGGAGCCCTTCCCCGCCGGAGGCCTGTCGGAGTCAGCTCCTTCCAGACTGGTGGGCGCAGCAGCCCAGCACA---GGCGTGCCGCTGGTGACGGGGTACACCACCTACGACGCGCACCATTCAGCGTTCTCCCAGATGGTGATCAGCTTCTACTATGGGGGCAAGCTGGTGGGCCAGGCCACCACCACCTGCCCCGAGGGCTGCCGCCTGTCCCTGAGCCAGCCCGGGCTGCCCGGCACCAAGCTGTATGGGCCCGAAGGCCTGGAGCTGGTGCGCTTCCCACCGGCCGACGCCATCCCCAGCGAGCGACAGAGGCAGGTGACGCGGAAGCTGTTCGGGCACCTGGAGCGCGGGGTGCTGCTGCACAGCAGCCGGCAGGGTGTGTTCGTCAAGCGGCTGTGCCAGGGCCGCGTGTTCTGCAGCGGCAAC---GCCGTGGTGTGCAAAGGCAGGCCCAACAAGCTGGAGCGTGATGAGGTGGTCCAGGTCTTCGACACCAGCCAGTTCTTTCGAGAGCTGCAGCAGTTCTATAACAGCCAGGGCCGGCTTCCTGACGGCAGGGTGGTGCTGTGCTTTGGGGAAGAGTTTCCGGATATGGCCCCCTTGCGCTCCAAACTCATTCTCGTGCAGATTGAG------CAGCTGTATGTCCGGCAACTGGCAGAAGAGGCTGGGAAGAGCTGTGGAGCCGGCTCTGTG---------ATGCAGGCCCCCGAGGAGCCGCCG---------------CCAGACCAGGTCTTCCGGATG---TTT------------------------------------CCAGATATT------------TGTGCCTCACACCAGAGATCGTTTTTCAGAGAAAACCAACAGATCACCGTCTAA---------------------------------------------

>Human_ENSG00000140968

---------------------------------------------------------------------------------------------------------------------------------------------------------------------------------------------------ATGTGTGACCGGAATGGTGGTCGGCGGCTTCGACAGTGGCTGATCGAGCAGATTGACAGTAGCATGTATCCAGGACTGATTTGGGAGAATGAGGAGAAGAGCATGTTCCGGATCCCTTGGAAACACGCTGGCAAGCAAGATTATAATCAGGAAGTGGATGCCTCCATTTTTAAGGCCTGGGCAGTTTTTAAAGGGAAGTTTAAAGAAGGGGACAAAGCTGAACCAGCCACTTGGAAGACGAGGTTACGCTGTGCTTTGAATAAGAGCCCAGATTTTGAGGAAGTGACGGACCGGTCCCAACTGGACATTTCCGAGCCATACAAAGTTTACCGAATTGTTCCTGAGGAAGAGCAAAAATGCAAACTAGGCGTGGCAACTGCTGGCTGCGTGAATGAAGTTACAGAGATGGAGTGCGGTCGCTCTGAAATCGACGAGCTGATCAAGGAGCCTTCTGTGGACGATTACATGGGGATGATCAAAAGGAGCCCTTCCCCGCCGGAGGCCTGTCGGAGTCAGCTCCTTCCAGACTGGTGGGCGCAGCAGCCCAGCACA---GGCGTGCCGCTGGTGACGGGGTACACCACCTACGACGCGCACCATTCAGCATTCTCCCAGATGGTGATCAGCTTCTACTATGGGGGCAAGCTGGTGGGCCAGGCCACCACCACCTGCCCCGAGGGCTGCCGCCTGTCCCTGAGCCAGCCTGGGCTGCCCGGCACCAAGCTGTATGGGCCCGAGGGCCTGGAGCTGGTGCGCTTCCCGCCGGCCGACGCCATCCCCAGCGAGCGACAGAGGCAGGTGACGCGGAAGCTGTTCGGGCACCTGGAGCGCGGGGTGCTGCTGCACAGCAGCCGGCAGGGCGTGTTCGTCAAGCGGCTGTGCCAGGGCCGCGTGTTCTGCAGCGGCAAC---GCCGTGGTGTGCAAAGGCAGGCCCAACAAGCTGGAGCGTGATGAGGTGGTCCAGGTCTTCGACACCAGCCAGTTCTTCCGAGAGCTGCAGCAGTTCTATAACAGCCAGGGCCGGCTTCCTGACGGCAGGGTGGTGCTGTGCTTTGGGGAAGAGTTTCCGGATATGGCCCCCTTGCGCTCCAAACTCATTCTCGTGCAGATTGAG------CAGCTGTATGTCCGGCAACTGGCAGAAGAGGCTGGGAAGAGCTGTGGAGCCGGCTCTGTG---------ATGCAGGCCCCCGAGGAGCCGCCG---------------CCAGACCAGGTCTTCCGGATG---TTT------------------------------------CCAGATATT------------TGTGCCTCACACCAGAGATCATTTTTCAGAGAAAACCAACAGATCACCGTCTAA---------------------------------------------

# IRF9

>Rat_ENSRNOG00000019478

ATGGCCACAGGCAAAGCACGCTGTACCCGAAAGCTCCGGAACTGGATAGTGGAACAGGTGGAAAGTGGGCAGTTCCCAGGGGTGTGCTGGGACGACGCAGCCAAGACCATGTTCCGGATTCCCTGGAAGCACGCAGGCAAGCAAGACTTCCGAGAAGACCAGGATGCTGCCATATTCAAGGCCTGGGCACTGTTTAAGGAAAAGCACAAAGACGGGGACATAGGAAACCCTGCTGTCTGGAAGACTCGCCTACGCTGTGCCCTCAACAAGAGTTCCGAATTTGAGGAGGTTCCCGAGAGAGGCCGGATGGATGTTGCAGAACCCTACAAAGTATATCGAATACTGCCAGCAGGAACCCTCCCCACCCAACCAAGAAACCAGAAATCGCCATTCAAGCGAAGTATCAGTTCTGTGTCACCTGAGAGGGGAGAG---------TGTAAGGAAAATGGGATTGTAAGCCACTCA---------GACAGCGGCAGCAATGTCTGTGGTGGTGGCAGCAACGGTGGCGGCAGCAGTGACAGC---------------------TGTGGCAGCACCAGC---AGCTCTGAGCTAGAGGAAGGAGCTGGCACAACTGAAGCCACCGTTCAAGAGGACCCAGTGTTCATGGAGAATCCGCTTCCTCCGAACTCAGACTACTCGCTGCTGCTCACCTTCATCTATGGCGGGCGCGTGGTGCATGAGATCCAGGTGCACAGCCTAGACTGTCGGCTTGTGGCTGAGCCCTCAGACCCGGAGAGCAGCATGGTGCAGCAGGTGGTGTTTCCCAGACCTGACCCACTGGAGCCCACCCAGAGCCTGCTGAATCAGCTCGAGAGAGGCATCCTGGTGGCCAGCAATTCCAGAGGCCTCTTTGTTCAGCGCCTCTGCCCCATCCCCATCTCCTGGAACGCACCCGAGGCCCCACCTGGGCCTGGGCCTCATCTGCTGCCCAGCAATAAGTGTGTGGAGCTCTTCAGTACCACATACTTCTGTAGAGATTTGGCCCAGTACTTCCAGGGCCAGGGACCTCTACCCAAGTACCAAGCAACACTGCATTTCTGGGACAGGAGTCCTAGCTCTAGCCATACCCAAGAGAATCTTATCACGGTGCGGATGGAGCAGGCCTTTGCCCGAAATTTACTAGAGAAGATT---CCAGAAGAGGAGAAAGCTGCCTTGTTCCTGGTACAGCACACAGAGCACCAACCTGCCACTCTCGCACTCTGA------------------

>Elephant_ENSLAFG00000026182

ATGGCATCAGGCAAGGCACGCTGCACCCGAAAGCTCCGGAACTGGGTGGTGGAGCAGGTGGAGAGCGGCAAGTTCCCTGGGGTATGCTGGGATGATGAAGCTAAAACCATGTTCCGGATTCCCTGGAAGCATGCAGGCAAGCAAGACTTCCGGGAGGACCAGGATGCTGCCTTCTTCAAGGCCTGGGCGATATTCAAGGGAAAGTACAAGGAGGGAAACTCAGAAGGCCCTGCCACCTGGAAGACTCGCCTGCGCTGTGCCCTCAACAAGAGTTCTGAATTTCAGGAGGTCCCTGAGAGGGGCCGTATGGATGGGGCTGAGCCCTACAAGGTGTATCGGTTGCTGCCACCAGGAACCCTCCTTGCGGAGCCAGGCACCCAAAAATCCCCATCAAAGCGACACCACAGTTCCGAGTCCTCGGACAGGGAGGAGGATGAGAGTCCCGCGAAGAGCCACGTACTCAGCCCCTTCTTGCTTCAGGACCCCTTCAATAATGAGAGG---GGAACCAGTGGGGGAGCAGACCATTCAGACACT------------------------------------------GCTAAGCCTCAGGAAGGCACAGACACAACTGAGGCCCCCTTTGAAGGGGATCAGGCATCTGTGGAATTCGTGCTCCCTCCAGACTTAGACTACTCGCTGCTGCTCACCTTCATCTACAATGGGCGCGTGGTTGGGGAGACCCAGGTGCACACCCTGGACTGCCGCCTTGTGGCTGAGCCCTCGGATTCCCAGAGC---AGCATGAAGCAAGTGGTTTTCCCCAAGGCTGACCCACTGGAGCCCACACAGCGCGTGCTGAGCCAGCTCAAGAGGGGCGTCCTGATGGCCAGCAACCCCAAAGGCCTCTTCGTGCAGCGCCTCTGCCCCATCCCCATCTCCTGGGATGCGCCCCAGACCCCACCTGGTCCAGGCCCACATCTGCTGCCCAGCAATGAGTGCGTGGAGCTCTTCAAAACAGCCTACTTCTACAGAGACTTGGCCAGGTACAACCAGGGCCTGGGCCCCCCACCCAAGTTCCAGGTGACCCTGAATTTCTTGGAGGAAAGTTCTGACCCTAGCCAAAACCCACAGAGTCTTATTACAGTGCAGATGGAGCAAGCCTTTGCCCGACATTTGCTGGAGAGGACAATGCCAGAGGAGCAGGCAGCCACTCTGCCCCTGAGCCTGGAGGATCCCCCCGCCTTCCCATCTCTT---------------------------

>Megabat_ENSPVAG00000007716

ATGGCATCAGGCAAGGCACGCTGTACCCGAAAGCTCCGGAACTGGGTGGTGGAGCAAGTGGAGAGCGGGCAGTTCCCAGGGGTGTGCTGGGAAGATGAAGCTAAGACCATGTTCCGGATTCCCTGGAAGCATGCAGGCAAGCAGGACTTCCGGGAGGACCAGGATGCTGCCTTCTTCAAGGCCTGGGCAATATTTAAGGGAAAGTACAAAGAGGGGGACACAGAAGGCCCCGCTATCTGGAAGACTCGTCTGCGCTGTGCTCTCAACAAGAGTCTCGAATTTGAGGAGGTTCCTGAGAATGGTCACAGGGATGGGGCTGAGCCCTACAAGGTGTATCGGCTGCTTCCTCCAGGTACCGTCCCTGCCCCACCAGACACCCAGAAACTACCACGAAAGAGACATCATAGCTCCGTGTCCCCTGAGAGGGAGGAGAATGCGGGTACTACAAAGAACTGTATACTTAGTCCCTCCTTGCTCCAGGACCCCCTCAAAAATGAGGAGGTGGGGGCCAATGGGGGAACAAGCCATTCAGACTTTGGG------AGCAGCAGCAGCAGCAGCAGCAGCAAC---AGCCCTGAACCTCAGGAAGGTACAGACACAACCGAGTCCCCTTTCCAAGGAGATCAGGTGTCTCCGGAGCTTCTGCCCCCTCCAGACTCAGACTACTCGCTGCTGCTTACCTTCATCTACAACGGGCGCGTGGTGGATGAGGTCCAGGTGCAGAGCCTGGACTGCCGCCTTGTGGCTGAGCCCTCAAGCTCTCAGTACGACGGCATGGAGCAGGTGGTATTTCCCAAGCCTGGCCCGCAAGAGCCCACCCAGCGCCTGCTGAGCCAGCTTGAGAAAGGTGTCCTGGTGGCCAGCAACTCCCGAGGCCTCTTCGTGGAGCGCCTTTGCCCCATTCCCATCTCCTGGAGCGCACCCCAGGCCCCGCCTGGTCCAGGCCCACACCTGCTGCCCAGTAATAAGTGTGTGGAGCTCTTCAGAACCACTTACTTCTGCAGAGACTTGGCCAGGTACTTCCAGGGCCTGGGCCCCCCACCCAAGTTCCAGGTGACACTGAATTTCTGGGAAGAGAGCCCTAGCCCCAGCCACACCCTACAGAGTCTTATCACAGTGCAGATGGAGCAGGCCTTTGCCCGACATTTACTGAAGGAGACT---CCAGAGGACCAGGCAGCCACTCTGTCCCTGGTGCAG------------------------------------------------------

>Camel_ENSCDRG00005016598

ATGGCATCAGGCCGGGCACGCTGCACCCGAAAGCTCCGGAACTGGGTAGTGGAACAAGTGGAAAGCGGGCAGTTCCCAGGAGTGTGCTGGGATGATGAGGCCAAGACCATGTTCCGGATTCCCTGGAAGCACGCAGGCAAGCAGGACTTCCGGGAGGATCAGGATGCTGCCTTCTTCAAGGCCTGGGCGATATTTAAGGGAAAGTACAAGGAGGGGGACACGGAAGGCCCTGCTACCTGGAAGACTCGTCTGCGCTGTGCCCTCAACAAGAGCCCTGAGTTTGAGGAGGTTCCTGAGAATGGCCATAGGGATGGGGCTGAGCCCTACAAGGTGTATCGGCTGGTACCACCAGGAACCCTTGCCGCTCAGCCAGTGACCCAGAAATCACCATCAAAGCGACACCACAGTTCCATGTCCTCTGAGAGGGAGGAGGATGAGGTTACTAGGAAGAACTGTACACTCAGCCCCTCCTTGCTCCAGGACCCCCTCAAAAACGAGTTGGTGGAGACCAATGGGGGAGCAAGCCATTCAGACCTCGGG---AGCAGCAGCGGGAGCGGCAGCAGCAGCAGC---AGCCCTGAGCCTCAGGAAGGCAGGGACATAGCTGAGGCCCCTTTCCAAGGAGATCAGGTGTCCCTGGAGCTTCTGCCCCCTCCAGATTCAGACTACTCGCTGCTGCTCACCTTCATCTACAGTGGGCGCGTGGTGGGTGAGGCCCAGGTGCAGAGCCTGGACTGCCGCCTCGTGGCTGAGCCCTCAGGCTCCCAGTGT---GGCATGGAGCAGGTGATATTTCCCAAACCCGGCCCACAAGAGCCCACCCAGCGCCTGCTGAGCCAGCTTAAGAGAGGGGTCCTGGTGGCCAGCAACTCCCGGGGCCTCTTCGTGCAGCGCCTTTGCCCCATCCCCATCTCCTGGAATGCACCCCAGGCTCCACCTGGTCCAGGCCCGCACCTGCTGCCCAGCAACGAGTGTGTGGAGCTCTTCAGAACCACCTACTTCTGCAGAGACCTGGCCAGATACTTCCAGGGCCTGGGTCCCCCACCTAAGTTCCAGGTGACACTGAATTTTTGGGAGGAGAGCCCCGGCCCCAACCACACCCCACAGAGTCTCATCACAGTGCAGATGGAGCAGGCCTTTGCCCGACATTTACTA---GAGACT---CCTGAGGAGCAGGCAGCCACCCTGTCCCTGCTGCAGAGCCTGGAGGACCCCCTCTCCTCCTCCCCTCTCTGTTCCTCCTACCTCCTCTGA

>Horse_ENSECAG00000024429

ATGGCATCCGGCAGGGCACGCTGCACCAGAAAGCTCCGGAACTGGGTGGTAGAGCAAGTGGAAAGTGGGCAGTTCCCAGGGGTGTGCTGGGATGATGCCGCTAAGACCATGTTCCGGATTCCCTGGAAGCACGCAGGCAAGCAGGACTTCAGGGAGGACCAGGATGCCGCCTTCTTCAAGGCCTGGGCGATATTTAAGGGGAAGTACAAGGAAGGGGACACAGAAGGCCCTGCTATCTGGAAGACCCGTCTGCGCTGTGCTCTCAACAAGAGTCTTGAATTTGAGGAGGTTCCTGAGAATGGCCATCGGGATGGGGCTGAGCCCTACAAGGTGTATCGGCTGCTGCCACCAGGAACCCTTCCTGCCCAGCCAGGGACCCAGAAATCACCATCAAAGCGACACCACAGTTCTGTGTCCTCTGAGAGGGAGGAGGATGGGGGTGCCAGGAAGAACTGCATACTCAGTCCCTCCTTGCCCCAGGACCCCCTCAAGAATGAGGAGGTAGTGGCCAATGGGGATTCGGGCCATTCAGACTTCGGG---------------AGCAGCAGCAGCAGCAAC---AGCCCTGAGCCTCAGGAAGGTACAGACAAAACTGAGGCCCCTGTCCAAGGAGATCAGGGGTCCCTGGAGCTTCTCTCCTCTCCAGACTCAGACTACTCGCTGCTGCTCACCTTCATCTACGATGGGCGCGTGGTGGGTGAGGCCCAGGTGCAGAGCCTGGACTGCCGCCTCGTGGCTGAGCCCTCAGGCTCCCAGTAT---GGCATGGAGCAGGTAGTATTTCCCAAGCCTGGCCCGCAAGAGCCCACCCAGCGCCTGCTGAGCCAGCTCGAGAGAGGCGTCCTGGTGGCCAGTAACTCCCGAGGCCTCTTCGTGCAACGCCTTTGCCCCATCCCCATCTCCTGGAACGCACCCAGGACCCCTCCTGGTCCAGGCCCGCATCTCCTGCCCAGCAATAAGTGCGTGGAGCTCTTCAGAACCACCCACTTCTGTAGAGACTTGGCCAGGTACTTCCAGGGCCTGGGCCCCCCACCCAAGTTCCGAGTAACACTGAATTTCTGGGAGGAGAGCCCTGACCGCAGCCACACCCCAAAGAGTCTGATCACAGTGCAGATGGAGCAGGCCTTTGCCCGACATTTACTGGAGGAGACT---CCAGAGGAGCAGGCAGCCATTCTGTCCTTGGTGCAG------------------------------------------------------

>Cat_ENSFCAG00000018849

ATGGCATCGGGCCGGGCACGCTGCACCCGAAAGCTGCGGAACTGGGTGGTGGAGCAAGTGGAGAGCGGGCAGTTCCCGGGGGTGTGCTGGGACGATGCAGCTAAGACCATGTTCCGGATTCCTTGGAAGCATGCAGGCAAGCAGGACTTCCGGGAGGACCAGGATGCCGCTTTCTTCAAGGCATGGGCGATATTTAAGGGGAAGTACAAGGAGGGAGACACAGAAGGCCCTGCTATCTGGAAGACTCGTCTGCGCTGTGCTCTCAACAAGAGCCCCGAATTTGAGGAGGTTCCTGAAAATGGCCATAGGGATGGAGCTGAGCCCTACAAGGTGTATCGGCTGCTGCCATCGGGGACCCTCCCTGCCCAGCCAGGGACCCAGAAATCACCGTCAAAGCGACATTACAGTTCTGTGTCCTCTGAGAGGGAAGAGGATGCGGGTACCACAAAGAACTGCATACTCAGTCCCTCTTTGATTGAGGACCCCCTCAAAAACGAGGAGGTGGGGGCCAGTGGGGAAGCAGGCCATTCAGAATTTGGG---------------AGCAGCAGCAGCAGCAAC---AGCCCTGAGCCTCAGGAAGGTACAGACACAACAGAAGCCCCTTTCCAAGGAGATCAGGTGTCATTGGAGAGTCTGCCTCCTCCAGACTCAGACTACTCGCTGCTGCTTACCTTCATCTACGGTGGGCGCGTGGTGGGCGAAGCCCAGGTGCAGAGCCTGGACTGCCGCCTTGTGGCTGAGCCCTCAGGCTCCCAGTAT---GGGATGGAGCAGGTGGTGTTTCCCAAGCCTGACCCACGAGAGCCCACCCAGCGCCTGCTGAGCCAGATCGAGAGAGGTGTCCTAGTGGCCAGCAACTCCAGAGGCCTCTTTGTGCAGCGTCTCTGCCCCATCCCCATCTCCTGGAACGCGCCCCAGGCGCCACCCGGTCCAGGCCCGCATCTGCTGCCCAGCAATGAGTGCGTGGAGCTCTTCAGAACCACCTACTTCTGCAGAGACCTGGCCAGGTACTTCCAGGGCCTGGGCCCCCCACCCAAGTTCCAGGTGACTCTGAACTTCTGGGAGGAGAGCCCTGGCCCCAGCCACACCCCGAAGAGTCTTATCACAGTGCAGATGGAGCAGGCCTTTGCCCGACGTTTACTGGAGGAAACT---CCTGAGGAGCAGGCAGCCGCTCTCTCCCTGCTGCAGAGCCTGGGAGACCCGCCTTCCTCCTCTTCGCTCTGTTCCTCCTATCTCCTTTGA

>Lion_ENSPLOG00000017710

ATGGCATCGGGCCGGGCACGCTGCACCCGAAAGCTGCGGAACTGGGTGGTGGAGCAAGTGGAGAGTGGGCAGTTCCCGGGGGTGTGCTGGGACGATGCAGCTAAGACCATGTTCCGGATTCCTTGGAAGCATGCAGGCAAGCAGGACTTCCGGGAGGACCAGGATGCCGCTTTCTTCAAGGCATGGGCGATATTTAAGGGGAAGTACAAGGAGGGAGACACAGAAGGCCCTGCTATCTGGAAGACTCGTCTGCGCTGTGCTCTCAACAAGAGCCCCGAATTTGAGGAGGTTCCTGAAAATGGCCATAGGGATGGAGCTGAGCCCTACAAGGTGTATCGGCTGCTGCCATCGGGGACCCTCCCTGCCCAGCCAGGGACCCAGAAATCACCATCAAAGCGACATTACAGTTCTGTGTCCTCTGAGAGGGAAGAGGATGAGGGTACCACAAAGAACTGCATACTGAGTCCCTCTTTGATTGAGGACCCCCTCAAAAACGAGGAGGTGGGGGCCAGTGGGGAAGCAGGCCATTCAGAATTTGGG---------------AGCAGCAGCAGCAGCAAC---AGCCCTGAGCCTCAGGAAGGTACAGACACAACAGAAGCCCCTTTCCAAGGAGATCAGGTGTCATTGGAGAGTCTGCCCCCTCCAGACTCAGACTACTCGCTGCTGCTTACCTTCATCTACGGTGGGCGCGTGGTGGGCGAAGCCCAGGTGCAGAGCCTGGACTGCCGCCTTGTGGCTGAGCCCTCAGGCTCCCAGTAT---GGGATGGAGCAGGTGGTGTTTCCCAAGCCTGACCCACGAGAGCCCACCCAGCGCCTGCTGAGCCAGATCGAGAGAGGTGTCCTAGTAGCCAGCAACTCCAGAGGCCTCTTCGTGCAGCGTCTCTGCCCCATCCCCATCTCCTGGAACGCGCCCCAGGCGCCACCCGGTCCAGGCCCACATCTGCTGCCCAGCAATGAGTGCGTGGAGCTCTTCAGAACCACCTACTTCTGCAGAGACCTGGCCAGGTACTTCCAGGGCCTGGGCCCCCCACCCAAGTTCCAGGTGACTCTGAACTTCTGGGAGGAGAGCCCTGGCCCCAGCCACACCCCGAAGAGTCTTATCACAGTGCAGATGGAGCAGGCCTTTGCCCGACGTTTACTGGAGGAGACT---CCTGAGGAGCAGGCAGCCGCTCTCTCCCTGCTGCAG------------------------------------------------------

>GreaterBambooLemur_ENSPSMG00000015728

ATGGCATCAGGCAGGGTGCGCTGCACCCGAAAACTCCGCAACTGGGTGGTGGAACAAGTGGAGAGCGGGCAGTTCCCAGGGGTGTGCTGGCATGATACAGCTAAGACCATGTTCCGGATTCCCTGGAAGCATGCAGGCAAGCAGGACTTCCGTGAGGACCAAGATGCTGCCTTCTTCAAGGCCTGGGCAATATTTAAGGGAAAGTACAAGGAAGGGGACACAGGAGGCCCTGCTACCTGGAAGACTCGCCTGCGCTGTGCCCTCAATAAGAGTCCCGAATTTGAAGAGGTTCCCGAGAACGGCCGTATGGATGTTGCTGAGCCCTACAAGGTGTATCGGCTACTACCGCCAGGAACCCTCTCTTCCCAACCAGGAACCCAGAAATCACCATCAAAGCGACACTATAGTTCTGTGTCCTCTGAGAAGGAGGAGGAAGAGGGTCCCATGAAGAACTGCATACCCAGTCCCTCCTTGCTTCAGGACCCCCTTGATAATGAGGAGGTGGGGGCCGCTGGGGGAGCATCCTGTTCAGACAGCAACAGCAGCAGCAGCAGCAGCAGCAGCAGCAGCAGCCACAGTCCTGAGCCACAGGAAGGTGCAGATACAACTGAGGCCCCCTTTCAAGGGGGTCAGGTGTCTCCAGAGTTTCTGCCCCCTCCGGAGTCAGACTACTCGCTGCTGCTCACCTTCATCTACAGCGGGCGCGTGGTGGGCAAGGCCCAGGTGCAGAGCCTGGACTGCCGCCTTGTGGCTGAGCCCTCAGGCTCCGAGAGC---AGCATGGAGCAAGTGGTGTTTCCCAAGCCTGACCCACTGGAGCCCACACAGCGCCTGCTGAGCCAGCTTGAGAGAGGTGTCCTGGTGGCCAGCAACTCCCGAGGCCTCTTTGCGCAGCGCCTTTGCCCCATCCCCATCTCCTGGAATGCACCACAGGCCCCACCTGGGCCAGGCCCACATCTGCTGCCCAGCAACGAGTGCGTGGAGCTCTTCAGAACTGCCTCCTTCTGCAGAGACTTGGCCAATTACCTCCGGGGCCTGGGCCCCCAACCCAAGTTCCAGGTAACACTGAATTTCTGGGAGGAGAGCCCTGGCCCCAGCTGTACGTCACAGAACCTTATCACAGTGCGGATGGAGCAGGCCTTTGCCCGACACTTACTGGAGGAGACT---TCAGAGGAACAGGCAGCCATTCTGTCCCTGATGTAG------------------------------------------------------

>CoquerelsSifaka_ENSPCOG00000015057

ATGGCATCAGGCAGGGTGCGCTGTACCCGAAAACTCCGCAACTGGGTGGTGGAACAAGTGGAGAGCGGGCAGTTCCCAGGGGTGTGCTGGCACGATACAGCTAAGACCATGTTCCGGATTCCCTGGAAGCATGCAGGCAAGCAGGACTTCCGTGAGGACCAAGATGCTGCCTTCTTCAAGGCCTGGGCAATATTTAAGGGAAAGTACAAGGAAGGGGACACAGGAGGCCCTGCTGCCTGGAAGACTCGCCTGCGCTGTGCCCTCAACAAGAGTCCCGAATTTGAAGAGGTTCCTGAGAATGGCCGTATGGATGTTGCTGAGCCCTATAAGGTGTATCGGCTACTACCGCCAGGAACCCTCTCTTCCCAACCAGGAACCCAGAAATCACCATCAAAGCGACGCTACAGTTCTGTGTCCTCTGAGAAGGAGGAGGAAGAGGGTGCCATGAAGAACTGCGTACACAGTCCCTCCTTGTTTCAGGACCCCCTTGATAATGAAGAGGTAGGAGCCACTGGGGGATCATCCCATTCAGACAGCAGC---------------AGCAGCAGCAGCAGCGGC---AGCCCTGAGCCACAGGAAGGTGCAGACACAACTGGGGCCCCCTTTCAAGGGGATCAGGTGTCTCCGGAGTTTCTGCTCCCTCCGGAGTCAGACTACTCGCTGCTGCTCACCTTCATCTACAGCGGGCGCGTGGTGGGTAAGGCCCAGGTGCAGAGCCTGGACTGCCGCCTTGTGGCTGAGCCCTCAGGTTCTGAGAGC---AGCATGGAGCAAGTGGTGTTTCCCAAGCCTGACCCACTGGAGCCCACACAGCGCCTGCTGAGCCAGCTTGAGAGAGGTGTCCTGGTGGCAAGCAACTCCCGAGGCCTCTTTGTGCAGCGCCTTTGCCCCATCCCGATCTCATGGAATGCACCGCAGGCCCCCCCTGGGCCAGGCCCACATCTGCTGCCCAGCAACGAGTGTGTGGAGCTCTTCAGAACCGCGTCCTTCTGCAGAGACTTGGCCAATTACCTCCAGGGCCTGGGCCCCCAACCCAAGTTCCAGGTAACACTGAATTTCTGGGAGGAGAGCCCTGGCCCCAGCTATATGTCACAGAATCTTATCACAGTGCGGATGGAGCAGGCCTTTGCCCGACACTTACTGGAGGAGACT---TCAGAGGAACAGGCAGCCATTCTGTCCCTGATGTAG------------------------------------------------------

>MouseLemur_ENSMICG00000027895

ATGGCATCAGGCAGGGTGCGTTGTACCCGAAAACTCCGCAACTGGGTGGTGGAACAAGTGGAGAGTGGGCAGTTCCCAGGGGTGTGCTGGCATGATACCACTAAGACCATGTTCCGAATTCCCTGGAAGCATGCAGGCAAGCAGGACTTCCGTGAGGACCAAGACGCTGCCTTCTTCAAGGCCTGGGCAATATTTAAGGGAAAGTACAAGGAAGGGGACCCAGGAGGCCCTGCTACCTGGAAGACTCGCCTGCGCTGTGCCCTCAACAAGAGTCCTGAATTTGAAGAAGTTCCCGAGAACGGCCGTATGGATGTTGCAGAGCCCTACAAGGTGTATCGGCTACTACCGCCAGGAACCCTCTCTTCCCAACCAGGAATCCAGAAATCACCATCAAAGCGACACTACAGTTCTGTGTCTTCTGAGAAGGAGGAGGAAGAGGGTGCCATGAAGAACTGCATACACAGTCCCTCCTTGCTTCAGGGCCCCCTTGATAATGAGGAGGCAGGGGCCGCTGGGGGAGCATCCCATTCAGACAGCAGC---------------AGTAGCAGCGGCAGCAGC---GGCCCTGAACCACAGGAAGGTGCAGACACAACTGAGGACCCCTTTCAAGGGGATCATGTGTCCCCGGAGTTTCTGCCCCCTCCAGAGTCAGACTACTCGCTGCTGCTCACCTTCATCTACAGCGGGCGCGTGGTGGGCAAGGCCCAGGTGCAGAGCCTGGACTGCCGCCTTGTGGCTGAGCCCTCAGGCTCTGAGAGC---AGCATGGAGCAAGTGGTGTTTCCCAAGCCTGACCCACTGGAGCCCACACAGCGCCTGCTGAGCCAGCTTGAGAGAGGTGTCCTGGTGGCCAGCAACTCCCGAGGCCTCTTCGTGCAGCGCCTTTGCCCCATCCCCATCTCCTGGAATGCACCACAGGCCCCGCCTGGGCCAGGCCCACATCTGCTGCCCAGCAATGAGTGCGTGGAGCTCTTCAGAACCACCTCTTTCTGCAGAGACTTGGCCAATTACCTCCAGGGCCTGGGCCCCCAACCCAAGTTCCAGGTAACACTGAATTTCTGGGAGGAGAGCCCTGGCCCCAGCTATATGTCACAGAATCTTATCACAGTGCGGATGGAGCAGGCCTTTGCCCGACACTTACTG---GAGACT---TCAGAGGAACGGGCAGCCGTTCTGTCCCTGATGTAG------------------------------------------------------

>Tarsier_ENSTSYG00000007445

ATGGCATCAGGCAGGGCACGCAGCACCCGAAAACTCCGGAACTGGGTAGTGGAGCAAGTAGAGAGCGGGCAGTTCCCAGGGGTGTGCTGGGATGATGCAGCTAGGACCATGTTCCGGATTCCCTGGAAGCATGCAGGCAAGCAGGACTTCCGGGAGGACCAGGATGCTGCCTTCTTCAAGGCCTGGGCAGTATTTAAGGGAAAGTTTAAGGAGGGAGACACAGGAGGCCCTGCTGCCTGGAAGACTCGCCTGCGCTGTGCCCTCAACAAGAGTCCTGAATTTGAGGAGGTACCCAAGAGAGGCCGTATGGACGTTGCCGAGCCCTACAAGGTGTATCGGCTGCTGCCACCAGGAAATCTCTCTGCCCAGCCAGGGACTCAGAAATCACCATCAAAGCGACACCACAGTCCTGTGTCCTCTGAGAGGGAGGAGGAAGAGGATGCTGTGAAGAACTGCACACTCAGTCCCTCTGTGCTCCAGGACCCCCTTGATGATGAAGAGGTGGGGGCCGATGGGGGAGCAGTCCATTCAGACATTGGGAGCAGCAGCAGCAGCAGCAGCAGCAGTAGCAGC---AGCCCTGAGCCACAGAAAGGTGCAGACACAACTGAGTCCCCCTTTCCAGGGGATCAGGTGTCCCTGAAGCTTCTGTTCCCTTCAGAGCCAGACTACTCGCTGCTGCTCACCTTCATCTACAGTGGGCGTGTGGTGGGCAAGACCCAGGTGCACAGCCTGGACTGCCGCCTCGTGGCTGAACCCTCAGGCTTTAAGAGC---AACATGGAGCAGGTGGTGTTTCCCAAGCCTAGCCCACTGGAGCCCACACAGCGCCTGCTGAGCCAGCTCGAGAAGGGCATCCTGGTGGCCAGCAACCCCCGAGGCCTCTTCGTTCAGCGCCTTTGCCCCATCCCCATCTCCTGGAACGCACCCCATGCCCCACCTGGGCCGGGCCCACATCTGCTGCCCAGCAACGAGTGTGTGGAGCTCTTCAGTCCTGACCACTTCTGCAGAGACTTGGCCAGGTACATCCAGGGCCTGGGCCCCCTACCCAAGTTCCAAGTAACACTGAATTTCTGGGAGGAGAGCTGTGGCCCCAGCCATACTCCACAGAATCTTATCACAGTGCAGATGGAACAGGCCTTTGCCCGACACTTATTGGAGGAGACT---CCAGAGCAGGAGGCAGCCATTCTGTCCCTGGTGTAG------------------------------------------------------

>Chimpanzee_ENSPTRG00000034494

ATGGCATCAGGCAGGGCACGCTGCACCCGAAAACTCCGGAACTGGGTGGTGGAGCAAGTGGAGAGTGGGCAGTTTCCCGGAGTGTGCTGGGATGATACAGCTAAGACCATGTTCCGGATTCCCTGGAAACATGCAGGCAAGCAGGACTTCCGGGAGGACCAGGATGCTGCCTTCTTCAAGGCCTGGGCAATATTTAAGGGAAAGTATAAGGAGGGGGACACAGGAGGTCCGGCTGTCTGGAAGACTCGCCTGCGCTGTGCACTCAACAAGAGTTCTGAATTTAAGGAGGTTCCTGAGAGGGGCCGCATGGATGTTGCTGAGCCCTACAAGGTGTATCAGTTGCTGCCACCAGGAACTGTCTCTGGCCAGCCAGGGACTCAGAAATCACCATCAAAGCGACATCACAGTTCTGTGTCCTCTGAGAGGGAGGAGGAAGAGGATGCCATGCAGAACTGCACACTCAGTCCCTCTGTGCTCCAGGACTCCCTCAATAATGAGGAGGAGGGGGCCAGTGGGGGAGCAGTCCATTCAGACATCGGG------------------AGCAGCAGCAGCAGC---AGCCCTGAGCCACAGGAAGTTACAGACACAACTGAGGCCCCCTTTCAAGGGGATCAGAGGTCCCTGGAGTTTCTGCTTCCTCCAGAGCCAGACTACTCACTGCTGCTCACCTTCATCTACAACGGGCGCGTGGTGGGCGAGGCCCAGGTGCAAAGCCTGGATTGCCGCCTTGTGGCTGAGCCCTCAGGCTCTGAGAGC---GGCATGGAGCAGGTGCTGTTCCCCAAGCCTGGCCCACTGGAGCCCACGCAGCGCCTGCTGAGCCAGCTTGAGAGGGGCATCCTAGTGGCCAGCAACCCCCGAGGCCTCTTCGTGCAGCGCCTTTGCCCCATCCCCATCTCCTGGAATGCACCCCAGGCTCCACCTGGGCCAGGCCCGCATCTGCTGCCCAGCAACGAGTGCGTGGAGCTCTTCAGAACCGCCTACTTCTGCAGAGACTTGGCCAGGTACTTTCAGGGCCTGGGCCCCCCACCGAAGTTCCAGGTAACACTGAATTTCTGGGAAGAGAGCCATGGCTCCAGCCATACTCCACAGAATCTTATCACAGTGAAGCACGAG---CCTTTGCCCAGATACTTGCTGCAGCAGAAG---CAGACTGCCAGAAGCAAGCAAAGCAGCCTATTC---------------------------------------------------------

>MasSightMonkey_ENSANAG00000037203

ATGGCATCAGGCAGGGCACGCTGTACCCGAAAACTGCGGAACTGGGTGGTGGAGCAAGTGGAGAGCGGGCAGTTCCCTGGAGTGTGCTGGGATGATGCAGCTAAGACCATGTTCCGGATTCCCTGGAAGCATGCAGGCAAGCAGGACTTCCGGGAGGACCAGGATGCTGCCTTCTTCAAGGCCTGGGCAATATTTAAGGGAAAGTATAAGGAGGGGGACACAGGAGGCCCTGCTGCCTGGAAGACTCGCTTACGCTGTGCACTCAACAAGAGTCCTGAATTTGAGGAAGTTCCTGAGAGAGGCCACATGGATGTTGCTGAGCCCTACAAGGTGTATCGCCTGCTGCCACCAGGAACCCTGTCTGGA------GGGACTCAGAAATCACCATCAAAGCGACAGCACAATTCTGTGTCTTCTGAGAGGAAGGAGGAAGAGGGTGCCATGCAGAACTGTACACTCAGTCCCTCTGTGCTCCAGGACTCCCTCAATAATGAAGAGGAGGGAGCCAGTGGGGGAGCAGTCCATTCAAACACTGGG------AGCAGCAGCAGCAGCAGCAGCAGCAGC---AGCCCTGAGCCACAGGAAGGTACAGACACAACTGAAGCTCCCTTACAAGGGGATCAGGTGTCCCTGGAGTTTCTGCTTCCTCCAGAGCCAGACTACTCACTACTGCTCACCTTCATCTACAACGGGCGCGTGGTGGGCAAGGCCCAGGTGCAAAGCCTGGACTGCCGCCTTGTGGCTGAGCCCTCAGGCTCTGAGAGC---AGCATGGAGCAGGTGCTATTCCCCAAGCCTGGTCCACTGGAGCCCATGCAGCGCCTGCTGAGTCAGCTTGAGAGGGGCATCCTGGTGGCCAGCAACCCCCGAGGCCTCTTCGTGCAGCGCCTTTGCCCCATCCCCATCTCCTGGAATGCACCCCAGGCTCCACCTGGGCCAGGTCCGCATCTGCTGCCCAGCAACGAGTGCGTGGAGCTCTTCAGAACTGCCTACTTCTGCAGAGACTTGGCCAGGTACTTCCAGGGCCTAGGCCCCCCACCCAAGTTCCAGGTAACACTGAATTTCTGGGAGGAGAACTGTGGCCCCAGCCATACTCCACAGAATCTTATCACAGTGAAGATGGAGCAGGCTTTTGCCCGACACTTACTAGAGGAGACT---CCAGAGCAGCAGGCAGCCATTCTGTCCCTGGTGTAG------------------------------------------------------

>Marmoset_ENSCJAG00000007065

ATGGCATCAGGCAGGGCACGCTGTACCCGAAAACTCCGGAACTGGGTGGTGGAGCAAGTGGAGAGCGGGCAGTTCCCCGGAGTGTGCTGGGATGATGCAGCTAAGACCATGTTCCGGATTCCCTGGAAGCATGCAGGCAAGCAGGACTTCCGGGAGGACCAGGATGCTGCCTTCTTCAAGGCCTGGGCAATATTTAAGGGAAAGTATAAGGAGGGGGACACAGGAGGCCCCGCTGCCTGGAAGACTCGCCTACGCTGTGCACTCAACAAGAGTCCTGAATTTGAGGAAGTTCCTGAGAGGGGCCGCATGGATGTTGCTGAGCCCTACAAGGTGTATCGCCTGCTGCCACCAGGAACCCTCTCTGGA------GGGACTCAGAAATCACCATCAAAGCAACAGCACAGTTCTGTGTCTTCTGAGAGGAAGGAGGAAGAGGGTGCCATGCAGAACTGTACACTCAGTCCCTCTGTGCTCCAGGACTCCCTCAATAATGAAGAGGAGGGAGCCAGTGGGGGAGCAGTCCATTCAGACATCGGA---------------AGCAGCAGCAGCAGCAGC---AGCCCTGAACCACAGGAAGGTACAGATACAACTGAGGCCCCCTTACAAGGGGATCAGGTGTCCCTGGAGTTTCTACTTCCTCCAGAGCCAGACTACTCACTGCTGCTCACCTTCATCTACAACGGGCGCGTGGTGGGCGAGGCCCAGGTGCAAAGCCTGGACTGCCGCCTCGTGGCTGAGCCCTCAGGCTCTGAGAGC---AGCATGGAGCAGGTGTTATTCCCCAAGCCTGGCCCACTGGAGCCCACGCAGCGCCTACTGAGTCAGCTTGAGAGGGGCATCCTGGTGGCCAGCAACCCCCGAGGCCTCTTCGTGCAGCGCCTTTGCCCCATCCCCATCTCCTGGAATGCACCCCAGGCTCCACCTGGGCCAGGCCCACATCTGCTGCCCAGCAACGAGTGTGTGGAGCTCTTCAGAACTGCCTACTTCTGCAGAGACTTGGCCAGGTACTTCCAGGGCCTGGGCCCCCCACCCAAGTTCCAGGTAACACTGAATTTCTGGGAGGAGAGCTGTGGCCCCAGCCATACTCCACAGAATCTTATCACAGTGAAGATGGAGCAGGCTTTTGCCCGACACTTACTGGAGGAGACT---CCAGAGCAGCAGGCAGCCATTCTGTCCCTGGTGTAG------------------------------------------------------

>BolivianSquirrelMonkey_ENSSBOG00000020055

ATGGCATCAGGCAGGGCACGCTGTACCCGAAAACTCCGGAACTGGGTGGTGGAGCAAGTGGAGAGCGGGCAGTTCCCCGGAGTGTGCTGGGATGATGCAGCTAAGACCATGTTCCGGATTCCCTGGAAGCATGCAGGCAAGCAGGACTTCCGGGAGGATCAGGATGCTGCCTTCTTCAAGGCCTGGGCAATATTTAAGGGAAAGTATAAGGAGGGGGACACAGGAGGCCCCGCTGCCTGGAAGACTCGCCTACGCTGTGCACTCAACAAGAGTCCTGAATTTGAGGAAGTCCCTGAGAGGGGCCGCATGGATGTTGCTGAGCCCTACAAGGTGTATCGCCTGCTGCCACCAGGAACCCTCTCTGGA------GGGACTCAGAAATCACCATCAAAGCGACAGCACAGTTCTGTGTCTTCTGAGAGGAAAGAGGAAGAGGGTGCCATGCAGAACTGTACACTCAGTCCCTCTGTGCTCCAGGACTCCCTCAATAATGAAGAGGAGGGAGCCAGTAGGGGAGCAGTCCATTCAGACATC------AGGAGCAACAGCAGCAGCAGCAGCAGCAGC---AGCCCTGAGCCACAGGAAGGTACAGACACAACTGATGCCCCCTTACAAGGGGATCAGGTGTTCCTGGAGTTTCTGCTTCCTCCAGAGTCCGACTACTCACTGCTGCTCACCTTCATCTACAATGGGCGCGTGGTGGGCGAGGCCCAGGTGCAAAGCCTGGACTGCCGCCTTGTGGCTGAGCCCTCAGGCTCTGAGAGC---AGCATGGAGCAGGTGCTATTCCCCAAGCCTGGTCCACTGGAGCCTACACAGCGCCTGCTGAGTCAGCTTGAGAGGGGCATCCTGGTGGCCAGCAACCCCCGAGGCCTCTTCGTGCAGCGCCTTTGCCCCATCCCCATCTCCTGGAATGCACCCCAGGATCCACCTGGGCCAGGCCCACATCTGCTGCCCAGCAACGAGTGCGTGGAGCTCTTCAGAACTGCCTACTTCTGCAGAGACTTGGCCAGGTACTTCCAGGGCCTGGGCCCCCCACCCAAGTTCCAGGTAACACTGAATTTCTGGGAGGAGAGCTGTGGCCCCAGCCATACTCCACAGAATCTTATCACGGTGAAGATGGAGCAGGCTTTTGCCCGACACTTACTGGAGGAGACT---CCAGAGCAGGAGGCAGCCATTCTGTCCCTGGTGTAG------------------------------------------------------

>Orangutan_ENSPPYG00000005686

ATGGCATCAGGCAGGGCACGCTGCACCCGAAAACTCCGGAACTGGGTGGTGGAGCAAGTGGAGAGTGGGCAGTTCCCCGGAGTGTGCTGGGATGATACAGCTAAGACCATGTTCCGGATTCCCTGGAAGCATGCAGGCAAGCAGGACTTCCGGGAGGACCAGGATGCTGCCTTCTTCAAGGCCTGGGCAATATTTAAGGGAAAGTATAAGGAGGGGGACACAGGAGGTCCGGCTGTCTGGAAGACTCGCCTGCGCTGTGCACTCAACAAGAGTTCTGAATTTAAGGAGGTTCCTGAGAGGGGCCGCATGGATGTTGCTGAGCCCTACAAGGTGTATCGGTTGCTGCCACCAGAAACCGTCTCTGGCCATCCAGGGACTCAGAAACCACCATCAAAGCGACAGCACAGTTCTGTGTCCTCTGAGAGGAAGGAGGAAGAGGGTGCCATGCAGAACTGCACACTCAGTCCCTCTGTGCTCCAGGACTCCCTCAATAATGAGGAGGAGGGGGCCAGTGGGGGAGCAGTCCATTCAGACATCGGG------------------AGCAGCAGCAGCAGC---AGCCCTGAGCCACAGGAAGTTACAGACACAACTGAGGCCCCCTTTCAAGGGGATCAGAGGTCCCTGGAGTTTCTGCTTCCTCCAGAGCCAGACTACTCACTGCTGCTCACCTTCATCTACAACGGGCGTGTGGTGGGCGAGGCCCAGGTGCAAAGCCTGGACTGCCGCCTTGTGGCTGAGCCCTCAGGCTCTGAGAGC---AGCATGGAGCAGGTGCTTTTCCCCAAGCCTGGCCCACTGGAGCCCACGCAGCGCCTGCTGAGCCAGCTTGAGAGGGGCATCCTGGTGGCCAGCAACCCCCGAGGCCTCTTCGTGCAGCGCCTTTGCCCCATCCCCATCTCCTGGAATGCACCCCAGGCTCCACCTGGGCCAGGCCCGCATCTGCTGCCCAGCAACGAGTGCGTGGAGCTCTTCAGAACCGCCTACTTCCGCAGAGACTTGGCCAGGTACTTTCAGGGCCTGGGCCCCCCACCAAAGTTCCAGGTAACACTGAATTTCTGGGAAGAGAGCCATGGCTCCAGCCATACTCCACAGAATCTTATCACAGTGAAGATGGAGCAGGCCTTTGCCCGATACTTGCTGGAGCAGACT---CCAGAGCAGCAGGCAGCCATTCTGTCCCTGGTGTAG------------------------------------------------------

>Gibbon_ENSNLEG00000015294

ATGGCATCAGGCAGGGCACGCTGCACCCGAAAACTCCGGAACTGGGTGGTGGAGCAAGTGGAGAGTGGGCAGTTTCCCGGAGTGTGCTGGGATGATACAGCTAAGACCATGTTCCGGATTCCCTGGAAGCATGCAGGCAAGCAGGACTTCCGGGAGGACCAGGATGCTGCCTTCTTCAAGGCTTGGGCAATATTTAAGGGAAAGTATAAGGAGGGGGACACAGGAGGTCCGGCTGTCTGGAAGACTCGCCTGCGCTGTGCACTCAACAAGAGTTCTGAATTTAAGGAGGTTCCTGAGAGGGGCCGCATGGATGTTGCTGAGCCCTACAAGGTGTATCAGTTGCTGCCACCAGGAACCGTCTCTGGCCAGCCAGGGACTCAGAAATCACCATCAAAGCAACAGCACAGTTCTGTGTCCTCTGAGAGGAAGGAGGAAGAGGGTGCCATGCAGAACTGCACACTCAGTCCCTCTGTGCTCCAGGACTCCCTCAATAATGAGGAGGAGGGGGCCAGTGGGGGAGCAGTCCATTCAGACATCGGG------------------AGCAGCAGCAGCAGC---AGCCCTGAGCCACAGGAAGTTACAGATACAACTGAGGCCCCCTTTCAAGGGGATCAGAGGTCCCTGGAGTTTCTGCTTCCTCCAGAGCCAGACTACTCACTGCTGCTCACCTTCATCTACAACGGGCGCGTGGTGGGCGAGGCCCAGGTGCAAAGCCTGGATTGCCGCCTTGTGGCTGAGCCCTCAGGCTCTGAGAGC---AGCATGGAGCAGGTGCTGTTCCCCAAGCCTGGCCCACTGGAGCCCACGCAGCGCCTGCTGAGCCAGCTTGAGAGGGGCATCCTGGTGGCCAGCAACCCCCGAGGCCTCTTCGTGCAGCGCCTTTGCCCCATCCCCATCTCCTGGAATGCACCCCAGGCTCCACCTGGGCCAGGCCCCCATCTGCTGCCCAGCAACGAGTGCGTGGAGCTCTTCAGAACCGCCTACTTCTGCAGAGACTTGGCCAGGTACTTTCAGGGCCTGGGCCCCCCACCGAAGTTCCAGGTAACACTGAATTTCTGGGAAGAGAGCCATGGCTCCAGCCATACTCCACAGAATCTTATCACAGTGAAGATGGAGCAGGCCTTTGCCCGATACTTGCTGGAGGAGACT---CCAGAGCAGCAGGCAGCCATTCTGTCCCTGGTGTAG------------------------------------------------------

>Bonobo_ENSPPAG00000012521

ATGGCATCAGGCAGGGCACGCTGCACCCGAAAACTCCGGAACTGGGTGGTGGAGCAAGTGGAGAGTGGGCAGTTTCCCGGAGTGTGCTGGGATGATACAGCTAAGACCATGTTCCGGATTCCCTGGAAACATGCAGGCAAGCAGGACTTCCGGGAGGACCAGGATGCTGCCTTCTTCAAGGCCTGGGCAATATTTAAGGGAAAGTATAAGGAGGGGGACACAGGAGGTCCGGCTGTCTGGAAGACTCGCCTGCGCTGTGCACTCAACAAGAGTTCTGAATTTAAGGAGGTTCCTGAGAGGGGCCGCATGGATGTTGCTGAGCCCTACAAGGTGTATCAGTTGCTGCCACCAGGAACCGTCTCTGGCCAGCCAGGGACTCAGAAATCACCATCAAAGCGACATCACAGTTCTGTGTCCTCTGAGAGGGAGGAGGAAGAGGATGCCATGCAGAACTGCACACTCAGTCCCTCTGTGCTCCAGGACTCCCTCAATAATGAGGAGGAGGGGGCCAGTGGGGGAGCAGTCCATTCAGACATCGGG------------------AGCAGCAGCAGCAGC---AGCCCTGAGCCACAGGAAGTTACAGACACAACTGAGGCCCCCTTTCAAGGGGATCAGAGGTCCCTGGAGTTTCTGCTTCCTCCAGAGCCAGACTACTCACTGCTGCTCACCTTCATCTACAACGGGCGCGTGGTGGGCGAGGCCCAGGTGCAAAGCCTGGATTGCCGCCTTGTGGCTGAGCCCTCAGGCTCTGAGAGC---GGCATGGAGCAGGTGCTGTTCCCCAAGCCTGGCCCACTGGAGCCCACGCAGCGCCTGCTGAGCCAGCTTGAGAGGGGCATCCTAGTGGCCAGCAACCCCCGAGGCCTCTTCGTGCAGCGCCTTTGCCCCATCCCCATCTCCTGGAATGCACCCCAGGCTCCACCTGGGCCAGGCCCGCATCTGCTGCCCAGCAACGAGTGCGTGGAGCTCTTCAGAACCGCCTACTTCTGCAGAGACTTGGCCAGGTACTTTCAGGGCCTGGGCCCCCCACCGAAGTTCCAGGTAACACTGAATTTCTGGGAAGAGAGCCATGGCTCCAGCCATACTCCACAGAATCTTATCACAGTGAAGATGGAGCAGGCCTTTGCCCGATACTTGCTGGAGCAGACT---CCAGAGCAGCAGGCAGCCATTCTGTCCCTGGTGTAG------------------------------------------------------

>Human_IRF9

ATGGCATCAGGCAGGGCACGCTGCACCCGAAAACTCCGGAACTGGGTGGTGGAGCAAGTGGAGAGTGGGCAGTTTCCCGGAGTGTGCTGGGATGATACAGCTAAGACCATGTTCCGGATTCCCTGGAAACATGCAGGCAAGCAGGACTTCCGGGAGGACCAGGATGCTGCCTTCTTCAAGGCCTGGGCAATATTTAAGGGAAAGTATAAGGAGGGGGACACAGGAGGTCCAGCTGTCTGGAAGACTCGCCTGCGCTGTGCACTCAACAAGAGTTCTGAATTTAAGGAGGTTCCTGAGAGGGGCCGCATGGATGTTGCTGAGCCCTACAAGGTGTATCAGTTGCTGCCACCAGGAATCGTCTCTGGCCAGCCAGGGACTCAGAAAGTACCATCAAAGCGACAGCACAGTTCTGTGTCCTCTGAGAGGAAGGAGGAAGAGGATGCCATGCAGAACTGCACACTCAGTCCCTCTGTGCTCCAGGACTCCCTCAATAATGAGGAGGAGGGGGCCAGTGGGGGAGCAGTCCATTCAGACATTGGG---------------AGCAGCAGCAGCAGCAGC---AGCCCTGAGCCACAGGAAGTTACAGACACAACTGAGGCCCCCTTTCAAGGGGATCAGAGGTCCCTGGAGTTTCTGCTTCCTCCAGAGCCAGACTACTCACTGCTGCTCACCTTCATCTACAACGGGCGCGTGGTGGGCGAGGCCCAGGTGCAAAGCCTGGATTGCCGCCTTGTGGCTGAGCCCTCAGGCTCTGAGAGC---AGCATGGAGCAGGTGCTGTTCCCCAAGCCTGGCCCACTGGAGCCCACGCAGCGCCTGCTGAGCCAGCTTGAGAGGGGCATCCTAGTGGCCAGCAACCCCCGAGGCCTCTTCGTGCAGCGCCTTTGCCCCATCCCCATCTCCTGGAATGCACCCCAGGCTCCACCTGGGCCAGGCCCGCATCTGCTGCCCAGCAACGAGTGCGTGGAGCTCTTCAGAACCGCCTACTTCTGCAGAGACTTGGTCAGGTACTTTCAGGGCCTGGGCCCCCCACCGAAGTTCCAGGTAACACTGAATTTCTGGGAAGAGAGCCATGGCTCCAGCCATACTCCACAGAATCTTATCACAGTGAAGATGGAGCAGGCCTTTGCCCGATACTTGCTGGAGCAGACT---CCAGAGCAGCAGGCAGCCATTCTGTCCCTGGTGTAG------------------------------------------------------

>Gorilla_ENSGGOG00000016046

ATGGCATCAGGCAGGGCACGCTGCACCCGAAAACTCCGGAACTGGGTGGTGGAGCAAGTGGAGAGTGGGCAGTTTCCCGGAGTGTGCTGGGATGATACAGCTAAGACCATGTTCCGGATTCCCTGGAAACATGCAGGCAAGCAGGACTTCCGGGAGGACCAGGATGCTGCCTTCTTCAAGGCCTGGGCAATATTTAAGGGAAAGTATAAGGAGGGGGACACAGGAGGTCCGGCTGTCTGGAAGACTCGCCTGCGCTGTGCACTCAACAAGAGTTCTGAATTTAAGGAGGTTCCTGAGAGGGGCCGCATGGATGTTGCTGAGCCCTACAAGGTGTATCAGTTGCTGCCACCAGGAACCGTCTCTGGCCAGCCAGGGACTCAGAAATCACCATCAAAGCGACAGCACAGTTCTGTGTCCTCTGAGAGGAAGGAGGAAGAGGATGCCATGCAGAACTGCACACTCAGTCCCTCTGTGCTCCAGGACTCCCTCAATAATGAGGAGGAGGGGGCCAGTGGGGGAGCAGTCCATTCAGACATCGGG------------------AGTAGCAGCAGCAGC---AGCCCTGAGCCACAGGAAGTTACAGACACAACTGAGGCCCCCTTTCAAGGGGATCAGAGGTCCCTGGAGTTTCTGCTTCCTCCAGAGCCAGACTACTCACTGCTGCTCACCTTCATCTACAACGGGCGCGTGGTGGGCGAGGCCCAGGTGCAAAGCCTGGATTGCCGCCTTGTGGCTGAGCCCTCAGGCTCTGAGAGC---AGCATGGAGCAGGTGCTGTTCCCCAAGCCTGGCCCACTGGAGCCCACGCAGCGCCTGCTGAGCCAGCTTGAGAGGGGCATCCTAGTGGCCAGCAACCCCCGAGGCCTCTTCGTGCAGCGCCTTTGCCCCATCCCCATCTCCTGGAATGCACCCCAGGCTCCACCTGGGCCAGGCCCGCATCTGCTGCCCAGCAACGAGTGCGTGGAGCTCTTCAGAACCGCCTACTTCTGCAGAGACTTGGCCAGGTACTTTCAGGGCCTGGGCCCCCCACCAAAGTTCCAGGTAACACTGAATTTCTGGGAAGAGAGCCATGGCTCCAGCCATACTCCACAGAATCTTATCACAGTGAAGATGGAGCAGGCCTTTGCCCGATACTTGCTGGAGCAGACT---CCAGAGCAGCAGGCAGCCATTCTGTCCCTGGTGTAG------------------------------------------------------

>BlackSnubNosedMonkey_ENSRBIG00000041525

ATGGCATCAGGCAGGGCACGCTGTACCCGAAAACTCCGGAACTGGGTGGTGGAGCAAGTGGAGAGCGGGCAGTTCCCCGGAGTGTGCTGGGATGATACAGCTAAGACCATGTTCCGGATTCCCTGGAAGCATGCAGGCAAGCAGGACTTCCGAGAGGACCAGGATGCTGCCTTCTTCAAGGCCTGGGCAATATTTAAGGGAAAGTATAAGGAAGGGGACACAGGAGGCCCCGCTGTCTGGAAGACTCGCCTGCGCTGTGCACTCAACAAGAGTACTGAATTTGAGGAGGTTCCTAAGAGGGGCCGCATGGATGTTGCTGAGCCCTACAAGGTGTATCGGCTGCTGCCACCAGGAACCTTCTATGGCCAGCCAGGGACTCAGAAATCACCATCAAAGCGACAGCACAGTTCTGTGTCCTCTGAGAGGAAGGAGGAAGCGGGTGCCATACAGAACTGCACACTCAGTCCTTCTGTGCTCCAGGACTCCCTCAATAAT------------------TGGGGAGCAGTCCATTCAGACATCGGG------------------AGCAGCAGCAGCAGC---AGCCCTGAGCCACAGGAAGGTACAGACACAACTGAGGCCCCCTTTCAAGGGGATCAGAGGTCCCTGGAGTTTCTGCTTCCTCCAGAGTCAGACTACTCACTGCTGCTCACCTTCATCTACAATGGACGTGTGGTGGGCGAGGCCCAGGTGCAAAGCCTGGATTGCCGCCTTGTGGCTGAGCCCTCAGGCTCTGAGAGC---AGCATGGAGCAGGTGCTGTTCCCCAAGCCTGGCCCACTGGAGCCCACGCAGCGCCTGCTGAGCCAGCTTGAGAGGGGCATCCTGGTGGCCAGCAACCCCCGAGGCCTCTTCGTGCAGCGCCTTTGCCCCATTCCCATCTCCTGGAATGCACCCCAGGCTCCCCCTGGGCCAGGCCCGCATCTGCTGCCCAGCAACGAGTGCGTGGAGCTCTTCAGAACCGCCTACTTCTGCAGAGACTTGGTCAAGTACTTCCAGGGCCTAGGCCCCCCACCGAAGTTCCAGGTAACACTGAATTTCTGGGAAGAGAGCCGTGGCCCCAGCCATACTCCACAGAATCTTATCACAGTGAAGATGGAGCAGGCCTTTGCCCGATACTTGCTGGAGGAGACT---CCAGAGCAGCAGGCAGCCATTCTGTCCCTGGTGTAG------------------------------------------------------

>GoldenSnubNosedMonkey_ENSRROG00000029393

ATGGCATCAGGCAGGGCACGCTGTACCCGAAAACTCCGGAACTGGGTGGTGGAGCAAGTGGAGAGCGGGCAGTTCCCCGGAGTGTGCTGGGATGATACAGCTAAGACCATGTTCCGGATTCCCTGGAAGCATGCAGGCAAGCAGGACTTCCGAGAGGACCAGGATGCTGCCTTCTTCAAGGCCTGGGCAATATTTAAGGGAAAGTATAAGGAAGGGGACACAGGAGGCCCCGCTGTCTGGAAGACTCGCCTGCGCTGTGCACTCAACAAGAGTACTGAATTTGAGGAGGTTCCTAAGAGGGGCCGCATGGATGTTGCTGAGCCCTACAAGGTGTATCGGCTGCTGCCACCAGGAACCTTCTCTGGCCAGCCAGGGACTCAGAAATCACCATCAAAGCGACAGCACAGTTCTGTGTCCTCTGAGAGGAAGGAGGAAGCGGGTGCCATACAGAACTGCACACTCAGTCCTTCTGTGCTCCAGGACTCCCTCAATAATGAGGAGGAGGGGGCCAGTGGGGGAGCAGTCCATTCAGACATCGGG------------------AGCAGCAGCAGCAGC---AGCCCTGAGCCACAGGAAGGTACAGACACAACTGAGGCCCCCTTTCAAGGGGATCAGAGGTCCCTGGAGTTTCTGCTTCCTCCAGAGTCAGACTACTCACTGCTGCTCACCTTCATCTACAACGGACGTGTGGTGGGCGAGGCCCAGGTGCAAAGCCTGGATTGCCGCCTTGTGGCTGAGCCCTCAGGCTCTGAGAGC---AGCATGGAGCAGGTGCTGTTCCCCAAGCCTGGCCCACTGGAGCCCACGCAGCGCCTGCTGAGCCAGCTTGAGAGGGGCATCCTGGTGGCCAGCAACCCCCGAGGCCTCTTCGTGCAGCGCCTTTGCCCCATTCCCATCTCCTGGAATGCACCCCAGGCTCCCCCTGGGCCAGGCCCGCATCTGCTGCCCAGCAACGAGTGCGTGGAGCTCTTCAGAACCGCCTACTTCTGCAGAGACTTGGTCAAGTACTTCCAGGGCCTAGGCCCCCCACCGAAGTTCCAGGTAACACTGAATTTCTGGGAAGAGAGCCGTGGCCCCAGCCATACTCCACAGAATCTTATCACAGTGAAGATGGAGCAGGCCTTTGCCCGATACTTGCTGGAGGAGACT---CCAGAGCAGCAGGCAGCCATTCTGTCCCTGGTGTAG------------------------------------------------------

>PigTailedMacaque_ENSMNEG00000031608

ATGGCATCAGGCAGGGCACGCTGTACCCGAAAACTCCGGAACTGGGTGGTGGAGCAAGTGGAGAGCGGGCAGTTCCCCGGAGTGTGCTGGGATGATACAGCTAAGACCATGTTCCGGATTCCCTGGAAGCATGCAGGCAAGCAGGACTTCCGAGAGGACCAGGATGCTGCCTTCTTCAAGGCCTGGGCAATATTTAAGGGAAAGTATAAGGAAGGGGACACAGGAGGCCCCGCTGTCTGGAAGACTCGCCTGCGCTGTGCACTCAACAAGAGTACCGAATTTGAGGAGGTTCCTAAGAGGGGCCGCATGGATGTTGCTGAGCCCTACAAGGTGTATCGGCTGCTGCCACCAGGAACCTTCTCTGGCCAGCCAGGGACTCAGAAATCACCATCAAAGCGACAGCACAGTTCTGTGTCCTCTGAGAGGAAGGAGGAAGAGGGTGCCATGCAGAACTGCACACTCAGTCCCTCTGTATTCCAGGAATCCCTCAATAATGAGGAGGAGGAGGTCAGTGGGGGAGCACTCCATTCAGACATCGGG------------------AGCAGCAGCAGCAGC---AGCCCTGAGCCACAGGAAGGTACAGACACAACTGAGGCCCCCTTTCAAGGGGATCAGAGGTCCCTGGAGTTTCTGCTTCCTCCAGAGCCAGACTACTCACTGCTGCTCACCTTCATCTACAACGGGCGTGTGGTGGGCGAGGCCCAGGTGCAAAGCCTGGATTGCCGCCTTGTGGCTGAGCCCTCAGGCTCTGAGAGC---AGCATGGAGCAGGTGCTGTTCCCCAAGCCTGGCCCACTGGAGCCCACGCAGCGCCTGCTGAGCCAGCTTGAGAGGGGCATCCTGGTGGCCAGCAACCCCCGAGGCCTCTTCGTGCAGCGCCTTTGCCCCATCCCCATCTTCTGGAATGCACCCCAGGCTCCACCTGGGCCAGGCCCACATCTGCTGCCCAGCAACGAGTGTGTGGAGCTCTTCAGAACCGCCTACTTCTGCAGAGACTTGGCCAAGTACTTCCAGGGCCTAGGCCCCCCACCGAAGTTCCAAGTAACACTGAATTTCTGGGAAGAGAGCCGTGGCCCCAGCCATACTCCACAGAATCTTATCACAGTGAAGATGGAGCAGGCCTTTGCCCGATACTTGCTGGAGGAGACT---CCAGAGCAGCAGGCAGCCATTCTGTCCCTGGTGTAG------------------------------------------------------

>OliveBaboon_ENSPANG00000025056

ATGGCATCAGGCAGGGCACGCTGTACCCGAAAACTCCGGAACTGGGTGGTGGAGCAAGTGGAGAGCGGGCAGTTCCCTGGAGTGTGCTGGGATGATACAGCTAAGACCATGTTCCGGATTCCCTGGAAGCATGCAGGCAAGCAGGACTTCCGAGAGGACCAGGATGCTGCCTTCTTCAAGGCCTGGGCAATATTTAAGGGAAAGTATAAGGAAGGGGACACAGGAGGCCCCGCTGTCTGGAAGACTCGCCTGCGCTGTGCACTCAACAAGAGTCCTGAATTTGAGGAGGTTCCTAAGAGGGGCCGCATGGATGTTGCTGAGCCCTACAAGGTGTATCGGCTGCTGCCACCAGGAACCTTCTCTGGCCAGCCAGGGACTCAGAAATCACCATCAAAGCAACAGCACAGTTCTGTGTCCTCTGAGAGGAAGGAGGAAGAGGGTGCCATGCAGAACTGCACACTCAGTCCCTCTGTGCTCCAGGAATCCCTCAATAATGAGGAGGAGGAGGCCAGTGGGGGAGCACTCCATTCAGACATCGGG------------------AGCAGCAGCAGCAGC---AGCCCTGAGCCACAGGAAGGTACAGACACAACTGAGGCCCCCTTTCAAGGGGATCAGAGGTCCCTGGAGTTTCTGCTTCCTCCAGAGCCAGACTACTCACTGCTGCTCACCTTCATCTACAATGGGCGTGTGGTGGGCGAGGCCCAGGTGCAAAGCCTGGATTGCCGCCTTGTGGCTGAGCCCTCAGGCTCTGAGAGC---AGCATGGAGCAGGTGCTGTTCCCCAAGCCTGGCCCACTGGAGCCCACGCAGCGCCTGCTGAGCCAGCTTGAGAGGGGCATCCTGGTGGCCAGCAACCCCCGAGGCCTCTTCGTGCAGCGCCTTTGCCCCATCCCCATCTCCTGGAATGCACCCCAGGCTCCACCTGGGCCAGGCCCGCATCTGCTGCCTAGCAACGAGTGCGTGGAGCTCTTCAGAACCGCCTACTTCTGCAGAGACTTGGCCAGGTACTTCCAGGGCCTAGGCCCCCCTCCGAAGTTCCAAGTAACACTGAATTTCTGGGAAGAGAGCCGTGGCCCCAGCCATACTCCACAGAATCTTATCACAGTGAAGATGGAGCAGGCCTTTGCCCGATACTTGCTGGAGGAGACT---CCAGAGCAGCAGGCAGCCATTCTGTCCCTGGTGTAG------------------------------------------------------

>GreenMonkey_ENSCSAG00000016071

ATGGCATCAGGCAGGGCACGCTGTACCCGAAAACTCCGGAACTGGGTGGTGGAGCAAGTGGAGAGCGGGCAGTTCCCCGGAGTGTGCTGGGATGATACAGCTAAGACCATGTTCCGGATTCCCTGGAAGCATGCAGGCAAGCAGGACTTCCGAGAGGACCAGGATGCTGCCTTCTTCAAGGCCTGGGCAATATTTAAGGGAAAGTATAAGGAAGGGGACACAGGAGGCCCCGCTGTCTGGAAGACTCGCCTGCGCTGTGCACTCAACAAGAGTCCTGAATTTGAGGAGGTTCCTAAGAGGGGCCGCATGGATGTTGCTGAGCCCTACAAGGTGTATCGGCTGCTGCCACCAGGAACCTTCTCTGGCCAGCCAGGGACTCAGAAATCACCATCAAAGCGACAGCACAGTTCTGTGTCCTCTGAGAGGAAGGAGGAAGAGGGTGCCATGCAGAACTGCACACTCAGTCCCTCTGTGCTCCAGGAATCCCTCAATAATGAGGAGGAGGAGGCCAGTGGGGGAGCACTCCATTCAGACATCGGG------------------AGCAGCAGCAGCAGC---AGCCCTGAGTCACAGGAAGGTACAGACACAACTGAGGCCCCTTTTCAAGGGGATCAGAGGTCCCTGGAGTTTCTGCTTCCTCCAGAGCCAGACTACTCACTGCTGCTCACCTTCATCTACAACGGGCGTGTGGTGGGCGAGGCCCAGGTGCAAAGCCTGGATTGCCGCCTTGTGGCTGAGCCCTCAGGTTCTGAGAGC---AGCATGGAGCAGGTGCTGTTCCCCAAGCCTGGCCCACTGGAGCCCACGCAGCGCCTGCTGAGCCAGCTTGAGAGGGGCATCCTGGTGGCCAGCAACCCCCGAGGCCTCTTCGTGCAGCGCCTTTGCCCCATCCCCATCTCCTGGAATGCACCCCAGGCTCCACCTGGGCCAGGCCCGCATCTGCTGCCCAGCAACGAGTGCGTGGAGCTCTTCAGAACCGCCTACTTCTGCAGAGACTTGGCCAGGTACTTCCAGGGCCTAGGCCCCCCACCGAAGTTCCAAGTAACACTGAATTTCTGGGAAGAGAGCCGTGGCCCCAGCCATACTCCACAGAATCTTATCACAGTGAAGATGGAGCAGGCCTTTGCCCGATACTTGCTGGAGGAGACT---CCAGAGCAGCAGGCAGCCATTCTGTCCCTGGTGTAG------------------------------------------------------

>Drill_ENSMLEG00000004606

ATGGCATCAGGCAGGGCACGCTGTACCCGAAAACTCCGGAACTGGGTGGTGGAGCAAGTGGAGAGCGGGCAGTTCCCTGGAGTGTGCTGGGATGATACAGCTAAGACCATGTTCCGGATTCCCTGGAAGCATGCAGGCAAGCAGGACTTCCGAGAGGACCAGGATGCTGCCTTCTTCAAGGCCTGGGCAATATTTAAGGGAAAGTATAAGGAAGGGGACACAGGAGGCCCCGCTGTCTGGAAGACTCGCCTGCGCTGTGCACTCAACAAGAGTCCTGAATTTGAGGAGGTTCCTAAGAGGGGCCGCATGGATGTTGCTGAGCCCTACAAGGTGTATCGGCTGCTGCCACCAGGAGCCTTCTCTGGCCAGCCAGGGACTCAGAAATCACCATCAAAGCGACAGCACAGTTCTGTGTCCTCTGAGAGGAAGGAGGAAGAGGGTGCCATGCAGAACTGCACACTCAGTCCCTCTGTGCTCCAGGAATCCCTCAATAATGGGGAGGAGGAGGCCAGTGGGGGAGCACTCCATTCAGACATCGGG------------------AGCAGCAGCAGCAGC---AGCCCTGAGCCACAGGAAGGTACAGACACAACTGAGGCCCCCTTTCAAGGGGATCAGAGGTCCCTGGAGTTTCTGCTTCCTCCAGAGCCAGACTACTCACTGCTGCTCACCTTCATCTACAACGGGCGTGTGGTGGGCGAGGCCCAGGTGCAAAGCCTGGATTGCCGCCTTGTGGCTGAGCCCTCAGGCTCTGAGAGC---AGCATGGAGCAGGTGCTGTTCCCCAAGCCTGGCCCACTGGAGCCCACGCAGCGCCTGCTGAGCCAGCTTGAGAGGGGCATCCTGGTGGCCAGCAACCCCCGAGGCCTCTTCGTGCAGCGCCTTTGCCCCATCCCCATCTCCTGGAACGCACCCCAGGCTCCACCTGGGCCAGGCCCACATCTGCTGCCCAGCAACGAGTGCGTGGAGCTCTTCAGAACCGCCTACTTCTGCAGAGACTTGGCCAGGTACTTCCAGGGCCTAGGCCCCCCTCCGAAGTTCCAAGTAACACTGAATTTCTGGGAAGAGAGCCGTGGCCCCAGCCATACTCCACAGAATCTTATCACAGTGAAGATGGAGCAGGCCTTTGCCCGATACTTGCTGGAGGAGACT---CCAGAGCAGCAGGCAGCCATTCTGTCCCTGGTGTAG------------------------------------------------------
