# Supplementary material for: Positive selection-driven fixation of a hominin-specific amino acid mutation related to dephosphorylation in IRF9
Source: BMC Ecol Evol. 2022 Nov 10;22:132. doi: 10.1186/s12862-022-02088-5 (PMC9650800; doi:10.1186/s12862-022-02088-5)
Supplement: Supplementary file 2 — Additional file 2. Recombination test by the SimPlot v3.5.1. [file 12862_2022_2088_MOESM2_ESM.pdf]

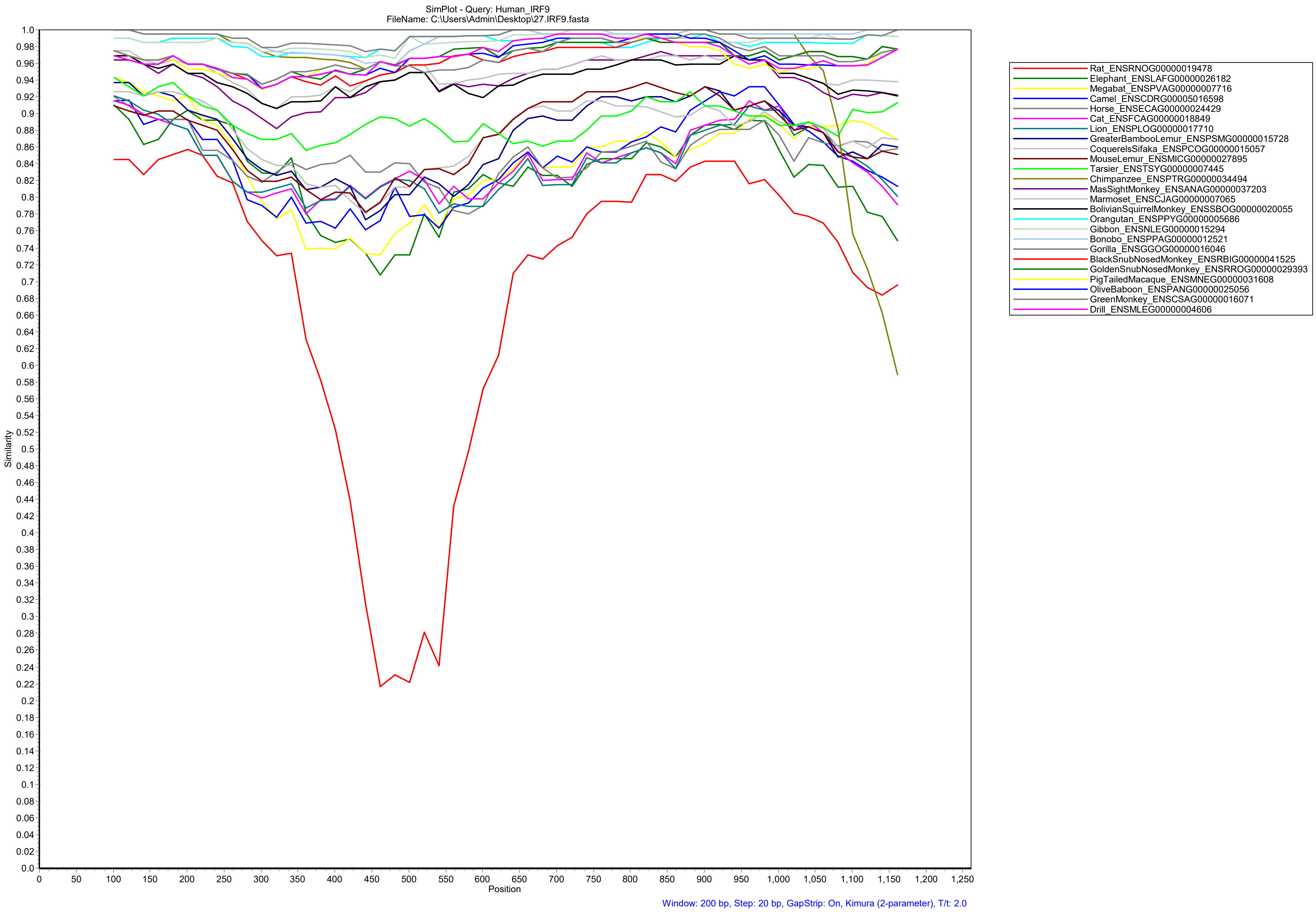
SimPlot - Query: Human_IRF9
FileName: C:\Users\Admin\Desktop\27.IRF9.fasta
Similarity
Position
Rat_ENSRNOG00000019478
Elephant_ENSLAFG00000026182
Megabat_ENSPVAG00000007716
Camel_ENSCDRG00005016598
Horse_ENSECAG00000024429
Cat_ENSFCAG00000018849
Lion_ENSPLOG00000017710
GreaterBambooLemur_ENSPSMG00000015728
CoquerelsSifaka_ENSPCOG00000015057
MouseLemur_ENSMICG00000027895
Tarsier_ENSTSYG00000007445
Chimpanzee_ENSPTRG00000034494
MasSightMonkey_ENSANAG00000037203
Marmoset_ENSCJAG00000007065
BolivianSquirrelMonkey_ENSSBOG00000020055
Orangutan_ENSPPYG00000005686
Gibbon_ENSNLEG00000015294
Bonobo_ENSPPAG00000012521
Gorilla_ENSGGOG00000016046
BlackSnubNosedMonkey_ENSRBIG00000041525
GoldenSnubNosedMonkey_ENSRROG00000029393
PigTailedMacaque_ENSMNEG00000031608
OliveBaboon_ENSPANG00000025056
GreenMonkey_ENSCSAG00000016071
Drill_ENSMLEG00000004606
Window: 200 bp, Step: 20 bp, GapStrip: On, Kimura (2-parameter), T/t: 2.0
