# Supplementary material for: Positive selection-driven fixation of a hominin-specific amino acid mutation related to dephosphorylation in IRF9
Source: BMC Ecol Evol. 2022 Nov 10;22:132. doi: 10.1186/s12862-022-02088-5 (PMC9650800; doi:10.1186/s12862-022-02088-5)
Supplement: Supplementary file 3 — Additional file 3. The aBSREL (HyPhy) results after correcting for multiple testing with the Holm-Bonferroni correction. [file 12862_2022_2088_MOESM3_ESM.pdf]

adaptive Branch Site REL

results summary

INPUT DATA | 6212ee834040572c69c542f2 | 26 sequences | 419 sites

Export

aBSREL **found evidence** of episodic diversifying selection on **1** out of **46** branches in your phylogeny.

A total of **1** branches were formally tested for diversifying selection. Significance was assessed using the Likelihood Ratio Test at a threshold of p ≤ 0.05, after correcting for multiple testing. Significance and number of rate categories inferred at each branch are provided in the **detailed results** table.

See **here** for more information about this method.

Please cite **PMID 25697341** if you use this result in a publication, presentation, or other scientific work.

Tree summary

ω rate classes	# of branches	% of branches	% of tree length	# under selection
1	39	85%	2.2%	0
2	7	15%	98%	1

This table contains a summary of the inferred aBSREL model complexity. Each row provides information about the branches that were best described by the given number of ω rate categories.

Fitted tree

Options

Export

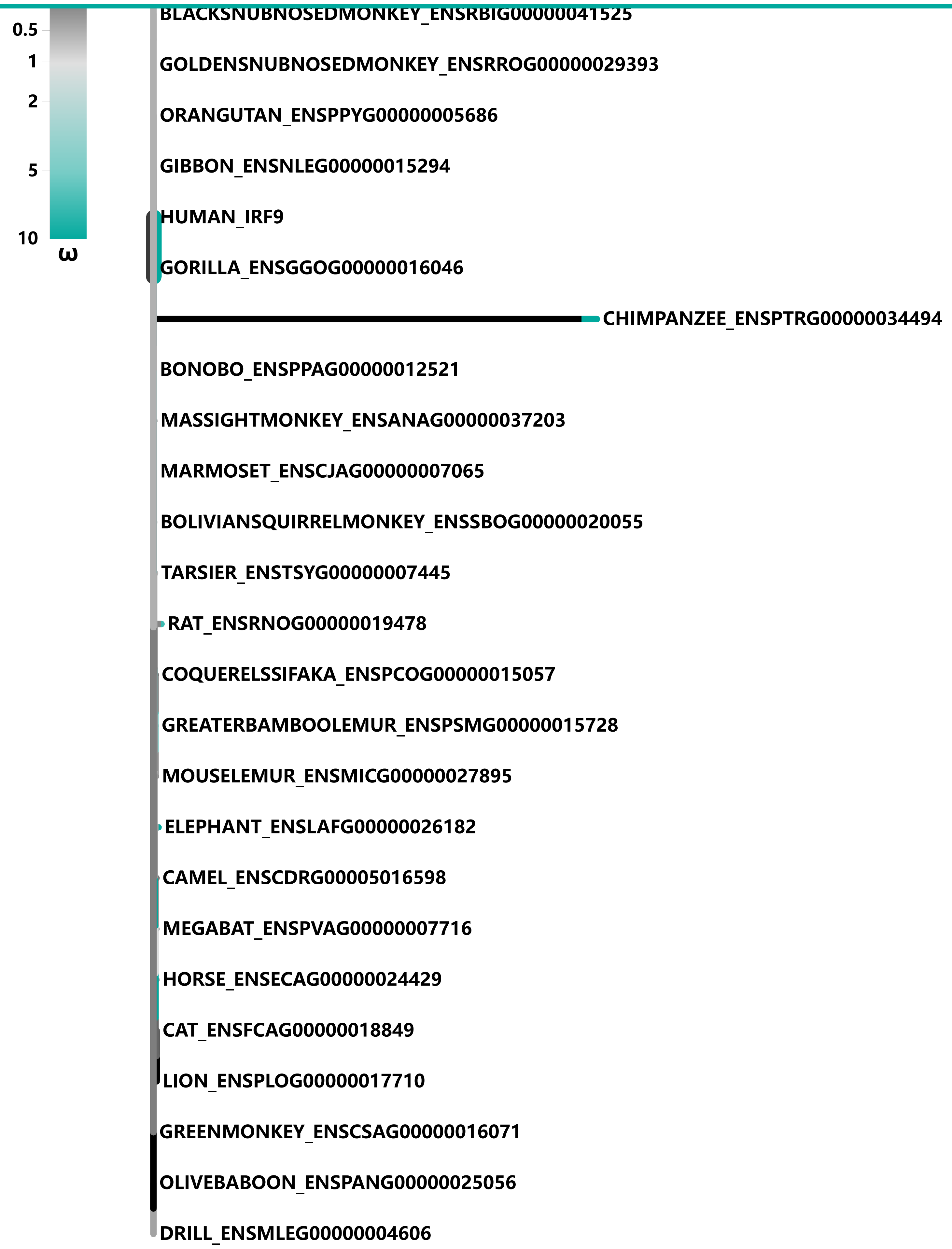

Detailed results

Name	B	LRT	Test p-value	Uncorrected p-value	ω distribution over sites	
HUMAN_IRF9	0.0000	6.8821	0.0114	0.0114	ω_1 = 0.0182 (99%) ω_2 = 96.2 (1.2%)	
BLACKSNUBNOSEDMONKEY_ENSRBIG00000041525	0.0000	test not run	1.0000	1.0000	ω_1 = 0.817 (100%)	
BOLIVIANSQUIRRELMONKEY_ENSSBOG00000020055	0.0000	test not run	1.0000	1.0000	ω_1 = 0.355 (100%)	
BONOBO_ENSPPAG00000012521	0.0000	test not run	1.0000	1.0000	ω_1 = 1.00 (100%)	
CAMEL_ENSCDRG00005016598	0.0000	test not run	1.0000	1.0000	ω_1 = 0.171 (100%)	

CAT_ENSFCAG00000018849	0.0000	run	1.0000	1.0000	ω_1 = 0.0774 (100%)	
CHIMPANZEE_ENSPTRG00000034494	0.0000	test not run	1.0000	1.0000	ω_1 = 0.00 (96%)<br>ω_2 = 100000 (4.3%)	
COQUERELSSIFAKA_ENSPCOG00000015057	0.0000	test not run	1.0000	1.0000	ω_1 = 0.177 (100%)	
DRILL_ENSMLEG00000004606	0.0000	test not run	1.0000	1.0000	ω_1 = 0.411 (100%)	
ELEPHANT_ENSLAFG00000026182	0.0000	test not run	1.0000	1.0000	ω_1 = 0.410 (97%)<br>ω_2 = 31.6 (2.6%)	
GIBBON_ENSNLEG00000015294	0.0000	test not run	1.0000	1.0000	ω_1 = 0.224 (100%)	
GOLDENSNUBNOSEDMONKEY_ENSRROG00000029393	0.0000	test not run	1.0000	1.0000	ω_1 = 1.00 (100%)	
GORILLA_ENSGGOG00000016046	0.0000	test not run	1.0000	1.0000	ω_1 = 0.00 (100%)	
GREATERBAMBOOLEMUR_ENSPSMG00000015728	0.0000	test not run	1.0000	1.0000	ω_1 = 0.355 (100%)	
GREENMONKEY_ENSCSAG00000016071	0.0000	test not run	1.0000	1.0000	ω_1 = 0.200 (100%)	
HORSE_ENSECAG00000024429	0.0000	test not run	1.0000	1.0000	ω_1 = 0.231 (100%)	
LION_ENSPLOG00000017710	0.0000	test not run	1.0000	1.0000	ω_1 = 0.00 (100%)	
MARMOSET_ENSCJAG00000007065	0.0000	test not run	1.0000	1.0000	ω_1 = 0.0466 (100%)	
MASSIGHTMONKEY_ENSANAG00000037203	0.0000	test not run	1.0000	1.0000	ω_1 = 0.195 (100%)	
MEGABAT_ENSPVAG00000007716	0.0000	test not run	1.0000	1.0000	ω_1 = 0.292 (100%)	
MOUSELEMUR_ENSMICG00000027895	0.0000	test not run	1.0000	1.0000	ω_1 = 0.285 (100%)	
Node11	0.0000	test not run	1.0000	1.0000	ω_1 = 0.141 (100%)	
Node12	0.0000	test not run	1.0000	1.0000	ω_1 = 0.436 (100%)	
Node14	0.0000	test not run	1.0000	1.0000	ω_1 = 0.198 (100%)	
Node16	0.0000	test not run	1.0000	1.0000	ω_1 = 0.264 (100%)	
Node20	0.0000	test not run	1.0000	1.0000	ω_1 = 10000000000 (100%)	
Node23	0.0000	test not run	1.0000	1.0000	ω_1 = 0.242 (99%)<br>ω_2 = 65.7 (0.54%)	
Node24	0.0000	test not run	1.0000	1.0000	ω_1 = 0.0466 (100%)	
Node29	0.0000	test not run	1.0000	1.0000	ω_1 = 0.300 (100%)	

Node31	0.0000	run	1.0000	1.0000	ω_1 = 0.539 (100%)	
Node32	0.0000	test not run	1.0000	1.0000	ω_1 = 0.161 (100%)	
Node34	0.0000	test not run	1.0000	1.0000	ω_1 = 0.00 (88%)<br>ω_2 = 7.15 (12%)	
Node36	0.0000	test not run	1.0000	1.0000	ω_1 = 4.14 (100%)	
Node39	0.0000	test not run	1.0000	1.0000	ω_1 = 0.325 (100%)	
Node4	0.0000	test not run	1.0000	1.0000	ω_1 = 0.00 (100%)	
Node41	0.0000	test not run	1.0000	1.0000	ω_1 = 0.00 (92%)<br>ω_2 = 11.8 (8.0%)	
Node43	0.0000	test not run	1.0000	1.0000	ω_1 = 1.04 (100%)	
Node45	0.0000	test not run	1.0000	1.0000	ω_1 = 10000000000 (100%)	
Node47	0.0000	test not run	1.0000	1.0000	ω_1 = 0.106 (100%)	
Node7	0.0000	test not run	1.0000	1.0000	ω_1 = 1.26 (100%)	
Node8	0.0000	test not run	1.0000	1.0000	ω_1 = 0.597 (100%)	
OLIVEBABOON_ENSPANG00000025056	0.0000	test not run	1.0000	1.0000	ω_1 = 0.200 (100%)	
ORANGUTAN_ENSPPYG00000005686	0.0000	test not run	1.0000	1.0000	ω_1 = 0.572 (100%)	
PIGTAILEDMACAQUE_ENSMNEG00000031608	0.0000	test not run	1.0000	1.0000	ω_1 = 0.510 (100%)	
RAT_ENSRNOG00000019478	0.0000	test not run	1.0000	1.0000	ω_1 = 0.241 (89%)<br>ω_2 = 7.27 (11%)	
TARSIER_ENSTSYG00000007445	0.0000	test not run	1.0000	1.0000	ω_1 = 0.562 (100%)	

Model fits

Model	AIC_C	log L	Parameters
Nucleotide GTR	14267.90	-7076.85	57
Baseline MG94xREV	13878.64	-6832.27	106
Full adaptive model	13654.02	-6705.66	120

This table reports a statistical summary of the models fit to the data. Here, **Baseline MG94xREV** refers to the MG94xREV baseline model that infers a single ω rate category per branch. **Full adaptive model** refers to the adaptive aBSREL model that infers an optimized number of ω rate categories per branch.

Tweets by @hyphy_software

HyPhy
@hyphy_software

Have a small dataset but still want to test for selection per-site? FEL with parametric bootstrap is now available in HyPhy 2.5.33 and Datamonkey. ♻️ Full description can be found here ➡️ hyphy.org/news/

Oct 16, 2021

HyPhy Retweeted

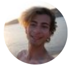

Spyros Lytras
@SpyrosLytras

Replying to @SpyrosLytras

We used an array of methods implemented in @hyphy_software to search for site-, branch- and ORF-specific selection in the phylogenetic clade SARS-CoV-2 emerged from (we refer to as the 'nCoV' clade) 9/18

Embed | View on Twitter
