# Supplementary material for: Positive selection-driven fixation of a hominin-specific amino acid mutation related to dephosphorylation in IRF9
Source: BMC Ecol Evol. 2022 Nov 10;22:132. doi: 10.1186/s12862-022-02088-5 (PMC9650800; doi:10.1186/s12862-022-02088-5)
Supplement: Supplementary file 4 — Additional file 4. The MEME (HyPhy) results. [file 12862_2022_2088_MOESM4_ESM.pdf]

Mixed Effects Model of Evolution

results summary

INPUT DATA | 621332434040572c69c5435d | 26 sequences | 419 sites

Export

MEME **found evidence** of

episodic positive/diversifying selection at 17 sites

with p-value threshold of 0.05.

See here for more information about the MEME method.

Please cite PMID 22807683 if you use this result in a publication, presentation, or other scientific work.

MEME Table

Sites that yielded a statistically significant result are highlighted in green.

Showing entries 129 through 148 out of 419.

Export Table to CSV

Site	Partition	α	β^-	p^-	β^+	p^+	LRT	p-value	# branches under selection	Total branch length	MEME LogL	FEL LogL
129	1	0.86	0.00	0.89	39.85	0.11	10.02	0.00	3.00	0.00	-34.93	-29.16
130	1	0.37	0.00	1.00	0.55	0.00	0.00	0.67	0.00	0.00	-10.93	-10.93
131	1	0.00	0.00	0.74	3.57	0.26	4.58	0.05	2.00	0.00	-19.71	-18.75
132	1	0.00	0.00	1.00	0.00	0.00	0.00	1.00	0.00	0.00	0.00	0.00
133	1	0.01	0.01	0.84	34.25	0.16	7.80	0.01	4.00	0.00	-27.66	-24.58
134	1	3.78	1.21	0.97	15.32	0.03	0.04	0.61	0.00	0.00	-33.79	-33.77
135	1	3.44	1.10	1.00	5.17	0.00	0.00	0.67	0.00	0.00	-25.28	-25.28
136	1	0.54	0.00	0.93	4.69	0.07	0.47	0.44	1.00	0.00	-14.80	-14.52
137	1	1.32	0.22	1.00	1.97	0.00	0.00	0.67	0.00	0.00	-17.70	-17.70
138	1	0.00	0.00	0.00	0.54	1.00	1.28	0.27	2.00	0.00	-12.43	-12.43
139	1	2.44	0.00	1.00	3.66	0.00	0.00	0.67	0.00	0.00	-15.86	-15.86
140	1	0.40	0.02	0.00	0.46	1.00	0.00	0.65	0.00	0.00	-16.49	-16.49
141	1	0.00	0.00	0.00	0.24	1.00	0.36	0.47	1.00	0.00	-8.83	-8.83
142	1	0.00	0.00	0.53	0.47	0.47	0.52	0.42	1.00	0.00	-9.23	-9.21
143	1	3.99	0.77	1.00	5.98	0.00	0.00	0.67	0.00	0.00	-26.06	-26.06
144	1	0.00	0.00	1.00	0.00	0.00	0.00	1.00	0.00	0.00	0.00	0.00
145	1	0.00	0.00	0.00	0.61	1.00	1.49	0.24	1.00	0.00	-15.05	-15.05
146	1	0.00	0.00	0.50	3.39	0.50	2.00	0.18	1.00	0.00	-24.65	-24.57

MEME Site Plot

Y-axis: p-value

Export Chart to PNG | Export Chart to SVG

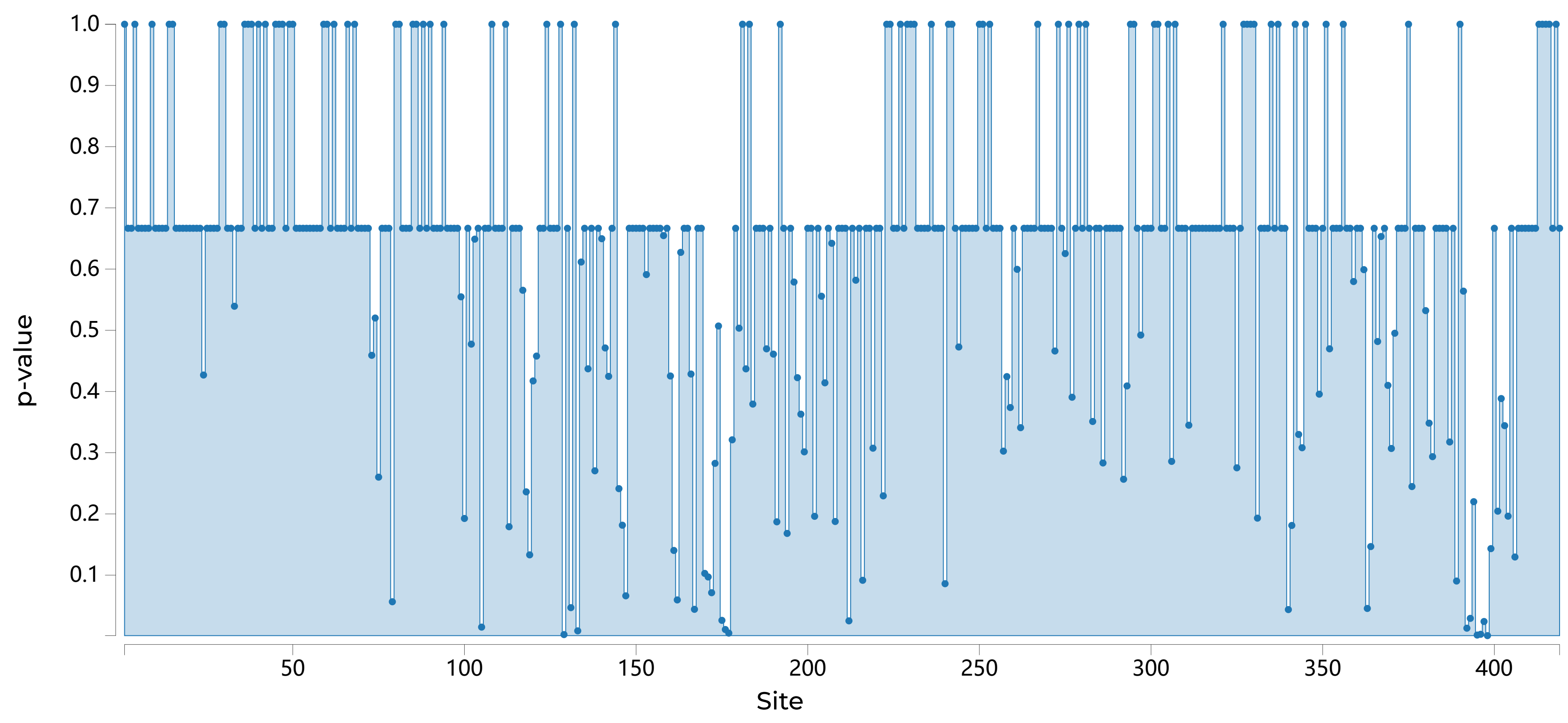

Fitted tree

Options | Export

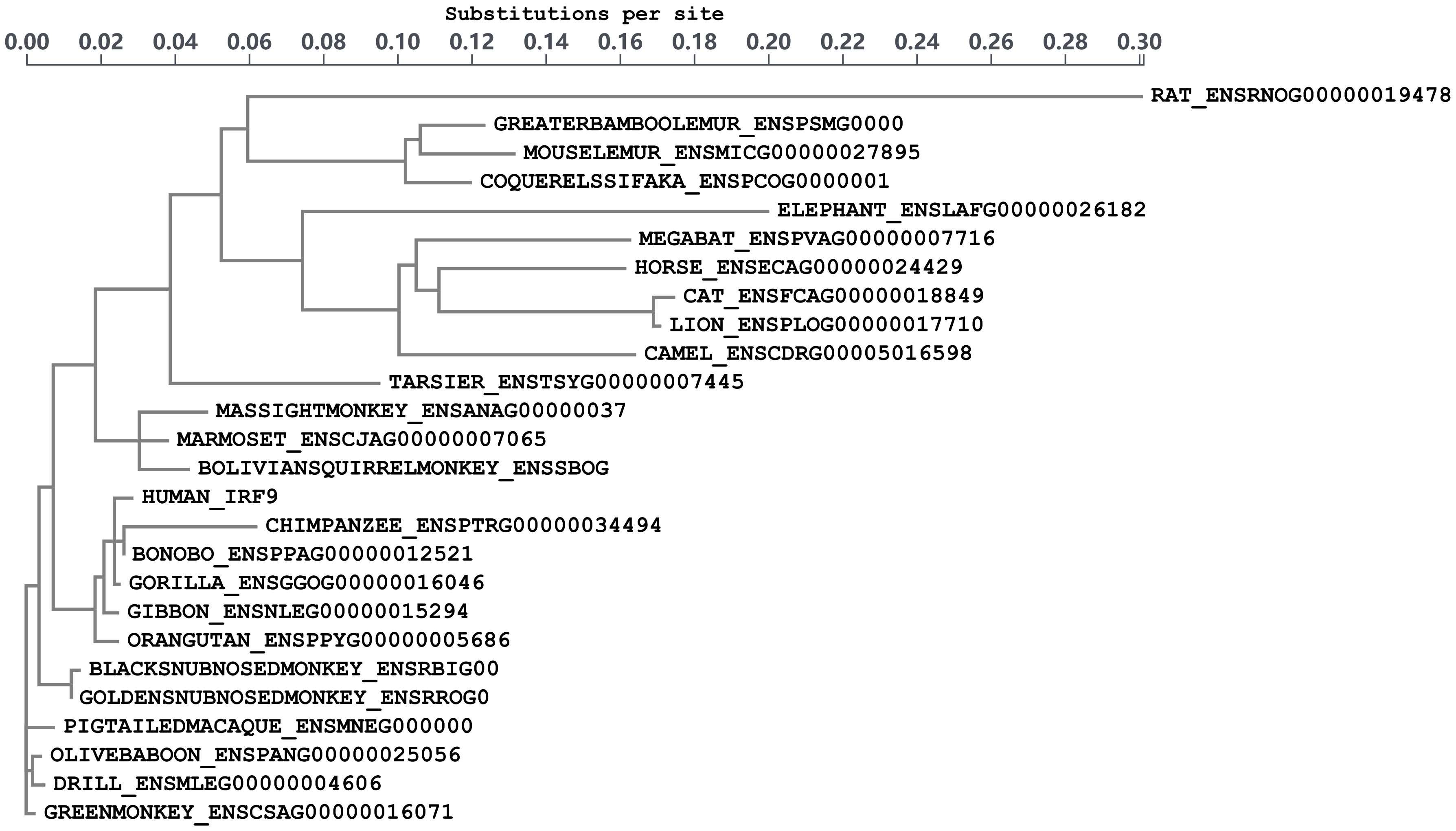

MEME Branch EBF Table

Showing entries 1 through 10 out of 15640.

Export Table to CSV

Site	Partition	Branch	EBF
10	0	BLACKSNUBNOSEDMONKEY_ENSRBIG00000041525	1

101	0	BLACKSNUBNOSEDMONKEY_ENSRBIG00000041525	1
102	0	BLACKSNUBNOSEDMONKEY_ENSRBIG00000041525	0.95
103	0	BLACKSNUBNOSEDMONKEY_ENSRBIG00000041525	0.92
104	0	BLACKSNUBNOSEDMONKEY_ENSRBIG00000041525	1
105	0	BLACKSNUBNOSEDMONKEY_ENSRBIG00000041525	0.1
106	0	BLACKSNUBNOSEDMONKEY_ENSRBIG00000041525	1
107	0	BLACKSNUBNOSEDMONKEY_ENSRBIG00000041525	1
109	0	BLACKSNUBNOSEDMONKEY_ENSRBIG00000041525	1

Model fits

Model	AIC_C	log L	Parameters	Rate distributions
Nucleotide GTR	14267.90	-7076.85	57	
Global MG94xREV	13869.04	-6873.17	61	non-synonymous/synonymous rate ratio for *test*<br>100% @ 0.330

This table reports a statistical summary of the models fit to the data. Here, **MG94** refers to the MG94xREV baseline model that infers a single ω rate category per branch.

Datamonkey is funded jointly by MIDAS and NIH award R01 GM093939

@hyphy_software

Have a small dataset but still want to test for selection per-site? 🥵FEL with parametric bootstrap is now available in HyPhy 2.5.33 and Datamonkey. ♻️ Full description can be found here ➡️ hyphy.org/news/

Oct 16, 2021

HyPhy Retweeted

Spyros Lytras
@SpyrosLytras

Replying to @SpyrosLytras

We used an array of methods implemented in @hyphy_software to search for site-, branch- and ORF-specific selection in the phylogenetic clade SARS-CoV-2 emerged from (we refer to as the 'nCoV' clade) 9/18

Embed View on Twitter
