# Supplementary material for: Positive selection-driven fixation of a hominin-specific amino acid mutation related to dephosphorylation in IRF9
Source: BMC Ecol Evol. 2022 Nov 10;22:132. doi: 10.1186/s12862-022-02088-5 (PMC9650800; doi:10.1186/s12862-022-02088-5)
Supplement: Supplementary file 5 — Additional file 5. The FEL (HyPhy) results. [file 12862_2022_2088_MOESM5_ESM.pdf]

FEL

Fixed Effects Likelihood

FEL analysis was performed on the alignment from /home/datamonkey/datamonkey-js-server/production/app/fel/output/621ec8277a2afa525226b7ea. Statistical significance is evaluated based on the asymptotic χ^2 distribution. This analysis **includes** site to site synonymous rate variation. .

Export

Suggested citation: Not So Different After All: A Comparison of Methods for Detecting Amino Acid Sites Under Selection (2005). Mol Biol Evol 22 (5): 1208-1222

p-value threshold

0.05 | Update

26
sequences in the alignment

419
codon sites in the alignment

1
partitions

46
median branches/partition used for testing

287
non-invariant sites tested

N/A
parametric bootstrap replicates

3
Sites under diversifying positive selection at p≤0.05

1
Sites under purifying selection at p≤0.05

Show

- [x] Diversifying
- [x] Purifying
- [x] Neutral
- [x] Invariable

Plot type

alpha/beta site-level estimates

Figure 1. Maximum likelihood estimates of synonymous (α) and non-synonymous rates (β) at each site shown as bars. The line shows the estimates under the null model (α=β). Estimates above 10 are censored at this value.

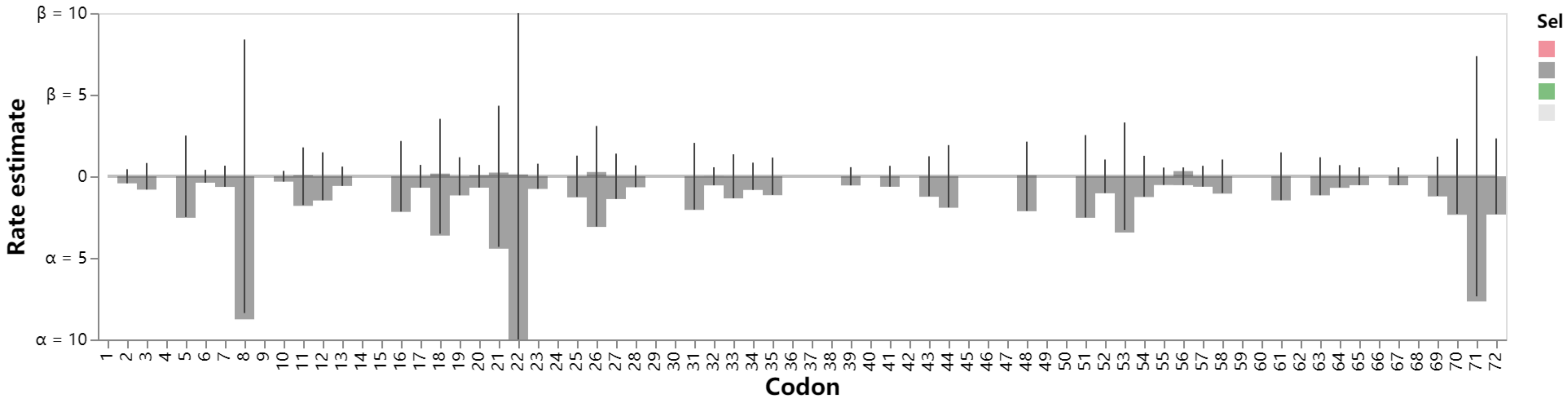

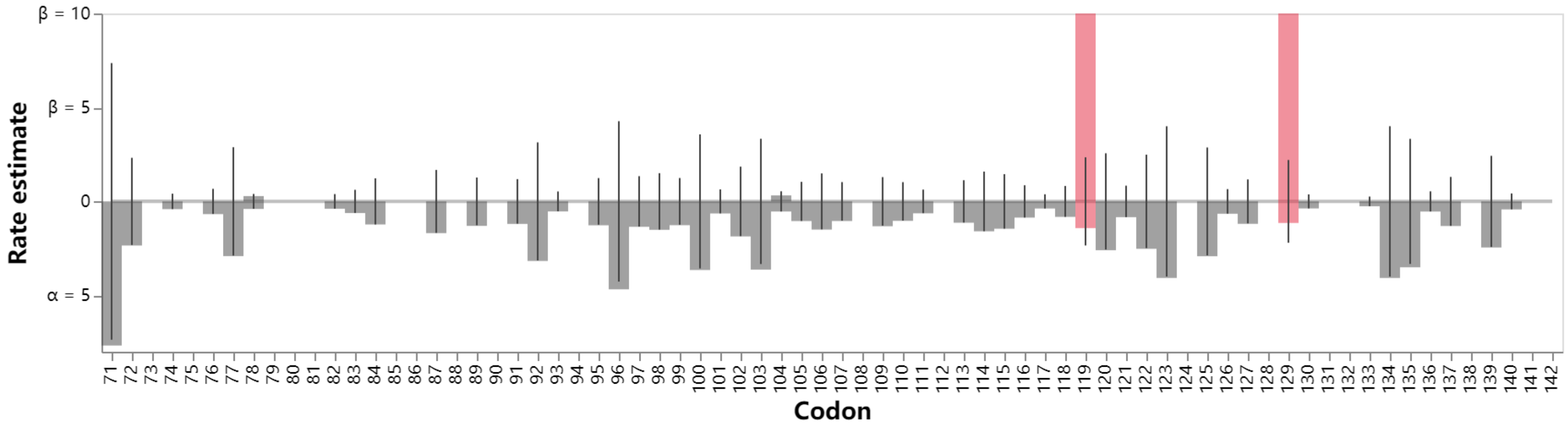

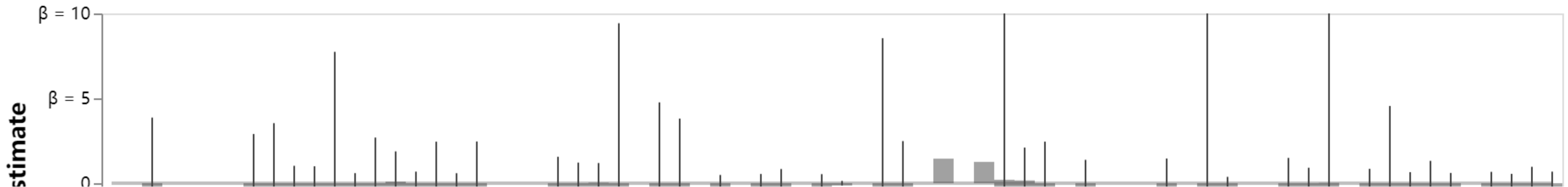

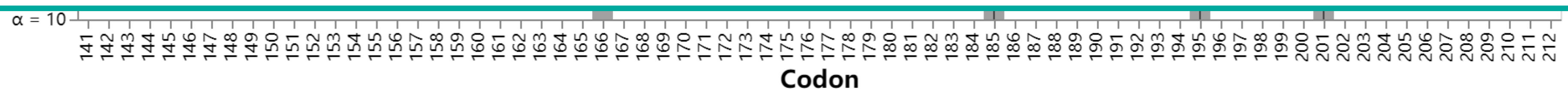

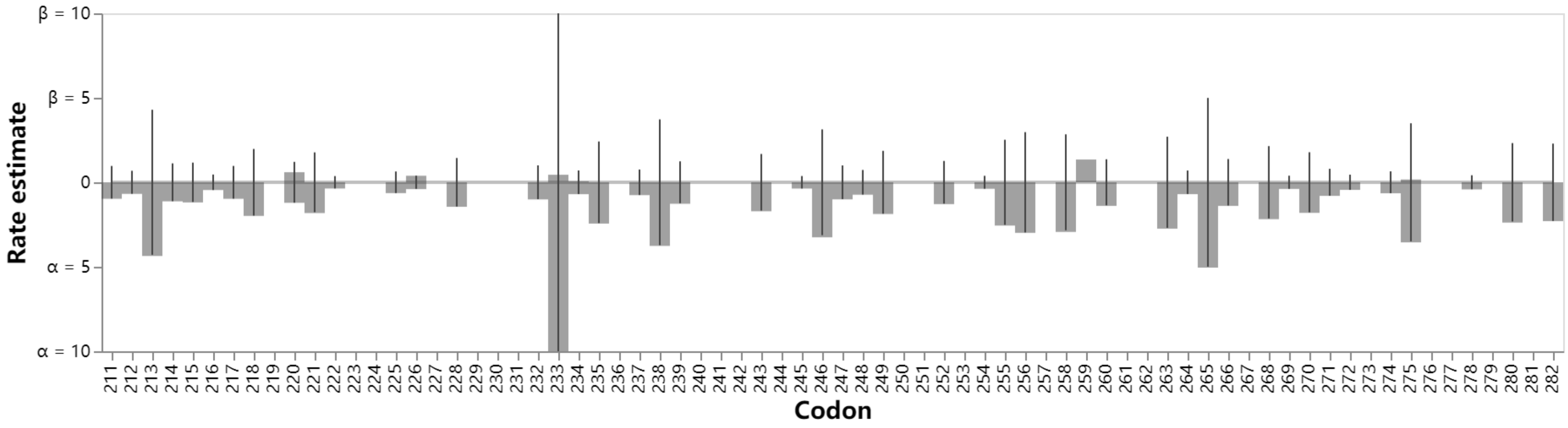

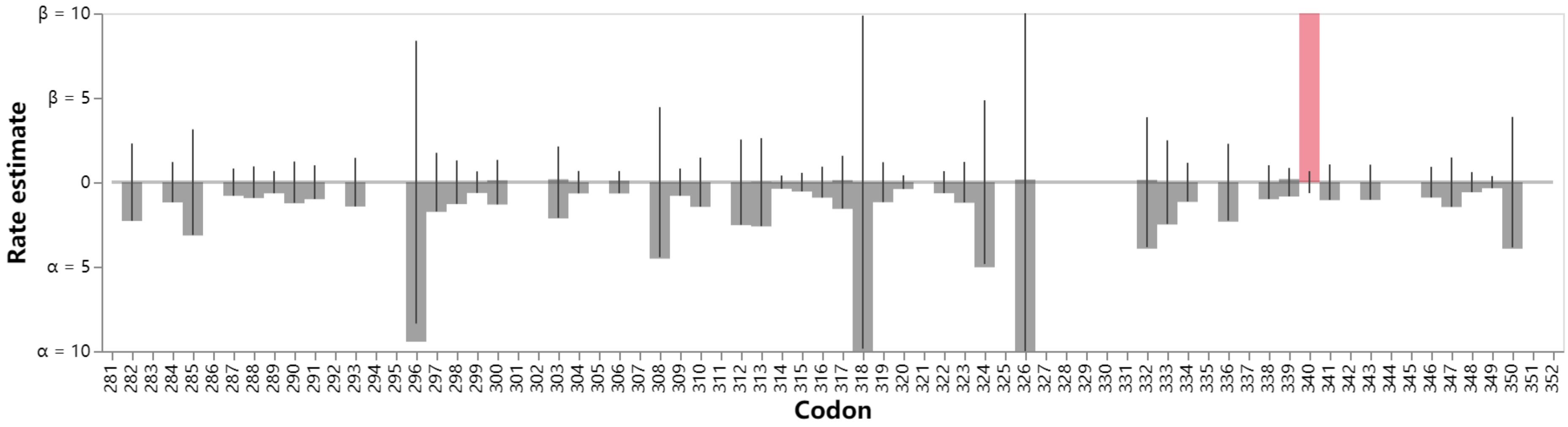

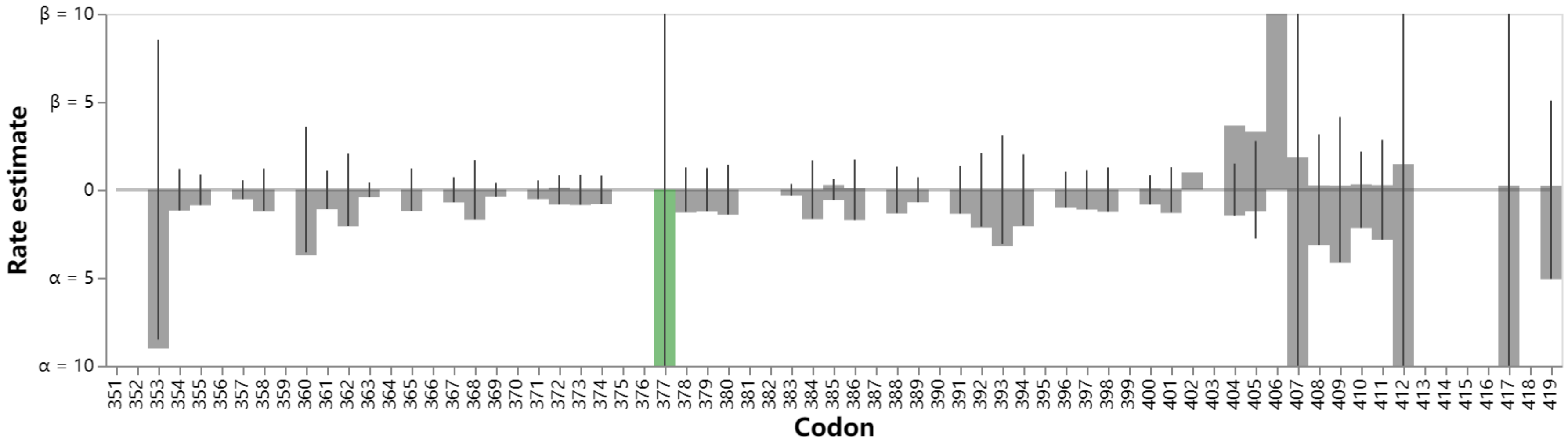

Table 1. Detailed site-by-site results from the FEL analysis

partition	codon	alpha	beta	alpha=beta	LRT	p-value	Total branch length	class
1	129	1.148	383.280	2.201	16.854	**0.0000**	0.116	Diversifying
1	340	0.000	116.021	0.649	10.086	**0.0015**	0.040	Diversifying
1	119	1.422	86.249	2.345	5.483	**0.0192**	0.109	Diversifying
1	377	10000.000	0.000	53.610	4.645	**0.0312**	1.686	Purifying
1	326	31.941	0.157	25.276	0.862	0.3532	0.782	Neutral
1	201	36.869	0.021	28.104	0.825	0.3637	0.888	Neutral
1	233	33.975	0.445	20.921	0.758	0.3839	0.769	Neutral
1	22	16.072	0.112	13.996	0.493	0.4824	0.433	Neutral
1	195	16.843	0.000	14.298	0.381	0.5370	0.462	Neutral
1	296	9.447	0.000	8.370	0.284	0.5941	0.259	Neutral
1	8	8.766	0.000	8.381	0.270	0.6031	0.275	Neutral

▸ **Table column definitions**

View tree for

Partition 1

1024 x 800 | Resize

Tree dimension (height x width in pixels), leave blank to auto-scale

Branches that are shown in red color are those that were included in testing for selection

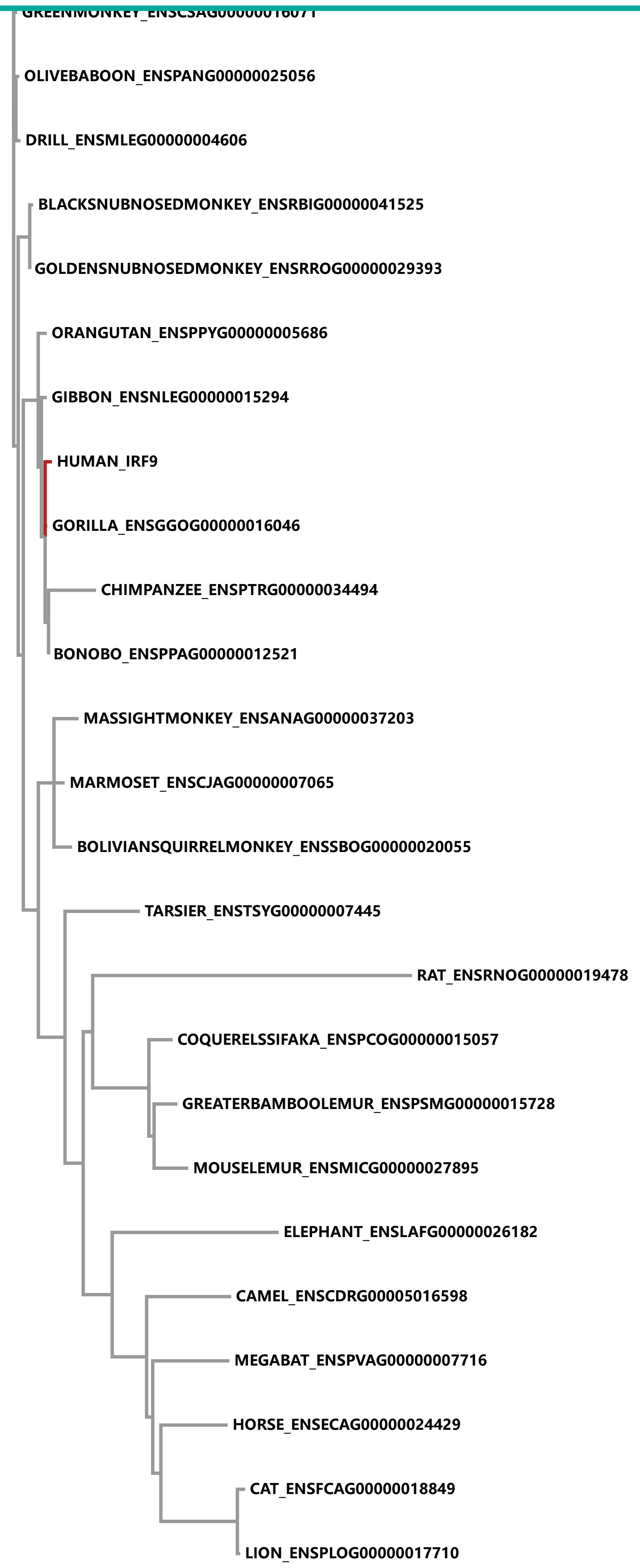

Datamonkey is funded jointly by MIDAS and NIH award R01 GM093939

@hyphy_software

Have a small dataset but still want to test for selection per-site? 🥶FEL with parametric bootstrap is now available in HyPhy 2.5.33 and Datamonkey. ♻️ Full description can be found here ➡️ hyphy.org/news/

Oct 16, 2021

HyPhy Retweeted

Spyros Lytras
@SpyrosLytras

Replying to @SpyrosLytras

We used an array of methods implemented in @hyphy_software to search for site-, branch- and ORF-specific selection in the phylogenetic clade SARS-CoV-2 emerged from (we refer to as the 'nCoV' clade) 9/18

Embed
View on Twitter
