# Supplementary figures and images for: Positive selection-driven fixation of a hominin-specific amino acid mutation related to dephosphorylation in IRF9
Source: BMC Ecol Evol. 2022 Nov 10;22:132. doi: 10.1186/s12862-022-02088-5 (PMC9650800; doi:10.1186/s12862-022-02088-5)

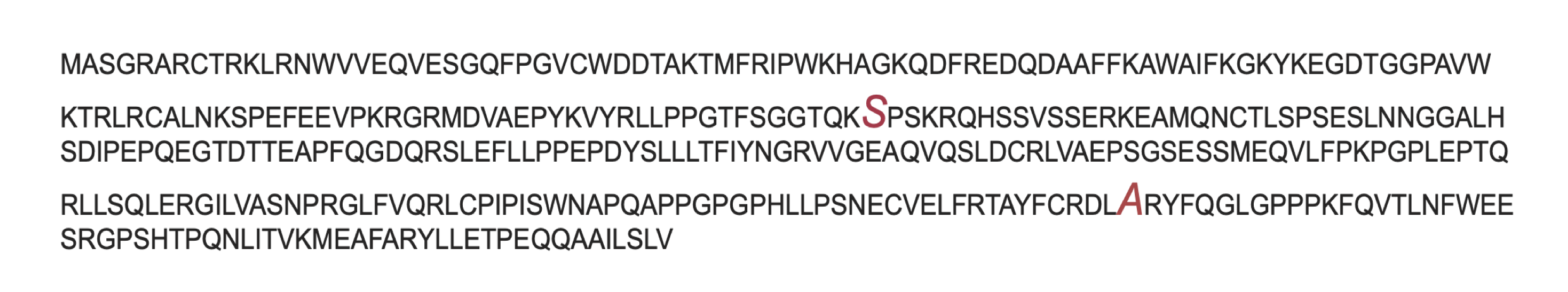

Supplement: Supplementary file 6 — Additional file 6. The ML inference of ancestral sequence by the Maximum likelihood method in PAML software. The reconstructed ancestral states of mammalian species are highlighted with larger red letters. [file 12862_2022_2088_MOESM6_ESM.png]

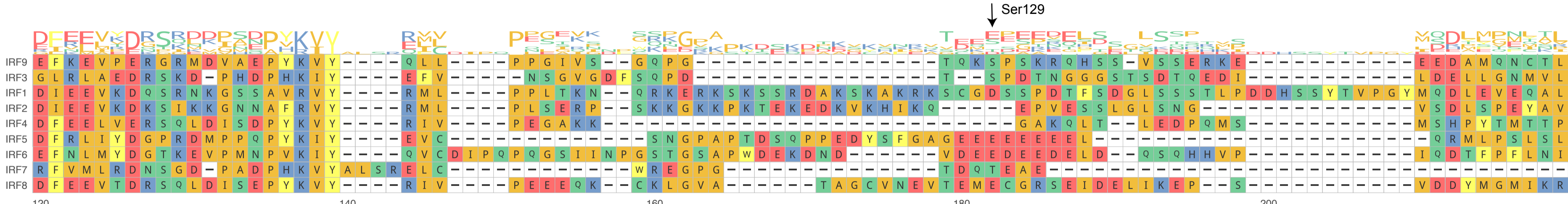

120

140

160

180

200

Supplement: Supplementary file 7 — Additional file 7. The CLUSTAL_W alignment of IRF proteins with a focus on the linker region around site 129 of IRF9. [file 12862_2022_2088_MOESM7_ESM.pdf]

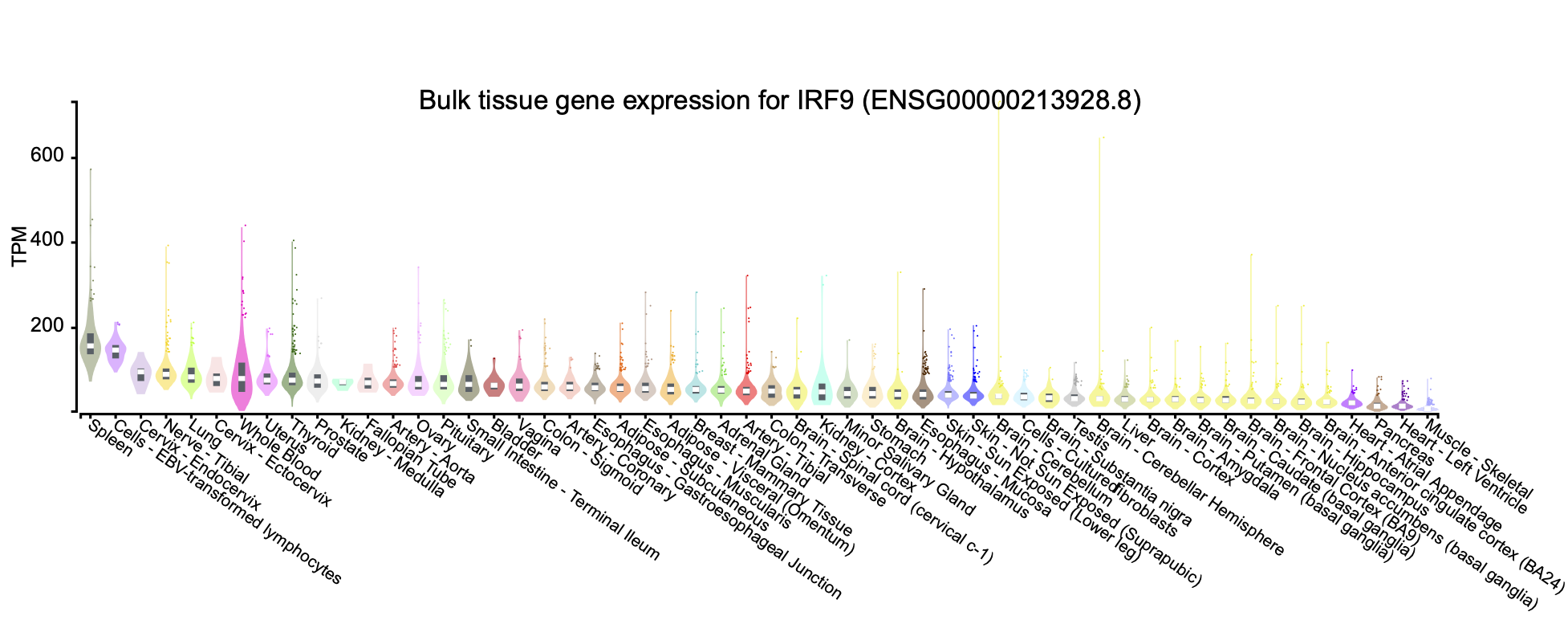

Supplement: Supplementary file 8 — Additional file 8. The population RNAseq expression quantification of IRF9. The boxplots show the expression levels within different tissues/organs/cells. [file 12862_2022_2088_MOESM8_ESM.png]

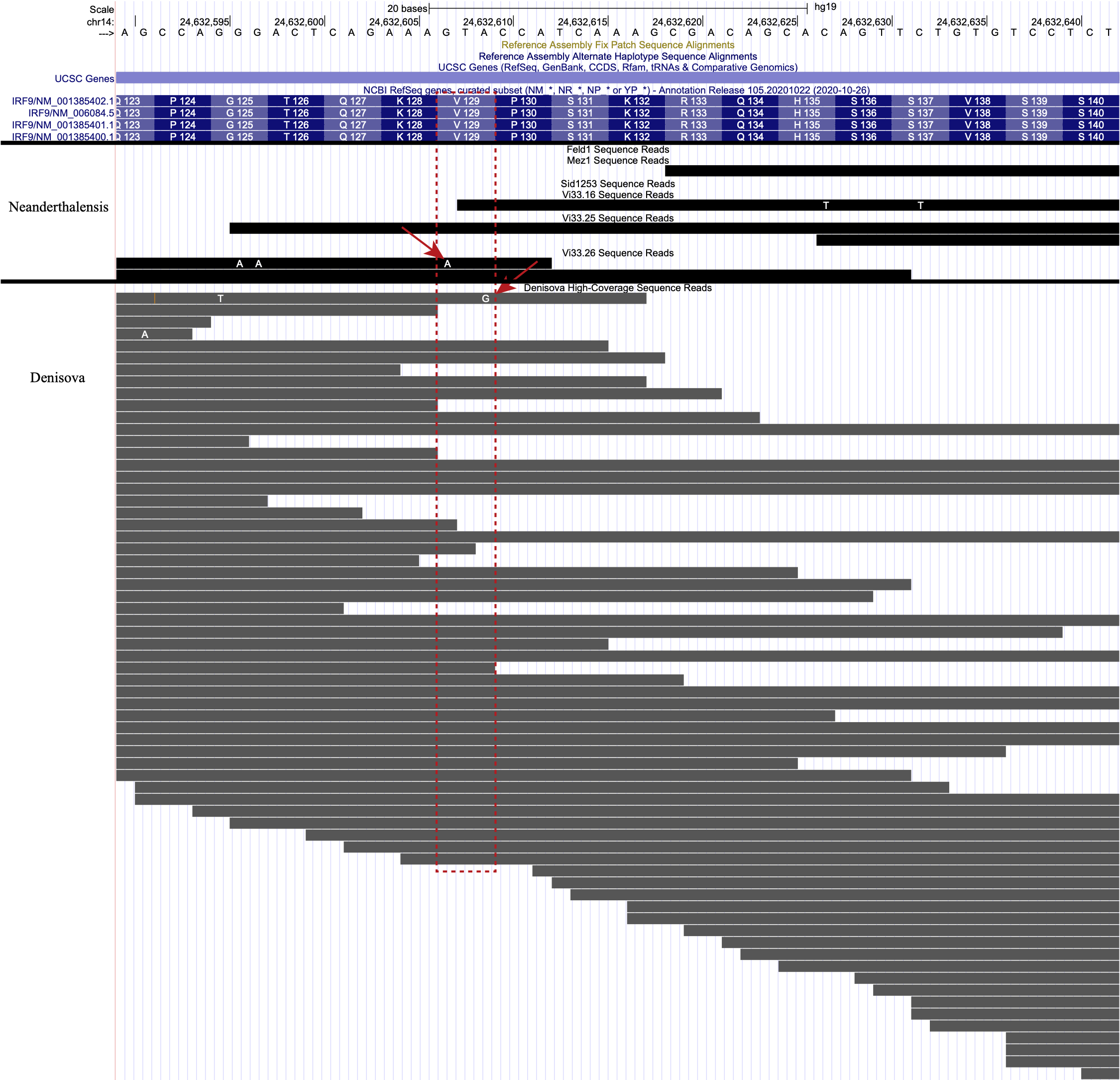

Supplement: Supplementary file 9 — Additional file 9. The UCSC mapping of Denisovan and Neanderthal reads to the human genome. The red arrows show the DNA substitutions in Denisovan and Neanderthal reads. The amino acid Val129, identical among all three Homo species, is boxed. [file 12862_2022_2088_MOESM9_ESM.png]
